# Leptin secreted from testicular microenvironment modulates hedgehog signaling to augment the endogenous function of Leydig cells

**DOI:** 10.1038/s41419-022-04658-3

**Published:** 2022-03-04

**Authors:** Himanshu Arora, Rehana Qureshi, Kajal Khodamoradi, Deepa Seetharam, Madhumita Parmar, Derek J. Van Booven, Isabelle Catherine Issa, Robert Sackstein, Dolores Lamb, Joshua M. Hare, Ranjith Ramasamy

**Affiliations:** 1 Department of Urology, Miller School of Medicine, University of Miami, Miami, FL USA; 2 The Interdisciplinary Stem Cell Institute, Miller School of Medicine, University of Miami, Miami, FL USA; 3 John P Hussman Institute for Human Genomics, Miller School of Medicine, University of Miami, Miami, FL USA; 4 Herbert Wertheim College of Medicine, Florida International University, Miami, FL USA; 5 Department of Urology, Englander Institute for Precision Medicine, Center for Reproductive Genomics, Weill Cornell Medicine, New York, NY USA; 6 Department of Medicine, Cardiology Division, Miller School of Medicine, University of Miami, Miami, FL USA

**Keywords:** Stem-cell differentiation, Translational research

## Abstract

Although testosterone deficiency (TD) may be present in one out of five men 40 years or older, the factors responsible for TD remain largely unknown. Leydig stem cells (LSCs) differentiate into adult Leydig cells (ALC) and produce testosterone in the testes under the pulsatile control of luteinizing hormone (LH) from the pituitary gland. However, recent studies have suggested that the testicular microenvironment (TME), which is comprised of Sertoli and peritubular myoid cells (PMC), plays an instrumental role in LSC differentiation and testosterone production under the regulation of the desert hedgehog signaling pathway (DHH). It was hypothesized that the TME releases paracrine factors to modulate LSC differentiation. For this purpose, cells (Sertoli, PMCs, LSCs, and ALCs) were extracted from men undergoing testis biopsies for sperm retrieval and were evaluated for the paracrine factors in the presence or absence of the TME (Sertoli and PMC). The results demonstrated that TME secretes leptin, which induces LSC differentiation and increases testosterone production. Leptin’s effects on LSC differentiation and testosterone production, however, are inversely concentration-dependent: positive at low doses and negative at higher doses. Mechanistically, leptin binds to the leptin receptor on LSCs and induces DHH signaling to modulate LSC differentiation. Leptin-DHH regulation functions unidirectionally insofar as DHH gain or loss of function has no effect on leptin levels. Taken together, these findings identify leptin as a key paracrine factor released by cells within the TME that modulates LSC differentiation and testosterone release from mature Leydig cells, a finding with important clinical implications for TD.

## Introduction

Male hypogonadism is a symptomatic clinical syndrome caused by testosterone deficiency (TD) [1]. In the European Male Ageing Study, 17.0% of men aged 40–79 years had serum testosterone levels below normal values, indicating a high prevalence of hypogonadism among middle-aged and elderly males [2]. The current standard of the cure for men with TD is lifelong exogenous testosterone therapy [3]. However, continuous exogenous testosterone supplementation negatively affects the hypothalamic-pituitary-gonadal (HPG) axis, inhibiting follicle-stimulating hormone (FSH) and luteinizing hormone (LH) production, resulting in infertility [3–8]. Consequently, there is a need to study ways to increase serum testosterone while simultaneously preserving the HPG axis and fertility.

The testis is comprised of Leydig cells, Sertoli cells, peritubular myoid cells, and germ cells. Together these cell types perform two important functions—spermatogenesis and testosterone production [9–12]. Leydig cells are among the interstitial cells which produce testosterone under the pulsatile control of the pituitary luteinizing hormone (LH) [13, 14]. We showed that in the absence of Sertoli and PMCs (which together constitute the testicular microenvironment (TME)), the function and rate of differentiation of Leydig stem cells (LSCs) is severely impaired [15]. This observation has also been supported by independent studies [16], suggesting that cells within the TME release paracrine factors that are critical for stimulating the differentiation of LSCs [15, 16].

In the present study, using cells dispersed from human testicular biopsies, we found that leptin is a paracrine factor secreted by the TME that is instrumental in regulating LSC function and differentiation to ALCs. The molecular events behind leptin-mediated regulation were defined to be through the desert hedgehog signaling pathway (DHH,) which is downstream of leptin. DHH signaling is closely linked to developmental processes, tissue and stem cell maintenance [17–21], and modulates LSC differentiation and testosterone production under physiological conditions [15, 16]. Considering these findings, this study unveils paracrine factors critical for LSC function and differentiation, providing new approaches for optimizing patient outcomes for men with testosterone deficiency.

## Results

### Isolation and characterization of testicular cells from human testis biopsies

**Fig. 1:**
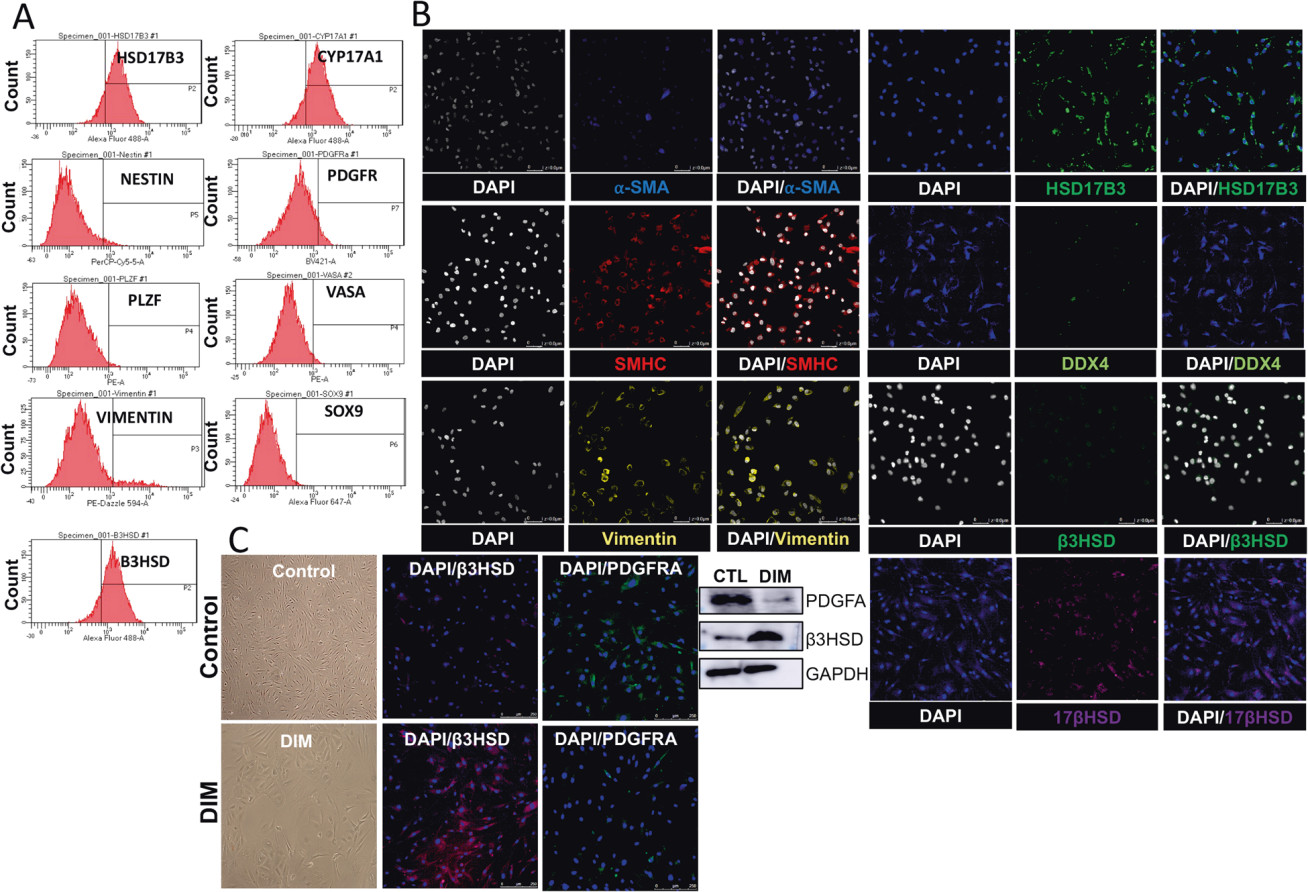
Characterization of testicular cells. **A** Flow cytometry results showing the percentage of cell types—Sertoli cells, PMCs, LSCs, ALCs, and germ cells in the TME. **B** Immunostaining results showing characterization of cell types in the TME at protein levels. **C** Transformation of LSCs to ALCs in the presence of differentiation-inducing media as indicated by the reduced expression of PDGFRα and increased expression of B3HSD.

Individual cell constituents were isolated from testis biopsies from men undergoing sperm retrieval (*n* = 18; Table 1). After cells were stabilized in culture, flow cytometry and immunostaining were performed to characterize the cell types for the presence of LSCs (PDGFRα and Nestin), ALCs (B3HSD and HSD17B3), Sertoli cells (SOX9, Vimentin), PMCs (SMHC and alpha SMA), and germ cells (PLZF, DDX4 (VASA)) (Fig. 1A, B). It is known that under normal physiological conditions, stimulation with LH modulates the testosterone secretion from Leydig cells [22]. To recapitulate the scenario in vitro, we treated the cells with HCG and evaluated the expression of B3HSD and PDGFRα. Results showed that upon stimulation with LH, the expression of B3HSD was increased and that of PDGFRα was decreased compared to unstimulated cells (Fig. 1C). Furthermore, we studied the architecture of the TME in 5 random testis biopsies with variable FSH and testosterone levels to confirm if variabilities in hormone levels could confound the TME. Results showed that the TME remained intact in all these patients (Sup Fig. 1A). Together, the data suggest that individual components of human testis could be successfully isolated, cultured, and differentiated in in vitro conditions.

**Table 1:** Details of subjects who underwent testis biopsy for sperm retrieval and whose biopsies were used to extract cells.

SNO	Age	Weight (lbs)	Height (in)	BMI	Vol (ml)	Total motility	Progressive motility	Total motile sperm Ct	Chromosome	Condition	T (ng/dL)	LH (mIU/ml)	FSH (mIU/ml)
B1	51	210	74	27.0	0.7	0	0	0	46, XY	Azoospermia	232	20.1	25.6
B2	32	193	72	26.2	NA	0	0	0	47, XXY	Azoospermia	349	NA	44
B3	36	154	69	22.7	2	0	0	0	46, XY	Azoospermia	365	2.9	16
B4	31	150	70	21.5	2.6	0	0	0	46, XY	Azoospermia	539	5.2	7.8
B5	44	175	72	23.7	NA	0	0	0	46, XY	Azoospermia	NA	NA	NA
B6	27	184	66	29.7	3.9	0	0	0	46, XY	Azoospermia/Prepubertal Cryptorchidism	470	4.7	10.9
B7	38	235	79	26.5	NA	0	0	0	46, XY	Azoospermia	736	17.6	30.1
B8	44	195	68	29.6	NA	0	0	0	46, XY	Azoospermia/Prepubertal Cryptorchidism	373	8	10
B9	36	207	72	28.1	6.5	0	0	0	46, XY	Azoospermia	486	6.1	22.8
B10	28	192	70	27.5	NA	0	0	0	46, XY	Azoospermia	271	8	10.9
B11	46	140	65	23.3	NA	0	0	0	46, XY	Azoospermia	410	NA	34.2
B12	31	160	73	21.1	2.1	0	0	0	46, XY	Azoospermia	450	1.6	3
B13	20	150	60	29.3	NA	0	0	0	46, XY	Azoospermia	1011	4.5	4.3
B14	30	185	5′9	27.32	0.7	0	0	0	46, XY	Congenital bilateral absence of the vas deferens	394	NA	1.5
B15	38	192	6′1″	25.33	0.2	0	0	0	46, XY	Congenital bilateral absence of the vas deferens	3350	4.4	7
B16	56	215	5′10″	30.85	NA	0	0	0	46, XY	Obstructive Azzospermia	NA	NA	7.3
B17	30	198	5′9	29.2	NA	0	0	0	46, XY	Azzospermia	930	9.46	5.8
B18	44	167	5′4″	28.66	3.5	0	0	0	46, XY	Azzospermia	652	4.1	9.7

### DHH is essential for the differentiation of human Leydig stem cells

**Fig. 2:**
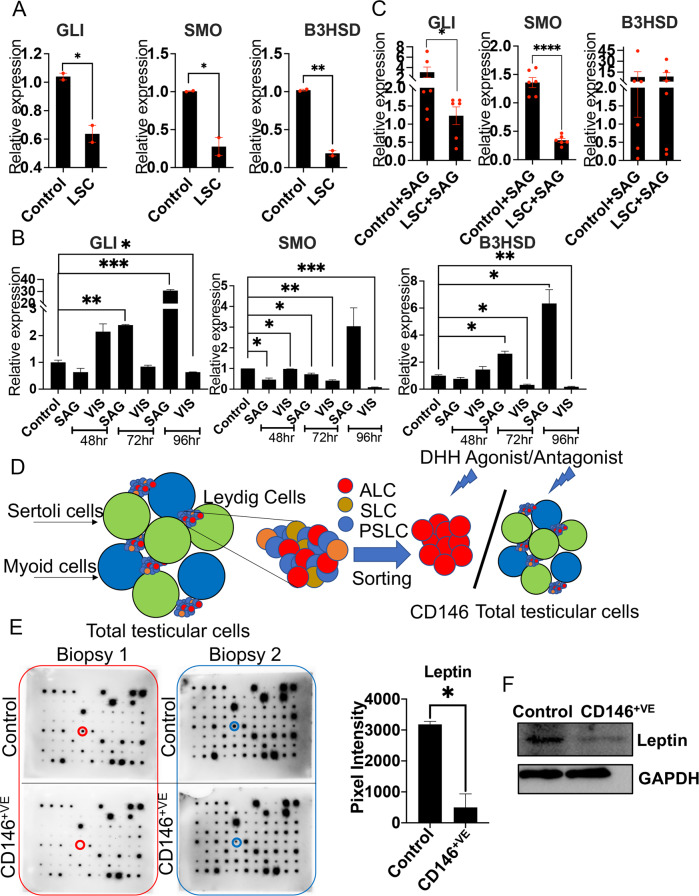
Role of DHH signaling in differentiation of LSCs. **A** Comparison of expression of GLI, SMO, and B3HSD in LSCs in the presence (control) or absence (LSC) of the TME (Sertoli and PMCs). **B** Expression of GLI, SMO, and B3HSD upon DHH agonist and antagonist treatment in LSCs in the presence of the TME in LSCs after 48, 72, and 96 h of treatment. **C** Schematic representation of steps taken for evaluating paracrine factors released by the TME to support LSC function. Here green circles represent Sertoli cells, blue-PMCs, small red-ALCs, small brown-SLCs, and small blue-PSLCs. Expression of GLI, SMO, and B3HSD upon DHH antagonist treatment in the presence (control) or absence (CD146^+^) of the TME in LSCs. **D**, **E** Results of cytokine array and **F** western blot in the presence (control) or absence (CD146^+^) of TME; leptin expression levels in these cells. Asterisks denote *p* values (e.g., *p < 0.05, ****p < 0.001).

To test whether DHH is important for differentiation of human LSCs, testosterone levels were compared in conditioned media extracted from the cellular composition (LSCs, ALCs, PMCs, and Sertoli cells) following treatment with either DHH agonist (SAG) or antagonist (Vismodigib). Testosterone levels were increased by 24.7% upon SAG (69.05 ± 3.00 ng/dL) (*p* = 0.039) and minimally (nonsignificantly) reduced by 3.2% upon Vismodigib treatment (53.52 ± 2.59 ng/dL) (*p* = 0.907) compared to untreated samples (55.31 ± 3.28 ng/dL) (Sup Fig. 1B). Furthermore, the expression of GLI, SMO (DHH markers), and B3HSD was compared in LSCs (CD146^+ve^-sorted cells) in the presence or absence of the TME (Sertoli and PMCs). CD146 is a perivascular pericyte marker, which expresses in Nestin-positive LSCs and other multipotent progenitor cells [23]. Results showed a 41.8% reduction in GLI (*p* = 0.024), a 73.5% reduction in SMO (*p* = 0.025), and a 81.4% reduction in B3HSD (*p* = 0.0019) expression in CD146^+ve^ cells compared to LSCs in the presence of the TME (Fig. 2A). Moreover, when LSCs were treated with Vismodigib, in the presence of the TME for 48, 72, and 96 h, the expression of GLI was reduced by 36.7% (*p* = 0.046), SMO by 90.76% (*p* = 0.0003), and B3HSD by 82.14% (*p* < 0.0073) after 96 h of treatment. Conversely, upon SAG treatment, the expression of GLI was increased by 2962% (*p* = 0.0009), SMO by 204% (*p* = 0.148), and B3HSD by 532.5% (*p* = 0.0351) after 96 h of treatment (Fig. 2B). Interestingly, comparing the expression of GLI, SMO, and B3HSD in CD146^+ve^ in the presence or absence of the TME after DHH agonist treatment showed that the extent of the increase in the expression of GLI was reduced by 152.4% (*p* = 0.049), of SMO by 74.8% (*p* < 0.0001), and of B3HSD nonsignificantly by 7.5% (*p* = 0.472) in CD146^+ve^ cells (Fig. 2C, D). To further validate the importance of DHH signaling on human LSC differentiation, the CD146^+^ cells were treated with SAG, and the expression of stem cell markers-Oct4, NANOG, PDGFRA, COUP-TF11, CD51, and CD90 was evaluated 48 h posttreatment. A significant decrease was observed in the expression of Oct4 (*p* = 0.02), NANOG (*p* = 0.04), CD90 (*p* = 0.005), COUP-TF11 (*p* = 0.04), and SOX4 (*p* = 0.03) (Sup Fig. 2), suggesting transformation of LSCs to ALCs in the presence of DHH agonist. Together, the results delineated that DHH signaling is important for human LSCs (HLSCs)-modulated testosterone levels and, in the absence of the TME, the impact of DHH signaling is reduced. These outcomes led us to hypothesize that the TME releases paracrine factors that are important in modulating DHH signaling-induced LSC differentiation and testosterone production.

### Paracrine factors released by the TME support LSC function

**Fig. 3:**
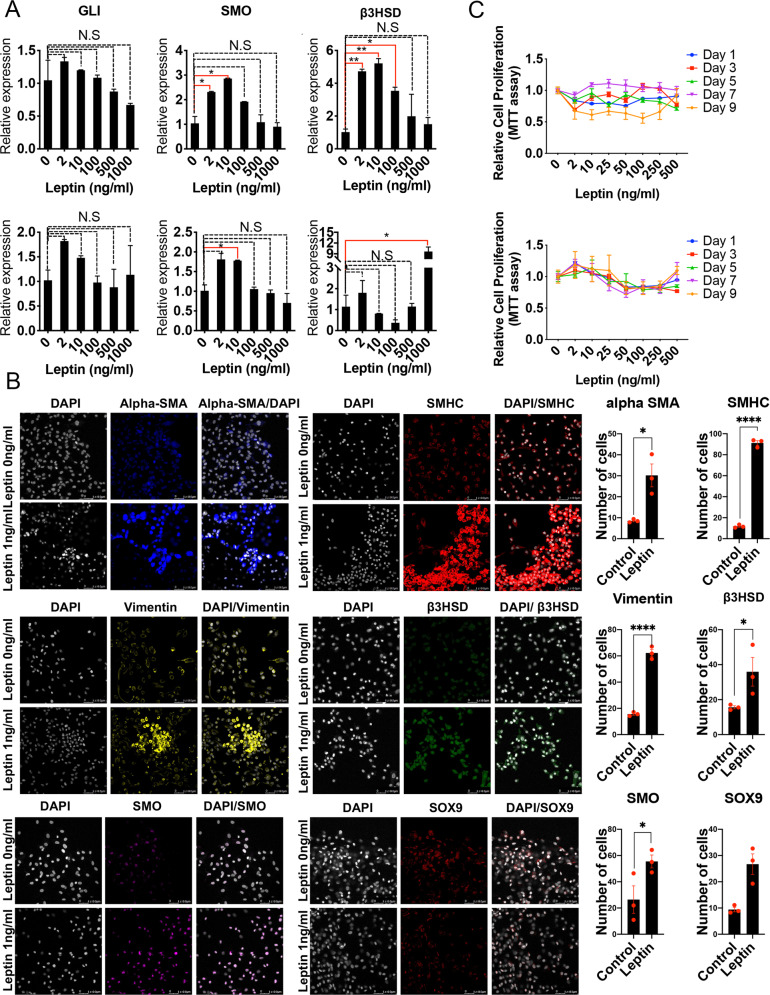
Studying Paracrine factors released by the TME. **A** Treatment of cellular composition of the testis (LSCs, ALCs, Sertoli cells, PMCs) with increasing doses of leptin at 0, 2, 10, 100, 500, and 1000 ng/ml, respectively, showing a specific pattern of expression for markers of DHH and LSC differentiation. **B** Effects of low doses of leptin on protein levels of alpha SMA, SMHC, Vimentin, and B3HSD. **C** Effects of varying doses of leptin on cell proliferation of cellular composition of the testis. Asterisks denote *p* values (e.g., *p < 0.05, ****p < 0.001).

To identify factors released by the TME, a cytokine antibody array was performed using conditioned media from LSCs in the absence (CD146^+ve^ cells) or presence of the TME (Fig. 2D). Among 80 screened cytokines, leptin was identified as one whose expression was significantly decreased (p = 0.026) in the absence of TME(Fig. 2E and Sup Fig. 3). In the cellular composition, we confirmed that leptin is expressed in LSCs, ALCs, and Sertoli cells, and the leptin receptors are expressed in Leydig cells (both LSCs and ALCs) (Sup Fig. 4A, B). Next, to evaluate the effects of leptin in conjunction with DHH signaling on LSC differentiation, the cellular composition was treated with varying doses of leptin ranging from 0 to 1000 ng/ml. Interestingly, lower concentrations (up to 2 ng/ml) of leptin increased the expression of markers of DHH signaling 122.7% for SMO (*p* = 0.045) and a nonsignificant increment of 27.6% for GLI (*p* = 0.452) and of the LSC differentiation marker (364.7% for B3HSD (*p* = 0.003)); however, these effects diminished with increasing leptin concentrations (from 10–1000 ng/ml) such that at 1000 ng/ml there was a nonsignificant 13.3% decrease in the expression of SMO (*p* = 0.712), a 36% decrease in GLI (*p* = 0.342), and a 48% increase in B3HSD (*p* = 0.388) compared to untreated cells (Fig. 3A and Sup Fig. 4C). Additionally, low dose leptin treatment increased the number of cells staining positive for alpha SMA by 253.7% (*p* = 0.01), SMHC by 674.3% (*p* < 0.0001), Vimentin for 298.5% (*p* < 0.0001), B3HSD by 130.1% (*p* = 0.034), SMO by 110% (*p* = 0.032), and SOX9 by 184% (*p* = 0.006) (Fig. 3B). This further suggested that leptin receptors are present in different cell types in the testis. Moreover, the question of whether leptin affects cell proliferation of LSCs was determined by treating the LSCs with varying concentrations of leptin ranging from 2, 10, 25, 50, 100, 250, and 500 ng/ml. Results showed suppressive (*p* > 0.05) or no effects of leptin on LSC cell proliferation (Fig. 3C). Together, the results delineated that leptin is a potent paracrine factor secreted by the TME and could induce LSC differentiation.

One important concern was if the processing of a testis biopsy and expansion of cells in culture could confound the leptin. To address this, a testis biopsy from a healthy fertile man was obtained and the protein was extracted from half of the piece within 4 h of its arrival. The remaining half was used to extract the cells [15]. Once the cells were stably cultured, the conditioned media was isolated; Aditionally in addition, the protein was extracted from the cells. Protein from two sources (biopsy and cells) and conditioned media were compared for paracrine factors. Results delineated that the leptin levels remained similar in all the conditions, whereas differences were observed for markers such as TIMP-2, TIMP-1, TGFB2, NT-3, MIF, IGFBP-2, MCP-1, IL-8, and IL-6 respectively (Sup Fig. 5). Together these results suggested that processing and cell isolation does not influence leptin.

### Leptin is upstream of DHH signaling in the TME

**Fig. 4:**
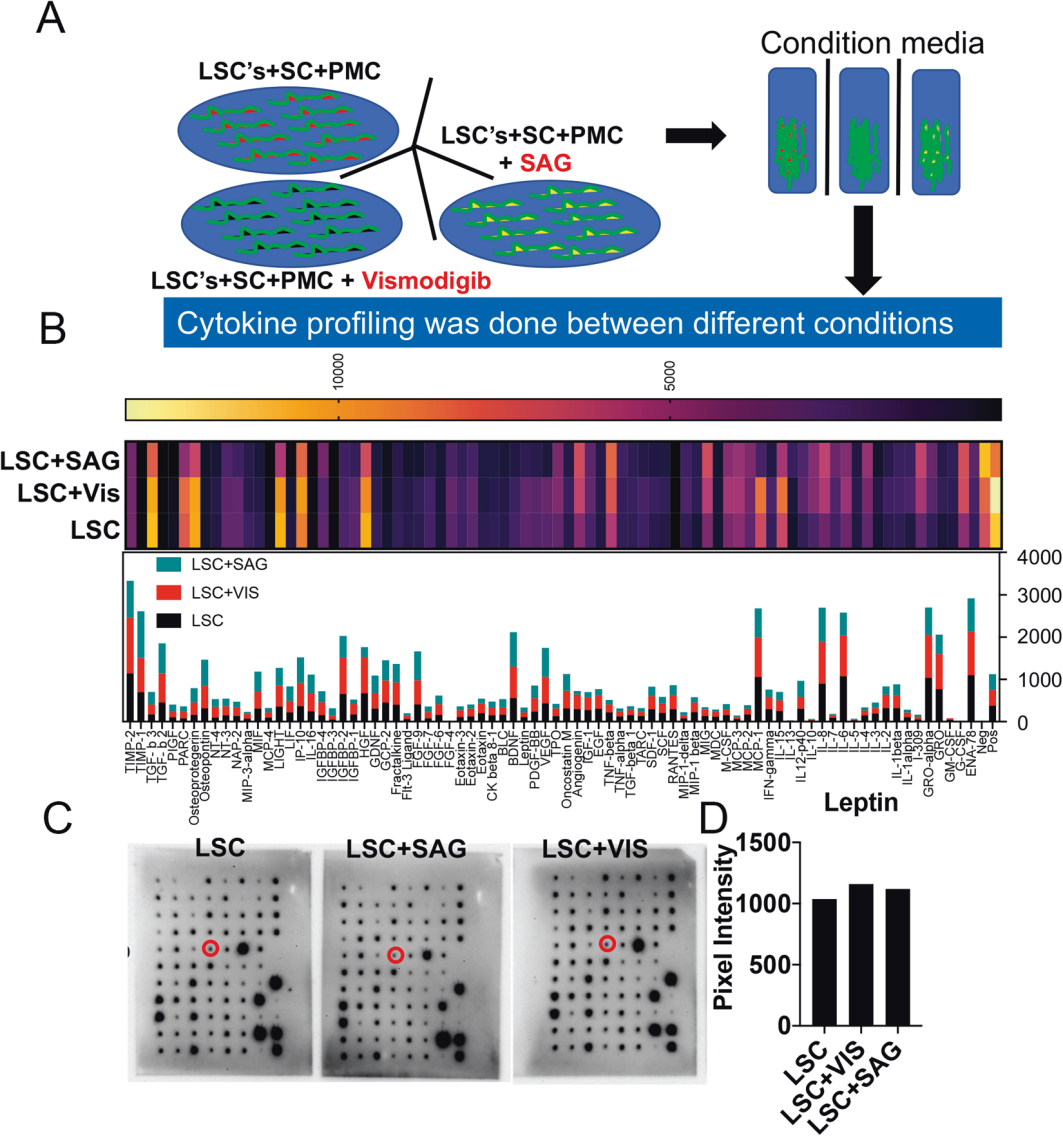
Leptin is upstream of DHH signaling in TME. Leptin is upstream of DHH signaling in TME. **A** Schematics of experiment in which LSCs were treated with DHH agonist or antagonist in the presence of the TME, followed by extraction of condition media to perform cytokine antibody array. **B** Analysis results of the cytokine array between three different conditions (control, DHH agonist treatment, and DHH antagonist treatment). **C** Blots showing cytokine array with three experimental conditions. **D** Quantification of Leptin expression levels between the three conditions.

Next, to study if the effects of leptin on DHH signaling are unidirectional or bidirectional, a cytokine antibody array was performed using condition media from LSCs treated with DHH agonist (SAG) or antagonist (Vismodigib) (Fig. 4A). No effects on the overall leptin levels were observed upon DHH signaling inhibition or induction (with a 1.6% increase in leptin upon DHH inhibition and a 2.4% increase with DHH induction) (Fig. 4B, C). However, as shown in Sup Fig. 1B, DHH agonist (SAG) and antagonist (Vismodigib) have direct impacts on testosterone levels (testosterone levels increased by 24.7% upon SAG and reduced by 3.2% upon Vismodigib treatment compared to untreated samples). Together, these results suggested that leptin is upstream of DHH signaling.

**Fig. 5:**
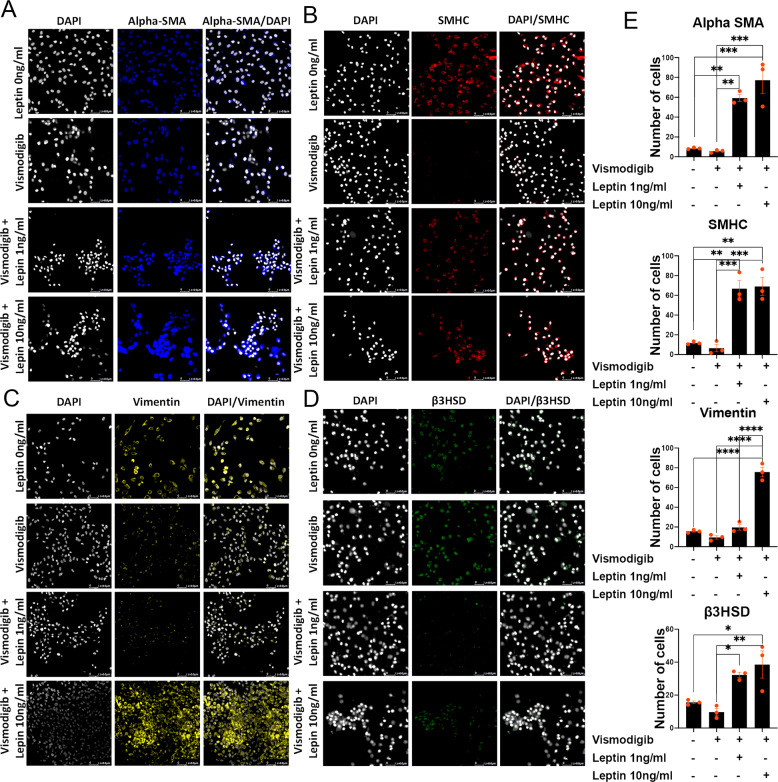
Leptin influences LSCs through DHH signaling. Leptin augments LSCs through DHH signaling. Cells (LSCs, ALCs, Sertoli cells, PMCs) were treated with Vismodigib (DHH antagonist) in the presence of Leptin (1 or 10 ng/ml). Immunostaining showing expression of **A** alpha SMA, **B** SMHC, **C** Vimentin, and **D** B3HSD upon post-Vismodigib and leptin treatment. **E** Quantification of the experimental data to compare the effects of treatment. Asterisks denote *p* values (e.g., *p < 0.05, ****p < 0.001).

To delineate that leptin augments LSCs through DHH signaling, LSCs were treated with leptin at 1 ng/ml or 10 ng/ml alone or in the presence of Vismodigib. Forty-eight hours posttreatment, the expression of alpha SMA, SMHC, Vimentin, and B3HSD was checked at the protein levels. Results showed that expression of alpha SMA was nonsignificantly reduced by 33% (*p* = 0.772), SMHC by 44.5% (*p* = 0.936), Vimentin by 40% (*p* = 0.489), and B3HSD by 38% (*p* = 0.498) (Fig. 5A–E). Leptin treatment bypassed the inhibitory effects of Vismodigib and induced the expression of alpha SMA by 594% (*p* = 0.003), SMHC by 467% (*p* = 0.001), Vimentin nonsignificantly by 106% (*p* = 0.100), and B3HSD nonsignificantly by 25.2% (*p* = 0.789) (Fig. 5A–E). Importantly, leptin treatment at a higher concentration (10 ng/ml), which generally poses an inhibitory effect on LSC differentiation, showed compensatory effects and thus was able to induce the expression of alpha SMA by 806% (*p* = 0.0005), SMHC by 486% (*p* = 0.001), Vimentin by 147% (*p* = 0.023), and B3HSD by 385% (*p* < 0.0001) (Fig. 5A–E and Sup Fig. 6), suggesting that leptin augments LSC through DHH signaling.

Furthermore, to evaluate the functional significance of the leptin and DHH signaling with respect to different cell types in the TME, the Sertoli cells, LSCs, and ALCs were sorted and then treated with DHH agonist (SAG) and antagonist (Vismodigib), in the presence or absence of increasing doses of leptin for 48 h. Posttreatment, the cells were used to evaluate the impact of leptin-DHH treatments on cell proliferation by MTT assay (Sup Fig. 7A, B). Results delineated that there is minimal to no impact of leptin on cell proliferation upon DHH agonist (SAG) or antagonist (Vismodigib) treatment in either cell type (Sup Fig. 7C). Together, the results demonstrate that leptin is upstream of DHH signaling but does not augment cell proliferation of LSC.

### Leptin modulates LSC differentiation to ALCs

**Fig. 6:**
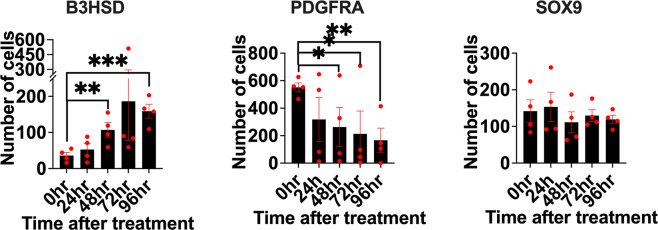
Leptin regulates LSC differentiation. Flow cytometry analysis showing the effects of increased concentration of leptin ranging from 0, 1, and 10 ng/ml on the percentage of Leydig cells, Sertoli cells, and peritubular myoid cells for 24, 48, 72, and 96 h, respectively. Asterisks denote *p* values (e.g., *p < 0.05, ****p < 0.001).

Considering the expression of ALC markers is induced upon leptin, it is likely to module LSC differentiation. To explore this, LSCs were treated with 1 ng/ml of leptin for 24, 48, 72, and 96 h, followed by flow cytometry analysis (Fig. 6). Results showed a positive shift in the total number of cells staining positive for B3HSD from 0–96 h (with a 46.5% nonsignificant increase at 24 h (*p* = 0.198), 197% increase at 48 h (*p* = 0.008), 418% increase at 72 h (*p* = 0.108), and 341% increase at 96 h (*p* = 0.0006)) when treated with 1 ng/ml of leptin, suggesting that leptin increases the differentiation to ALCs at low concentration. For PDGFRα, it was found that leptin treatment induces a negative shift in the total number of cells staining positive for PDGFRα from 0–96 h (with a 42.3% nonsignificant decrease at 24 h (*p* = 0.100), 52% decrease at 48 h (*p* = 0.047), 61% decrease at 72 h (*p* = 0.046), and 70% decrease at 96 h (*p* = 0.003)), supporting the previous observation that leptin increases the transformation of LSCs to ALCs. Furthermore, it was found that there is no overall impact (*p* > 0.05) of low doses of leptin treatment on the total number of cells staining positive for SOX9 (with an 8% nonsignificant increase at 24 h (*p* = 0.411), 21% decrease at 48 h (*p* = 0.249), 8% decrease at 72 h (*p* = 0.371), and 15% decrease at 96 h (*p* = 0.260)) and Nestin (with a 7% increase at 24 h (*p* = 0.130), 24% decrease at 48 h (*p* = 0.014), 3.4% increase at 72 h (*p* = 0.244), and 2.7% increase at 96 h (*p* = 0.370)), suggesting that leptin treatment might not have a direct impact on sperm production.

To further evaluate the role of leptin in modulating LSC differentiation, the leptin receptors (LEPR) were inhibited (using siRNA) before treating the cells with leptin (1 ng/ml), (Sup Fig. 8A). The changes in the population of TME cells were evaluated at 24, 48, 72, and 96 h using flow cytometry. Results showed that there was no overall impact (*p* > 0.05) of leptin treatment upon LEPR inhibition on the total number of cells staining positive for B3HSD (with a 40.7% overall increase in cell population (*p* = 0.434) compared to a 13.1% increase (*p* = 0.887) without leptin treatment), PDGFRα (with a 5.8% overall decrease in cell population (*p* = 0.586) compared to a 2.8% increase (*p* = 0.606) without leptin treatment), SOX9 (with a 34.1% overall decrease in cell population (*p* = 0.070) compared to a 3.5% decrease (*p* = 0.445) without leptin treatment), and Nestin (with a 10.9% overall decrease in cell population (*p* = 0.13) compared to a 0.54% decrease (*p* = 0.990) without leptin treatment) (Sup Fig. 8B).

To confirm if the impacts of leptin on LSC differentiation are specific, CD146+ cells were sorted and confirmed for the presence of leptin and leptin receptors (Sup Fig. 4A, B). These cells were then exposed to siLEPR (25 pmol), followed by leptin treatment at 1l and 10 ng/ml for 24, 48, and 72 h. Flow cytometry was performed using antibodies against ALCs (B3HSD) (Sup Fig. 9A, B). Results delineated that leptin treatment did not show any impact on the differentiation of LSCs, which were exposed to LEPR inhibition (Sup Fig. 9C) therefore suggesting that the effects of leptin on LSC differentiation are specific. Furthermore, we studied the distribution of LEPR across TME architecture in five random testis biopsies with variable FSH and Testosterone levels. Results showed that LEPR was localized in the interstitium, Leydig cells, mainly in spermatocytes (Sup Fig. 9D).

### Enrichment analysis to study leptin-modulated molecular events

**Fig. 7:**
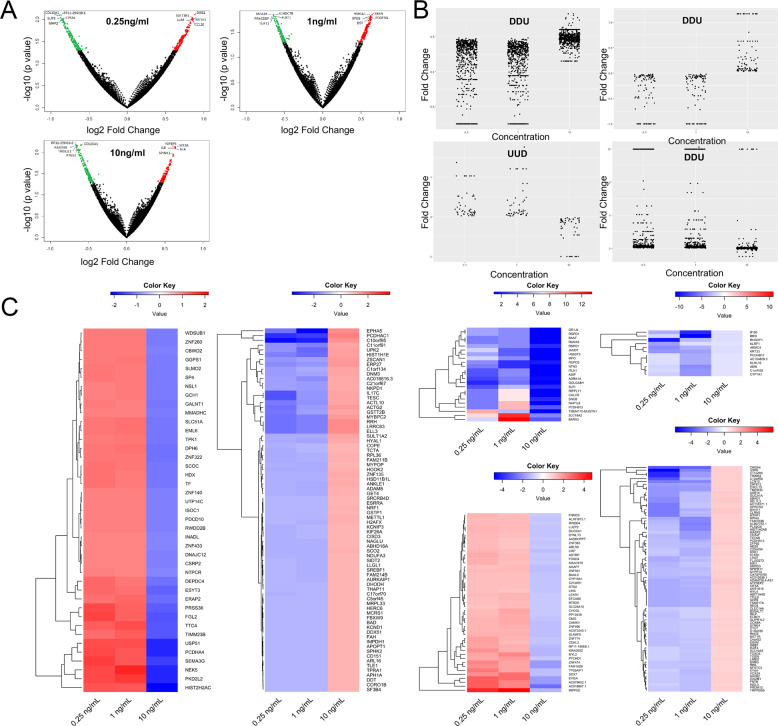
Leptin-modulated molecular events, an enrichment analysis. Enrichment analysis showing **A** Volcano plots showing significantly altered candidates upon increasing doses of leptin (0.25, 1, and 10 ng/ml) with respect to control. Green dots are those genes downregulated in the case sample and red dots are those upregulated in the case sample, with the top five genes in each direction being labeled. **B** The different cohorts represent the experimental observations UUD, UUN, DDU, and DDN, where U represents upregulated, D represents downregulated, and N represents no change. Each dot represents an individual gene within each designated pattern, with concentration plotted against fold change. **C** Heatmaps showing fold change with respect to the control for all patterns in genes across all concentrations. Heatmaps for UUN and DDN are split into two subplots showing the most differential fold change in the two major subgroups of fold change since fold change is not consistently the same across all genes.

To study the molecular events targeted by leptin to induce changes in LSC function, RNA from LSCs, postexposure to increasing concentrations of leptin were subjected to RNA sequencing. Significantly changed candidates were stratified into four cohorts based on their expression in different leptin concentrations (UUD, UUN, DDU, and DDN, where U represents upregulated, D represents downregulated, and N represents no change) (Fig. 7A, B). Protein coding candidates were selected and subjected to (Sup Table 1) hierarchical clustering. Results showed patterns suggesting no hidden subgroups within each comparison (Fig. 7C). Each of these groups were subjected to Ingenuity pathway analysis for enrichment. Results showed that these candidates were directly involved in several important canonical pathways; Leptin Signaling in Obesity, Estrogen Biosynthesis, Androgen Biosynthesis, Sonic Hedgehog Signaling, PDGF Signaling, Human Embryonic Stem Cell Pluripotency, and the pathway for the regulation of several important cytokines like IL-6, IL-8, IL-3, and IL-15 (Table 2, Sup Table 2, and Sup Figs. 10, 11A). Additionally, to highlight the leptin-induced molecular interactions that could influence DHH signaling, the candidates from four cohorts were overlaid with DHH signaling genes. Results (Sup Fig. 11B) revealed important interactions between DHH signaling and leptin-induced markers.

**Table 2:** Canonical pathways with which leptin-induced/suppressed candidates were involved.

Ingenuity canonical pathways	-log(*p* value)	Ratio	Overlaps with dataset	No overlap with dataset	Molecules
Leptin signaling in obesity	0.593	0.0811	6/74 (8%)	68/74 (92%)	ADCY2,ADCY8,MAPK3,PLCB3,PLCD1,PRKACB
Estrogen biosynthesis	2.68	0.195	8/41 (20%)	33/41 (80%)	AKR1B15,AKR1C4,CYP19A1,CYP1A1,CYP1A2,CYP2D6,CYP4X1,HSD17B3
Androgen biosynthesis	1.36	0.214	3/14 (21%)	11/14 (79%)	AKR1C4,EBP,HSD17B3
Androgen signaling	0.583	0.0735	10/136 (7%)	126/136 (93%)	CACNA1E,GTF2E1,MAPK3,POLR2C,POLR2D,POLR2F,POLR2I,PRKACB,SHBG,SRC
Sonic hedgehog signaling	0.281	0.0667	2/30 (7%)	28/30 (93%)	PRKACB,STK36
PDGF signaling	0.91	0.093	8/86 (9%)	78/86 (91%)	ERAS,INPP5D,MAPK3,RALA,SPHK1,SPHK2,SRC,TYK2
Human embryonic stem cell pluripotency	0.595	0.0741	10/135 (7%)	125/135 (93%)	BMP8A,FGFR4,NTRK3,S1PR4,SMAD1,SMAD5,SPHK1,TCF7L2,TGFB2,WNT6
IL-15 production	1.04	0.0909	11/121 (9%)	110/121 (91%)	CSK,DDR1,EPHA5,EPHB3,ERBB2,FGFR4,MST1R,NTRK3,RET,SRC,TYK2
IL-8 signaling	0.583	0.0704	14/199 (7%)	185/199 (93%)	CR2,ERAS,IRAK4,MAPK3,MPO,MYL2,NOX1,NOX3,PGF,PLD5,RAB11FIP2,RALA,RHOT1,SRC
IL-22 signaling	0.39	0.0833	2/24 (8%)	22/24 (92%)	MAPK3,TYK2
IL-6 signaling	0.362	0.064	8/125 (6%)	117/125 (94%)	ABCB1,CRP,CYP19A1,ERAS,HSPB7,IL37,MAPK3,RALA
IL-3 signaling	0.316	0.0633	5/79 (6%)	74/79 (94%)	BAD,ERAS,INPP5D,MAPK3,RALA
IL-4 signaling	0.261	0.0588	5/85 (6%)	80/85 (94%)	ERAS,INPP5D,NFATC2,RALA,TYK2
IL-10 signaling	0	0.0435	3/69 (4%)	66/69 (96%)	FCGR2B,IL37,TYK2
IL-17 signaling	0	0.05	4/80 (5%)	76/80 (95%)	CRP,ERAS,MAPK3,RALA
IL-15 signaling	0	0.0563	4/71 (6%)	67/71 (94%)	ERAS,MAPK3,RALA,TYK2
IL-1 signaling	0	0.0549	5/91 (5%)	86/91 (95%)	ADCY2,ADCY8,IRAK4,MYD88,PRKACB
IL-2 signaling	0	0.0492	3/61 (5%)	58/61 (95%)	ERAS,MAPK3,RALA
IL-7 signaling pathway	0	0.0513	4/78 (5%)	74/78 (95%)	BAD,FOXO4,FOXO6,MAPK3
IL-23 signaling pathway	0	0.0227	1/44 (2%)	43/44 (98%)	TYK2
Sperm motility	1.05	0.0814	18/221 (8%)	203/221 (92%)	CNGA4,CSK,DDR1,EPHA5,EPHB3,ERBB2,FGFR4,MST1R,NPPB,NTRK3,PLA2G12A,PLA2G5,PLCB3,PLCD1,PRKACB,RET,SRC,TYK2

Furthermore, during the differentiation of LSCs to ALCs, signaling pathways such as DHH, Activin, TGFβ, PDGF, Notch, and WNT signaling plays a key role [24]. Among these, DHH and Activin signaling are known to increase the differentiation of LSCs to ALCs, whereas TGFβ, PDGF, Notch, and WNT signaling pathways are known to potentially induce de-differentiation of ALCs to LSCs [25, 26]. To study how leptin (1 ng/ml) could modulate changes through these signaling pathways, we extracted the key markers associated with each of these pathways and studied how (if) leptin treatment altered their expression using RNA sequencing data (Sup Table 3). Results showed that low concentrations of leptin were able to increase the expression of differentiation, inducing DHH and Activin signaling markers, and reduce the expression of de-differentiation, inducing WNT, TGFb, Notch, and PDGF signaling markers (Sup Table 3 and Sup Fig. 12). Together, the results suggest that leptin is important to modulate molecular events that affect Leydig cell regulation.

## Discussion

Found in the interstitial space of the testis, ALCs are downstream of the HPG regulatory axis [27–29] and are the main source of testosterone production in males [30–32]. LH stimulates testosterone synthesis by ALCs, and the resultant increase in testosterone exerts negative feedback on the pituitary to decrease LH [27]. In our previous study, we defined the potential of subcutaneously autografting LSCs in combination with the TME (Sertoli and PMCs) to increase serum testosterone without affecting the HPG axis (in murine models). These studies further revealed that DHH signaling is an essential modulator that is critical for LSC differentiation to the testosterone-producing ALCs in grafts [16, 21]. Furthermore, autograft survival and testosterone production are impaired in the absence of the TME (Sertoli and PMCs), suggesting the relevance of the TME on LSC function and the understudied role of paracrine factors released from the TME in the regulation of LSCs.

The present study used cells extracted from human testicular biopsies to explore the paracrine factors that could play an instrumental role in regulating LSC differentiation and testosterone production. Results revealed that leptin is the paracrine factor, which is released by the TME and plays an important role in LSC differentiation. Leptin is a 16-kDa protein produced primarily by adipose tissue which exerts a significant influence on reproduction and fertility in mammals [33]. The normal reproductive function requires an adequate amount of leptin that regulates these functions via central (HPG) and testicular steroidogenesis [34]. Leptin exerts a rapid and dose-dependent inhibition of hCG-stimulated testosterone production in primary cultures of rat Leydig cells [35]. This effect of leptin is associated with the attenuation of androstenedione levels and an increased level of the precursor molecules progesterone, 17-OH progesterone, and pregnenolone [35]. Moreover, leptin reduces the expression of key elements of the steroidogenic machinery such as SF-1, StAR, and P450scc [36]. Germ cells may exert paracrine control of human Leydig cell steroidogenesis via leptin secretion [37]. At the hypothalamic and pituitary levels, leptin increases GnRH, LH, and FSH production, which leads to a downstream increase in serum testosterone levels [38, 39]. At the level of the testes, leptin decreases testosterone secretion by directly inhibiting testosterone synthesis via mechanisms including downregulation of STAT transcriptional activity and interfering with cAMP signaling [39]. Conversely, testosterone is also important for leptin regulation, and it can inhibit leptin secretion by acting on white adipose tissue [39]. In infertile men with obstructive azoospermia, Sertoli cell-only syndrome, and varicocele, leptin receptor expression in Leydig cells is inversely correlated with the serum levels of testosterone. Thus, overexpression of the leptin receptor by Leydig cells appears to inhibit testosterone production in infertile men [37], suggesting that signaling transduction pathway(s) downstream of leptin exert negative control over steroidogenesis by human Leydig cells. This may explain why low levels of leptin treatment were able to increase LSC differentiation and testosterone levels in cells extracted from the testis biopsies of azoospermia men in our study.

Knowledge regarding the intracellular signaling pathways that control the development of different populations of human Leydig cells is still incomplete [40]. This study is the first to (1) use cells from human testicular biopsies to study paracrine factors critical for LSCs; (2) identify leptin as a paracrine factor and demonstrated its functional significance on LSC function and differentiation; (3) established the molecular events behind leptin-mediated LSC function and differentiation. However, the study is limited by: (1) not exploring if LSCs from men with low testosterone behave differently from men with normal testosterone, not accounting for leptin resistance and its implications on testosterone production; (2) using a method of estimating testosterone (radioimmunoassay) that has been reported to have a cross-reactivity which induces (up to a certain extent) non-specificity compared to other methods of testosterone estimation such as liquid chromatography/tandem mass spectrometry (LC-MS/MS) [41]; (3) lacking a Sertoli-Sertoli tight junction in in vitro condition [42, 43], which impacted the immunostaining of Sertoli cell marker Vimentin in comparison to in situ human testis, and (4) using biological replicates (testis biopsies) that are each different from one another in terms of testicular phenotype. In future studies, these limitations will be ameliorated to define leptin as a potential new niche-based therapy for testosterone deficiency.

In conclusion, the results from this study demonstrated the instrumental role of leptin as a paracrine factor secreted by the TME on human LSC function and differentiation. Several aspects of human Leydig cell physiology require further investigation to improve our understanding of the molecular mechanisms through which human reproduction and fertility are regulated.

## Methods

### Human sample

A total of 18 testis biopsies from men undergoing sperm retrieval (microTESE) were obtained. The IRB protocol was approved by the University of Miami Miller School of Medicine, Miami, FL (protocol no. 20150740). A majority of these men were azoospermic, with average (avg) serum testosterone levels of 474.33 ng/dL, avg LH levels of 7.87 mIU/ml, avg FSH levels of 18.3 mIU/ml, avg body weight of 180.38 lbs, avg BMI of 25.86, and avg age of 35 years. The complete details of the patients can be found in Table 1. The protocol for LSC isolation has been described previously [12]. Briefly, interstitial cells from the testes were dissociated from the seminiferous tubules by treatment with 1 mg/ml trypsin followed by collagenase (collagenase D; Roche Molecular Biochemicals) treatment in DMEM for up to 1 h at 34 °C with shaking. The separated cells were filtered through two layers of 70-μm pore-size nylon mesh, centrifuged at 250×*g*, and resuspended in DMEM. The cells were washed with PBS, centrifuged at 250×*g*, resuspended in phenol red-free 1:1 DMEM: F12, and plated for cell culture.

### Expansion and characterization of cells

LSCs from all 13 testis biopsies were cultured in an expansion medium (EM) adapted for embryonic stem cell culture with little modification [44]. LSCs were maintained in this medium for at least 14 days. To induce differentiation, these cells were replated in a new medium containing differentiation-inducing factors. Differentiation-inducing medium (DIM) included DMEM containing 2 mM l-glutamine and 15 mM HEPES (Thermo Fisher Scientific), 10% fetal bovine serum (FBS) (Thermo Fisher Scientific), 1% penicillin/streptomycin (Thermo Fisher Scientific), 2 ng/ml EGF (Sigma-Aldrich), 1 ng/ml LIF (Sigma-Aldrich), 10 ng/ml PDGF-AA (Sigma-Aldrich), 1 μM dexamethasone (Sigma-Aldrich), and the insulin transferrin selenium (ITS) supplement (Thermo Fisher Scientific).

### Flow cytometry

Flow cytometry was performed using cells from three independent biopsies. For this, cells were washed with FACS buffer (eBioscience^TM^ 00-4222-57) two times. Cells in one tube (unstained) were resuspended in 100 ul FACS buffer. Cells in the second tube (stained) were fixed with 100 ul of antibody + FACS buffer mix, according to recommended concentrations, and left for 30 min at 4 °C. Cells in both tubes were again washed two times with FACS buffer and then fixed with BD Cytofix/Cytoperm™ (Ct No. 554714) Fixation/Permeabilization solution for 20 min at 4 °C. After washing, cells in the unstained tube were resuspended in 100 ul of perm wash buffer, while conjugating antibodies against PDGFRα, B3HSD, SOX9, Nestin, Vimentin, and PLZF were added to the stained tube. Cells were incubated for 30 min at 4 °C and then washed two times with perm wash buffer. Finally, cells were fixed with 500 ul of 4% PFA (paraformaldehyde) and stored at 4 °C before analyzing using FACS.

### Immunohistochemistry and fluorescence staining

For immunohistochemical staining, tissue sections of the graft (both experimental and negative control) were stained with hematoxylin and eosin. A genitourinary pathologist (who was blinded to the samples) independently verified the presence of LCSs under 10x and 60x magnification. To confirm the presence of different cell types, fluorescence staining was performed on the three testis biopsies that were used for flow cytometry using (1) antibody against B3HSD (sc-30820) followed by Alexa Fluor 488 dye (Thermo Fisher Scientific); (2) anti-alpha SMA antibody (AB5694) followed by Alexa Fluor 488 dye; (6) anti-smooth muscle Myosin heavy chain 11 mAb (SMHC11) followed by Alexa Fluor 568 dye; (7) anti-Vimentin mAb (ab45939) followed by Alexa Fluor 568 dye. All samples were assessed under a fluorescence microscope (Leica Microsystem, Wetzlar, Germany) at 60x. Images were acquired using MetaMorph version 4.6 (Molecular Devices, Sunnyvale, CA, USA) (information on antibodies can be found in Resource Table).

### Assay of testosterone concentration

Condition media was collected from cells (LSCs and CD146 sorted cells) from three random testis biopsies, pre- and posttreatment with either DHH agonist (SAG), Vismodigib, or leptin in the absence or presence of Vismodigib. All hormone assays were measured at the University of Virginia (UVA) Center for Research and Reproduction Ligand Assay and Analysis Core Laboratory (Charlottesville, VA). Testosterone was measured using a commercially available solid-phase RIA kit (Coat-a-Count Total Testosterone Kit; Siemens Medical Solutions Diagnostics, Tarrytown, NY, USA). Testosterone assay sensitivity = 10 ng/dl; intra-assay coefficient of variation (CV) = 5.0%; inter-assay CV = 8.2%. To avoid variability, an equal number of cells were seeded and were allowed an equal amount of time to expand before extracting the conditioned media for testosterone estimation. Reportable range = 10.0–1600.0 ng/dL; Rodent QC1 (32.0–60.0 ng/dL), Rodent QC 2 (122–228 ng/dL), Rodent QC 3 (510.0–950.0 ng/dL).

### RNA preparation and quantitative real-time PCR

Total RNA was isolated from cells using the TRIzol method. This was followed by inducing reverse transcription to complementary DNA using High-Capacity cDNA Reverse Transcription Kits (Applied Biosystems, USA) according to the manufacturer’s protocol. The quantitative RT-PCR for indicated genes was performed in TaqMan Universal PCR Master Mix (Applied Biosystems, USA). Quantitation of mRNAs was performed using Applied Biosystems™ TaqMan™. Gene expression assays were performed according to the manufacturer’s protocol. Samples were analyzed using the BIORAD sequence detection system. All PCRs were performed in triplicate, and the specificity of the reaction was determined by melting curve analysis at the dissociation stage. The relative quantitative method was used for the quantitative analysis. The calibrator was the averaged ΔCt from the untreated cells. The endogenous control was glyceraldehyde 3-phosphate dehydrogenase (GAPDH). All the experiments suggested in section 3 were repeated twice (primer sequences are given in Resource Table).

### Cytokine antibody array

Condition media was extracted from LSCs from two random testis biopsies in the presence of absence (CD146^+ve^) of the TME, with cells treated with DHH agonist (SAG) or antagonist (Vismodigib) etc. After quantification using Bradford assay, 4 mg/ml protein were screened using RayBio Human Antibody Array C Series 1000 (RayBiotech, Norcross, GA, USA) according to the manufacturer’s instructions. The blots were analyzed using ImageJ software (National Institutes of Health, Bethesda, MD, USA). A total of 80 molecules were selected for detection: Positive control, Negative control, ENA-78 (CXCL5), G-CSF, GM-CSF, GRO a/b/g, GRO alpha (CXCL1), I-309 (CCL1), IL-1 alpha (IL-1 F1), IL-1 -beta (IL-1 F2), IL-2, IL-3, IL-4, IL-5, IL-6, IL-7, IL-8 (CXCL8), IL-10, IL-12 p40/p70, IL-13, IL-15, IFN-gamma, MCP-1 (CCL2), MCP-2(CCL8), MCP-3 (CCL7), M-CSF, MDC (CCL22), MIG (CXCL9), MIP-1 beta (CCL4), MIP-1 delta, RANTES (CCL5), SCF, SDF-1 alpha, TARC (CCL17), TGF beta 1, TNF alpha, TNF beta (TNFSF1B), EGF, IGF-1, Angiogenin, OSM, TPO, VEGF-A, PDGF-BB, Leptin, BDNF, BLC (CXCL13), Ck beta 8-1 (CCL23), Eotaxin-1 (CCL11), Eotaxin-2 (CCL24), Eotaxin-3 (CCL26), FGF-4, FGF-6, FGF-7 (KGF), FGF-9, FLT-3 Ligand, Fractalkine (CX3CL1), GCP-2 (CXCL6), GDNF, HGF, IGFBP-1, IGFBP-2, IGFBP-3, IGFBP-4, IL-16, IP-10 (CXCL10), LIF, LIGHT (TNFSF14), MCP-4 (CCL13), MIF, MIP-3 alpha, NAP-2 (CXCL7), NT-3, NT-4, OPN (SPP1), OPG (TNFRSF11), PARC, PLGF, TGF beta 2, TGF beta 3, TIMP-1, TIMP-2, and Positive control.

### Cell proliferation assay

Cells from three random testis biopsies were seeded in 96-well plate in quadruplets and were treated with varying doses of leptin (Sigma-Aldrich) ranging from 2, 10, 25, 50, 100, 250, and 500 ng/ml. MTT (3-(4,5-dimethylthiazole)-2,5-diphenyltetrazolium bromide) assay reagents (EMD Millipore) were added, and the absorbance was measured at 562 nm after 0, 3, 5, 7, and 9 days, following standard protocol. All the experiments suggested in section 4 were repeated twice.

### RNA sequencing

FastQ files were put through the FastQC program to check for the quality of reads. Acceptable reads were then put through the adapter trimming software TrimGalore, which runs off the cutadaptpython package. Reads were then aligned against the hg19 genome with the STAR RNAseq aligner and quantified against the GENCODE v19 database at the same time. Post-alignment QC stats were run through PicardTools to check for ribosomal contented transcriptome alignment percentages. After alignment, differential expression comparisons were made with the DESeq2 R package. Due to the lack of replicates, results from this comparison included nominal *p* value significance instead of FDR corrected *p* values, as well as normalized expression values. The normalized expression values were then put through a custom pipeline to determine patterns. First, minimum, maximum, and mean values were calculated from the four separate samples for each gene. Then, a standard deviation was calculated across the samples.

Using 0.75X standard deviation, a range was formed using a maximum value of the baseline concentration expression value +0.75X standard deviation and a minimum value of the baseline concentration expression value –0.75X standard deviation. Different standard deviations were tested to check different specificity and sensitivity levels and 0.75X was determined to give the most viable results. Samples were then lined up according to concentration from 0 to 2. Comparisons to the baseline concentration classifications were then calculated based on comparing the expression value to the calculated range of values. Those that were greater than the max range value were determined to be an upward change of expression in a given sample, and those that were less than the min range value were determined to be a downregulation expression change in a given sample. Based on these patterns of change, we then calculated the patterns that were of most interest. Ingenuity pathway analysis (QIAGEN) was used to enrich the selected markers from each section with respect to their involvement in molecular signaling networks.

### Statistical analysis and sample size calculation

GraphPad Prism (GraphPad Software) was used for statistical analysis. All data were presented as the means ± SEM. The statistical significance between the two groups was estimated by an unpaired two-tailed *t*-test. Multiple group comparisons were performed using a one-way analysis of variance with the least significant difference test. In all cases, *p* < 0.05 was considered statistically significant.

## Supplementary information

Supplementary Material 1

Supplementary Material 2

AJ CHECKLIST

## Data Availability

All data needed to evaluate the conclusions in the paper are present in the paper. Additional data related to this paper may be requested from the corresponding author.
