# Supplementary material for: Leptin secreted from testicular microenvironment modulates hedgehog signaling to augment the endogenous function of Leydig cells
Source: Cell Death Dis. 2022 Mar 4;13(3):208. doi: 10.1038/s41419-022-04658-3 (PMC8897450; doi:10.1038/s41419-022-04658-3)
Supplement: Supplementary file 1 — Supplementary Material 1 [file 41419_2022_4658_MOESM1_ESM.pdf]

SI Appendix

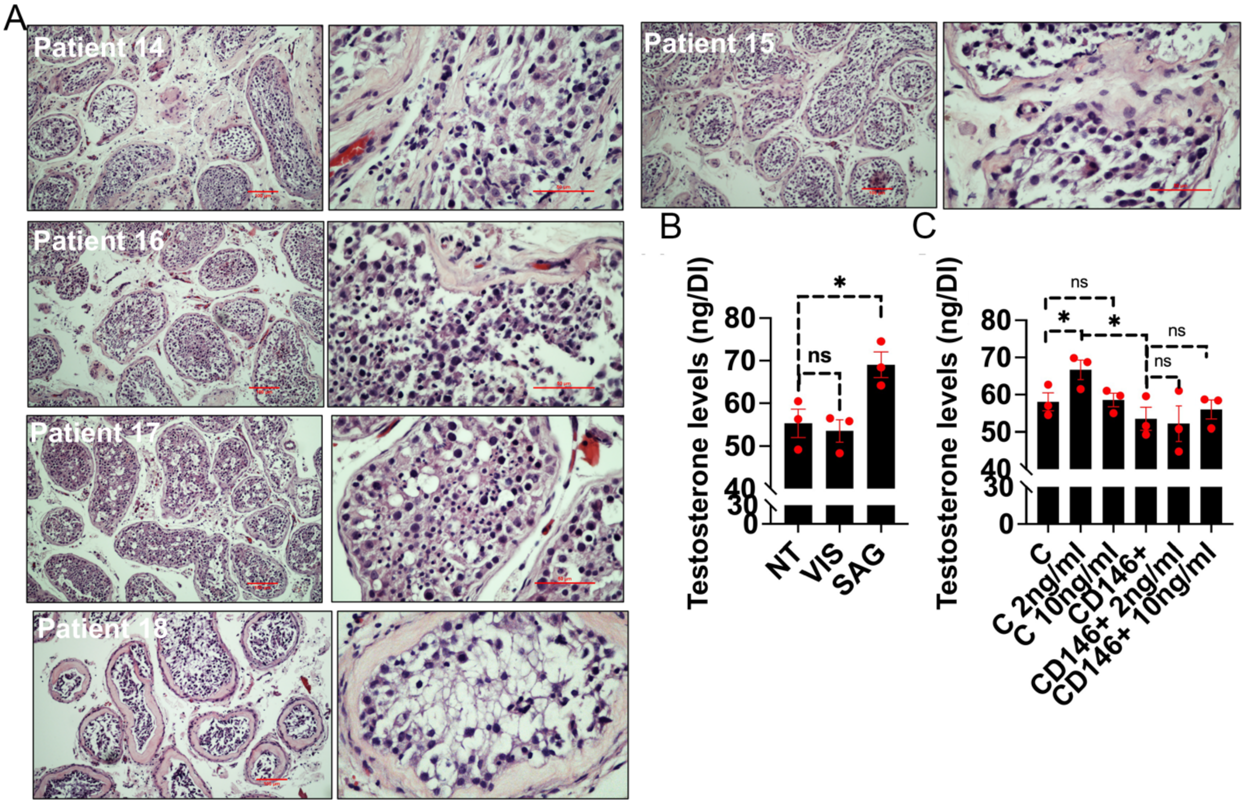

Supplementary Figure 1. A) Representative histology images of patient 14 (FSH 1.5), 15 (FSH 7), 16 (FSH 7.3), 17 (FSH 5.8), 18 (FSH 9.7), testis stained with hematoxylin and eosin. B) Testosterone levels from condition media extracted from LSCs treated with DHH antagonist (Vismodigib) or agonist (SAG). C) Testosterone levels from condition media extracted from LSCs treated with different concentrations of leptin (0,2 or 10ng/DL) in the presence or absence ($CD146^{+VE}$) of TME.

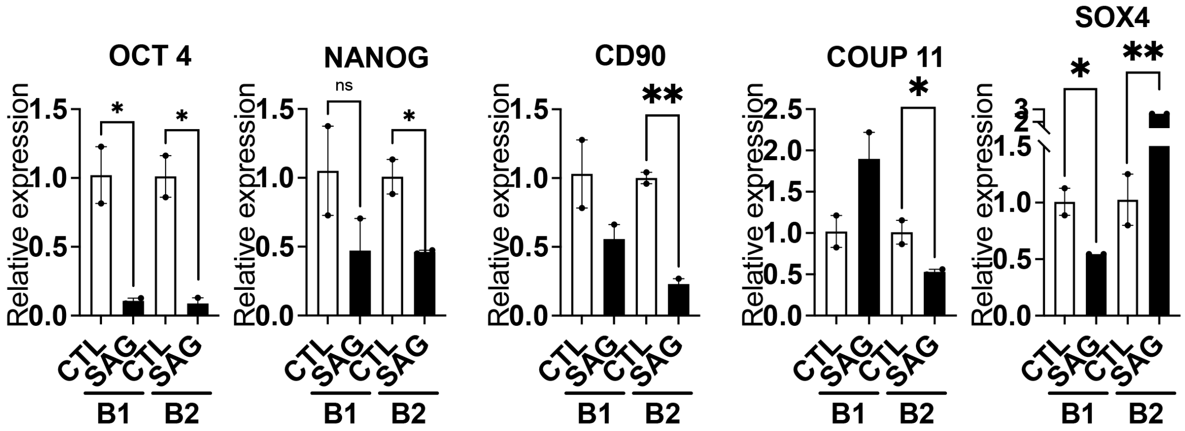

Supplementary Figure 2. Expression of OCT4, NANOG, CD90, COUP-TFII, SOX4 in sorted LSCs (CD146$^+$) from two independent testis biopsies (B1 and B2), treated with DHH agonist SAG for 48 hours.

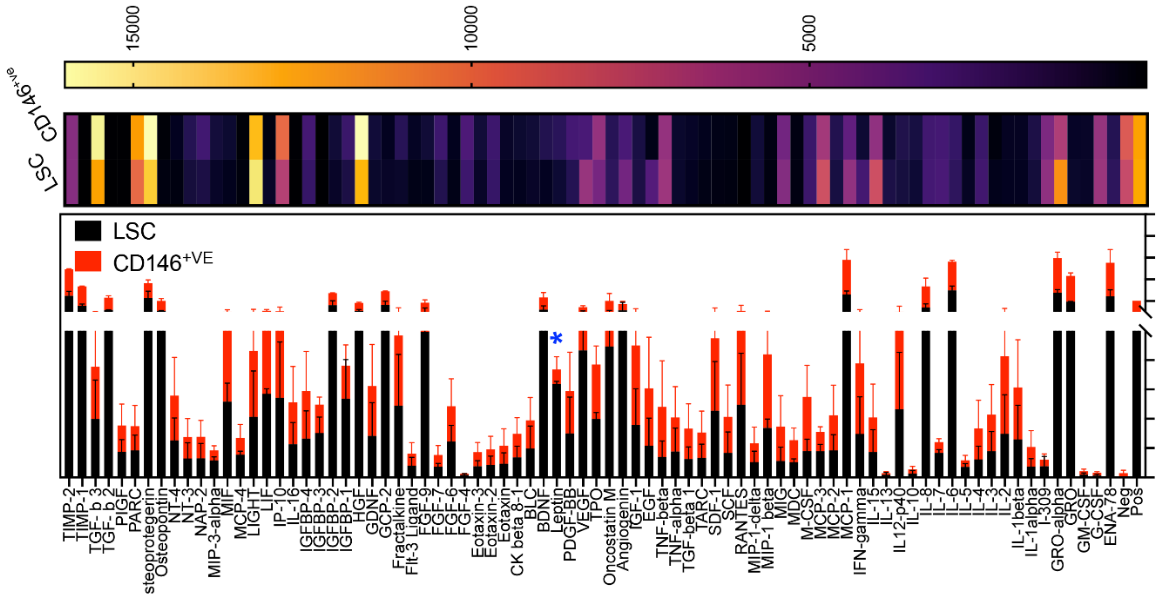

Supplementary Figure 3: Quantification data showing the different levels of cytokines in an antibody array performed using condition media from LSCs in the presence or absence ($CD146^{+VE}$) of TME.

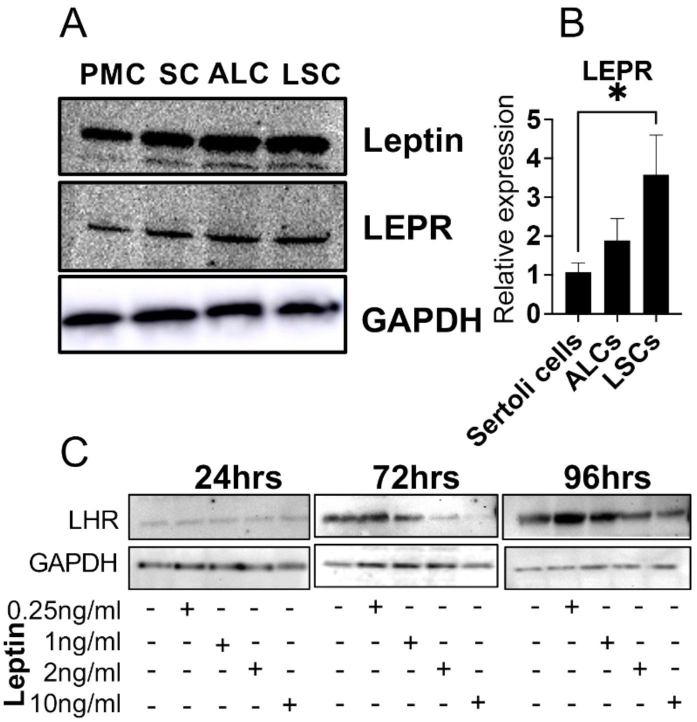

Supplementary Figure 4: Evaluation of A) Leptin and B) LEPR expression in LSCs, ALCs, and Sertoli cells in the cellular composition. C) Impacts of increasing doses of Leptin, ranging from 0.25, 1, 2, and 10ng/ml, on the protein levels expression of LHR at 24, 72 and 96 hours, respectively.

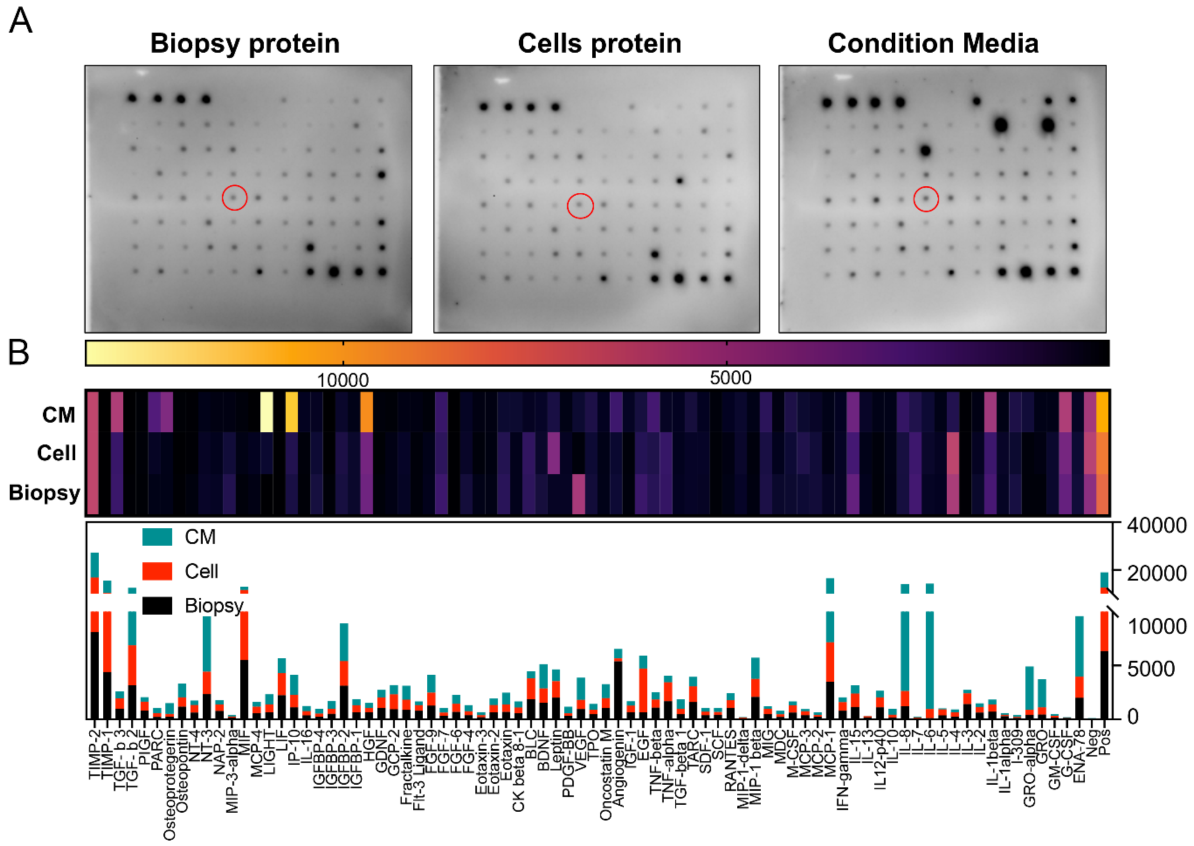

Supplementary Figure 5. A) Impact of different sample sources (direct biopsy, cellular proteins, condition media) over the levels of cytokines. B) Results showing quantification of data from cytokine antibody array.

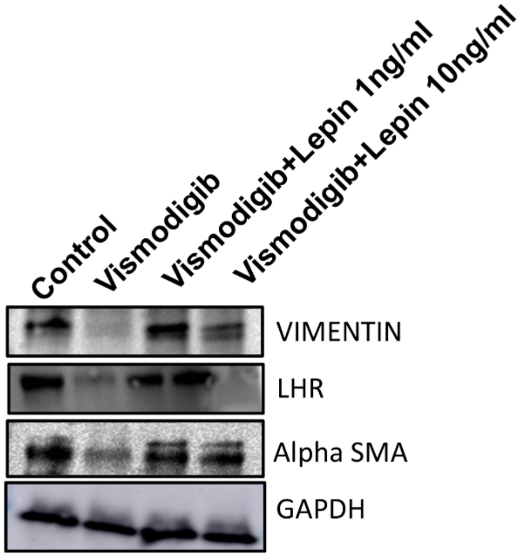

Supplementary Figure 6. Leptin augments LSCs through DHH signaling. Cells (LSCs, ALCs, Sertoli cells, PMCs) were treated with Vismodigib (DHH antagonist) in the presence of Leptin (1ng/ml or 10ng/ml). Western blot shows the expression of A) Vimentin, B) LHR, and C) alpha-SMA upon post Vismodigib and leptin treatment.

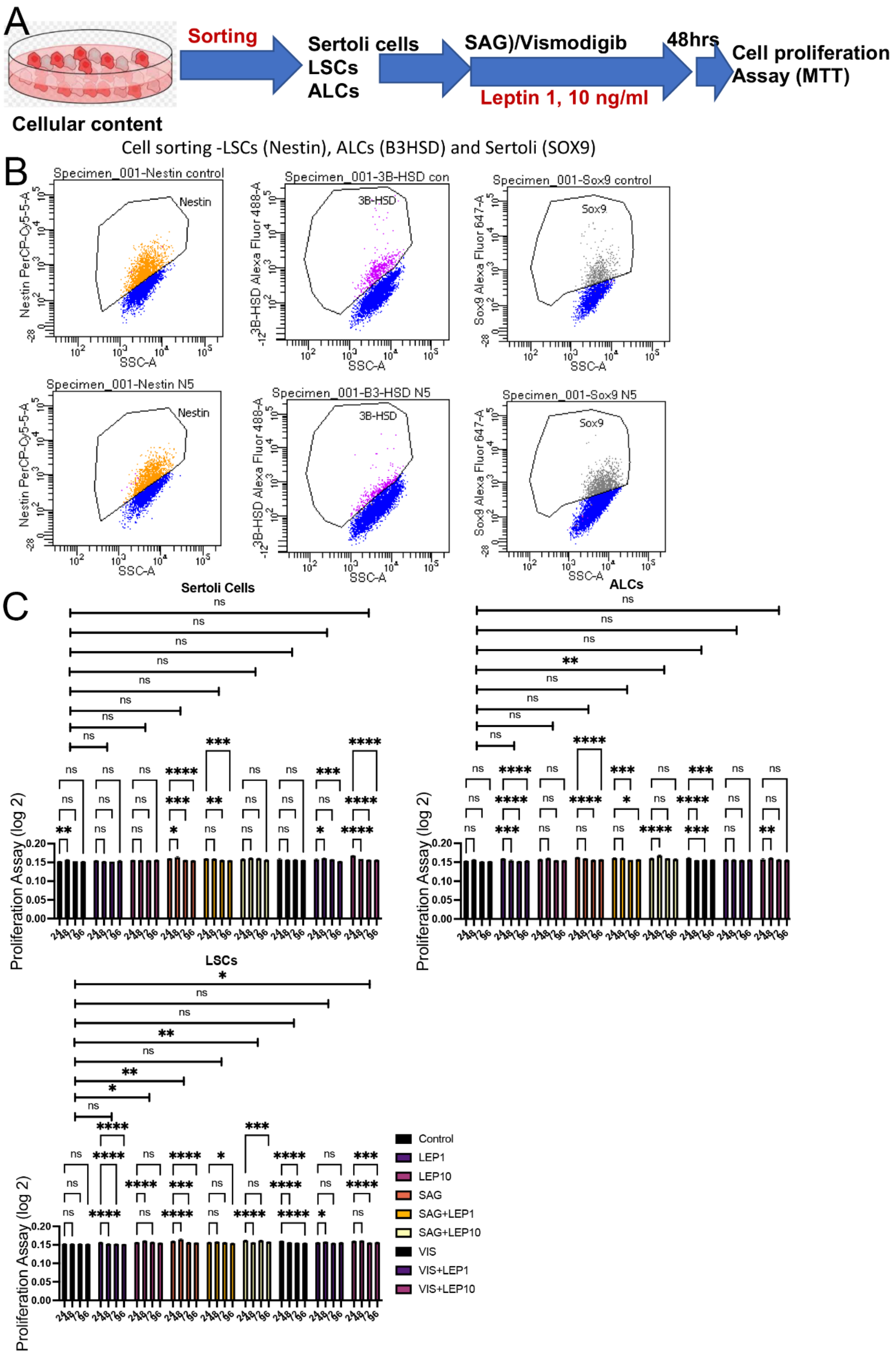
A
Sorting
Sertoli cells
LSCs
ALCs
SAG)/Vismodigib
Leptin 1, 10 ng/ml
48hrs
Cell proliferation
Assay (MTT)
Cellular content
Cell sorting -LSCs (Nestin), ALCs (B3HSD) and Sertoli (SOX9)
B
Specimen_001-Nestin control
Specimen_001-3B-HSD con
Specimen_001-Sox9 control
Specimen_001-Nestin N5
Specimen_001-B3-HSD N5
Specimen_001-Sox9 N5
Nestin
3B-HSD
Sox9
Nestin PerCP-Cy5-5-A
3B-HSD Alexa Fluor 488-A
Sox9 Alexa Fluor 647-A
SSC-A
C
Sertoli Cells
ALCs
LSCs
Proliferation Assay (log 2)
Control
LEP1
LEP10
SAG
SAG+LEP1
SAG+LEP10
VIS
VIS+LEP1
VIS+LEP10

Supplementary Figure 7. A) Overview of experimental design. B) Flow cytometry results showing the population of LSCs, ALCs, and Sertoli cells. C) MTT assay results post-treatment of LSCs, ALCs, and Sertoli cells with DHH agonist and antagonist, followed by Leptin treatment at 1 and 10ng/mL at 24, 48, 72, and 96 hours, respectively.

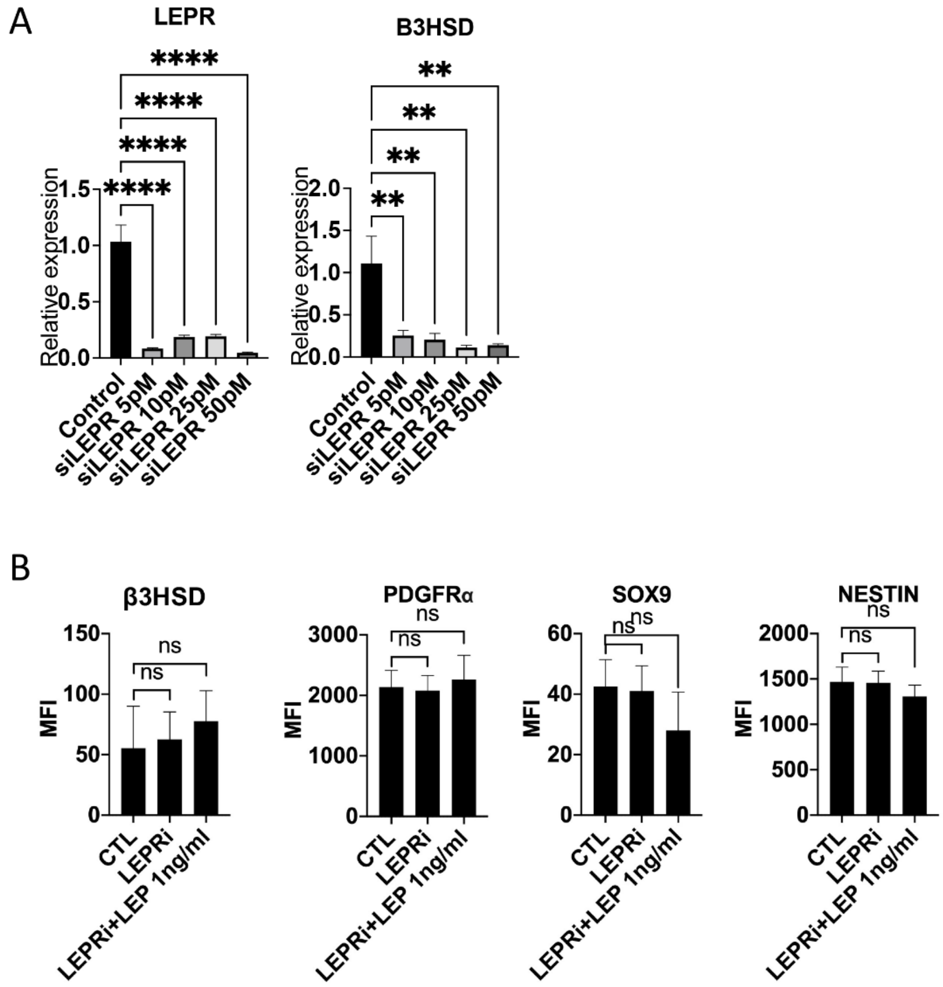

Supplementary Figure 8. A) Impact of different doses of siRNA against LEPR to reliably inhibit LEPR and B3HSD expression. B) Impact of leptin receptor inhibition on leptin-induced differentiation of LSCs. Leptin receptors were inhibited in LSCs using siRNA against LEPR, followed by treating the cells with 1ng/ml of leptin for 24, 48, 72, and 96 hours. Cumulative changes in the total number of cells staining positive for B3HSD, PDGFRa, SOX9, and NESTIN.

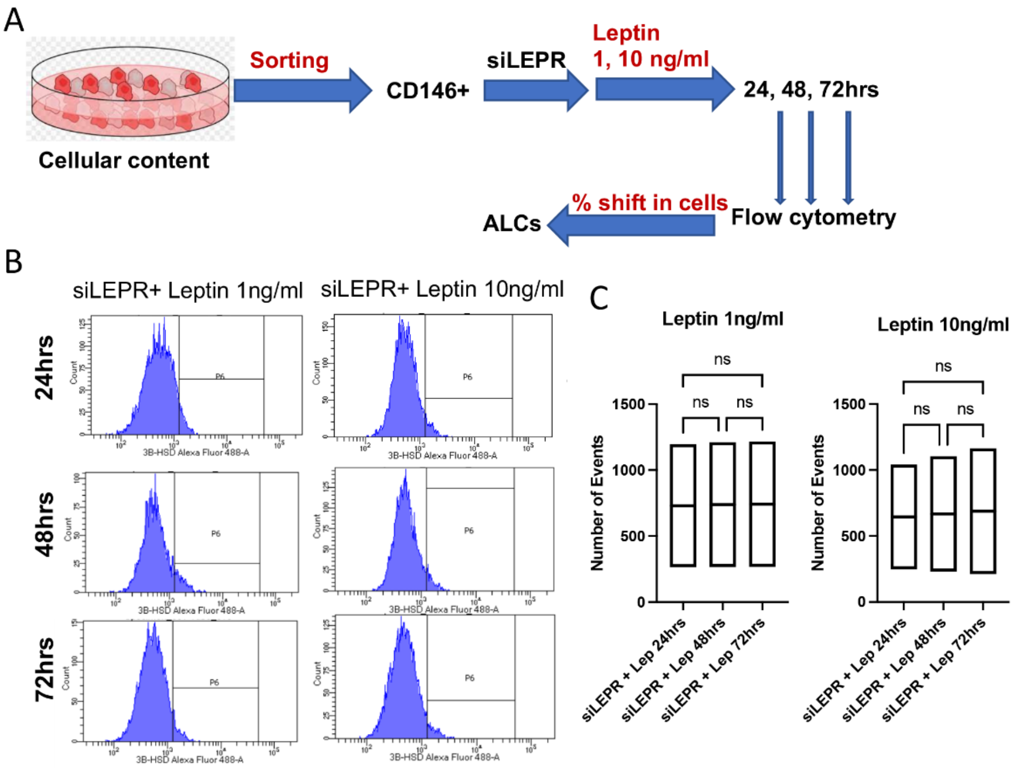

Supplementary Figure 9. A) Overview of experimental design. B) and C) Flow cytometry results showing the shift in population of ALCs upon exposing CD146+ cells with siLEPR followed by Leptin treatment at 1 or 10ng/ml for 24, 48 and 72, hours respectively. C) MTT assay results post treatment of LSCs, ALCs and Sertoli cells with DHH agonist and antagonist, followed by Leptin treatment at 1 and 10ng/mL at 24, 48, and 72 hours respectively.

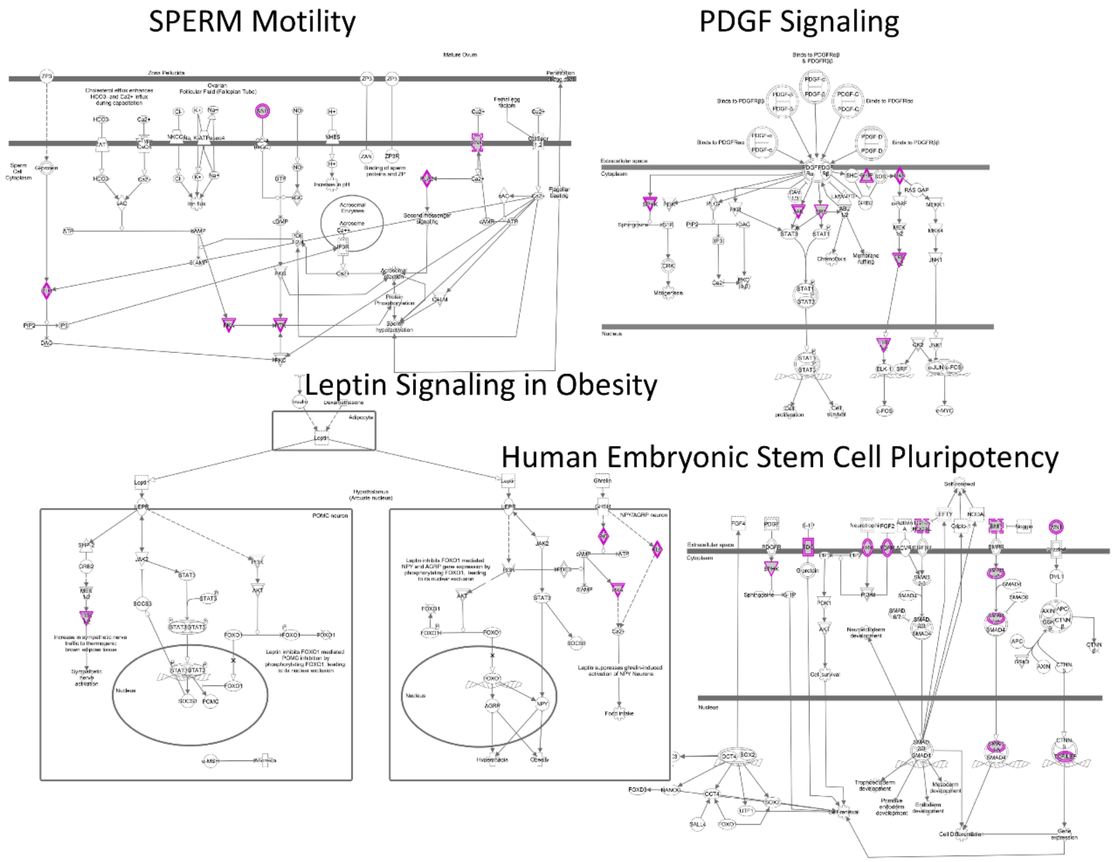

Supplementary Figure 10. Results of enrichment analysis showing different signaling pathways with which the markers that were significantly altered upon leptin treatment were involved.

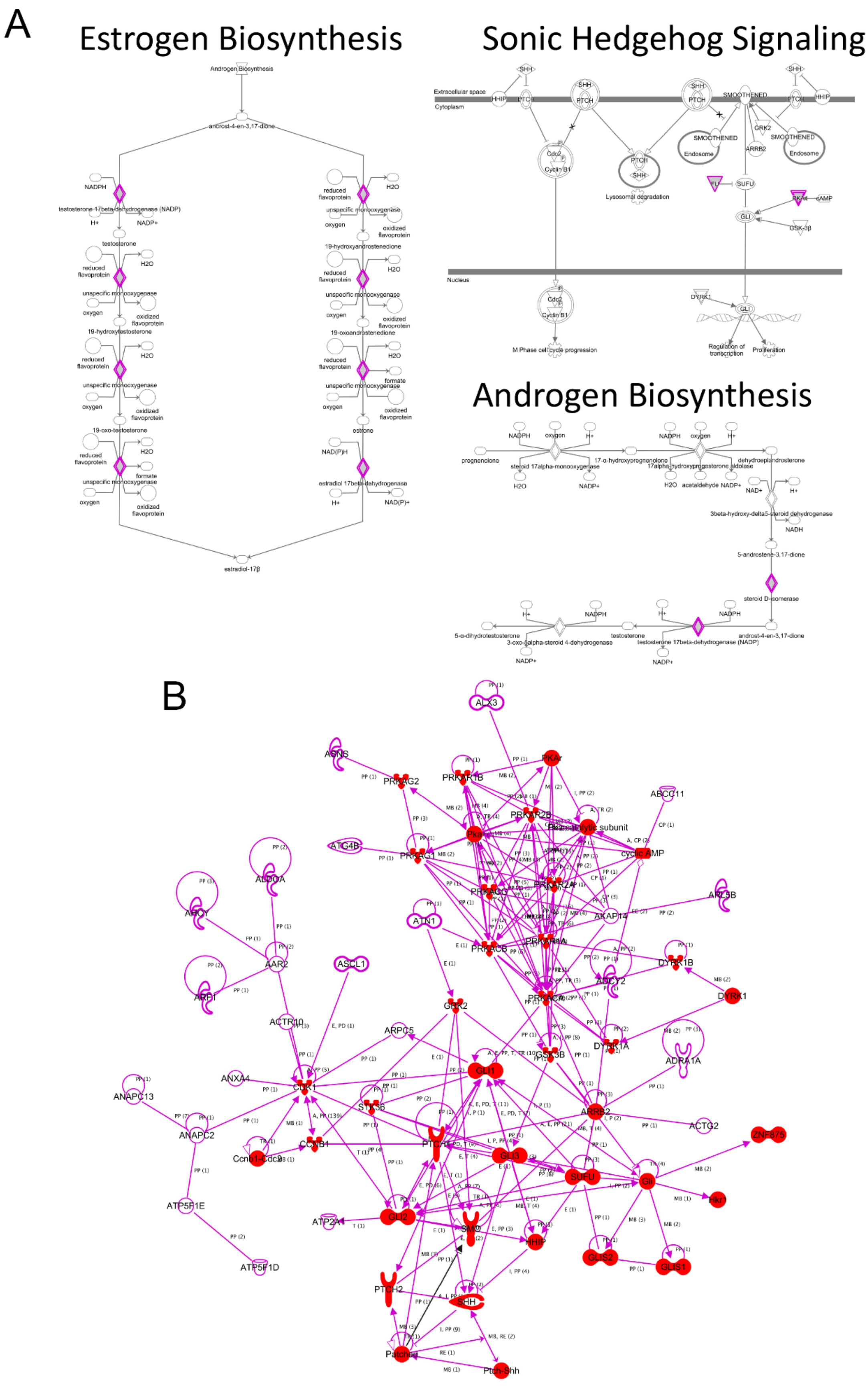
A
Estrogen Biosynthesis
Sonic Hedgehog Signaling
Androgen Biosynthesis
B
ALX3
PKAr
AGNS
PRKAG2
PRKAR1B
ABCC11
PRKAR2B
Pka-catalytic subunit
ATG4B
Pka
PRKAG1
cyclic AMP
ALDOA
AKAP14
ARL5B
ACCY
ATN1
PRKACB
AAR2
ASCL1
DYRK1B
ARPI
ADCY2
DYRK1
ACTR10
GRK2
ARPC5
GSK3B
DYRK1A
ADRA1A
ANXA4
CDK1
GLI1
ANAPC13
STK36
ARRB2
ACTG2
ZNF675
ANAPC2
CCNB1
PTCH1
GLI3
Ccnb1-Cdc2
SUFU
Gli
Hkr1
ATP5F1E
ATP2A1
GLI2
SMO
HHIP
GLIS2
GLIS1
ATP5F1D
PTCH2
SHH
Patched
Ptch-Shh

Supplementary Figure 11. A) Results of enrichment analysis showing different signaling pathways with which the markers that were significantly altered upon leptin treatment were involved. B) Results of enrichment analysis showing several important direct interactions between markers of DHH signaling and leptin-induced markers.

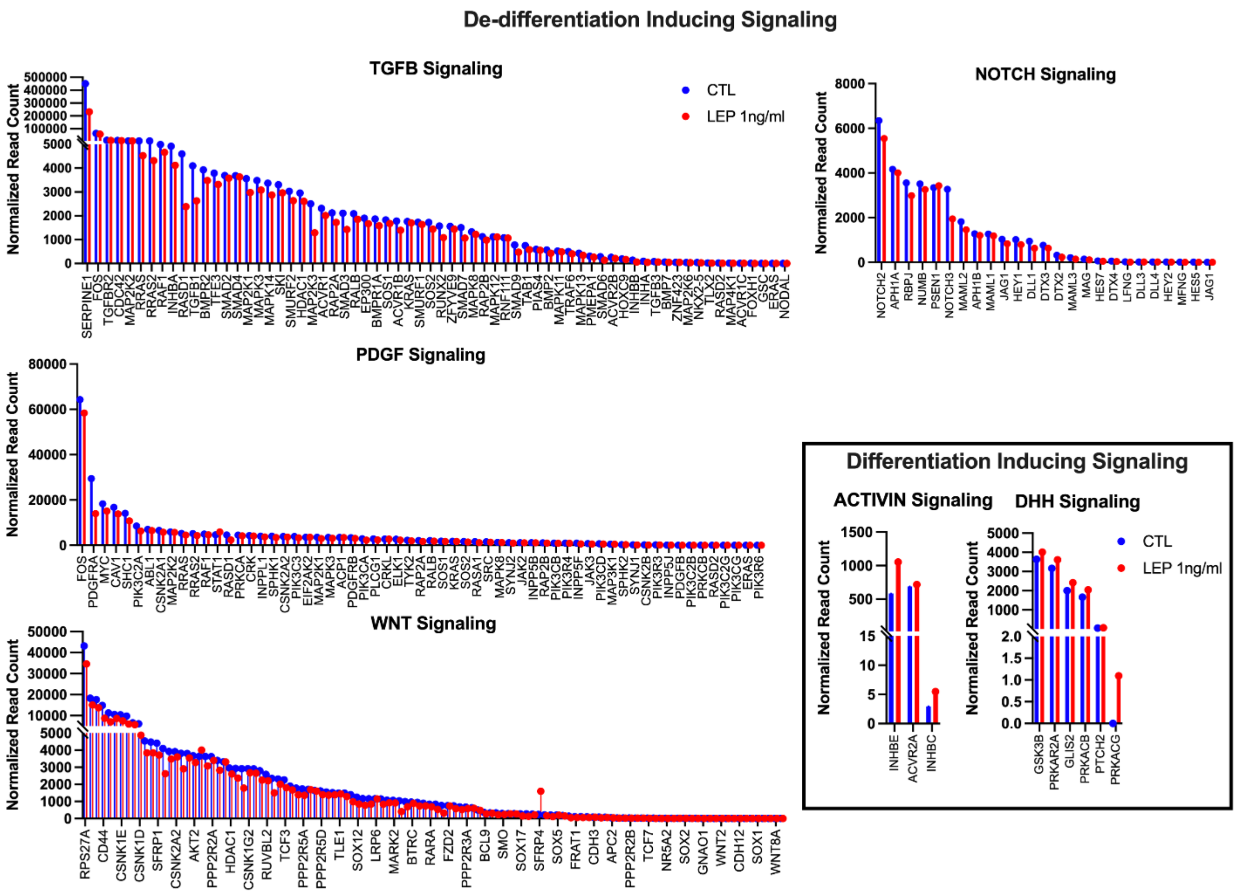

Supplementary Figure 12. Enrichment analysis showing the molecular events that are potentially modulated by Leptin during the differentiation of Leydig stem cells to adult Leydig cells.

RESOURCE TABLE

REAGENT or RESOURCE	SOURCE	IDENTIFIER
**Antibodies**		
Anti-GAPDH (14C10) Rabbit mAb	Cell Signaling Technology	Cat#2118; RRID: AB_561053
Anti-Sox2 (D6D9) XP Rabbit mAb	Cell Signaling Technology	Cat#3579S; RRID: AB_2195767
Anti-OCT-4 Rabbit polyclonal Ab	Cell Signaling Technology	Cat#2750; RRID: AB_823583
Anti-Leptin antibody	Abcam	Cat # ab87011
Anti-Ob-R/Leptin Receptor Ab	Abcam	Cat# ab16227
Anti-LHR Ab	Santa Cruz	Cat# sc-8391
Anti-DDX4 (VASA)	Abcam	Cat# ab13840
Anti-PLZF	BD Biosciences	Cat# 565738
Anti-Nestin	BD Biosciences	Cat# 561231
Anti-Vimentin	BD Biosciences	Cat# 562337
Anti-HSD17B3	Abcam	Cat# ab126228
Anti-SMHC	Abcam	Cat# ab133567
Anti-alpha SMA	Abcam	Cat# ab5694
Normal mouse IgG	Abcam	Cat# ab6785
Normal Rabbit IgG	Santa Cruz	Cat#SC-2025
Anti-β-Actin (AC-15) Mouse	Santa Cruz	Cat# SC-2027
Anti-Mouse IgG (H+L), HRP Conjugate	Sigma	Cat#A1978; RRID:AB_476692
Anti-Rabbit IgG (H+L), HRP Conjugate	Promega	Cat#W4021; RRID:AB_430834
**Biological Samples**	Promega	Cat#W4011; RRID:AB_430833
Testis Biopsies		
**Chemicals, Peptides, and Recombinant Proteins**	University of Miami	

Collagenase from Clostridium histolyticum Type IA		
TRIzol Reagent	Sigma-Aldrich	
iQ™ SYBR® Green Supermix	Invitrogen	Cat#C9891
Insulin, human recombinant, zinc solution	Bio-Rad	Cat#10296-028
B27 Supplement (50X), serum free	Thermo Fisher Scientific	Cat#170-8886
Recombinant Human EGF Protein, CF	Thermo Fisher Scientific	Cat#12585014
FGF-Basic (AA 10-155) Recombinant Human	R&D Systems	Cat#17504044
RIPA Buffer	Thermo Fisher Scientific	Cat#236-EG
PhosphataseArrest™ Phosphatase Inhibitor Cocktail	Cell Signaling	Cat# PHG0026
ProteaseArrest™ Protease Inhibitor Cocktail	G-Biosciences	Cat#9806
Pierce™ ECL Western Blotting Substrate	G-Biosciences	Cat#786-450
**Critical Commercial Assays**	Thermo Scientific	Cat#786-331
iScript cDNA Synthesis Kit	Thermo Scientific	Cat#32106
Truseq standard Total RNA Library Prep	Bio-Rad	
RayBio Cytokine antibody Array Kit	RayBio	Cat#1708891
NEBNext® Ultra™ II RNA Library Prep Kit for Illumina	Illumina,San Diego	Cat # E7765S

**Oligonucleotides**		
QPCR PRIMER		
Primer PCR: GAPDH Forward: 5´-ATCAAGTGGGGCGATGCTG-3’	This paper	N/A
Primer PCR: GAPDH Reverse:5´-ACCCATGACGAACATGGGG-3’	This paper	N/A
Primer PCR SOX2 Forward: 5′-CGAGTAGGACATGCTGTAGGT-3´	This Paper	N/A
Primer PCR SOX2 Reverse: 5′-TGGACAGTTACGCGCACAT-3	This Paper	N/A
Primer PCR NANOG Forward: 5´-GCTTTGAAGCATCCGACTG-3´	This Paper	N/A
Primer PCR NANOG Reverse: 5´-GATAGTTTTCTTCAGGCCCACA-3	This Paper	N/A
Primer PCR OCT4 Forward :5’-GAGAAGGATGTGGTCCGAG- 3’	This Paper	N/A

Primer PCR OCT4 Reverse: 5’-TCCTCTCGTTGTGCATAGTC -3’	This Paper	N/A
Primer PCR CD90 Forward: 5′- ATGAACCTGGCCATCAGCA -3´	This Paper	N/A
Primer PCR CD90 Reverse: 5′- GTGTGCTCAGGCACCCC -3´	This Paper	N/A
Primer PCR CD51 Forward: 5′- ATCTGTGAGGTCGAAACAGGA -3´	This Paper	N/A
Primer PCR CD51 Reverse: 5′- TGGAGCATACTCAACAGTCTTTG -3´	This Paper	N/A
Primer PCR COUP-TFII Forward: 5′- AAGCCATCGTGCTGTTCAC -3´	This Paper	N/A
Primer PCR COUP-TFII Reverse: 5′- GCTCCTCACGTACTCCTCCA -3´	This Paper	N/A
Primer PCR LEPR Forward: 5′- ACCTCTGGTTCCCCAAAAAGG -3´	This Paper	N/A
Primer PCR LEPR Reverse: 5′- TTGGCACAGGCACAAGACAT -3´	This Paper	N/A
Primer PCR LEPTIN Forward: 5′- TGCCTTCCAGAAACGTGATCC -3´	This Paper	N/A
Primer PCR LEPTIN Reverse: 5′- CTCTGTGGAGTAGCCTGAAGC -3´	This Paper	N/A
Primer PCR Nestin Forward: 5′- CTGCTACCCTTGAGACACCTG -3´	This Paper	N/A
Primer PCR Nestin Reverse: 5′- GGGCTCTGATCTCTGCATCTAC -3´	This Paper	N/A
Primer PCR PDGFRA Forward: 5′- GCAAGACCAGGACGGTCATTT -3´	This Paper	N/A
Primer PCR PDGFRA Reverse: 5′- GGCACTTGACACTGCTCGT -3´	This Paper	N/A
Primer PCR B3HSD Forward: 5′- CACATGGCCCGCTCCATAC -3´	This Paper	N/A
Primer PCR B3HSD Reverse: 5′- GTGCCGCCGTTTTTCAGATTC -3´	This Paper	N/A
Primer PCR GLI Forward: 5′- AGTGGATGTACCCAACAGGTG -3´	This Paper	N/A
Primer PCR GLI Reverse: 5′- TACCAGCAGTCTCAGTTCTCC -3´	This Paper	N/A
Primer PCR SMO Forward: 5′- GAACATGCTCTTGGAGTGGTG -3´	This Paper	N/A
Primer PCR SMO Reverse: 5′- CTGCGTAGTCAAAGGCGTCA -3´	This Paper	N/A

**Software and Algorithms**		
Adobe Illustrator	Adobe Systems, San Jose, CA	https://www.adobe.com/ca/products/illustrator.html
FlowJo software V10	FlowJo, LLC	https://www.flowjo.com/

Data and Code Availability

RNA-seq data is deposited in Gene Expression Omnibus (GEO) repository.

EXPERIMENTAL MODEL AND SUBJECT DETAILS

Experiments were carried out in compliance with the Institutional Review Boards (IRBs) of the University of Miami. Molecular analyses were performed using standard procedures. A more detailed description and additional data are provided in Materials and Methods.

Supplementary Table 1. Showing hierarchical clustering showing protein coding candidates that were selected from RNA sequencing data and subjected to further enrichment analysis.

Symbol	Location	0.y	0.25.y	1.y	10.y	min	max	0.25	1.00	10.00
KCNA7	19:49570675-49576198	0.99	0.00	0.00	2.15	0.23	1.76	D	D	U
ZFR2	19:3804022-3869030	0.99	0.00	0.00	2.15	0.23	1.76	D	D	U
SLC6A12	12:299243-323736	0.99	0.00	0.00	2.15	0.23	1.76	D	D	U
CNTN6	3:1134260-1445901	0.99	0.00	0.00	2.15	0.23	1.76	D	D	U
CLDN14	21:37832919-37948867	0.99	0.00	0.00	2.15	0.23	1.76	D	D	U
SERPINA9	14:94929054-94946026	0.99	0.00	0.00	2.15	0.23	1.76	D	D	U
PCP2	19:7696497-7698634	0.99	0.00	0.00	2.15	0.23	1.76	D	D	U
HORMAD2	22:30476163-30573064	0.99	0.00	0.00	2.15	0.23	1.76	D	D	U
GPR97	16:57702099-57723975	0.99	0.00	0.00	2.15	0.23	1.76	D	D	U
SDR42E2	16:22177618-22203075	0.99	0.00	0.00	2.15	0.23	1.76	D	D	U
AKAP14	X:119029800-119054679	0.99	0.00	0.00	2.15	0.23	1.76	D	D	U
ERAS	X:48687283-48688548	0.99	0.00	0.00	2.15	0.23	1.76	D	D	U
OR7A5	19:14903302-14946188	0.99	0.00	0.00	2.15	0.23	1.76	D	D	U
NCCRP1	19:39687601-39692524	0.99	0.00	0.00	2.15	0.23	1.76	D	D	U
FAM47C	X:37026432-37029739	0.99	0.00	0.00	2.15	0.23	1.76	D	D	U
C1orf141	1:67557848-67697536	0.99	0.00	0.00	2.15	0.23	1.76	D	D	U
AC011997.1	2:198557830-198639547	0.99	0.00	0.00	2.15	0.23	1.76	D	D	U
KRTAP5-7	11:71238313-71239210	0.99	0.00	0.00	2.15	0.23	1.76	D	D	U
CCDC166	8:144788864-144790279	0.99	0.00	0.00	2.15	0.23	1.76	D	D	U
CBLN1	16:49311828-49315742	1.98	0.00	0.00	4.30	0.45	3.52	D	D	U
VIPR2	7:158820866-158937649	1.98	0.00	0.00	3.22	0.79	3.17	D	D	U
XG	X:2670091-2734539	1.98	0.00	0.00	3.22	0.79	3.17	D	D	U
ELL3	15:44064798-44069741	1.98	1.01	1.09	3.22	1.21	2.76	D	D	U
LRRC63	13:46786083-46851501	1.98	1.01	1.09	3.22	1.21	2.76	D	D	U
RRH	4:110749150-110765760	1.98	1.01	1.09	3.22	1.21	2.76	D	D	U
TLR9	3:52255096-52273183	1.98	0.00	0.00	3.22	0.79	3.17	D	D	U
ZSCAN1	19:58545400-58565999	2.98	2.02	2.19	4.30	2.20	3.76	D	D	U
PCDHAC1	5:140306302-140391929	2.98	1.01	1.09	5.37	1.44	4.51	D	D	U
EPHA5	4:66185281-66536213	3.97	2.02	1.09	6.45	2.19	5.74	D	D	U
CTD-2116N17.1	15:64472547-64665980	3.97	0.00	0.00	6.45	1.59	6.35	D	D	U
TESC	12:117476728-117537284	4.96	2.02	3.28	6.45	3.51	6.41	D	D	U
IL17C	16:88704999-88706881	4.96	2.02	3.28	6.45	3.51	6.41	D	D	U
ERP27	12:15066969-15092016	4.96	3.02	3.28	7.52	3.41	6.51	D	D	U
MYBPC2	19:50936160-50969578	5.95	3.02	3.28	8.59	3.99	7.91	D	D	U
C10orf95	10:104209594-104211300	6.95	2.02	2.19	10.74	3.80	10.09	D	D	U
HIST1H1E	6:26156559-26157343	8.93	5.04	4.38	11.82	6.31	11.54	D	D	U
DNM3	1:171810621-172387606	9.92	7.06	7.66	12.89	7.94	11.90	D	D	U
ABHD16A	6:31654726-31671221	9.92	9.07	8.76	10.74	9.25	10.59	D	D	U
SULT1A2	16:28603264-28608430	11.91	10.08	9.85	15.04	10.11	13.70	D	D	U
SRCRB4D	7:76018651-76039012	12.90	12.09	12.04	13.96	12.22	13.57	D	D	U
UPK2	11:118795873-118829269	13.89	9.07	6.57	18.26	10.00	17.78	D	D	U
ACTG2	2:74119441-74146992	13.89	7.06	9.85	19.34	9.89	17.89	D	D	U
AC018816.3	3:4855978-4928977	15.87	12.09	9.85	19.34	12.74	19.01	D	D	U
ACTL10	20:32254304-32256331	16.87	9.07	10.95	21.48	12.61	21.12	D	D	U
KIF26A	14:104605060-104647231	18.85	17.13	17.52	20.41	17.74	19.96	D	D	U
C21orf67	21:46352729-46359828	19.84	15.12	14.23	23.63	16.56	23.12	D	D	U
GSTT2B	22:24299601-24303373	26.79	14.11	19.71	33.30	20.52	33.05	D	D	U
C11orf91	11:33719807-33722347	32.74	21.17	18.61	41.89	24.65	40.83	D	D	U
NKPD1	19:45653008-45663408	37.70	28.22	26.28	44.04	31.46	43.94	D	D	U
C1orf134	1:16555368-16556038	38.69	27.21	31.75	48.34	31.80	45.59	D	D	U
HYAL1	3:50337320-50349812	58.54	49.39	45.99	68.75	50.88	66.19	D	D	U
ANKLE1	19:17392454-17398455	66.47	57.45	56.94	79.49	58.56	74.39	D	D	U
ZNF135	19:58570607-58597677	67.47	60.47	60.22	79.49	60.69	74.24	D	D	U
KCND1	X:48818639-48827976	69.45	65.51	65.70	73.05	66.77	72.13	D	D	U
HERC6	4:89299891-89364263	75.40	71.56	72.27	79.49	72.69	78.12	D	D	U

ADAM8	10:135075907-135090372	107.15	94.74	91.98	121.39	97.07	117.24	D	D	U
GET4	7:916189-936073	117.07	101.80	97.45	135.35	104.20	129.94	D	D	U
DHODH	16:72042487-72058954	181.56	169.33	167.53	193.36	172.55	190.58	D	D	U
METTL1	12:58162254-58166576	227.20	212.67	210.24	252.44	212.67	241.74	D	D	U
TCTA	3:49449639-49453908	239.11	205.61	221.19	259.96	221.55	256.67	D	D	U
KCNIP3	2:95963052-96051825	253.99	231.82	238.71	273.93	239.99	268.00	D	D	U
FAM211B	22:24981588-24989175	260.94	213.68	231.04	292.19	235.04	286.84	D	D	U
HSD11B1L	19:5680615-5688533	297.65	263.06	271.56	337.31	272.63	322.67	D	D	U
SPHK2	19:49122548-49133974	323.44	309.43	302.22	339.45	311.11	335.77	D	D	U
FBXW9	19:12798867-12807457	351.22	331.60	328.49	372.76	335.87	366.58	D	D	U
MYPOP	19:46393278-46405862	395.87	349.74	331.78	429.69	362.55	429.19	D	D	U
SCO2	22:50961997-50964868	526.84	481.78	471.94	574.71	491.50	562.17	D	D	U
NRF1	7:129251555-129396922	548.66	521.09	517.93	597.27	521.13	576.20	D	D	U
APOPT1	14:104029299-104073860	548.66	522.09	514.64	568.26	530.15	567.17	D	D	U
C5orf45	5:179261436-179289173	572.48	527.13	537.64	604.79	545.99	598.96	D	D	U
NDUFA3	19:54606036-54612564	580.41	529.15	519.02	624.12	543.88	616.95	D	D	U
HOOK2	19:12873817-12983554	792.73	670.26	654.80	871.20	715.39	870.08	D	D	U
CISD3	17:36886488-36891297	800.67	726.70	740.21	865.82	752.90	848.45	D	D	U
DDT	22:24313554-24322660	824.48	799.27	800.43	866.90	800.76	848.21	D	D	U
FAM214B	9:35104109-35116338	830.44	782.13	757.73	879.79	789.88	870.99	D	D	U
DDX51	12:132621139-132628880	910.80	860.75	858.47	950.69	877.66	943.94	D	D	U
THAP11	16:67876213-67878097	952.47	889.98	878.18	1004.40	908.48	996.46	D	D	U
ARL16	17:79648204-79650954	1025.89	994.80	999.72	1059.18	1003.74	1048.04	D	D	U
C17orf70	17:79506911-79520987	1105.26	1015.97	1052.28	1155.86	1059.36	1151.17	D	D	U
SF3B4	1:149895209-149900236	1140.98	1111.72	1108.12	1190.24	1112.52	1169.45	D	D	U
NAGLU	17:40688190-40696467	1150.90	1022.01	1027.09	1239.65	1072.13	1229.68	D	D	U
LLGL1	17:18128901-18148189	1222.34	1156.07	1103.74	1289.07	1161.99	1282.68	D	D	U
MRPL33	2:27994584-28210954	1257.06	1159.09	1174.92	1328.81	1198.07	1316.05	D	D	U
MCRS1	12:49950327-49961936	1261.03	1182.27	1180.39	1350.30	1200.65	1321.41	D	D	U
SIDT2	11:117049449-117068160	1328.50	1241.74	1168.35	1409.38	1250.05	1406.95	D	D	U
ESRRA	11:64073044-64084215	1406.88	1325.39	1326.02	1527.55	1335.33	1478.43	D	D	U
H2AFX	11:118964564-118966177	1432.68	1319.35	1305.22	1569.44	1340.73	1524.62	D	D	U
TLE1	9:84198598-84304220	1489.23	1445.33	1445.38	1536.14	1456.73	1521.73	D	D	U
COPE	19:19010323-19030206	1603.33	1421.14	1484.80	1729.50	1501.28	1705.38	D	D	U
BAD	11:64037302-64052176	1842.44	1722.51	1718.03	1923.93	1767.60	1917.28	D	D	U
TPRA1	3:127291912-127317094	2050.79	1985.57	1968.78	2140.93	1992.23	2109.35	D	D	U
FAH	15:80444832-80479288	2172.83	2097.45	2052.00	2258.02	2105.11	2240.55	D	D	U
AURKAIP1	1:1309110-1310875	2450.63	2276.85	2246.90	2564.17	2338.40	2562.86	D	D	U
CORO1B	11:67205519-67211292	2519.09	2452.23	2459.33	2624.33	2459.40	2578.78	D	D	U
IMPDH1	7:128032331-128050306	2702.64	2603.42	2505.32	2806.95	2605.41	2799.87	D	D	U
SREBF1	17:17713713-17740325	3329.68	3125.51	3036.39	3564.27	3153.54	3505.82	D	D	U
APH1A	1:150237804-150241980	4165.08	4037.66	4006.54	4314.08	4060.01	4270.15	D	D	U
GSTP1	11:67351066-67354131	7409.44	7010.98	6979.42	8054.53	7034.08	7784.79	D	D	U
CD151	11:832843-839831	10247.01	9717.20	9559.20	10700.34	9856.51	10637.50	D	D	U
RPL36	19:5674958-5691887	14211.67	11850.93	12696.33	15462.38	13009.29	15414.05	D	D	U
UTS2	1:7903143-7913572	0.99	0.00	0.00	1.07	0.54	1.44	D	D	N
STON1-GTF2A1L	2:48757064-49003654	0.99	0.00	0.00	1.07	0.54	1.44	D	D	N
NOX3	6:155716504-155777037	0.99	0.00	0.00	1.07	0.54	1.44	D	D	N
CAPN6	X:110488331-110513751	0.99	0.00	0.00	1.07	0.54	1.44	D	D	N
SEZ6L	22:26565440-26779562	0.99	0.00	0.00	1.07	0.54	1.44	D	D	N
NTSR1	20:61340189-61394123	0.99	0.00	0.00	1.07	0.54	1.44	D	D	N
PCDH11X	X:91034260-91878229	0.99	0.00	0.00	1.07	0.54	1.44	D	D	N
TAS2R8	12:10958650-10959892	0.99	0.00	0.00	1.07	0.54	1.44	D	D	N
S1PR4	19:3172344-3180329	0.99	0.00	0.00	1.07	0.54	1.44	D	D	N
TSPAN16	19:11406824-11437672	0.99	0.00	0.00	1.07	0.54	1.44	D	D	N
G6PC	17:41052814-41065386	0.99	0.00	0.00	1.07	0.54	1.44	D	D	N

LRRC9	14:60386431-60530277	0.99	0.00	0.00	1.07	0.54	1.44	D	D	N
CA1	8:86239837-86291243	0.99	0.00	0.00	1.07	0.54	1.44	D	D	N
TTC29	4:147627790-147867034	0.99	0.00	0.00	1.07	0.54	1.44	D	D	N
LBX1	10:102986733-102989551	0.99	0.00	0.00	1.07	0.54	1.44	D	D	N
LMAN1L	15:75105057-75118099	0.99	0.00	0.00	1.07	0.54	1.44	D	D	N
ABCG8	2:44066103-44105605	0.99	0.00	0.00	1.07	0.54	1.44	D	D	N
SYT8	11:1848709-1858751	0.99	0.00	0.00	1.07	0.54	1.44	D	D	N
ASZ1	7:117003276-117068177	0.99	0.00	0.00	1.07	0.54	1.44	D	D	N
ERG	21:39751949-40033704	0.99	0.00	0.00	1.07	0.54	1.44	D	D	N
ASGR2	17:7004641-7019019	0.99	0.00	0.00	1.07	0.54	1.44	D	D	N
FAM151A	1:55074855-55089229	0.99	0.00	0.00	1.07	0.54	1.44	D	D	N
OXER1	2:42989642-42991401	0.99	0.00	0.00	1.07	0.54	1.44	D	D	N
FCAMR	1:207131310-207143970	0.99	0.00	0.00	1.07	0.54	1.44	D	D	N
TCF23	2:27371872-27376378	0.99	0.00	0.00	1.07	0.54	1.44	D	D	N
GPR111	6:47624172-47665533	0.99	0.00	0.00	1.07	0.54	1.44	D	D	N
MC4R	18:58038564-58040001	0.99	0.00	0.00	1.07	0.54	1.44	D	D	N
XAGE3	X:52891557-52897099	0.99	0.00	0.00	1.07	0.54	1.44	D	D	N
CCDC63	12:111284573-111345339	0.99	0.00	0.00	1.07	0.54	1.44	D	D	N
CYSLTR1	X:77526961-77583048	0.99	0.00	0.00	1.07	0.54	1.44	D	D	N
GLIPR1L1	12:75728419-75764340	0.99	0.00	0.00	1.07	0.54	1.44	D	D	N
FMR1NB	X:147062849-147108187	0.99	0.00	0.00	1.07	0.54	1.44	D	D	N
ZNF366	5:71738479-71803554	0.99	0.00	0.00	1.07	0.54	1.44	D	D	N
KLHDC7A	1:18807424-18812478	0.99	0.00	0.00	1.07	0.54	1.44	D	D	N
HCAR2	12:123185840-123187890	0.99	0.00	0.00	1.07	0.54	1.44	D	D	N
OLIG1	21:34442450-34444726	0.99	0.00	0.00	1.07	0.54	1.44	D	D	N
C9orf171	9:135285430-135448704	0.99	0.00	0.00	1.07	0.54	1.44	D	D	N
FAM150A	8:53446597-53478067	0.99	0.00	0.00	1.07	0.54	1.44	D	D	N
C17orf102	17:32901142-32906388	0.99	0.00	0.00	1.07	0.54	1.44	D	D	N
SPN	16:29674300-29682187	0.99	0.00	0.00	1.07	0.54	1.44	D	D	N
RD3	1:211649864-211666259	0.99	0.00	0.00	1.07	0.54	1.44	D	D	N
CD3E	11:118175260-118186890	0.99	0.00	0.00	1.07	0.54	1.44	D	D	N
METTL11B	1:170115142-170136931	0.99	0.00	0.00	1.07	0.54	1.44	D	D	N
FCGR3A	1:161511549-161600917	0.99	0.00	0.00	1.07	0.54	1.44	D	D	N
CLPSL1	6:35748794-35761452	0.99	0.00	0.00	1.07	0.54	1.44	D	D	N
IGFL4	19:46543006-46580376	0.99	0.00	0.00	1.07	0.54	1.44	D	D	N
SERPINB4	18:61304493-61311532	0.99	0.00	0.00	1.07	0.54	1.44	D	D	N
LL22NC03-63E9.3	22:22901750-22909007	0.99	0.00	0.00	1.07	0.54	1.44	D	D	N
PRR5-ARHGAP8	22:45098113-45258586	0.99	0.00	0.00	1.07	0.54	1.44	D	D	N
RP11-487E13.1	4:178163693-178169927	0.99	0.00	0.00	1.07	0.54	1.44	D	D	N
PCDHA12	5:140255058-140391929	0.99	0.00	0.00	1.07	0.54	1.44	D	D	N
RP11-796G6.2	14:102196774-102198859	0.99	0.00	0.00	1.07	0.54	1.44	D	D	N
AC006116.20	19:56784138-56821819	0.99	0.00	0.00	1.07	0.54	1.44	D	D	N
TNFRSF9	1:7979907-8000926	1.98	1.01	0.00	2.15	1.24	2.73	D	D	N
RDH8	19:10123925-10132955	1.98	1.01	0.00	2.15	1.24	2.73	D	D	N
ZDHHC15	X:74588262-74743337	1.98	0.00	0.00	2.15	1.09	2.88	D	D	N
DLX5	7:96649704-96654409	1.98	0.00	1.09	2.15	1.24	2.72	D	D	N
ACADL	2:211052663-211090215	1.98	0.00	1.09	2.15	1.24	2.72	D	D	N
WNT6	2:219724544-219738955	1.98	0.00	0.00	2.15	1.09	2.88	D	D	N
ATP10B	5:159990127-160279221	1.98	0.00	1.09	2.15	1.24	2.72	D	D	N
CRHR1	17:43699267-43913194	1.98	1.01	0.00	2.15	1.24	2.73	D	D	N
NPPB	1:11917521-11918988	1.98	0.00	1.09	2.15	1.24	2.72	D	D	N
REG4	1:120336641-120354283	1.98	1.01	1.09	2.15	1.54	2.43	D	D	N
SLCO1B1	12:21284136-21392180	1.98	0.00	1.09	2.15	1.24	2.72	D	D	N
FAM217A	6:4049668-4087578	1.98	1.01	1.09	2.15	1.54	2.43	D	D	N
TTBK1	6:43211418-43255997	1.98	1.01	0.00	2.15	1.24	2.73	D	D	N

GDF6	8:97154562-97173020	1.98	1.01	1.09	2.15	1.54	2.43	D	D	N
DPPA2	3:109012635-109035364	1.98	0.00	0.00	2.15	1.09	2.88	D	D	N
COL22A1	8:139600478-139926249	1.98	0.00	1.09	2.15	1.24	2.72	D	D	N
TTLL6	17:46839597-46894576	1.98	1.01	1.09	2.15	1.54	2.43	D	D	N
GIMAP8	7:150147718-150176480	1.98	0.00	1.09	2.15	1.24	2.72	D	D	N
DAND5	19:13075973-13085567	1.98	0.00	1.09	2.15	1.24	2.72	D	D	N
TEX38	1:47134527-47139266	1.98	0.00	1.09	2.15	1.24	2.72	D	D	N
PAQR9	3:142668006-142682178	1.98	0.00	0.00	2.15	1.09	2.88	D	D	N
HIST1H4D	6:26188938-26189304	1.98	1.01	1.09	2.15	1.54	2.43	D	D	N
MYL4	17:45277812-45301045	1.98	1.01	1.09	2.15	1.54	2.43	D	D	N
HIST1H2AL	6:27833034-27833606	1.98	0.00	1.09	2.15	1.24	2.72	D	D	N
C2CD4B	15:62455734-62457482	1.98	1.01	0.00	2.15	1.24	2.73	D	D	N
AC104472.1	3:155459933-155461515	1.98	0.00	0.00	2.15	1.09	2.88	D	D	N
AF131215.5	8:10983980-10987745	1.98	0.00	1.09	2.15	1.24	2.72	D	D	N
AKR1B15	7:134233888-134264627	1.98	1.01	1.09	2.15	1.54	2.43	D	D	N
OMP	11:76813886-76814377	1.98	0.00	0.00	2.15	1.09	2.88	D	D	N
RP11-87C12.2	12:122326715-122359376	1.98	0.00	1.09	2.15	1.24	2.72	D	D	N
AP001055.1	21:45587818-45593580	1.98	0.00	0.00	2.15	1.09	2.88	D	D	N
NRIP2	12:2934514-2944710	2.98	1.01	0.00	2.15	2.00	3.95	D	D	N
AQP6	12:50360977-50370922	2.98	0.00	0.00	2.15	1.84	4.11	D	D	N
UPK3A	22:45680863-45691755	2.98	0.00	1.09	2.15	2.01	3.94	D	D	N
CHRNA4	20:61975420-62009753	2.98	0.00	0.00	2.15	1.84	4.11	D	D	N
GPRC5D	12:13093709-13105081	2.98	1.01	2.19	3.22	2.23	3.72	D	D	N
CD70	19:6583194-6604114	2.98	0.00	0.00	3.22	1.63	4.32	D	D	N
PLA2G5	1:20354672-20417683	2.98	2.02	1.09	3.22	2.25	3.71	D	D	N
TRPM1	15:31293264-31453476	2.98	1.01	0.00	2.15	2.00	3.95	D	D	N
ADAMTS14	10:72432559-72522197	2.98	2.02	0.00	2.15	2.03	3.92	D	D	N
IGSF6	16:21652609-21663981	2.98	1.01	0.00	2.15	2.00	3.95	D	D	N
ALX3	1:110602616-110613322	2.98	0.00	0.00	2.15	1.84	4.11	D	D	N
RBFOX3	17:77085427-77613550	2.98	0.00	1.09	3.22	1.82	4.13	D	D	N
CYP4F11	19:16023177-16045677	2.98	2.02	0.00	2.15	2.03	3.92	D	D	N
ANKS4B	16:21244986-21263750	2.98	0.00	0.00	2.15	1.84	4.11	D	D	N
LCN12	9:139844003-139849949	2.98	1.01	1.09	3.22	2.09	3.87	D	D	N
CYP4X1	1:47427036-47516423	2.98	0.00	0.00	2.15	1.84	4.11	D	D	N
C4orf22	4:81256874-81884910	2.98	2.02	2.19	3.22	2.54	3.42	D	D	N
KEL	7:142638201-142659768	2.98	2.02	0.00	2.15	2.03	3.92	D	D	N
MTRNR2L10	X:55207824-55208944	2.98	0.00	1.09	2.15	2.01	3.94	D	D	N
AC110771.1	4:187111913-187112626	2.98	1.01	2.19	3.22	2.23	3.72	D	D	N
STMN4	8:27092840-27115937	3.97	2.02	0.00	4.30	2.48	5.46	D	D	N
CNGA4	11:6255995-6265659	3.97	1.01	1.09	4.30	2.63	5.31	D	D	N
NTRK3	15:88418230-88799999	3.97	0.00	1.09	3.22	2.59	5.35	D	D	N
CCDC83	11:85566144-85631064	3.97	0.00	2.19	3.22	2.67	5.26	D	D	N
GRAP	17:18923986-18950950	3.97	2.02	0.00	4.30	2.48	5.46	D	D	N
NLRP14	11:7041677-7092539	3.97	0.00	1.09	3.22	2.59	5.35	D	D	N
KLHL10	17:39991937-40004636	3.97	3.02	1.09	3.22	3.05	4.89	D	D	N
KLKB1	4:187130133-187179625	3.97	3.02	2.19	4.30	3.25	4.68	D	D	N
C12orf50	12:88373816-88427814	3.97	1.01	2.19	4.30	2.81	5.13	D	D	N
HMX2	10:124907638-124910188	3.97	0.00	1.09	3.22	2.59	5.35	D	D	N
CACNA1E	1:181382238-181777219	3.97	1.01	0.00	4.30	2.36	5.57	D	D	N
AC104809.3	2:241858202-241932728	3.97	3.02	1.09	3.22	3.05	4.89	D	D	N
PCDHB17	5:140535577-140538639	3.97	3.02	1.09	3.22	3.05	4.89	D	D	N
NOX1	X:100098313-100129334	4.96	3.02	2.19	4.30	4.03	5.90	D	D	N
GUCA1A	6:42123144-42147794	4.96	2.02	3.28	5.37	3.80	6.12	D	D	N
DLX3	17:48067369-48072588	4.96	3.02	2.19	4.30	4.03	5.90	D	D	N
PRDM13	6:100054606-100063454	4.96	4.03	3.28	5.37	4.26	5.66	D	D	N
ABCC11	16:48200821-48281479	4.96	2.02	0.00	5.37	3.05	6.87	D	D	N

TMEM35	X:100333709-100351353	4.96	2.02	2.19	5.37	3.63	6.29	D	D	N
CATSPERB	14:92047040-92247051	4.96	3.02	3.28	5.37	4.08	5.84	D	D	N
TRIM54	2:27505260-27530307	4.96	1.01	2.19	5.37	3.37	6.55	D	D	N
PRDM14	8:70963886-70983928	4.96	3.02	2.19	4.30	4.03	5.90	D	D	N
CXCL13	4:78432907-78532988	4.96	2.02	2.19	5.37	3.63	6.29	D	D	N
TXLNB	6:139561198-139613276	4.96	3.02	2.19	5.37	3.82	6.10	D	D	N
AVPR1A	12:63539014-63544722	4.96	3.02	3.28	5.37	4.08	5.84	D	D	N
C11orf85	11:64704989-64739557	4.96	1.01	2.19	5.37	3.37	6.55	D	D	N
SH3TC2	5:148303202-148442726	4.96	2.02	3.28	4.30	4.00	5.92	D	D	N
DIRC1	2:189598882-189654831	4.96	4.03	2.19	4.30	4.07	5.85	D	D	N
SSTR3	22:37600278-37608362	4.96	0.00	0.00	4.30	2.95	6.98	D	D	N
C1orf168	1:57184477-57285369	4.96	3.02	1.09	4.30	3.68	6.24	D	D	N
CD247	1:167399877-167487847	4.96	3.02	2.19	5.37	3.82	6.10	D	D	N
MYH11	16:15797029-15950890	5.95	2.02	3.28	7.52	4.08	7.83	D	D	N
ATP5EP2	13:28519343-28519727	5.95	3.02	3.28	7.52	4.33	7.58	D	D	N
KCNH4	17:40308909-40333296	5.95	2.02	4.38	5.37	4.65	7.25	D	D	N
NLRC4	2:32449522-32490923	5.95	4.03	3.28	5.37	5.04	6.87	D	D	N
USP6	17:5019733-5078329	5.95	1.01	2.19	6.45	3.92	7.98	D	D	N
HIST1H2AE	6:26217165-26217711	5.95	4.03	2.19	6.45	4.49	7.41	D	D	N
AC021860.1	4:38628029-38666430	5.95	4.03	4.38	5.37	5.29	6.62	D	D	N
SULT1C4	2:108994367-109004513	5.95	1.01	0.00	4.30	3.87	8.04	D	D	N
PCDHA13	5:140261793-140391929	5.95	4.03	3.28	6.45	4.82	7.09	D	D	N
AANAT	17:74449433-74466199	6.95	0.00	2.19	5.37	4.60	9.29	D	D	N
SLC13A5	17:6588032-6616886	6.95	5.04	4.38	6.45	6.05	7.84	D	D	N
CRIP1	14:105952654-105955284	6.95	5.04	3.28	6.45	5.71	8.18	D	D	N
C11orf94	11:45928085-45928833	6.95	4.03	2.19	6.45	5.29	8.60	D	D	N
SEL1L2	20:13829893-13977089	7.94	3.02	5.47	9.67	5.76	10.11	D	D	N
ABHD1	2:27346682-27353680	7.94	3.02	5.47	9.67	5.76	10.11	D	D	N
ASB4	7:95107756-95169544	7.94	3.02	4.38	6.45	6.31	9.57	D	D	N
GNRH2	20:3024268-3026393	7.94	3.02	3.28	6.45	6.13	9.75	D	D	N
KLRF1	12:9980077-9997606	7.94	2.02	5.47	6.45	6.05	9.82	D	D	N
GPR110	6:46965440-47010099	7.94	5.04	4.38	7.52	6.61	9.27	D	D	N
RNF183	9:116059373-116065656	7.94	6.05	3.28	7.52	6.36	9.52	D	D	N
IRX2	5:2745959-2752969	7.94	3.02	2.19	6.45	5.88	9.99	D	D	N
SUMO4	6:149721495-149722177	7.94	7.06	6.57	7.52	7.49	8.38	D	D	N
CLRN3	10:129676105-129691211	8.93	3.02	5.47	10.74	6.34	11.52	D	D	N
KRT23	17:39078948-39093886	8.93	2.02	2.19	6.45	6.39	11.46	D	D	N
C16orf71	16:4784273-4799397	8.93	6.05	6.57	8.59	7.85	10.01	D	D	N
VCX3A	X:6451659-6453159	8.93	7.06	5.47	9.67	7.51	10.35	D	D	N
HES5	1:2460184-2461684	8.93	6.05	5.47	9.67	7.37	10.49	D	D	N
ZNF812	19:9800600-9811452	8.93	4.03	4.38	9.67	6.71	11.15	D	D	N
ASB14	3:57302375-57326710	9.92	4.03	6.57	11.82	7.32	12.52	D	D	N
ABCB1	7:87133175-87342611	9.92	3.02	3.28	7.52	7.40	12.44	D	D	N
RHOXF1	X:119243011-119249847	9.92	1.01	3.28	7.52	6.90	12.94	D	D	N
TMC5	16:19421818-19510435	9.92	5.04	5.47	9.67	7.95	11.89	D	D	N
CYP1A1	15:75011883-75017951	9.92	5.04	2.19	8.59	7.30	12.55	D	D	N
SDK1	7:3341080-4308632	9.92	8.06	5.47	10.74	8.17	11.67	D	D	N
TNFRSF13C	22:42321045-42322822	9.92	5.04	5.47	8.59	8.14	11.71	D	D	N
TMPRSS5	11:113558272-113577095	9.92	8.06	6.57	10.74	8.52	11.33	D	D	N
CADM2	3:85008132-86123579	9.92	4.03	6.57	9.67	7.82	12.02	D	D	N
IFI30	19:18283972-18288927	9.92	3.02	1.09	7.52	6.89	12.96	D	D	N
RP11-863K10.7	8:37592279-37594944	10.91	8.06	5.47	9.67	9.15	12.67	D	D	N
AL020996.1	1:26145212-26147288	10.91	6.05	8.76	10.74	9.21	12.61	D	D	N
IDS	X:148564074-148615470	10.91	3.02	4.38	8.59	8.17	13.66	D	D	N
RP11-712L6.5	11:126164193-126174213	10.91	4.03	6.57	9.67	8.59	13.24	D	D	N
C16orf95	16:87117168-87351022	10.91	7.06	5.47	9.67	9.06	12.76	D	D	N

MFNG	22:37865101-37882439	11.91	6.05	4.38	12.89	8.74	15.07	D	D	N
PPFIA2	12:81652045-82153332	11.91	6.05	5.47	11.82	9.26	14.55	D	D	N
IRF8	16:85932409-85956215	11.91	7.06	1.09	8.59	8.51	15.30	D	D	N
FAM183B	7:38724946-38726637	11.91	7.06	4.38	12.89	8.88	14.93	D	D	N
FLJ00273	9:35042390-35042824	11.91	6.05	7.66	12.89	9.44	14.37	D	D	N
HSD17B3	9:98997588-99064434	12.90	10.08	9.85	12.89	11.63	14.17	D	D	N
VANGL2	1:160370376-160398468	12.90	6.05	7.66	10.74	10.60	15.20	D	D	N
RPRM	2:154333852-154335322	12.90	8.06	7.66	11.82	10.92	14.88	D	D	N
AC010536.1	16:87727144-87729753	13.89	8.06	7.66	16.11	10.72	17.06	D	D	N
HGFAC	4:3443614-3451211	13.89	10.08	6.57	11.82	11.57	16.21	D	D	N
PACRG	6:163148164-163736524	13.89	8.06	6.57	11.82	11.37	16.41	D	D	N
GATSL2	7:74807499-74867509	13.89	10.08	8.76	12.89	12.10	15.68	D	D	N
GOLGA8O	15:32737307-32747835	13.89	11.09	12.04	13.96	12.83	14.95	D	D	N
BCAS1	20:52553316-52687304	14.88	10.08	13.14	13.96	13.32	16.45	D	D	N
ZNF541	19:48023942-48059113	14.88	9.07	7.66	13.96	12.21	17.55	D	D	N
ELMO1	7:36893961-37488852	14.88	10.08	13.14	15.04	13.15	16.61	D	D	N
ARMC4	10:28064115-28287977	14.88	3.02	4.38	10.74	10.72	19.05	D	D	N
TBC1D3	17:36337711-36358166	14.88	8.06	6.57	12.89	11.94	17.83	D	D	N
MTFP1	22:30821518-30825045	14.88	5.04	8.76	17.19	10.71	19.06	D	D	N
PCDHGB1	5:140729828-140892546	14.88	6.05	8.76	12.89	11.89	17.87	D	D	N
AL807752.1	9:139980807-139981121	15.87	10.08	5.47	17.19	11.81	19.94	D	D	N
SMPD3	16:68392231-68482591	16.87	10.08	12.04	17.19	14.21	19.52	D	D	N
SYT3	19:51124564-51171651	16.87	12.09	12.04	16.11	14.94	18.79	D	D	N
ZFP92	X:152683780-152687087	17.86	12.09	10.95	21.48	14.14	21.57	D	D	N
MASP2	1:11086580-11107290	17.86	14.11	7.66	18.26	14.18	21.54	D	D	N
ODAM	4:71062213-71070293	17.86	6.05	8.76	15.04	13.76	21.95	D	D	N
NUDT10	X:51075083-51080377	17.86	9.07	12.04	16.11	14.88	20.84	D	D	N
MST1R	3:49924435-49941299	17.86	12.09	14.23	19.34	15.38	20.34	D	D	N
AMER3	2:131513008-131525707	17.86	7.06	9.85	17.19	13.83	21.88	D	D	N
ZNF763	19:12035890-12091196	17.86	8.06	10.95	17.19	14.27	21.45	D	D	N
HDAC10	22:50683612-50689834	18.85	12.09	16.42	17.19	16.69	21.01	D	D	N
EYA4	6:133561736-133853258	18.85	16.13	12.04	19.34	16.34	21.36	D	D	N
RAB39A	11:107799229-107834208	18.85	8.06	13.14	17.19	15.25	22.46	D	D	N
PHF21B	22:45277042-45405880	19.84	17.13	13.14	18.26	17.70	21.99	D	D	N
DLL3	19:39989535-39999121	19.84	15.12	16.42	19.34	18.14	21.55	D	D	N
ESAM	11:124622026-124632186	19.84	13.10	15.33	21.48	16.93	22.76	D	D	N
MEI1	22:42095503-42195460	20.84	12.09	15.33	23.63	16.92	24.75	D	D	N
FAM89B	11:65339820-65341669	20.84	7.06	15.33	18.26	16.35	25.32	D	D	N
CYP2D6	22:42522501-42526908	21.83	7.06	16.42	21.48	16.66	26.99	D	D	N
COLEC11	2:3642426-3692048	21.83	13.10	15.33	20.41	18.73	24.92	D	D	N
ADORA2A	22:24813847-24838328	21.83	11.09	7.66	19.34	16.81	26.85	D	D	N
CABP1	12:121078355-121105127	21.83	14.11	13.14	20.41	18.54	25.11	D	D	N
CCDC116	22:21987005-21991616	25.80	13.10	14.23	22.56	21.13	30.47	D	D	N
ERVMER34-1	4:53588785-53617807	25.80	21.17	12.04	21.48	21.46	30.13	D	D	N
ADAMTSL4-AS1	1:150533480-150533969	26.79	14.11	15.33	33.30	19.85	33.73	D	D	N
PRR5L	11:36317838-36486754	26.79	24.19	22.99	26.86	25.34	28.23	D	D	N
RUNDC3A	17:42385781-42396039	27.78	18.14	17.52	24.71	24.01	31.55	D	D	N
DIO2	14:80663873-80854100	27.78	23.18	26.28	26.86	26.29	29.28	D	D	N
AP003068.23	11:64945077-64949305	27.78	23.18	17.52	25.78	24.44	31.12	D	D	N
NFATC2	20:50003494-50179370	28.77	21.17	19.71	29.00	25.09	32.46	D	D	N
SIM2	21:38071433-38122218	28.77	16.13	14.23	25.78	23.42	34.12	D	D	N
LYL1	19:13209847-13213975	29.76	19.15	21.90	26.86	26.18	33.35	D	D	N
C16orf98	16:31213206-31214773	29.76	21.17	20.80	27.93	26.31	33.22	D	D	N
KIF5A	12:57943781-57980415	30.76	16.13	17.52	33.30	24.11	37.40	D	D	N
B3GALT1	2:168675182-168730551	30.76	23.18	27.37	32.23	27.74	33.77	D	D	N
LIN28B	6:105404923-105531207	30.76	15.12	16.42	26.86	24.97	36.54	D	D	N

SHBG	17:7517382-7536700	31.75	23.18	20.80	33.30	27.11	36.39	D	D	N
FMO5	1:146646930-146714700	32.74	17.13	21.90	27.93	27.62	37.86	D	D	N
SMIM1	1:3689352-3692546	32.74	26.21	14.23	27.93	26.84	38.64	D	D	N
INSL3	19:17927321-17932383	32.74	28.22	21.90	34.38	28.55	36.93	D	D	N
IQUB	7:123092454-123175131	33.73	29.23	21.90	30.08	30.01	37.45	D	D	N
TMEM229B	14:67913801-68000456	34.73	26.21	29.56	33.30	31.85	37.60	D	D	N
LPPR3	19:812518-821967	35.72	25.20	31.75	35.45	32.04	39.39	D	D	N
C1QL4	12:49726200-49730971	35.72	27.21	22.99	35.45	31.00	40.44	D	D	N
SLC10A5	8:82605842-82608409	36.71	31.25	28.47	38.67	33.16	40.26	D	D	N
TSNAXIP1	16:67840668-67866051	37.70	30.24	22.99	37.60	32.43	42.97	D	D	N
OPRL1	20:62711526-62731996	37.70	17.13	24.09	35.45	30.44	44.97	D	D	N
C1orf210	1:43747554-43751288	37.70	14.11	21.90	30.08	30.06	45.35	D	D	N
GLIPR1L2	12:75784850-75826468	39.69	30.24	29.56	44.04	34.33	45.04	D	D	N
RET	10:43572475-43625799	39.69	26.21	32.85	36.52	35.34	44.03	D	D	N
C10orf35	10:71390007-71393352	39.69	29.23	31.75	40.82	35.38	44.00	D	D	N
AC011484.1	19:46997765-46999755	39.69	23.18	17.52	36.52	31.75	47.63	D	D	N
FOXO6	1:41827594-41849262	40.68	22.17	26.28	36.52	34.20	47.15	D	D	N
GRB7	17:37894180-37903544	41.67	27.21	31.75	45.12	35.40	47.94	D	D	N
ACAP1	17:7239848-7254797	42.66	30.24	25.18	38.67	36.72	48.61	D	D	N
MFSD7	4:675618-683230	44.65	37.29	24.09	40.82	37.95	51.35	D	D	N
TNFRSF18	1:1138888-1142071	44.65	24.19	24.09	38.67	36.84	52.45	D	D	N
MMP24	20:33814457-33864801	45.64	25.20	25.18	39.75	37.85	53.43	D	D	N
TEX22	14:105864916-105916443	45.64	37.29	36.13	46.19	41.64	49.64	D	D	N
RHCE	1:25688740-25756683	46.63	32.25	38.32	52.64	39.89	53.37	D	D	N
AMN	14:103388993-103399933	47.62	26.21	9.85	39.75	35.20	60.04	D	D	N
KDM4D	11:94706845-94732682	47.62	31.25	37.23	46.19	41.82	53.42	D	D	N
SLC22A31	16:89262406-89268072	47.62	31.25	40.51	44.04	42.35	52.90	D	D	N
RP11-287D1.3	2:74438731-74590504	47.62	31.25	37.23	45.12	42.01	53.24	D	D	N
LIN37	19:36239262-36245420	47.62	24.19	32.85	48.34	38.79	56.46	D	D	N
C1orf213	1:23695490-23698332	48.62	31.25	39.42	45.12	42.93	54.30	D	D	N
LIPE	19:42905659-42931578	49.61	34.27	35.04	45.12	43.93	55.28	D	D	N
NPIPB11	16:29392675-29415350	49.61	31.25	31.75	50.49	41.57	57.65	D	D	N
SCARF1	17:1537152-1549041	50.60	41.32	29.56	45.12	43.92	57.28	D	D	N
TLR6	4:38825336-38858438	50.60	39.31	31.75	45.12	44.55	56.65	D	D	N
C18orf56	18:641320-658340	51.59	49.39	45.99	51.56	49.61	53.57	D	D	N
CLEC14A	14:38723308-38725574	52.58	42.33	45.99	56.93	47.68	57.49	D	D	N
LRRC73	6:43474707-43478424	52.58	31.25	41.61	48.34	45.61	59.56	D	D	N
CCDC88B	11:64107695-64125006	54.57	28.22	44.89	52.64	45.58	63.56	D	D	N
EPHB3	3:184279572-184300197	55.56	35.28	42.70	49.41	49.01	62.11	D	D	N
EYA2	20:45523263-45817492	56.55	37.29	48.18	54.79	50.01	63.09	D	D	N
CCDC7	10:32735068-32863492	56.55	42.33	33.94	54.79	48.52	64.59	D	D	N
SEMA3F	3:50192478-50226508	59.53	44.35	28.47	52.64	49.50	69.56	D	D	N
PRR22	19:5782971-5784776	60.52	42.33	41.61	56.93	53.18	67.86	D	D	N
FAM198A	3:43020759-43101703	63.50	50.40	43.80	60.16	56.73	70.27	D	D	N
OTUB2	14:94492675-94515276	64.49	62.49	60.22	64.45	62.97	66.01	D	D	N
SYNE4	19:36494209-36499695	64.49	48.38	52.56	68.75	57.26	71.72	D	D	N
CARD9	9:139256355-139268133	64.49	57.45	50.37	60.16	60.05	68.93	D	D	N
CENPP	9:95087766-95382815	66.47	60.47	60.22	65.53	64.01	68.94	D	D	N
RPS17	15:82821158-82824972	67.47	58.46	60.22	65.53	64.27	70.67	D	D	N
FAM151B	5:79783788-79838382	68.46	51.40	52.56	66.60	61.69	75.23	D	D	N
HCN3	1:155247374-155259639	71.44	58.46	60.22	73.05	65.80	77.07	D	D	N
SLC37A1	21:43916118-44001550	71.44	54.43	51.46	67.68	64.10	78.78	D	D	N
FAM131C	1:16384264-16400127	76.40	60.47	54.75	69.82	69.17	83.62	D	D	N
ARHGEF16	1:3370990-3397677	77.39	58.46	68.98	77.34	70.66	84.12	D	D	N
PGF	14:75408537-75422487	79.37	57.45	63.51	74.12	71.91	86.83	D	D	N
ZNF169	9:97021593-97063736	80.36	72.57	68.98	85.94	74.64	86.09	D	D	N

TDRKH	1:151742583-151763892	81.36	74.58	68.98	83.79	76.33	86.39	D	D	N
GPSM3	6:32158543-32163300	81.36	63.50	53.65	74.12	72.25	90.46	D	D	N
ZDHHC14	6:157802165-158099178	82.35	66.52	70.08	82.72	76.09	88.61	D	D	N
HSPA1L	6:31777396-31783437	82.35	77.61	75.55	81.64	79.91	84.79	D	D	N
ICOSLG	21:45642874-45660849	83.34	77.61	68.98	79.49	78.79	87.89	D	D	N
RILP	17:1549444-1553371	83.34	58.46	74.46	83.79	74.46	92.22	D	D	N
ALDH4A1	1:19197926-19229275	86.32	77.61	81.03	87.01	82.96	89.67	D	D	N
NOXA1	9:140317802-140328858	86.32	81.64	79.93	87.01	83.71	88.92	D	D	N
LIN7B	19:49617581-49621717	89.29	58.46	58.03	84.86	76.74	101.85	D	D	N
CD72	9:35609530-35646807	90.29	61.48	54.75	80.57	77.92	102.65	D	D	N
EEPD1	7:36192758-36341152	91.28	87.69	87.60	90.23	89.89	92.66	D	D	N
FIBCD1	9:133777825-133814673	93.26	74.58	68.98	90.23	84.40	102.12	D	D	N
RAD51D	17:33426811-33448541	99.22	88.70	83.22	97.75	93.52	104.91	D	D	N
SH2B2	7:101928405-101962178	106.16	85.67	74.46	116.02	91.97	120.35	D	D	N
ZNF691	1:43312280-43318148	106.16	87.69	74.46	97.75	95.92	116.40	D	D	N
POLD4	11:67118248-67124443	109.14	93.74	93.07	111.72	101.72	116.55	D	D	N
POC1A	3:52109269-52188706	110.13	88.70	100.74	106.35	103.11	117.15	D	D	N
DLX2	2:172964167-172967628	111.12	86.68	75.55	109.57	98.01	124.24	D	D	N
C16orf93	16:30768744-30774031	111.12	95.75	90.88	111.72	103.14	119.10	D	D	N
PRSS21	16:2867164-2876305	113.11	67.53	88.69	99.90	98.63	127.58	D	D	N
C2orf81	2:74641304-74648718	115.09	94.74	78.84	126.76	99.12	131.06	D	D	N
CBWD5	9:70432004-70497240	117.07	110.87	105.12	120.31	112.02	122.13	D	D	N
HMHA1	19:1065922-1086627	118.07	85.67	106.21	114.94	107.12	129.01	D	D	N
LPAR2	19:19734477-19739739	123.03	115.91	110.59	123.54	118.39	127.67	D	D	N
TMEM88	17:7758383-7759417	124.02	94.74	78.84	114.94	108.79	139.25	D	D	N
PIWIL4	11:94277006-94354587	125.01	106.84	114.97	132.13	116.68	133.35	D	D	N
PRSS27	16:2762419-2770552	126.00	104.82	96.36	117.09	116.18	135.83	D	D	N
METTL12	11:62432781-62435968	126.00	121.96	114.97	125.68	122.16	129.85	D	D	N
BOP1	8:145486055-145515082	131.96	89.70	87.60	117.09	115.76	148.16	D	D	N
ZNF517	8:146024261-146036554	131.96	118.93	109.50	129.98	124.13	139.79	D	D	N
SLC26A10	12:58013310-58019934	134.93	115.91	105.12	135.35	123.77	146.10	D	D	N
ATP2A1	16:28889726-28915830	135.93	109.86	112.78	128.91	126.50	145.35	D	D	N
TMEM91	19:41856816-41889988	136.92	107.85	83.22	123.54	119.62	154.22	D	D	N
ADAMTS15	11:130318869-130346532	138.90	102.81	82.12	123.54	120.36	157.45	D	D	N
GPR37	7:124386051-124405681	138.90	125.99	122.64	141.80	131.83	145.98	D	D	N
KRT80	12:52562780-52585784	142.87	89.70	112.78	129.98	125.62	160.12	D	D	N
PRRT1	6:32116136-32122150	146.84	118.93	90.88	131.06	129.11	164.57	D	D	N
FAAH	1:46859937-46879520	151.80	102.81	111.69	146.09	133.46	170.14	D	D	N
SLC2A11	22:24198890-24228496	156.76	123.97	145.63	148.24	146.30	167.23	D	D	N
GRK4	4:2965335-3042474	159.74	109.86	128.11	146.09	143.47	176.00	D	D	N
GPR143	X:9693386-9754337	161.72	145.14	122.64	151.47	149.31	174.13	D	D	N
ZNF551	19:58193337-58228669	163.71	145.14	108.40	150.39	145.96	181.45	D	D	N
EPHX2	8:27348296-27403081	164.70	146.15	145.63	157.91	157.71	171.69	D	D	N
NPIPB15	16:74411776-74425978	166.68	125.99	123.73	152.54	151.01	182.36	D	D	N
RINL	19:39358470-39368919	168.67	120.95	119.35	170.80	147.18	190.15	D	D	N
PLIN5	19:4522543-4535236	168.67	150.18	131.40	162.21	156.38	180.95	D	D	N
PCDH7	4:30722037-31148422	170.65	137.07	142.35	163.28	158.53	182.77	D	D	N
C20orf24	20:35234137-35240960	171.64	130.02	91.98	171.88	142.87	200.42	D	D	N
ZNF334	20:45129701-45142198	171.64	164.29	162.06	174.02	167.34	175.94	D	D	N
NDUFA9	12:4758261-4798454	173.63	160.26	144.54	183.69	160.90	186.36	D	D	N
MMP15	16:58059470-58080805	178.59	160.26	128.11	176.17	161.15	196.02	D	D	N
LIMD2	17:61773262-61778532	181.56	159.25	134.68	194.43	161.87	201.26	D	D	N
RAB24	5:176728199-176730745	189.50	167.31	140.16	184.77	172.76	206.25	D	D	N
MAMSTR	19:49215999-49222978	189.50	167.31	162.06	185.84	179.36	199.65	D	D	N
LOH12CR1	12:12510013-12619840	192.48	180.41	169.72	195.51	183.61	201.35	D	D	N
HTRA3	4:8271492-8308838	192.48	171.34	152.20	182.62	179.52	205.44	D	D	N

HOXC9	12:54388679-54397121	195.46	160.26	169.72	186.91	183.46	207.45	D	D	N
SMIM4	3:52568029-52613253	203.39	156.23	180.67	212.70	184.47	222.32	D	D	N
FNTB	14:65381203-65529368	208.35	176.38	143.44	223.44	181.69	235.01	D	D	N
CCDC23	1:43272723-43282954	216.29	202.59	193.81	211.62	208.82	223.76	D	D	N
ZNF324	19:58978423-58984945	220.26	191.50	186.15	211.62	208.11	232.41	D	D	N
ZBTB20	3:114056941-114866118	223.24	203.60	202.57	228.81	213.15	233.32	D	D	N
GJC2	1:228337553-228347527	225.22	204.60	177.39	236.33	205.79	244.65	D	D	N
MOCS1	6:39867354-39902290	234.15	183.44	183.96	216.99	215.27	253.03	D	D	N
ZNF554	19:2819872-2835771	242.09	203.60	202.57	243.85	224.81	259.37	D	D	N
USP2	11:119225925-119252436	247.05	221.74	213.52	256.74	231.71	262.39	D	D	N
ZNF362	1:33722146-33766320	258.95	232.83	227.76	266.41	244.66	273.25	D	D	N
GTF2H2C	5:68856035-68890550	258.95	250.97	248.56	258.89	254.92	262.99	D	D	N
SCNN1D	1:1215816-1227409	263.91	188.48	231.04	248.15	239.53	288.30	D	D	N
MAP4K2	11:64556290-64570713	263.91	220.73	181.77	248.15	236.93	290.90	D	D	N
ZBTB9	6:33422356-33425325	270.86	261.05	259.51	276.08	264.91	276.81	D	D	N
MUTYH	1:45794835-45806142	270.86	237.87	222.28	257.81	254.77	286.95	D	D	N
SETMAR	3:4344988-4359251	271.85	229.80	249.66	266.41	257.65	286.06	D	D	N
DENND6B	22:50747459-50765489	271.85	237.87	216.81	258.89	253.73	289.98	D	D	N
FIZ1	19:56102746-56113336	274.83	225.77	233.23	283.60	253.02	296.64	D	D	N
ACTR5	20:37377085-37400834	281.77	240.89	258.42	292.19	264.45	299.10	D	D	N
PSD	10:104162376-104181296	285.74	264.07	245.28	280.37	272.07	299.41	D	D	N
ALDH16A1	19:49956426-49974305	287.73	255.00	235.42	305.08	264.16	311.30	D	D	N
IKZF4	12:56401443-56432219	291.69	257.02	205.86	293.26	260.97	322.42	D	D	N
CLDN15	7:100875373-100882101	293.68	246.94	250.75	297.56	273.36	314.00	D	D	N
FANCC	9:97861336-98079991	303.60	283.22	285.79	301.86	295.65	311.55	D	D	N
ZNF689	16:30613879-30635333	304.59	269.11	246.37	296.49	284.64	324.54	D	D	N
SGK223	8:8175258-8244008	305.58	242.90	222.28	280.37	277.60	333.57	D	D	N
CEP85	1:26560691-26605299	312.53	224.76	232.14	284.67	280.90	344.16	D	D	N
PLCD1	3:38048987-38071253	325.43	278.18	263.89	321.19	302.34	348.52	D	D	N
TMEM141	9:139685807-139687709	326.42	223.75	241.99	299.71	290.31	362.53	D	D	N
FOXO4	X:70316047-70323385	328.40	281.21	238.71	346.97	291.87	364.94	D	D	N
SNRNP35	12:123942188-123957701	331.38	290.28	308.79	321.19	318.11	344.65	D	D	N
TOE1	1:45805342-45809647	335.35	307.41	291.27	338.38	318.34	352.36	D	D	N
TREX1	3:48506445-48509044	336.34	280.20	256.23	314.75	309.62	363.06	D	D	N
BABAM1	19:17378159-17392058	339.32	250.97	272.65	312.60	309.61	369.03	D	D	N
NAIF1	9:130823512-130830485	339.32	309.43	326.30	345.90	327.25	351.38	D	D	N
CTU1	19:51600863-51611627	345.27	258.02	279.22	352.35	309.88	380.66	D	D	N
CDK20	9:90581356-90589668	349.24	308.42	304.41	364.16	326.96	371.52	D	D	N
PAFAH1B3	19:42801185-42807698	357.18	278.18	323.02	359.86	328.57	385.78	D	D	N
TRAF1	9:123664671-123691451	367.10	306.40	271.56	339.45	336.10	398.09	D	D	N
HAUS5	19:36103646-36116251	371.07	344.70	338.35	377.05	356.73	385.41	D	D	N
SPATA2L	16:89762751-89768113	372.06	325.55	340.54	360.94	356.49	387.63	D	D	N
FAM98C	19:38893775-38899728	375.04	309.43	332.87	356.64	353.64	396.43	D	D	N
KRBA1	7:149411872-149431664	375.04	338.66	344.92	383.50	358.49	391.58	D	D	N
NAGPA	16:5074845-5084142	377.02	344.70	356.96	381.35	364.11	389.93	D	D	N
NR2C2AP	19:19312225-19314233	377.02	340.67	338.35	374.90	361.21	392.83	D	D	N
FAM173A	16:770581-772601	378.01	319.51	336.16	394.24	351.79	404.24	D	D	N
MKS1	17:56282803-56296966	381.98	349.74	335.06	401.76	359.27	404.69	D	D	N
MYO16	13:109248500-109860355	384.96	371.92	354.77	392.09	372.66	397.25	D	D	N
TIGD5	8:144680074-144682485	396.86	354.78	294.55	417.87	356.07	437.65	D	D	N
RCE1	11:66610306-66614017	397.86	357.81	325.21	424.32	365.10	430.61	D	D	N
GSTZ1	14:77787227-77797940	405.79	346.72	370.10	406.06	384.01	427.58	D	D	N
PGP	16:2261998-2264808	411.75	332.61	329.59	412.50	376.65	446.84	D	D	N
IL11RA	9:34650699-34661889	413.73	377.96	316.45	395.31	382.07	445.39	D	D	N
CHCHD10	22:24108021-24110630	422.66	347.73	366.82	453.32	386.00	459.32	D	D	N
COX8A	11:63742079-63744015	422.66	313.46	353.68	443.65	377.35	467.96	D	D	N

DCAF4	14:73393040-73426411	427.62	393.08	386.53	430.76	410.42	444.82	D	D	N
TMCO6	5:140019012-140024993	430.60	369.90	383.24	444.73	403.48	457.71	D	D	N
ZBTB45	19:59024897-59050278	439.53	356.80	266.08	386.72	385.01	494.04	D	D	N
ZNF710	15:90544624-90625438	441.51	385.02	362.44	444.73	410.66	472.36	D	D	N
PTPRN	2:220154345-220174370	443.49	418.28	382.15	447.95	420.85	466.13	D	D	N
NOD1	7:30464143-30518400	460.36	411.22	388.72	457.62	433.88	486.84	D	D	N
KLHL26	19:18747775-18781309	466.31	380.99	353.68	430.76	428.57	504.06	D	D	N
TIMM10	11:57295936-57298276	483.18	456.58	450.04	478.03	471.09	495.27	D	D	N
TGIF2	20:35201891-35222353	485.17	453.56	452.23	495.22	468.72	501.61	D	D	N
HIRA	22:19318221-19435224	488.14	452.55	475.22	487.70	475.63	500.65	D	D	N
SPATA2	20:48519928-48532080	488.14	474.72	471.94	486.62	481.98	494.30	D	D	N
GFOD2	16:67708434-67753324	491.12	451.54	423.76	510.26	461.93	520.30	D	D	N
SLC19A1	21:46913486-46964325	492.11	444.49	417.19	493.07	464.09	520.13	D	D	N
DEDD2	19:42702750-42724292	493.10	439.45	442.37	512.40	465.66	520.54	D	D	N
LTB4R	14:24780656-24787242	495.09	465.65	424.85	488.77	471.25	518.93	D	D	N
C19orf54	19:41246761-41257458	497.07	452.55	456.61	501.66	477.59	516.56	D	D	N
RASL11A	13:27844464-27847827	499.06	419.29	454.42	493.07	471.19	526.92	D	D	N
ZNF628	19:55987699-55995854	500.05	425.34	372.29	468.36	458.51	541.58	D	D	N
FUK	16:70488324-70514177	505.01	454.56	409.52	501.66	471.18	538.83	D	D	N
IGHMBP2	11:68671310-68708070	508.98	488.83	473.03	503.81	496.83	521.12	D	D	N
PLA2G15	16:68279207-68294961	510.96	462.63	459.89	536.04	482.93	539.00	D	D	N
PRR3	6:30524663-30531500	516.91	452.55	453.32	500.59	492.23	541.60	D	D	N
LRRC8E	19:7953390-7966901	531.80	510.00	511.36	525.30	523.80	539.80	D	D	N
METTL23	17:74722912-74730018	532.79	454.56	447.85	504.89	502.21	563.36	D	D	N
ARRDC1	9:140500106-140509812	532.79	495.89	474.13	523.15	512.81	552.77	D	D	N
ZDHHC12	9:131483148-131486406	533.78	446.50	471.94	538.19	499.61	567.95	D	D	N
RABEP2	16:28915742-28947847	539.73	493.87	447.85	540.33	506.63	572.84	D	D	N
PHKG2	16:30759591-30772490	542.71	463.64	478.51	521.00	515.18	570.24	D	D	N
ICAM5	19:10400657-10407454	547.67	468.68	459.89	525.30	515.55	579.79	D	D	N
CHST12	7:2443223-2474242	551.64	497.90	526.69	567.19	528.91	574.37	D	D	N
ORAI3	16:30960387-30967782	556.60	513.02	468.65	542.48	527.46	585.75	D	D	N
HOMER3	19:19040010-19052070	560.57	539.23	505.88	555.37	542.08	579.06	D	D	N
URB2	1:229761981-229795946	560.57	505.97	499.31	558.60	535.82	585.32	D	D	N
ARL2	11:64781585-64789656	561.56	444.49	474.13	531.74	521.61	601.51	D	D	N
TMEM8B	9:35814448-35854844	573.47	508.99	476.32	586.53	534.14	612.79	D	D	N
SLC47A1	17:19398698-19482347	579.42	522.09	487.27	551.08	549.81	609.03	D	D	N
SLC22A18	11:2920951-2946476	582.40	536.20	517.93	568.26	560.33	604.46	D	D	N
RAB40C	16:639357-679272	584.38	501.94	528.88	558.60	557.55	611.22	D	D	N
ABCD4	14:74752126-74769759	586.37	527.13	494.93	558.60	556.74	615.99	D	D	N
TRNAU1AP	1:28879597-28905051	586.37	537.21	493.84	570.41	555.69	617.04	D	D	N
KIFC2	8:145691426-145699585	595.29	557.37	488.36	592.97	557.89	632.70	D	D	N
ZNF646	16:31085743-31095517	596.29	559.39	516.83	604.79	566.17	626.41	D	D	N
FN3KRP	17:80674559-80688204	606.21	576.52	562.82	602.64	590.55	621.87	D	D	N
HYAL2	3:50355221-50360337	614.15	556.36	490.55	613.38	570.16	658.14	D	D	N
FBXL15	10:104178946-104182893	618.11	573.50	550.78	619.83	592.53	643.69	D	D	N
RFX5	1:151313116-151319833	628.04	573.50	513.55	609.08	590.27	665.80	D	D	N
MIIP	1:12079523-12092102	638.95	579.54	580.34	621.98	616.46	661.44	D	D	N
TIMM22	17:900357-906911	641.93	606.76	609.91	655.28	623.99	659.86	D	D	N
USE1	19:17326155-17330638	647.88	568.46	549.68	615.53	614.41	681.35	D	D	N
HCFC1R1	16:3072621-3074287	660.78	524.11	493.84	646.68	597.27	724.29	D	D	N
ARHGEF9	X:62854847-63005426	663.75	547.29	539.83	638.09	616.55	710.96	D	D	N
RPUSD1	16:834974-838397	664.75	602.73	594.58	650.98	638.64	690.85	D	D	N
ZNF580	19:56146382-56154835	685.58	570.47	509.17	672.46	622.32	748.84	D	D	N
GPR137	11:64037534-64056972	688.56	587.61	603.34	697.17	646.03	731.09	D	D	N
NPRL2	3:50384761-50388522	690.54	642.03	616.48	724.03	654.38	726.71	D	D	N
DPH7	9:140449356-140473387	702.45	652.11	596.77	697.17	665.64	739.26	D	D	N

POLR2I	19:36604612-36606248	712.37	536.20	581.44	664.94	652.67	772.06	D	D	N
AHDC1	1:27860546-27930942	718.32	664.21	571.58	690.73	670.54	766.10	D	D	N
MGMT	10:131265448-131566271	718.32	600.71	651.51	740.14	670.57	766.08	D	D	N
MDC1	6:30667584-30685666	728.24	585.59	630.71	686.43	681.35	775.14	D	D	N
WDR91	7:134868590-134896316	729.24	680.34	621.95	733.69	690.11	768.37	D	D	N
PYCARD	16:31212806-31214771	747.10	651.11	555.16	697.17	685.84	808.35	D	D	N
DPP3	11:66247484-66277130	756.02	697.47	667.94	733.69	726.82	785.23	D	D	N
HPS6	10:103825147-103827792	768.92	708.56	700.79	753.03	743.95	793.90	D	D	N
C6orf1	6:34214157-34217247	770.91	655.14	634.00	760.55	717.95	823.86	D	D	N
SFI1	22:31884674-32014572	776.86	658.16	531.07	704.69	699.35	854.37	D	D	N
NMNAT2	1:183217372-183387737	777.85	695.45	727.07	789.55	744.83	810.87	D	D	N
PHLDA2	11:2949503-2950685	778.84	683.36	648.23	820.71	718.46	839.22	D	D	N
VAV2	9:136627016-136857726	783.80	705.53	648.23	745.51	740.33	827.28	D	D	N
EBP	X:48379546-48387104	794.72	728.71	734.73	812.11	763.17	826.27	D	D	N
ISYNA1	19:18545198-18549111	803.65	701.50	648.23	855.08	733.01	874.29	D	D	N
NT5C	17:73126320-73127890	811.59	660.18	733.64	797.07	759.73	863.44	D	D	N
CNPY3	6:42896938-42907025	815.55	727.71	717.21	780.96	780.92	850.19	D	D	N
TRAPPC1	17:7833663-7835441	825.48	765.00	693.12	809.96	780.97	869.98	D	D	N
IQSEC2	X:53262058-53350522	832.42	591.64	733.64	761.62	756.62	908.22	D	D	N
CRIP2	14:105939299-105946499	835.40	768.02	725.97	889.46	781.19	889.60	D	D	N
PNKD	2:219135115-219211516	839.37	755.93	734.73	811.04	803.12	875.61	D	D	N
COQ9	16:57481337-57495187	848.30	831.52	821.24	855.08	836.70	859.89	D	D	N
STK36	2:219536749-219567439	850.28	757.94	678.89	827.15	792.40	908.15	D	D	N
ORAI1	12:122064455-122080583	852.26	795.24	743.49	877.64	807.27	897.26	D	D	N
ZDHHC18	1:27153201-27184093	854.25	751.90	741.30	829.30	812.21	896.29	D	D	N
PRR14	16:30662038-30667761	860.20	774.07	700.79	852.93	803.90	916.50	D	D	N
FAM207A	21:46359925-46396904	867.15	786.17	725.97	863.68	816.33	917.96	D	D	N
RP11-1055B8.7	17:79373540-79433357	876.08	751.90	604.43	829.30	786.89	965.27	D	D	N
JOSD2	19:51009255-51014610	888.97	845.63	792.77	865.82	858.19	919.75	D	D	N
OCIAD2	4:48887036-48908954	889.97	860.75	878.18	886.23	880.22	899.71	D	D	N
USP20	9:132596977-132644107	894.93	789.19	725.97	860.45	838.44	951.42	D	D	N
MRP63	13:21750784-21753223	912.79	815.39	846.42	948.54	867.22	958.35	D	D	N
MYD88	3:38179969-38184513	915.76	842.61	734.73	933.50	848.04	983.49	D	D	N
DCXR	17:79993012-79995608	921.71	839.58	811.38	932.43	876.77	966.66	D	D	N
BOK	2:242498136-242513546	937.59	857.73	881.46	968.95	899.41	975.77	D	D	N
PRAF2	X:48928813-48931730	941.56	800.28	707.36	874.42	866.14	1016.98	D	D	N
COPZ2	17:46103533-46115392	943.54	914.17	872.70	934.57	919.90	967.19	D	D	N
PHF5A	22:41855721-41864729	946.52	881.92	844.23	959.28	905.76	987.28	D	D	N
ISOC2	19:55964352-55973710	949.50	861.76	884.75	939.95	917.62	981.37	D	D	N
ATP5SL	19:41937223-41946622	952.47	820.43	833.28	960.36	896.18	1008.76	D	D	N
ELOF1	19:11661962-11670051	953.46	908.12	858.47	944.24	921.14	985.79	D	D	N
POLR3GL	1:145456236-145470388	955.45	907.11	820.14	924.91	911.91	998.98	D	D	N
MRPS12	19:39421188-39423802	958.42	849.66	871.61	973.25	912.16	1004.69	D	D	N
PPM1M	3:52279841-52284613	958.42	828.50	755.54	904.50	891.96	1024.89	D	D	N
ST6GALNAC4	9:130670165-130679317	968.35	849.66	791.67	933.50	908.26	1028.43	D	D	N
BYSL	6:41888926-41900784	969.34	844.62	842.04	929.20	921.84	1016.84	D	D	N
THYN1	11:134118173-134123264	974.30	960.53	943.88	976.47	963.02	985.58	D	D	N
ACAP3	1:1227756-1244989	980.25	780.12	731.45	896.98	895.75	1064.75	D	D	N
CITED4	1:41326729-41328018	989.18	806.32	721.59	931.35	898.56	1079.80	D	D	N
ENDOG	9:131580753-131584956	993.15	733.75	835.47	932.43	907.90	1078.40	D	D	N
SLC27A1	19:17579578-17616977	994.14	905.10	848.61	960.36	946.23	1042.06	D	D	N
POLL	10:103338639-103348027	997.12	891.99	833.28	967.88	941.47	1052.77	D	D	N
FBXL12	19:9920943-9938492	999.10	903.08	810.29	973.25	935.75	1062.46	D	D	N
C19orf24	19:1275026-1279248	1002.08	885.95	940.59	1019.44	956.36	1047.80	D	D	N
SYNGR1	22:39745930-39781593	1006.05	882.92	830.00	1011.92	938.02	1074.08	D	D	N
CRYL1	13:20977806-21099996	1013.99	991.78	994.24	1025.88	1001.75	1026.22	D	D	N

MRPL23	11:1968508-2005752	1016.96	908.12	905.55	1003.33	972.03	1061.90	D	D	N
TAZ	X:153639854-153650065	1026.88	871.84	798.24	968.95	950.75	1103.02	D	D	N
MPI	15:75182346-75191798	1028.87	877.88	912.12	995.81	976.01	1081.72	D	D	N
DNPH1	6:43193367-43197222	1029.86	916.18	947.16	1044.15	983.07	1076.65	D	D	N
PFDN6	6:33257079-33266178	1035.81	953.48	980.01	1063.48	998.04	1073.58	D	D	N
PUS1	12:132413745-132428406	1039.78	969.60	930.74	1034.48	1000.26	1079.31	D	D	N
GHDC	17:40340817-40346531	1044.74	839.58	800.43	983.99	957.72	1131.76	D	D	N
AAAS	12:53701240-53718648	1047.72	966.58	865.04	1009.77	988.62	1106.82	D	D	N
ATAD3A	1:1447531-1470067	1053.67	949.45	960.30	1038.77	1013.72	1093.63	D	D	N
TAF1C	16:84211458-84220669	1055.66	951.46	831.09	1001.18	983.80	1127.51	D	D	N
PPP2R2D	10:133747955-133773331	1057.64	1000.85	977.82	1050.59	1028.64	1086.64	D	D	N
KIAA0195	17:73437240-73496171	1062.60	965.57	879.27	1061.33	996.65	1128.55	D	D	N
TRMT2A	22:20099389-20104915	1071.53	1038.14	996.43	1095.71	1039.24	1103.82	D	D	N
LRP5	11:68080077-68216743	1071.53	995.81	916.50	1094.63	1011.03	1132.03	D	D	N
POLR2F	22:38348614-38437922	1073.52	1020.00	1011.76	1096.78	1042.58	1104.45	D	D	N
C11orf31	11:57508825-57510986	1074.51	836.56	928.55	1064.56	988.73	1160.28	D	D	N
SLIRP	14:78174414-78227447	1078.48	936.34	983.29	1054.89	1029.49	1127.47	D	D	N
OPLAH	8:145106167-145118735	1079.47	997.82	879.27	1051.67	1013.02	1145.91	D	D	N
PWP2	21:45527171-45551063	1094.35	926.26	873.80	1058.11	1015.59	1173.11	D	D	N
HSPB7	1:16340523-16346089	1095.34	1026.05	969.06	1098.93	1048.81	1141.87	D	D	N
PCIF1	20:44563267-44576662	1098.32	990.77	995.34	1110.75	1049.88	1146.76	D	D	N
ANAPC2	9:140069236-140082989	1098.32	1017.98	938.40	1086.04	1043.15	1153.49	D	D	N
ZNHIT3	17:34842473-34855154	1101.30	995.81	1031.47	1073.15	1066.53	1136.06	D	D	N
ANKRD9	14:102973179-102976136	1102.29	939.37	919.79	1096.78	1028.43	1176.15	D	D	N
HELZ2	20:62189439-62205592	1106.26	1054.27	966.87	1086.04	1060.13	1152.38	D	D	N
THOC6	16:3074028-3077756	1109.23	1067.37	1022.71	1090.34	1081.26	1137.20	D	D	N
QTRT1	19:10812106-10824113	1111.22	1028.06	894.60	1089.26	1038.19	1184.24	D	D	N
SGSH	17:78180515-78194722	1113.20	1061.32	1050.09	1122.56	1085.91	1140.49	D	D	N
CECR5	22:17618401-17646177	1117.17	1021.01	1040.23	1151.57	1070.65	1163.69	D	D	N
C19orf70	19:5678432-5680907	1117.17	925.26	1021.62	1104.30	1050.68	1183.66	D	D	N
MACROD1	11:63766030-63933578	1131.06	1050.24	1024.90	1111.82	1093.46	1168.66	D	D	N
FBXO31	16:87360593-87425748	1144.95	1017.98	1019.43	1109.67	1096.70	1193.20	D	D	N
ATOX1	5:151121877-151152093	1167.77	1054.27	1077.46	1202.06	1114.69	1220.85	D	D	N
FZR1	19:3506271-3538328	1173.72	1051.24	1017.24	1177.35	1111.65	1235.79	D	D	N
BEST1	11:61717293-61732987	1182.65	952.47	1076.37	1224.62	1091.45	1273.85	D	D	N
ROMO1	20:34287194-34288906	1184.64	967.59	998.62	1203.13	1092.70	1276.57	D	D	N
LYPLA2	1:24117460-24122029	1185.63	1080.47	994.24	1149.42	1122.41	1248.85	D	D	N
PRKRIP1	7:102004319-102067123	1191.58	1062.33	970.15	1123.64	1121.04	1262.13	D	D	N
ARHGEF40	14:21538429-21558399	1193.57	1122.80	1008.48	1173.05	1131.43	1255.70	D	D	N
AAR2	20:34824381-34858840	1208.45	1165.14	1136.59	1190.24	1185.04	1231.86	D	D	N
NIPSNAP1	22:29950797-29977326	1245.16	1189.33	1144.26	1232.13	1210.89	1279.43	D	D	N
STX10	19:13254872-13261197	1265.99	1063.34	1030.38	1197.76	1182.59	1349.40	D	D	N
ZNF787	19:56598732-56632649	1266.99	1150.02	1001.91	1242.88	1176.89	1357.08	D	D	N
NME3	16:1820287-1821731	1273.93	1074.43	1159.59	1289.07	1197.94	1349.92	D	D	N
OBSCN	1:228395831-228566577	1298.73	1100.63	930.74	1188.09	1182.21	1415.26	D	D	N
PLCB3	11:64018995-64036622	1306.67	1167.15	1136.59	1268.66	1246.01	1367.34	D	D	N
ZSCAN18	19:58595205-58629794	1310.64	1207.47	1200.10	1321.30	1261.90	1359.38	D	D	N
BCOR	X:39909068-40036582	1310.64	1223.60	1174.92	1274.03	1266.23	1355.05	D	D	N
FKBP2	11:64008475-64011604	1315.60	1242.75	1131.12	1277.25	1255.99	1375.22	D	D	N
MAP1S	19:17830051-17845325	1323.54	1233.67	1181.49	1336.33	1268.06	1379.02	D	D	N
NENF	1:212606229-212619714	1394.97	1211.50	1217.62	1362.12	1323.23	1466.72	D	D	N
PEX6	6:42931608-42946958	1418.79	1258.87	1126.74	1387.90	1318.60	1518.97	D	D	N
UQCR10	22:30163358-30166402	1429.70	1189.33	1252.66	1352.45	1349.95	1509.45	D	D	N
TTC7A	2:47143296-47303276	1472.36	1364.70	1214.34	1433.01	1387.18	1557.55	D	D	N
DPF2	11:65101225-65120720	1472.36	1341.52	1207.77	1416.90	1386.43	1558.29	D	D	N
STARD3	17:37793318-37819737	1474.35	1404.01	1357.78	1510.36	1422.86	1525.84	D	D	N

MRPL11	11:66202546-66234209	1479.31	1343.54	1370.92	1466.32	1428.41	1530.21	D	D	N
C16orf13	16:684429-686358	1487.24	1279.03	1310.69	1416.90	1415.19	1559.30	D	D	N
FAM83G	17:18872102-18908117	1488.24	1344.54	1208.86	1438.39	1396.11	1580.37	D	D	N
WDR55	5:140044261-140053709	1515.03	1366.72	1265.80	1569.44	1411.07	1618.98	D	D	N
SNRPG	2:70508494-70520903	1515.03	1300.20	1313.98	1488.87	1430.16	1599.89	D	D	N
MOB3A	19:2071037-2096672	1520.98	1394.94	1340.26	1465.24	1461.60	1580.35	D	D	N
PHRF1	11:576486-612222	1527.92	1374.78	1211.05	1456.65	1425.74	1630.11	D	D	N
CSK	15:75074398-75095539	1545.78	1470.53	1318.36	1502.84	1471.58	1619.98	D	D	N
SRC	20:35973088-36034453	1548.76	1446.34	1372.01	1593.07	1473.88	1623.64	D	D	N
DNAJB2	2:220143989-220151622	1559.67	1428.20	1472.75	1571.59	1507.85	1611.50	D	D	N
MOV10	1:113215763-113243368	1572.57	1382.84	1307.41	1617.78	1461.00	1684.14	D	D	N
PACSIN3	11:47199076-47207994	1597.37	1408.04	1341.35	1586.63	1501.03	1693.72	D	D	N
TRMT1	19:13215716-13228381	1601.34	1521.93	1408.15	1602.74	1532.54	1670.14	D	D	N
SDHC	1:161284047-161332984	1632.10	1559.23	1541.74	1636.04	1595.49	1668.71	D	D	N
MRPL20	1:1337288-1342693	1635.08	1533.02	1575.68	1655.38	1593.15	1677.00	D	D	N
SNAI1	20:48599536-48605423	1637.06	1458.44	1281.13	1594.15	1516.85	1757.27	D	D	N
ZMIZ2	7:44788180-44809477	1638.05	1507.82	1458.52	1680.09	1559.35	1716.76	D	D	N
ITPKC	19:41223008-41246765	1688.65	1473.56	1422.38	1657.53	1589.46	1787.84	D	D	N
TBCB	19:36605191-36616849	1703.54	1541.09	1508.89	1651.08	1634.94	1772.13	D	D	N
GPATCH4	1:156564279-156571288	1745.21	1676.14	1619.48	1743.47	1699.95	1790.46	D	D	N
ATPIF1	1:28562620-28573417	1774.97	1562.25	1650.14	1869.15	1673.52	1876.42	D	D	N
CRAT	9:131857089-131873468	1776.96	1636.84	1461.80	1741.32	1670.91	1883.00	D	D	N
ZDHHC8	22:20116979-20135530	1779.93	1646.92	1545.02	1763.88	1697.46	1862.40	D	D	N
ATG4B	2:242576628-242613272	1795.81	1718.48	1560.35	1721.98	1721.43	1870.18	D	D	N
B4GALT2	1:44444615-44456840	1923.79	1799.11	1749.78	1902.45	1861.53	1986.06	D	D	N
IAH1	2:9613787-9636672	1934.71	1848.50	1864.76	1907.82	1905.11	1964.31	D	D	N
GPSM1	9:139221932-139254057	1945.62	1627.76	1402.67	1791.81	1771.27	2119.97	D	D	N
DDX56	7:44605016-44614650	1974.39	1856.56	1727.88	1923.93	1894.34	2054.45	D	D	N
ERBB2	17:37844167-37886679	1979.36	1779.96	1669.85	1888.49	1878.85	2079.87	D	D	N
UPP1	7:48128225-48148330	1982.33	1900.91	1870.23	1945.42	1945.38	2019.29	D	D	N
ZNF358	19:7580178-7585912	1997.21	1877.73	1827.53	2058.21	1917.63	2076.79	D	D	N
SKIV2L	6:31926857-31937532	2060.71	1891.84	1629.33	1969.05	1921.53	2199.90	D	D	N
WDR46	6:33246885-33257304	2068.65	1964.40	1822.05	2126.96	1968.32	2168.98	D	D	N
POLR2C	16:57496299-57505922	2072.62	1997.67	1893.23	2097.96	2003.70	2141.54	D	D	N
COX5A	15:75212132-75230509	2097.42	1769.88	1759.64	2012.02	1969.13	2225.71	D	D	N
ZC3H12A	1:37940153-37949980	2102.38	1913.00	1700.51	2047.47	1968.22	2236.55	D	D	N
SGSM3	22:40766595-40806293	2120.24	1999.68	1880.09	2154.89	2026.54	2213.95	D	D	N
PSMB5	14:23485752-23504439	2145.05	1859.58	1886.66	2048.54	2043.45	2246.65	D	D	N
NDUFB10	16:2009509-2011976	2151.00	1873.69	1914.03	2086.14	2050.93	2251.07	D	D	N
AP1S1	7:100797678-100804877	2152.98	2024.88	1887.75	2115.15	2064.55	2241.42	D	D	N
GSE1	16:85645015-85709810	2152.98	2033.95	1827.53	2137.71	2040.50	2265.46	D	D	N
SNRPD3	22:24951471-25005947	2172.83	2096.44	2020.24	2164.56	2119.59	2226.06	D	D	N
TBRG4	7:45139699-45151646	2174.81	2007.74	1917.31	2251.57	2060.36	2289.27	D	D	N
VPS26B	11:134094539-134117686	2203.58	2076.28	1904.18	2152.74	2105.39	2301.78	D	D	N
SMARCC2	12:56556767-56583351	2222.43	2052.09	1754.16	2116.22	2071.88	2372.99	D	D	N
TYK2	19:10461209-10491352	2239.30	2068.22	1973.16	2213.98	2145.17	2333.43	D	D	N
UQCRQ	5:132202252-132203723	2242.28	1836.40	2060.76	2236.53	2098.82	2385.74	D	D	N
SPG7	16:89557325-89624176	2256.17	2114.58	1906.37	2293.47	2124.65	2387.69	D	D	N
CUEDC2	10:104183002-104192418	2266.09	2139.78	2053.09	2254.80	2190.20	2341.98	D	D	N
TMA7	3:48481667-48485616	2293.87	1927.11	2020.24	2222.57	2165.53	2422.21	D	D	N
LRRC8A	9:131644391-131680318	2303.79	2062.17	2047.62	2221.49	2210.26	2397.32	D	D	N
PIM3	22:50354161-50357728	2308.75	2009.76	1943.59	2275.21	2170.35	2447.16	D	D	N
PELO	5:52083774-52099880	2323.63	2191.18	2100.18	2340.73	2238.04	2409.23	D	D	N
NELFB	9:140149625-140167998	2388.12	2289.96	2166.97	2429.89	2300.41	2475.84	D	D	N
ATP5D	19:1241749-1244824	2416.90	1852.53	1991.77	2291.32	2221.24	2612.55	D	D	N
ZNHIT1	7:100860949-100867471	2431.78	2316.16	2229.38	2471.79	2348.93	2514.63	D	D	N

GUSB	7:65425671-65447301	2498.25	2386.72	2206.39	2519.05	2390.90	2605.60	D	D	N
CPSF1	8:145618444-145634753	2510.16	2290.97	2084.85	2396.59	2374.49	2645.83	D	D	N
CPSF3L	1:1246965-1260071	2517.10	2355.47	2289.61	2601.77	2409.40	2624.81	D	D	N
SPATA20	17:48620419-48633213	2566.71	2307.09	2278.66	2556.65	2449.95	2683.48	D	D	N
TSC22D4	7:100060982-100076902	2581.60	2412.92	2154.93	2481.46	2444.94	2718.25	D	D	N
UBE2S	19:55912652-55919145	2598.46	2359.50	2438.53	2696.30	2484.23	2712.69	D	D	N
PGLS	19:17622438-17632097	2610.37	2365.55	2212.96	2613.59	2463.02	2757.72	D	D	N
UQCRFS1	19:29698173-29704448	2617.31	2448.20	2310.41	2599.62	2509.35	2725.27	D	D	N
ITM2C	2:231729354-231743963	2741.33	2569.15	2320.27	2751.09	2590.14	2892.53	D	D	N
TMEM109	11:60681346-60690915	2763.16	2559.07	2341.07	2664.08	2627.52	2898.80	D	D	N
HSF1	8:145515280-145538385	2765.14	2456.26	2371.73	2670.52	2627.96	2902.32	D	D	N
DDR1	6:30844198-30867933	2766.14	2619.54	2378.30	2762.90	2629.45	2902.82	D	D	N
PTPRS	19:5158506-5340814	2849.48	2490.53	2067.33	2657.63	2599.52	3099.44	D	D	N
FAM127A	X:134166333-134167576	2849.48	2630.63	2263.33	2716.71	2661.11	3037.85	D	D	N
MTA2	11:62360686-62369312	2976.47	2754.60	2519.56	2936.93	2820.01	3132.94	D	D	N
MRPL37	1:54649714-54691137	2995.33	2824.15	2665.19	3065.83	2860.56	3130.09	D	D	N
ARAP1	11:72396114-72504644	3021.12	2756.62	2545.84	2903.63	2867.46	3174.78	D	D	N
SAT2	17:7529552-7531194	3081.64	2969.28	2763.74	3043.28	2975.35	3187.94	D	D	N
TRMT112	11:64083932-64085556	3089.58	2747.55	2689.28	3015.35	2942.07	3237.09	D	D	N
BCAR1	16:75262928-75301951	3092.56	2826.16	2434.15	2913.30	2884.05	3301.06	D	D	N
SAE1	19:47616531-47713886	3153.08	2967.27	2972.88	3131.36	3078.22	3227.94	D	D	N
YIF1A	11:66052051-66056641	3198.72	3018.67	2879.80	3260.27	3068.76	3328.68	D	D	N
MLLT6	17:36861795-36886056	3236.42	3015.65	2656.43	3124.92	3047.92	3424.92	D	D	N
CDIPT	16:29869678-29875057	3251.30	3040.85	3017.77	3281.75	3147.92	3354.68	D	D	N
EMP3	19:48824766-48833810	3429.89	3074.11	2837.10	3318.28	3231.72	3628.06	D	D	N
MAPK3	16:30125426-30134827	3481.48	3309.96	3082.38	3582.54	3317.28	3645.68	D	D	N
TNRC18	7:5346421-5465045	3721.58	3307.94	2626.86	3439.66	3373.09	4070.08	D	D	N
SEZ6L2	16:29882480-29910868	3775.16	3536.73	3203.92	3813.49	3564.85	3985.47	D	D	N
RANGAP1	22:41641615-41682255	3844.61	3605.27	3396.64	3712.52	3702.60	3986.62	D	D	N
AHCY	20:32868074-32899608	3938.87	3582.09	3744.84	3833.90	3825.58	4052.15	D	D	N
SMARCA4	19:11071598-11176071	3957.72	3734.28	3425.11	3880.10	3781.36	4134.07	D	D	N
TRAF7	16:2205699-2228130	3996.41	3548.83	3256.48	3862.91	3747.90	4244.93	D	D	N
SPHK1	17:74372665-74383941	4000.38	3594.19	3431.68	3951.00	3793.36	4207.40	D	D	N
PPP4C	16:30087299-30096698	4046.02	3750.41	3628.77	4072.38	3881.50	4210.54	D	D	N
PRDX2	19:12907634-12912694	4060.90	3751.42	3811.64	4039.08	3943.01	4178.79	D	D	N
GCN1L1	12:120565007-120632513	4063.88	3749.40	3424.01	4048.75	3837.39	4290.37	D	D	N
VARS	6:31745295-31763730	4079.75	3771.58	3555.41	3986.44	3904.22	4255.29	D	D	N
BIN1	2:127805603-127864931	4109.52	3783.67	3490.81	3986.44	3906.80	4312.24	D	D	N
C19orf43	19:12841454-12845589	4351.61	3981.22	3617.82	4187.32	4114.29	4588.92	D	D	N
C12orf57	12:7052141-7055166	4363.51	3730.25	3867.48	4215.25	4142.29	4584.73	D	D	N
ATP5E	20:57600522-57607437	4443.88	3417.80	3940.84	4347.38	4093.55	4794.20	D	D	N
PVR	19:45147098-45166850	4733.59	4475.09	4043.77	4549.34	4514.52	4952.65	D	D	N
PSMB7	9:127115745-127177723	4739.54	4331.97	4302.19	4583.71	4582.64	4896.44	D	D	N
ATN1	12:7033626-7051484	4811.97	4375.31	3810.54	4605.20	4487.96	5135.97	D	D	N
TMED3	15:79603404-79704334	4862.57	4478.12	4281.38	4711.55	4670.54	5054.59	D	D	N
ERP29	12:112451120-112461255	5339.79	4838.95	4803.69	5346.41	5113.59	5566.00	D	D	N
MGAT4B	5:179224597-179233952	5691.02	5265.29	4883.62	5704.13	5396.95	5985.08	D	D	N
TCEB2	16:2821415-2827298	5783.29	5098.99	5180.36	5575.22	5539.84	6026.74	D	D	N
PVRL2	19:45349432-45392485	5850.76	5487.03	4926.33	5744.95	5540.83	6160.68	D	D	N
TMED9	5:177019159-177023125	5885.48	5628.14	5433.30	6086.55	5670.50	6100.46	D	D	N
ATXN2L	16:28834356-28848558	6163.29	5736.99	4926.33	5858.82	5767.25	6559.32	D	D	N
LY6E	8:144099399-144105249	6383.54	5891.20	5630.40	6270.24	6123.42	6643.67	D	D	N
PTMS	12:6874682-6880116	6608.76	5931.51	6242.50	6499.05	6382.78	6834.75	D	D	N
TUBB4B	9:140135665-140138159	6805.21	6109.91	6392.51	6855.70	6539.44	7070.99	D	D	N
LENG8	19:54960065-54973217	6936.18	6244.97	5250.44	6557.06	6394.61	7477.74	D	D	N
CCT3	1:156278759-156337664	7528.50	7240.78	6914.82	7365.95	7333.49	7723.50	D	D	N

LTBP3	11:65306276-65326401	7831.10	7347.62	6688.15	7951.40	7400.82	8261.39	D	D	N
PER1	17:8043790-8059824	8549.43	7564.32	6607.13	8493.89	7861.83	9237.02	D	D	N
PLEC	8:144989321-145050902	9353.07	8537.95	6909.34	8707.66	8573.41	10132.74	D	D	N
EIF5A	17:7210318-7215774	12878.21	11725.95	11648.43	12783.26	12381.62	13374.80	D	D	N
ARF1	1:228270361-228286912	17197.08	15725.32	15193.98	16593.54	16528.44	17865.71	D	D	N
ZFP36	19:39897453-39900052	20719.24	19575.51	19151.25	21383.50	19949.51	21488.97	D	D	N
IFITM3	11:319669-327537	25984.62	23516.41	22112.08	25506.37	24636.52	27332.72	D	D	N
RPLP2	11:809647-812880	26742.63	21110.55	21244.86	25131.47	24623.15	28862.11	D	D	N
IGFBP4	17:38599702-38613983	32889.05	30288.52	27415.08	31889.41	31096.28	34681.81	D	D	N
ALDOA	16:30064411-30081778	37051.15	35442.94	34378.08	37162.77	36045.14	38057.16	D	D	N
RPLP1	15:69745123-69748255	43241.22	35826.95	35678.92	40498.24	40462.24	46020.21	D	D	N
JUNB	19:12902310-12904124	51674.57	48263.48	45841.46	54221.45	48904.17	54444.96	D	D	N
RPS19	19:42363988-42376994	54604.41	50423.42	50955.02	56595.48	52386.36	56822.46	D	D	N
RPL13	16:89627065-89630950	67961.83	58428.19	56099.25	65552.36	63724.97	72198.70	D	D	N
MYLK3	16:46740891-46824319	0.99	2.02	2.19	0.00	0.23	1.75	U	U	D
ABCD2	12:39943835-40013553	0.99	2.02	2.19	0.00	0.23	1.75	U	U	D
PLD5	1:242246288-242687998	0.99	2.02	2.19	0.00	0.23	1.75	U	U	D
COL6A6	3:130279178-130396999	0.99	2.02	2.19	0.00	0.23	1.75	U	U	D
RP11-507M3.1	2:24347236-24413305	0.99	2.02	2.19	0.00	0.23	1.75	U	U	D
KRBOX1	3:42850938-43097363	0.99	2.02	2.19	0.00	0.23	1.75	U	U	D
PKD2L2	5:137223657-137278436	1.98	3.02	3.28	1.07	1.22	2.75	U	U	D
DMRT1	9:841690-969090	1.98	4.03	5.47	0.00	0.19	3.78	U	U	D
VPREB3	22:24094930-24096655	2.98	7.06	5.47	0.00	0.66	5.29	U	U	D
PCDHA4	5:140186659-140391929	3.97	6.05	5.47	2.15	2.66	5.28	U	U	D
NEK5	13:52611093-52703214	5.95	9.07	10.95	3.22	3.39	8.51	U	U	D
TTC4	1:55181495-55208330	5.95	8.06	8.76	4.30	4.43	7.48	U	U	D
SEMA3G	3:52467069-52479101	6.95	11.09	9.85	4.30	4.66	9.23	U	U	D
HIST2H2AC	1:149858525-149858961	6.95	10.08	9.85	3.22	4.54	9.35	U	U	D
TIMM23B	10:51371390-51387768	8.93	11.09	12.04	6.45	7.06	10.80	U	U	D
FGL2	7:76822688-76829143	16.87	26.21	21.90	11.82	12.20	21.54	U	U	D
USP51	X:55511049-55515635	19.84	29.23	29.56	10.74	13.13	26.56	U	U	D
DEPDC4	12:100597447-100660857	21.83	28.22	25.18	18.26	18.61	25.05	U	U	D
PRSS36	16:31150246-31161415	21.83	33.26	28.47	16.11	16.19	27.46	U	U	D
SLC51A	3:195938358-195970049	33.73	38.30	39.42	30.08	30.50	36.96	U	U	D
TF	3:133464800-133497850	40.68	48.38	44.89	35.45	36.50	44.86	U	U	D
EML6	2:54950636-55199157	46.63	52.41	54.75	40.82	41.95	51.31	U	U	D
ESYT3	3:138153428-138200528	67.47	85.67	78.84	51.56	56.28	78.65	U	U	D
TPK1	7:144149034-144533488	93.26	101.80	106.21	80.57	84.79	101.73	U	U	D
ZNF433	19:12125547-12146556	101.20	103.81	104.02	98.83	99.36	103.04	U	U	D
ERAP2	5:96211643-96255420	131.96	154.21	168.63	107.42	111.90	152.02	U	U	D
ZNF322	6:26636518-26659980	144.86	168.32	164.25	123.54	129.45	160.26	U	U	D
SP4	7:21467652-21554440	190.49	213.68	206.95	170.80	176.16	204.83	U	U	D
HDX	X:83572882-83757487	228.20	273.14	255.13	196.58	203.17	253.22	U	U	D
DNAJC12	10:69556427-69597924	251.02	258.02	259.51	239.55	244.19	257.84	U	U	D
DPH6	15:35509546-35838394	292.69	336.64	342.73	249.22	260.03	325.34	U	U	D
WDSUB1	2:160092304-160143310	446.47	498.91	486.17	412.50	416.99	475.95	U	U	D
CSRP2	12:77252495-77272840	447.46	458.60	458.80	429.69	437.19	457.74	U	U	D
CBWD2	2:114195268-114253766	512.95	572.49	552.97	481.25	482.33	543.56	U	U	D
ZNF260	19:37001597-37019562	569.50	627.92	614.29	526.37	534.92	604.08	U	U	D
ZNF140	12:133656424-133684130	587.36	616.84	619.76	555.37	564.78	609.93	U	U	D
NSL1	1:212899495-212965124	672.68	719.64	732.54	615.53	632.91	712.46	U	U	D
UTP14C	13:52598827-52607736	789.76	832.53	826.71	743.36	758.93	820.59	U	U	D
INADL	1:62208149-62629592	837.38	862.77	852.99	823.93	824.54	850.22	U	U	D
NTPCR	1:233086351-233119628	887.98	944.41	915.41	860.45	860.94	915.03	U	U	D
ISOC1	5:128430444-128449721	961.40	1017.98	1026.00	898.05	916.92	1005.88	U	U	D
RWDD2B	21:30376705-30391699	986.21	1060.31	1042.42	933.50	943.07	1029.34	U	U	D
GCH1	14:55308726-55369570	1414.82	1515.89	1550.50	1283.70	1325.00	1504.64	U	U	D
GGPS1	1:235490665-235507847	1465.42	1633.81	1645.76	1336.33	1354.61	1576.22	U	U	D
PDCD10	3:167401086-167452727	1819.62	1935.18	1934.83	1746.69	1750.18	1889.06	U	U	D
GALNT1	18:33161081-33291798	2157.94	2368.57	2310.41	1916.42	2006.54	2309.35	U	U	D

SCOC	4:141178440-141306880	3206.65	3808.87	3604.68	2800.50	2871.86	3541.45	U	U	D
SLMO2	20:57608200-57617964	5693.00	6507.03	6153.80	5089.67	5233.47	6152.54	U	U	D
MMADHC	2:150426148-150444330	5843.81	6266.14	6199.79	5238.99	5491.13	6196.49	U	U	D
H2AFV	7:44866390-44887682	2317.68	2430.06	2450.57	2269.83	2252.25	2383.11	U	U	N
TRDMT1	10:17184253-17244053	234.15	272.13	259.51	220.22	216.46	251.84	U	U	N
PAK1IP1	6:10694928-10710015	1126.10	1240.73	1189.15	1074.22	1071.58	1180.61	U	U	N
BRIX1	5:34915481-34926101	2122.23	2400.83	2346.55	1973.35	1973.00	2271.45	U	U	N
COQ10B	2:198318147-198340032	2411.94	2616.52	2539.27	2339.66	2318.62	2505.25	U	U	N
COX7A2L	2:42560686-42652228	7670.37	8064.24	7948.48	7533.53	7486.86	7853.89	U	U	N
OLFM3	1:102268130-102462586	0.99	3.02	2.19	0.00	0.00	1.99	U	U	N
NMI	2:152126979-152146571	984.22	1139.94	1081.84	936.72	915.15	1053.29	U	U	N
DTD2	14:31915242-31926716	860.20	1063.34	992.05	786.33	766.27	954.14	U	U	N
ACTR10	14:58666798-58701750	2586.56	2927.96	2954.26	2462.12	2401.93	2771.18	U	U	N
CRP	1:159682079-159684379	7.94	11.09	9.85	6.45	6.40	9.48	U	U	N
MED10	5:6371994-6378707	2041.86	2116.60	2194.35	1989.46	1974.86	2108.86	U	U	N
KIAA1468	18:59854491-59974355	842.34	895.02	879.27	818.56	816.29	868.40	U	U	N
CYP19A1	15:51500254-51630807	26.79	32.25	32.85	23.63	23.46	30.12	U	U	N
LINS	15:101099574-101143435	238.12	280.20	272.65	216.99	215.87	260.37	U	U	N
FAM63A	1:150969025-150980851	132.95	147.15	144.54	127.83	126.03	139.87	U	U	N
SKP2	5:36152091-36184421	627.04	655.14	678.89	604.79	602.78	651.31	U	U	N
INIP	9:115446206-115480516	756.02	866.80	821.24	720.80	707.02	805.03	U	U	N
C21orf91	21:19161284-19191703	629.03	768.02	747.87	581.16	560.96	697.10	U	U	N
MTERFD1	8:97251626-97273838	625.06	682.35	658.08	606.94	599.82	650.30	U	U	N
ZNF474	5:121465208-121515312	3.97	9.07	6.57	2.15	1.70	6.24	U	U	N
BAALC	8:104152938-104242533	25.80	31.25	31.75	22.56	22.47	29.12	U	U	N
PRDX3	10:120927215-120938345	5487.63	5552.54	5540.61	5454.91	5453.32	5521.94	U	U	N
TMEM41B	11:9302201-9336327	2473.45	2882.61	2675.04	2350.40	2298.24	2648.66	U	U	N
ZCCHC4	4:25314407-25372005	454.41	487.83	479.60	438.28	437.30	471.51	U	U	N
UBE2V2	8:48920960-48977268	3334.64	3980.21	3754.70	3131.36	3044.62	3624.67	U	U	N
CCDC126	7:23636998-23684327	219.27	243.91	236.52	211.62	208.06	230.48	U	U	N
NAT1	8:18027986-18081198	185.53	210.65	212.43	174.02	171.33	199.74	U	U	N
ZDHHC21	9:14611069-14693469	798.69	948.44	911.03	751.96	729.36	868.02	U	U	N
TBL1XR1	3:176737143-176915261	7895.59	9419.87	8579.19	7342.32	7223.04	8568.15	U	U	N
NEUROG2	4:113434672-113437328	0.99	3.02	3.28	0.00	-0.20	2.19	U	U	N
CEP57L1	6:109416313-109485135	603.23	687.39	670.13	562.89	559.67	646.79	U	U	N
FAM162B	6:117073363-117086886	1.98	4.03	3.28	1.07	0.99	2.97	U	U	N
ZFP90	16:68563993-68609975	1271.95	1450.37	1366.54	1209.58	1192.60	1351.30	U	U	N
ZNF501	3:44771088-44778575	127.00	163.28	159.87	108.50	107.16	146.83	U	U	N
ZNF490	19:12688775-12750912	16.87	23.18	25.18	13.96	12.92	20.81	U	U	N
ZNF383	19:37708828-37734828	190.49	244.92	217.90	172.95	166.83	214.16	U	U	N
SMIM15	5:60453536-60458301	2850.47	3255.53	3121.80	2729.60	2669.25	3031.69	U	U	N
GPN1	2:27851114-27874375	1277.90	1344.54	1336.97	1246.10	1242.36	1313.44	U	U	N
AC073343.1	7:6713376-6715991	80.36	108.85	112.78	67.68	63.92	96.81	U	U	N
AGAP7	10:51464162-51486327	4.96	6.05	6.57	4.30	4.19	5.73	U	U	N
AC018867.1	2:187361840-187365393	2.98	9.07	6.57	1.07	0.29	5.66	U	U	N
RP11-156E8.1	1:245132652-245134390	25.80	42.33	38.32	20.41	18.06	33.53	U	U	N
CALCR	7:93053799-93204042	0.99	4.03	7.66	1.07	-1.37	3.36	U	U	N
HCCS	X:11129421-11141198	1050.70	1225.61	1147.54	1083.89	992.88	1108.51	U	U	N
MEOX1	17:41717756-41739322	0.00	1.01	1.09	0.00	-0.46	0.46	U	U	N
MPO	17:56347217-56358296	0.99	6.05	4.38	1.07	-0.89	2.87	U	U	N
GCFC2	2:75879126-75938115	1117.17	1320.35	1237.33	1110.75	1041.36	1192.98	U	U	N
LAMP2	X:119561682-119603220	8662.53	10531.59	9625.99	8782.85	8012.11	9312.96	U	U	N
RALA	7:39663082-39747723	5240.58	6560.45	6134.09	5573.07	4800.99	5680.17	U	U	N
AGK	7:141250989-141355044	1138.01	1183.28	1190.25	1144.05	1118.00	1158.01	U	U	N
USH1C	11:17515442-17565963	11.91	23.18	20.80	15.04	8.02	15.79	U	U	N
VPS41	7:38762563-38971994	2099.41	2560.08	2269.90	2176.38	1948.26	2250.56	U	U	N
CDKL3	5:133541305-133706738	20.84	36.28	27.37	18.26	14.81	26.86	U	U	N

SPRTN	1:231472850-231490769	529.81	586.60	583.63	539.26	507.70	551.92	U	U	N
AGPS	2:178257372-178408564	2096.43	2682.03	2360.78	2123.74	1892.79	2300.07	U	U	N
VSIG2	11:124617368-124622134	11.91	22.17	25.18	12.89	6.93	16.89	U	U	N
RUNX3	1:25226002-25291612	0.99	4.03	4.38	1.07	-0.39	2.37	U	U	N
RB1CC1	8:53535016-53658403	4995.52	6406.24	5724.57	5198.17	4522.96	5468.07	U	U	N
ERP44	9:102741461-102861322	2486.35	3035.81	2830.53	2650.11	2309.05	2663.65	U	U	N
SNAPC1	14:62229075-62263146	1733.30	2092.41	1958.92	1762.80	1606.01	1860.59	U	U	N
TBPL1	6:134273308-134311570	832.42	994.80	932.93	815.34	768.70	896.14	U	U	N
ASTE1	3:130732719-130746493	177.60	203.60	231.04	189.06	160.29	194.90	U	U	N
BARX2	11:129245835-129322171	0.99	6.05	13.14	3.22	-2.97	4.95	U	U	N
PER3	1:7844380-7905237	495.09	638.00	593.48	532.82	447.53	542.65	U	U	N
KIAA2022	X:73952684-74145282	2.98	6.05	4.38	2.15	1.69	4.26	U	U	N
GINM1	6:149887430-149912884	3491.40	4333.99	3970.41	3710.37	3219.60	3763.21	U	U	N
C4orf27	4:170650616-170679104	850.28	1140.95	1048.99	894.83	749.02	951.54	U	U	N
BCAT1	12:24964295-25102393	17113.73	23251.34	20193.67	18709.76	15154.61	19072.86	U	U	N
THUMPD1	16:20744986-20753406	2155.96	2580.23	2517.37	2143.08	1981.95	2329.97	U	U	N
IDI1	10:1085848-1095110	3031.04	3524.64	3321.08	3078.73	2859.33	3202.76	U	U	N
VPS35	16:46690054-46723430	5557.08	6377.01	6054.16	5646.12	5272.12	5842.03	U	U	N
SCT	11:626431-627143	0.00	4.03	2.19	1.07	-1.29	1.29	U	U	N
POLB	8:42195972-42229326	744.12	880.91	852.99	778.81	696.45	791.79	U	U	N
ASNS	7:97481430-97501854	10848.26	12374.04	12206.87	11306.21	10302.12	11394.39	U	U	N
ASAH2C	10:48001603-48055018	0.00	4.03	3.28	0.00	-1.60	1.60	U	U	N
FCGR2B	1:161551101-161648444	0.00	1.01	1.09	0.00	-0.46	0.46	U	U	N
DERL2	17:5374571-5390131	1505.10	1727.55	1599.77	1507.14	1426.46	1583.75	U	U	N
SNCB	5:176047085-176057530	0.99	4.03	7.66	2.15	-1.20	3.18	U	U	N
DNAJC10	2:183580999-183659191	5906.32	7350.64	6511.86	6023.17	5413.99	6398.65	U	U	N
UBE2A	X:118708501-118718381	2690.73	3198.08	2940.03	2811.24	2527.72	2853.74	U	U	N
ACER3	11:76571911-76737841	1649.96	2248.63	2072.80	1838.00	1453.04	1846.88	U	U	N
ADCY2	5:7396321-7830194	0.00	3.02	3.28	1.07	-1.18	1.18	U	U	N
BPIFB2	20:31595406-31611515	0.00	1.01	2.19	0.00	-0.78	0.78	U	U	N
STX7	6:132767006-132834337	2396.06	2827.17	2656.43	2487.90	2253.31	2538.81	U	U	N
DDX43	6:74104471-74127292	50.60	60.47	54.75	49.41	46.85	54.35	U	U	N
RAB21	12:72148654-72184699	2695.69	3231.34	3025.44	2709.19	2500.73	2890.65	U	U	N
FAM135A	6:71122644-71270877	505.01	665.22	602.24	522.07	449.26	560.75	U	U	N
TRAF5	1:211499957-211548288	1245.16	1594.50	1410.34	1286.92	1127.60	1362.72	U	U	N
EPYC	12:91357456-91398803	0.00	1.01	1.09	0.00	-0.46	0.46	U	U	N
CHMP2B	3:87276421-87304698	2299.82	2901.76	2729.79	2397.67	2088.71	2510.93	U	U	N
RAB10	2:26256976-26360323	7423.33	8890.72	8370.05	7783.83	6938.26	7908.39	U	U	N
HBQ1	16:230452-231180	13.89	16.13	19.71	13.96	11.84	15.94	U	U	N
ZW10	11:113603909-113644533	605.22	698.48	640.56	612.31	573.42	637.02	U	U	N
F11	4:187187099-187210835	0.00	2.02	1.09	0.00	-0.73	0.73	U	U	N
RBBP9	20:18467184-18477887	680.62	850.67	826.71	718.66	618.86	742.39	U	U	N
C20orf26	20:20033158-20341346	0.00	3.02	3.28	1.07	-1.18	1.18	U	U	N
LHX5	12:113899839-113910085	0.00	1.01	1.09	0.00	-0.46	0.46	U	U	N
CMTM6	3:32522804-32544900	3067.75	3527.66	3325.46	2998.16	2885.17	3250.33	U	U	N
RPGRIP1	14:21756098-21819460	1.98	4.03	6.57	2.15	0.38	3.59	U	U	N
TGFB2	1:218519577-218617961	1909.90	2721.34	2341.07	2134.48	1651.32	2168.49	U	U	N
ECHDC1	6:127609855-127664754	2827.65	3653.65	3169.98	2925.11	2550.93	3104.38	U	U	N
CBX5	12:54624724-54673886	4371.45	4967.96	4700.76	4422.58	4164.56	4578.33	U	U	N
IFT74	9:26947037-27062928	700.46	879.90	826.71	717.58	635.69	765.24	U	U	N
BRMS1L	14:36295524-36401531	468.30	542.25	500.41	451.17	438.25	498.35	U	U	N
SIRPB1	20:1544167-1600707	1.98	6.05	6.57	3.22	0.33	3.64	U	U	N
MAPRE1	20:31407699-31438211	4466.70	5325.76	4978.89	4642.80	4181.84	4751.55	U	U	N
PXMP4	20:32294512-32308125	108.15	133.04	142.35	111.72	95.74	120.55	U	U	N
ASIP	20:32782375-32857150	0.99	3.02	4.38	1.07	-0.24	2.22	U	U	N
VAPA	18:9913999-9960018	12914.92	15934.96	14883.01	13692.06	11919.14	13910.70	U	U	N

RIOK3	18:21032787-21066567	3045.93	3711.10	3572.93	3123.84	2799.84	3292.01	U	U	N
TAF7L	X:100523241-100548059	23.81	30.24	27.37	24.71	21.64	25.98	U	U	N
DNAJC3	13:96329393-96447243	3530.10	4042.70	3752.51	3469.74	3335.44	3724.75	U	U	N
ARL2BP	16:57279010-57287516	2584.57	2899.74	2710.08	2571.69	2470.42	2698.72	U	U	N
PRSS33	16:2833954-2837949	0.00	4.03	2.19	0.00	-1.46	1.46	U	U	N
IQCH	15:67547138-67794598	117.07	144.13	142.35	125.68	107.23	126.92	U	U	N
GABPB1	15:50569389-50647605	1109.23	1352.61	1293.17	1123.64	1017.90	1200.57	U	U	N
EIF3J	15:44829255-44855227	4527.22	5412.44	5160.65	4650.31	4213.12	4841.32	U	U	N
MYEF2	15:48431625-48470714	407.78	549.31	498.22	446.88	361.61	453.95	U	U	N
ZDHHC2	8:17013538-17082308	1377.12	1598.54	1477.13	1372.86	1297.39	1456.84	U	U	N
PPP2CB	8:30631973-30671830	6105.74	6672.32	6378.27	5947.98	5866.95	6344.53	U	U	N
CCDC114	19:48799714-48825151	2.98	10.08	12.04	5.37	-0.15	6.11	U	U	N
CABP5	19:48533210-48547310	0.00	1.01	1.09	0.00	-0.46	0.46	U	U	N
TWISTNB	7:19735085-19748710	2010.11	2177.07	2251.28	1970.13	1909.72	2110.51	U	U	N
DUS4L	7:107203929-107218906	324.44	363.85	385.43	319.04	300.54	348.33	U	U	N
STEAP1B	7:22459063-22672544	91.28	109.86	104.02	94.53	84.86	97.69	U	U	N
FAM188B	7:30811033-30932002	7.94	11.09	10.95	8.59	6.73	9.15	U	U	N
MEOX2	7:15650837-15726437	28.77	51.40	41.61	35.45	21.56	35.99	U	U	N
RHEB	7:151163098-151217206	5176.09	5901.28	5594.27	4983.32	4866.24	5485.93	U	U	N
RAB11FIP2	10:119764427-119806114	1685.68	2176.06	1906.37	1727.35	1518.38	1852.97	U	U	N
ATE1	10:123499939-123688316	1135.03	1387.88	1224.19	1199.91	1054.36	1215.69	U	U	N
TMEM33	4:41937137-41962589	3360.44	4325.92	4062.39	3555.68	3026.26	3694.62	U	U	N
DCUN1D4	4:52709166-52783003	3328.69	3995.33	3588.26	3257.05	3078.31	3579.07	U	U	N
LAMTOR3	4:100799493-100815647	1538.84	1725.53	1616.19	1543.66	1473.47	1604.20	U	U	N
TRIM2	4:154073494-154260472	1779.93	2216.38	1949.07	1805.77	1629.88	1929.99	U	U	N
CRYAB	11:111779289-111794446	622.08	675.30	649.32	638.09	605.29	638.87	U	U	N
KIAA1377	11:101785746-101871789	136.92	213.68	189.43	143.95	109.35	164.49	U	U	N
SLC11A2	12:51373184-51422349	2314.70	2622.57	2416.63	2407.34	2217.26	2412.15	U	U	N
MAGOHB	12:10758612-10766222	466.31	523.10	534.35	480.18	441.68	490.95	U	U	N
MYL2	12:111348623-111358526	2.98	5.04	6.57	2.15	1.47	4.48	U	U	N
RERGL	12:18233803-18473041	0.00	4.03	2.19	0.00	-1.46	1.46	U	U	N
TMEM14C	6:10723148-10731362	4727.63	5040.53	4883.62	4675.02	4603.88	4851.39	U	U	N
ADTRP	6:11712287-11807279	40.68	63.50	53.65	38.67	31.94	49.42	U	U	N
SOD2	6:160090089-160183561	47921.23	55038.61	52832.92	47601.01	45161.82	50680.65	U	U	N
RBM24	6:17281577-17294106	25.80	42.33	36.13	26.86	19.88	31.71	U	U	N
CAP2	6:17393447-17558023	296.66	359.82	321.92	287.89	272.47	320.84	U	U	N
CLIC5	6:45868045-46048132	0.00	1.01	1.09	0.00	-0.46	0.46	U	U	N
SEMA5A	5:9035138-9546187	211.33	253.99	233.23	211.62	196.03	226.63	U	U	N
MRPS30	5:44809027-44820530	1741.24	2086.36	1860.38	1775.69	1624.80	1857.68	U	U	N
CNOT6	5:179921412-180005405	1103.28	1219.56	1262.52	1091.41	1039.58	1166.98	U	U	N
PPP2CA	5:133530025-133561833	5641.41	6373.98	6082.63	5616.04	5366.78	5916.05	U	U	N
SMAD5	5:135468534-135524435	3660.07	4471.06	3945.22	3670.62	3375.16	3944.98	U	U	N
LRRC31	3:169556967-169587718	0.00	1.01	2.19	0.00	-0.78	0.78	U	U	N
EIF1B	3:40351175-40353915	5021.31	5635.19	5321.62	5206.76	4828.09	5214.54	U	U	N
MOB1A	2:74379655-74406025	7398.52	8926.00	8039.36	7351.99	6847.30	7949.75	U	U	N
OTX1	2:63277192-63284971	16.87	20.16	21.90	18.26	15.22	18.52	U	U	N
TXNDC9	2:99935445-99957165	989.18	1116.76	1117.98	1008.70	937.55	1040.81	U	U	N
CD207	2:71057347-71062952	0.00	3.02	2.19	0.00	-1.16	1.16	U	U	N
ALMS1	2:73612886-73837920	370.07	461.62	406.24	394.24	341.01	399.14	U	U	N
BCAS2	1:115110178-115124260	1519.99	1786.01	1670.94	1474.91	1413.03	1626.94	U	U	N
CD58	1:117057157-117113661	287.73	335.63	318.64	281.45	268.48	306.97	U	U	N
RPF1	1:84944942-84963473	1347.35	1574.35	1497.94	1408.31	1272.55	1422.15	U	U	N
CTBS	1:85015289-85040163	2374.23	3067.05	2861.19	2520.13	2137.44	2611.03	U	U	N
CR2	1:207627575-207663240	5.95	7.06	7.66	6.45	5.40	6.51	U	U	N
RCN2	15:77223960-77242601	4608.57	5383.21	5269.06	4841.53	4336.45	4880.70	U	U	N
CREB1	2:208394461-208468155	1401.92	1795.08	1557.07	1367.49	1255.84	1548.00	U	U	N

TRIM67	1:231297858-231357302	0.00	2.02	1.09	0.00	-0.73	0.73	U	U	N
AFTPH	2:64751465-64820139	1413.83	1590.47	1595.39	1443.76	1342.15	1485.50	U	U	N
CAAP1	9:26840683-26892802	778.84	919.21	881.46	754.11	719.26	838.43	U	U	N
PCDHB12	5:140588269-140591696	0.99	4.03	8.76	3.22	-1.46	3.44	U	U	N
GORAB	1:170501270-170522587	719.31	985.73	830.00	777.74	633.56	805.07	U	U	N
TP53AIP1	11:128804626-128813040	3.97	10.08	6.57	3.22	1.64	6.29	U	U	N
SPG20OS	13:36920568-36943738	20.84	36.28	45.99	26.86	12.57	29.10	U	U	N
ADRA1A	8:26605667-26724790	2.98	8.06	12.04	4.30	-0.08	6.04	U	U	N
PAPD5	16:50186829-50269221	2232.36	2721.34	2484.52	2195.71	2048.67	2416.04	U	U	N
GTDC1	2:144695635-145090135	662.76	832.53	753.35	691.80	606.41	719.11	U	U	N
GTF3A	13:27998681-28009958	2619.30	2870.51	2706.80	2652.26	2535.65	2702.94	U	U	N
43715.00	7:35840542-35944917	10751.02	12788.29	11496.22	10913.04	10057.59	11444.46	U	U	N
NT5C3A	7:33053742-33102409	1780.92	1987.59	2008.20	1774.62	1685.38	1876.47	U	U	N
SPINK4	9:33218363-33248565	0.00	1.01	1.09	0.00	-0.46	0.46	U	U	N
OR2S2	9:35957105-35958151	0.00	2.02	1.09	0.00	-0.73	0.73	U	U	N
P4HA1	10:74766975-74856732	8866.92	10661.61	9923.83	9393.01	8292.05	9441.79	U	U	N
RNF11	1:51701943-51739127	4886.38	5955.70	5357.75	5026.29	4529.54	5243.22	U	U	N
SPRYD7	13:50486842-50510626	1055.66	1146.99	1127.83	1033.40	1014.44	1096.87	U	U	N
RAP2C	X:131337053-131353471	1954.55	2380.67	2215.15	1933.60	1793.05	2116.06	U	U	N
PLA2G12A	4:110631145-110651233	1449.54	1673.12	1643.57	1526.47	1371.59	1527.50	U	U	N
SLPI	20:43880880-43883205	0.99	3.02	5.47	2.15	-0.43	2.42	U	U	N
TP53TG5	20:44002526-44036529	0.00	4.03	4.38	1.07	-1.63	1.63	U	U	N
GLO1	6:38643701-38670917	8723.05	9573.07	9451.89	8475.63	8318.75	9127.35	U	U	N
IL37	2:113670548-113676459	0.00	1.01	1.09	0.00	-0.46	0.46	U	U	N
NAPB	20:23355159-23402125	522.87	621.88	578.15	537.11	489.44	556.30	U	U	N
LAMP5	20:9495005-9511171	0.00	1.01	1.09	0.00	-0.46	0.46	U	U	N
SGPP1	14:64150932-64194757	1619.20	2004.72	1883.37	1681.16	1485.29	1753.11	U	U	N
RHOT1	17:30469473-30580393	1801.76	2153.89	1939.21	1875.59	1688.03	1915.49	U	U	N
EVI2A	17:29644578-29648902	1.98	6.05	5.47	1.07	0.12	3.85	U	U	N
OMG	17:29599031-29624557	10.91	15.12	17.52	9.67	8.18	13.65	U	U	N
PPCS	1:42921788-42939056	1439.62	1587.45	1523.12	1477.06	1391.86	1487.39	U	U	N
ZNF780B	19:40534167-40562116	218.27	244.92	247.47	219.14	206.34	230.20	U	U	N
DNAJB9	7:108210012-108215294	2675.85	3171.87	3107.56	2738.20	2486.49	2865.21	U	U	N
ORMDL1	2:190635049-190649097	2793.92	3503.47	3241.15	2993.86	2563.45	3024.39	U	U	N
ANAPC13	3:134196548-134205558	2185.72	2419.98	2435.24	2203.23	2084.53	2286.92	U	U	N
AIPL1	17:6297013-6338519	0.00	4.03	3.28	0.00	-1.60	1.60	U	U	N
PHF20L1	8:133787618-133861052	2188.70	2558.06	2325.74	2193.57	2058.88	2318.52	U	U	N
PARP2	14:20811741-20826064	786.78	875.87	848.61	807.82	756.75	816.81	U	U	N
REEP5	5:112212084-112258236	5299.12	6156.28	5774.94	5551.59	5026.47	5571.76	U	U	N
PRRG3	X:150863596-150874396	0.00	1.01	1.09	0.00	-0.46	0.46	U	U	N
TULP4	6:158733692-158932860	999.10	1225.61	1082.94	1030.18	923.91	1074.30	U	U	N
RTN4IP1	6:107018903-107077373	216.29	242.90	225.57	223.44	207.82	224.76	U	U	N
MED18	1:28655513-28662476	459.37	492.87	501.50	456.55	442.16	476.58	U	U	N
GFAP	17:42982376-42994305	3.97	5.04	5.47	4.30	3.45	4.48	U	U	N
CCT6B	17:33254878-33308097	91.28	127.00	124.83	92.38	76.50	106.05	U	U	N
TIMM10B	11:6502677-6505909	640.93	749.88	694.22	668.17	606.09	675.78	U	U	N
TMEM128	4:4237269-4249950	905.84	920.22	942.78	915.24	894.08	917.60	U	U	N
KANK4	1:62702651-62785085	0.00	1.01	1.09	0.00	-0.46	0.46	U	U	N
FOPNL	16:15959577-15982482	2253.19	2559.07	2564.45	2295.62	2128.08	2378.31	U	U	N
ERN2	16:23701647-23724821	0.00	1.01	1.09	0.00	-0.46	0.46	U	U	N
BTF3L4	1:52521797-52556388	4093.64	5153.41	5008.45	4342.01	3709.68	4477.61	U	U	N
AGTPBP1	9:88161455-88356944	559.58	770.04	650.42	589.75	489.77	629.38	U	U	N
MPHOSPH6	16:82181403-82203831	475.24	556.36	519.02	470.51	444.90	505.58	U	U	N
CHAD	17:48541857-48546327	0.00	2.02	1.09	0.00	-0.73	0.73	U	U	N
SCN7A	2:167260083-167350757	0.00	1.01	1.09	0.00	-0.46	0.46	U	U	N
STAM	10:17686124-17757913	1386.04	1573.34	1482.61	1385.75	1318.54	1453.55	U	U	N
NIPSNAP3A	9:107509969-107522403	1100.30	1193.36	1324.93	1094.63	1019.51	1181.09	U	U	N

STX17	9:102668915-102732618	502.03	621.88	579.25	540.33	463.44	540.62	U	U	N
BAAT	9:104122699-104145801	0.99	4.03	4.38	1.07	-0.39	2.37	U	U	N
SLCO2B1	11:74811608-74917594	0.99	2.02	2.19	1.07	0.53	1.46	U	U	N
DYNC2LI1	2:44001178-44037149	844.33	1020.00	974.53	890.53	784.70	903.95	U	U	N
SMEK2	2:55774428-55846015	3911.09	4913.53	4386.50	3837.13	3537.68	4284.50	U	U	N
SIX3	2:45168902-45173216	0.00	3.02	3.28	0.00	-1.37	1.37	U	U	N
ATAD1	10:89511269-89601100	3905.13	4541.62	4294.52	4105.68	3701.61	4108.66	U	U	N
CPNE8	12:39040624-39301232	427.62	545.28	517.93	460.84	387.56	467.68	U	U	N
ZCRB1	12:42705880-42719920	3144.15	3521.62	3409.78	3153.92	3002.79	3285.51	U	U	N
LLPH	12:66516842-66524548	552.63	643.04	635.09	550.00	514.55	590.71	U	U	N
ASCL1	12:103351464-103354294	0.00	1.01	1.09	0.00	-0.46	0.46	U	U	N
FAM222A	12:110152033-110208312	17.86	22.17	30.66	19.34	13.57	22.15	U	U	N
SLC39A5	12:56623833-56631630	1.98	5.04	6.57	3.22	0.47	3.50	U	U	N
VPS33A	12:122714111-122751068	437.54	495.89	512.45	459.77	412.05	463.03	U	U	N
SLC24A4	14:92788925-92962596	0.00	2.02	1.09	0.00	-0.73	0.73	U	U	N
DUOXA1	15:45409569-45422136	5.95	8.06	7.66	5.37	4.98	6.93	U	U	N
BBS4	15:72978527-73030817	691.53	721.66	718.31	699.32	680.59	702.48	U	U	N
CCDC33	15:74509613-74628813	0.00	3.02	1.09	0.00	-1.07	1.07	U	U	N
CYP1A2	15:75041185-75048543	0.00	1.01	1.09	0.00	-0.46	0.46	U	U	N
LRRC46	17:45908993-45915079	5.95	7.06	8.76	6.45	5.04	6.87	U	U	N
SLC25A52	18:29339525-29340843	0.00	1.01	1.09	0.00	-0.46	0.46	U	U	N
SIGLEC10	19:51913275-51921057	0.00	1.01	1.09	0.00	-0.46	0.46	U	U	N
PTH2	19:49925671-49926698	0.00	1.01	1.09	0.00	-0.46	0.46	U	U	N
PRKACB	1:84543745-84704181	1665.83	2181.10	2046.52	1726.28	1479.38	1852.28	U	U	N
DMRTB1	1:53925072-53933161	0.00	1.01	1.09	0.00	-0.46	0.46	U	U	N
TIPRL	1:168148171-168169950	1700.56	2121.64	1898.70	1720.91	1553.90	1847.22	U	U	N
LYPLAL1	1:219347186-219386207	674.67	753.91	718.31	661.72	643.05	706.28	U	U	N
HORMAD1	1:150670536-150693364	0.00	2.02	1.09	0.00	-0.73	0.73	U	U	N
AQP10	1:154293566-154297801	0.00	1.01	2.19	0.00	-0.78	0.78	U	U	N
FBXO28	1:224301789-224349749	1815.65	2354.46	2176.83	1957.24	1637.33	1993.97	U	U	N
POLR2D	2:128603840-128615731	571.48	695.45	662.46	575.78	524.75	618.21	U	U	N
SPAG16	2:214149113-215275225	605.22	794.23	738.02	644.53	540.55	669.88	U	U	N
DYNC1LI1	3:32567463-32612366	1507.09	1787.01	1667.66	1555.48	1413.48	1600.69	U	U	N
CISD2	4:103790135-103810399	1430.69	1758.79	1593.20	1492.10	1323.18	1538.21	U	U	N
METTL14	4:119606523-119636588	1077.48	1295.16	1170.54	1029.11	989.62	1165.35	U	U	N
COMMD10	5:115420688-115748459	722.29	779.11	784.01	707.91	693.14	751.44	U	U	N
AIG1	6:143381633-143661441	1308.66	1520.93	1415.81	1363.19	1240.81	1376.51	U	U	N
TMEM209	7:129804555-129847610	1135.03	1256.86	1180.39	1129.01	1090.78	1179.28	U	U	N
ASB10	7:150872785-150884919	0.00	1.01	1.09	0.00	-0.46	0.46	U	U	N
ZNF157	X:47229982-47273704	0.99	2.02	2.19	1.07	0.53	1.46	U	U	N
RAB41	X:69501943-69504852	0.00	2.02	1.09	0.00	-0.73	0.73	U	U	N
ITGB1BP2	X:70521584-70525221	22.82	26.21	29.56	24.71	20.68	24.96	U	U	N
RIPPLY1	X:106143293-106146565	0.99	4.03	7.66	1.07	-1.37	3.36	U	U	N
GOLGA7	8:41347915-41368499	2662.95	3268.63	3161.22	2799.43	2446.84	2879.06	U	U	N
PMP2	8:82352561-82359758	0.00	1.01	1.09	0.00	-0.46	0.46	U	U	N
ATP6V0D2	8:86999552-87166457	0.00	2.02	1.09	0.00	-0.73	0.73	U	U	N
TATDN1	8:125500726-125551699	1120.15	1428.20	1341.35	1131.16	1004.78	1235.51	U	U	N
FAM135B	8:139142266-139509065	0.00	1.01	1.09	0.00	-0.46	0.46	U	U	N
AK3	9:4711155-4742043	4108.53	5401.36	4824.49	4439.77	3692.11	4524.94	U	U	N
HIATL1	9:97136833-97223324	2569.69	3036.81	2816.30	2660.85	2416.37	2723.01	U	U	N
FAM188A	10:15820169-15902519	1242.18	1530.00	1343.54	1282.62	1146.72	1337.65	U	U	N
ST8SIA6	10:17360382-17496329	0.99	5.04	5.47	0.00	-1.10	3.08	U	U	N
RGR	10:86004809-86019716	0.00	1.01	1.09	0.00	-0.46	0.46	U	U	N
TCF7L2	10:114710009-114927437	1450.54	1608.61	1529.69	1455.57	1394.74	1506.34	U	U	N
TTC12	11:113185251-113254266	148.82	174.37	187.24	154.69	135.52	162.13	U	U	N
DRD2	11:113280318-113346413	0.00	2.02	1.09	0.00	-0.73	0.73	U	U	N

AASDHPPT	11:105946228-105969437	2483.37	3155.75	2841.48	2378.33	2217.41	2749.33	U	U	N
SLX4IP	20:10415951-10617477	20.84	29.23	27.37	23.63	18.01	23.66	U	U	N
CNTN5	11:98891683-100229616	0.00	2.02	2.19	0.00	-0.91	0.91	U	U	N
KLHL1	13:70274726-70682591	0.00	1.01	1.09	0.00	-0.46	0.46	U	U	N
NEK7	1:198126093-198291550	12620.25	16279.66	14235.87	12312.75	11255.95	13984.55	U	U	N
AMN1	12:31824071-31882108	432.58	482.79	452.23	435.06	415.23	449.93	U	U	N
GUF1	4:44680444-44702943	1494.19	1824.31	1616.19	1526.47	1382.74	1605.64	U	U	N
C5orf28	5:43444354-43483995	1309.65	1623.73	1485.89	1354.60	1203.49	1415.80	U	U	N
TMEM178A	2:39892122-39945103	174.62	225.77	193.81	172.95	156.21	193.03	U	U	N
PLEKHH2	2:43864412-43995126	233.16	291.28	271.56	241.70	213.00	253.31	U	U	N
NADK2	5:36192694-36242381	1695.60	2030.93	1831.91	1624.23	1561.39	1829.81	U	U	N
RASGRP3	2:33661391-33789817	18.85	35.28	27.37	19.34	13.03	24.68	U	U	N
SAR1B	5:133936834-133984961	2935.80	3420.83	3336.41	3006.75	2756.21	3115.38	U	U	N
NRSN1	6:24126350-24155128	0.99	2.02	3.28	1.07	0.19	1.79	U	U	N
RGPD3	2:107021446-107084832	2.98	13.10	6.57	5.37	-0.27	6.22	U	U	N
SLC25A27	6:46620678-46645930	145.85	180.41	170.82	140.72	131.46	160.24	U	U	N
FAM49B	8:130851839-131029375	1489.23	1988.59	1828.62	1583.41	1318.30	1660.16	U	U	N
FAM81B	5:94727048-94786158	0.00	3.02	7.66	2.15	-2.42	2.42	U	U	N
FBXL2	3:33318517-33445154	253.00	304.39	286.89	263.18	235.60	270.40	U	U	N
GTF2E1	3:120461484-120501916	379.00	423.32	399.67	378.13	363.01	395.00	U	U	N
PPP2R5E	14:63838075-64010092	3103.47	3821.97	3398.83	3070.13	2842.14	3364.80	U	U	N
CCSAP	1:229456758-229479041	543.70	628.93	574.87	554.30	515.26	572.15	U	U	N
CHODL	21:19273580-19639690	9.92	13.10	16.42	9.67	7.54	12.30	U	U	N
FGD5	3:14860469-14975895	0.00	4.03	2.19	0.00	-1.46	1.46	U	U	N
DKK2	4:107842959-108204963	0.99	2.02	3.28	1.07	0.19	1.79	U	U	N
MARCKS	6:114178541-114184648	11533.84	14113.68	13693.85	12276.23	10629.82	12437.85	U	U	N
ZCCHC10	5:132332677-132362296	528.82	611.80	590.20	512.40	493.04	564.61	U	U	N
RBM45	2:178977151-179003738	237.13	291.28	317.55	251.37	209.51	264.74	U	U	N
SLC26A2	5:149340300-149373018	741.14	882.92	837.66	719.73	682.86	799.42	U	U	N
ADCY8	8:131792547-132054672	0.00	1.01	1.09	0.00	-0.46	0.46	U	U	N
UNC5D	8:35092975-35654068	0.00	1.01	1.09	0.00	-0.46	0.46	U	U	N
MS4A1	11:60223225-60238233	0.00	1.01	1.09	0.00	-0.46	0.46	U	U	N
TBC1D31	8:124054208-124164393	654.82	767.01	704.07	650.98	614.16	695.49	U	U	N
ZNF19	16:71498453-71598992	41.67	45.36	47.08	40.82	39.44	43.90	U	U	N
RPS4Y2	Y:22918050-22942918	0.00	2.02	4.38	1.07	-1.40	1.40	U	U	N
RAB28	4:13362978-13485989	890.96	1065.35	971.25	905.57	831.36	950.56	U	U	N
GPRASP2	X:101967104-101973607	384.96	425.34	460.99	385.65	357.63	412.29	U	U	N
SLAMF8	1:159796540-159807039	8.93	13.10	13.14	7.52	6.77	11.09	U	U	N
M1AP	2:74785010-74875465	5.95	10.08	13.14	7.52	3.59	8.31	U	U	N
ZNF235	19:44732882-44809199	72.43	106.84	87.60	74.12	60.49	84.36	U	U	N
CCDC58	3:122078438-122102078	440.52	502.94	520.12	461.92	413.08	467.95	U	U	N
FGFR4	5:176513887-176525145	74.41	85.67	86.50	75.20	69.51	79.31	U	U	N
PGLYRP2	19:15579456-15609767	0.00	1.01	1.09	0.00	-0.46	0.46	U	U	N
IKZF3	17:37921198-38020441	0.00	3.02	3.28	0.00	-1.37	1.37	U	U	N
NTN3	16:2521500-2524146	0.99	4.03	2.19	1.07	-0.07	2.05	U	U	N
ARPC5	1:183592401-183604892	13714.60	17448.83	16867.12	14917.75	12417.82	15011.39	U	U	N
PEX13	2:61244360-61279125	1943.64	2304.07	2147.26	1988.39	1820.62	2066.66	U	U	N
KIAA1841	2:61293006-61391960	252.01	382.00	347.11	273.93	206.25	297.77	U	U	N
MEMO1	2:32090129-32236299	192.48	220.73	208.05	189.06	181.49	203.47	U	U	N
C1QTNF7	4:15341442-15447790	10.91	21.17	24.09	12.89	6.15	15.68	U	U	N
S100A9	1:153330330-153333503	0.00	1.01	1.09	0.00	-0.46	0.46	U	U	N
GABRB1	4:46995740-47428461	1.98	3.02	3.28	2.15	1.50	2.46	U	U	N
TMEM183A	1:202976514-202993976	1958.52	2182.11	2083.75	1988.39	1882.63	2034.41	U	U	N
EFHB	3:19920964-19988517	6.95	13.10	9.85	7.52	4.85	9.04	U	U	N
SMIM14	4:39547950-39640710	5624.54	6718.69	6383.75	5530.10	5189.58	6059.51	U	U	N
TTC14	3:180319918-180335616	1355.29	1734.60	1550.50	1400.79	1226.87	1483.70	U	U	N

LZTFL1	3:45864808-45957534	789.76	958.52	918.69	822.86	730.32	849.20	U	U	N
UBXN7	3:196074533-196159345	3055.85	3581.08	3289.33	3085.17	2874.19	3237.50	U	U	N
PIGX	3:196366646-196462878	1579.52	1765.85	1655.61	1627.45	1520.26	1638.77	U	U	N
INTU	4:128544426-128647892	770.91	811.36	819.05	782.03	753.63	788.18	U	U	N
CAMKV	3:49895421-49907655	8.93	12.09	14.23	7.52	6.65	11.21	U	U	N
STARD4	5:110831731-110848288	1211.42	1551.16	1407.05	1192.39	1083.41	1339.44	U	U	N
PRIMPOL	4:185570767-185616117	445.48	518.06	508.07	461.92	419.11	471.85	U	U	N
GJB7	6:87992696-88038996	0.00	3.02	5.47	0.00	-1.99	1.99	U	U	N
MB21D1	6:74123238-74161999	486.16	678.32	583.63	534.96	424.66	547.66	U	U	N
PEX2	8:77892494-77913280	1544.79	1680.18	1665.47	1508.21	1480.29	1609.29	U	U	N
SBSPON	8:73976775-74036323	1.98	5.04	3.28	2.15	0.93	3.04	U	U	N
ORC5	7:103766788-103848495	886.00	1064.35	1145.35	968.95	801.38	970.61	U	U	N
PHAX	5:125935960-125962944	2270.06	2588.30	2497.66	2334.29	2160.38	2379.74	U	U	N
SNAPC3	9:15422702-15465951	1898.00	2290.97	2087.04	1877.74	1753.29	2042.70	U	U	N
CPA6	8:68334360-68658620	5.95	15.12	9.85	6.45	2.79	9.12	U	U	N
DYNLT3	X:37696010-37706890	1039.78	1341.52	1196.82	980.77	917.89	1161.67	U	U	N
PTCHD1	X:23352133-23422489	9.92	16.13	18.61	9.67	6.55	13.29	U	U	N
ATP7A	X:77166194-77305892	433.57	580.55	496.03	465.14	386.18	480.97	U	U	N
CLDN2	X:106143394-106174091	0.99	4.03	4.38	0.00	-0.65	2.63	U	U	N
MICU2	13:22066836-22178353	1097.33	1278.02	1212.15	1074.22	1025.11	1169.55	U	U	N
AMER2	13:25735822-25746426	0.00	1.01	1.09	0.00	-0.46	0.46	U	U	N
SLC18A2	10:119000604-119038941	0.99	8.06	12.04	4.30	-2.59	4.57	U	U	N
VWA2	10:115999018-116051272	3.97	8.06	8.76	4.30	2.10	5.84	U	U	N
E2F7	12:77415027-77459360	223.24	279.19	277.03	240.63	202.53	243.94	U	U	N
PTER	10:16478942-16555736	232.16	292.29	252.94	237.40	211.77	252.56	U	U	N
ARL5B	10:18948334-18970568	5825.95	7793.11	6652.02	5767.51	5116.32	6535.59	U	U	N
STXBP4	17:53046088-53241646	178.59	219.72	202.57	177.25	163.27	193.91	U	U	N
C16orf46	16:81087102-81110872	29.76	32.25	37.23	31.15	27.33	32.20	U	U	N
TMX3	18:66340925-66382535	7817.21	10042.75	8955.86	7733.34	6999.46	8634.96	U	U	N
HSP90B1	12:104323885-104347423	32873.17	36858.04	35270.49	33407.29	31507.10	34239.24	U	U	N
TAC3	12:57403784-57422667	0.00	1.01	1.09	0.00	-0.46	0.46	U	U	N
AKTIP	16:53524952-53538323	1030.85	1195.37	1158.49	1081.74	975.18	1086.52	U	U	N
DUSP18	22:31048038-31063877	139.89	150.18	153.30	138.57	134.38	145.41	U	U	N
HDHD2	18:44633774-44676891	1009.02	1229.64	1158.49	1034.48	930.90	1087.14	U	U	N
MMP26	11:4726157-5013659	0.00	1.01	1.09	0.00	-0.46	0.46	U	U	N
IRGQ	19:44088521-44100287	547.67	614.82	575.96	565.04	526.33	569.02	U	U	N
GGT6	17:4460222-4464113	0.00	1.01	1.09	0.00	-0.46	0.46	U	U	N
TMEM68	8:56608983-56685966	1128.08	1380.83	1253.76	1130.08	1037.77	1218.39	U	U	N
HOOK3	8:42752075-42885682	3758.29	4742.19	4194.88	3754.41	3407.32	4109.27	U	U	N
PCMTD1	8:52730140-52811735	3691.82	4818.79	4185.02	3670.62	3286.87	4096.77	U	U	N
RFWD3	16:74655292-74700779	1032.84	1237.71	1140.97	1042.00	960.64	1105.04	U	U	N
MTNR1A	4:187454809-187476721	0.00	1.01	2.19	0.00	-0.78	0.78	U	U	N
DNAJC21	5:34929698-34959069	3317.78	3967.11	3536.79	3426.77	3104.46	3531.09	U	U	N
INPP5D	2:233924677-234116549	2.98	12.09	14.23	2.15	-1.67	7.62	U	U	N
RSPO1	1:38076951-38100595	1.98	10.08	8.76	4.30	-0.85	4.82	U	U	N
GCSAML	1:247670360-247740992	0.00	2.02	1.09	0.00	-0.73	0.73	U	U	N
SLC33A1	3:155538813-155572218	2272.04	2710.25	2506.42	2175.30	2091.72	2452.36	U	U	N
CD52	1:26644448-26647014	0.00	1.01	1.09	0.00	-0.46	0.46	U	U	N
DTWD2	5:118173017-118324240	164.70	186.46	212.43	175.10	149.30	180.09	U	U	N
RGPD8	2:113127669-113192065	55.56	60.47	65.70	58.01	52.31	58.81	U	U	N
CSGALNACT2	10:43633934-43680756	4032.13	5009.28	4382.12	4113.20	3700.06	4364.20	U	U	N
ZNF35	3:44690219-44702283	140.89	166.30	175.20	151.47	129.43	152.34	U	U	N
FOXD4	9:116237-118417	19.84	29.23	26.28	18.26	15.94	23.75	U	U	N
FAM161A	2:62051989-62081278	162.71	183.44	192.72	156.84	150.01	175.42	U	U	N
FRMPD2	10:49364601-49482941	0.00	1.01	2.19	0.00	-0.78	0.78	U	U	N
SMAD1	4:146402346-146479231	333.37	347.73	349.30	338.38	327.66	339.07	U	U	N

SOX7	8:10581278-10697357	3.97	8.06	10.95	3.22	1.25	6.69	U	U	N
WIPF2	17:38375556-38440388	1718.42	1889.82	1789.20	1687.61	1651.10	1785.74	U	U	N
FRMD3	9:85857905-86153461	8.93	12.09	10.95	8.59	7.68	10.18	U	U	N
RASGRP1	15:38780304-38857776	167.67	200.57	188.34	166.50	155.24	180.11	U	U	N
LCLAT1	2:30670092-30867091	748.09	926.26	873.80	773.44	685.20	810.98	U	U	N
ARPP21	3:35680437-35835988	0.00	1.01	1.09	0.00	-0.46	0.46	U	U	N
KIAA1919	6:111580551-111592370	345.27	501.94	420.47	338.38	287.84	402.71	U	U	N
EIF1AX	X:20142636-20159962	4675.05	5750.09	5547.18	4687.91	4251.66	5098.44	U	U	N
KIAA1239	4:37245842-37451087	0.99	2.02	2.19	1.07	0.53	1.46	U	U	N
LIG4	13:108859787-108870716	766.94	1174.21	919.79	867.97	636.92	896.96	U	U	N
GOLGA6L2	15:23684645-23692381	0.00	1.01	2.19	0.00	-0.78	0.78	U	U	N
SLC26A9	1:205882176-205912588	0.99	3.02	2.19	1.07	0.26	1.72	U	U	N
DHX36	3:153990335-154042286	3741.43	4436.79	4067.86	3726.48	3490.10	3992.75	U	U	N
INHBC	12:57828543-57844611	2.98	9.07	5.47	4.30	1.01	4.94	U	U	N
APOF	12:56754353-56756607	0.99	2.02	2.19	1.07	0.53	1.46	U	U	N
SLC35E3	12:69139886-69187508	465.32	516.05	500.41	455.47	443.85	486.79	U	U	N
PLEKHD1	14:69951409-69995215	2.98	9.07	9.85	4.30	0.41	5.54	U	U	N
HMGB4	1:34326076-34330392	0.00	2.02	1.09	0.00	-0.73	0.73	U	U	N
CCDC121	2:27848506-27851879	71.44	81.64	98.55	71.97	61.94	80.93	U	U	N
TCEANC	X:13671225-13700083	42.66	49.39	49.27	42.97	39.84	45.49	U	U	N
C10orf85	10:122357721-122359629	10.91	12.09	13.14	10.74	10.07	11.75	U	U	N
MBOAT4	8:29989340-30002202	0.00	1.01	1.09	0.00	-0.46	0.46	U	U	N
ODF3	11:196738-200261	0.00	1.01	1.09	0.00	-0.46	0.46	U	U	N
MLF1	3:158288952-158325041	716.34	765.00	812.48	700.39	678.31	754.37	U	U	N
ALOXE3	17:7999218-8022365	7.94	9.07	9.85	7.52	7.14	8.74	U	U	N
HNRNPCL1	1:12907261-12908578	0.00	1.01	2.19	0.00	-0.78	0.78	U	U	N
HTR1D	1:23516993-23521222	2.98	8.06	5.47	4.30	1.35	4.60	U	U	N
C1orf194	1:109648573-109656479	0.00	1.01	2.19	0.00	-0.78	0.78	U	U	N
ITLN1	1:160846329-160854960	0.99	3.02	4.38	1.07	-0.24	2.22	U	U	N
BBS10	12:76738254-76742222	795.71	1048.22	954.83	798.15	702.62	888.81	U	U	N
PRKRA	2:179296141-179316239	2249.22	2619.54	2460.43	2290.25	2121.84	2376.60	U	U	N
SSTR2	17:71161151-71167185	0.00	3.02	2.19	0.00	-1.16	1.16	U	U	N
HIGD1A	3:42798669-42846023	3676.94	4226.14	4261.67	3657.73	3426.97	3926.91	U	U	N
OFCC1	6:9596343-10211841	0.00	1.01	1.09	0.00	-0.46	0.46	U	U	N
WRB	21:40752170-40800454	1576.54	1733.59	1677.51	1559.77	1514.40	1638.68	U	U	N
FAM89A	1:231154704-231175992	196.45	227.79	262.80	211.62	175.10	217.79	U	U	N
RGMA	15:93586636-93632433	4.96	9.07	7.66	5.37	3.50	6.42	U	U	N
ATP6AP2	X:40440146-40465889	5581.88	6530.21	5922.76	5628.93	5254.58	5909.18	U	U	N
TRAPPC6B	14:39617015-39639736	1681.71	1987.59	1858.19	1661.82	1565.82	1797.60	U	U	N
RWDD4	4:184560788-184580378	803.65	1039.15	1001.91	792.78	706.65	900.64	U	U	N
GRID1	10:87359312-88126250	5.95	12.09	9.85	6.45	3.77	8.14	U	U	N
SLC25A18	22:18043139-18073760	1.98	4.03	4.38	2.15	1.05	2.92	U	U	N
SLC25A10	17:79670404-79688042	66.47	75.59	88.69	65.53	58.41	74.54	U	U	N
RIMBP3C	22:21899646-21905750	0.00	1.01	1.09	0.00	-0.46	0.46	U	U	N
GOLGA8G	15:28764757-28778160	0.00	1.01	1.09	0.00	-0.46	0.46	U	U	N
PP13439	3:171509580-171527714	21.83	27.21	35.04	21.48	17.08	26.58	U	U	N
BMP8A	1:39957318-39991607	10.91	12.09	12.04	10.74	10.37	11.45	U	U	N
NPBWR1	8:53850991-53853677	0.00	1.01	1.09	0.00	-0.46	0.46	U	U	N
KREMEN1	22:29469066-29564321	348.25	371.92	362.44	342.68	338.26	358.23	U	U	N
PPP1R2	3:195241221-195270209	3033.03	3631.48	3602.49	3164.66	2804.90	3261.15	U	U	N
HIST1H2AK	6:27805658-27806117	6.95	13.10	12.04	8.59	4.78	9.11	U	U	N
PIWIL3	22:25115001-25170687	0.00	1.01	1.09	0.00	-0.46	0.46	U	U	N
RBM43	2:152104454-152118393	721.30	913.16	867.23	757.33	653.56	789.03	U	U	N
PURA	5:139487362-139496321	1030.85	1146.99	1093.89	1020.51	986.60	1075.10	U	U	N
GP1BA	17:4835592-4838325	0.00	4.03	2.19	1.07	-1.29	1.29	U	U	N
HS6ST3	13:96743093-97485671	0.99	5.04	3.28	1.07	-0.47	2.45	U	U	N

STAC3	12:57637236-57644976	35.72	56.44	52.56	39.75	28.26	43.18	U	U	N
CCDC30	1:42929001-43120335	71.44	91.72	79.93	76.27	64.95	77.92	U	U	N
TRDN	6:123537483-123958238	0.00	3.02	2.19	0.00	-1.16	1.16	U	U	N
INSIG1	7:155089486-155101945	2525.04	3035.81	2802.06	2680.19	2363.36	2686.72	U	U	N
LYRM7	5:130506503-130541119	810.59	1057.29	909.93	806.74	722.41	898.77	U	U	N
TNFRSF4	1:1146706-1149518	9.92	12.09	14.23	10.74	8.51	11.33	U	U	N
SELV	19:40005753-40011326	0.00	3.02	2.19	0.00	-1.16	1.16	U	U	N
EFCAB6	22:43924624-44208217	42.66	51.40	59.13	40.82	36.32	49.00	U	U	N
CTD-2600O9.1	16:57832098-57850850	0.00	1.01	2.19	0.00	-0.78	0.78	U	U	N
LUZP2	11:24518516-25104150	5.95	8.06	7.66	5.37	4.98	6.93	U	U	N
RGPD1	2:87135076-87241104	0.99	5.04	4.38	1.07	-0.61	2.60	U	U	N
DNAJB13	11:73661364-73681411	6.95	14.11	15.33	9.67	4.02	9.87	U	U	N
ADH1A	4:100197524-100212185	0.00	1.01	1.09	0.00	-0.46	0.46	U	U	N
TMEM221	19:17546318-17559376	2.98	5.04	7.66	3.22	1.35	4.60	U	U	N
AGAP4	10:46321042-46349323	24.80	37.29	39.42	29.00	19.64	29.97	U	U	N
PLSCR1	3:146232967-146262651	2544.89	3264.60	2976.16	2723.16	2310.08	2779.69	U	U	N
BLOC1S5	6:8013800-8064647	395.87	443.48	460.99	380.28	367.18	424.57	U	U	N
SLC4A5	2:74443369-74570541	40.68	58.46	54.75	46.19	34.62	46.73	U	U	N
ZNF563	19:12428291-12444534	59.53	84.66	68.98	65.53	51.48	67.58	U	U	N
BTBD8	1:92545862-92613393	10.91	13.10	14.23	10.74	9.63	12.19	U	U	N
GJB5	1:35220648-35224113	0.00	1.01	1.09	0.00	-0.46	0.46	U	U	N
C6orf222	6:36283534-36304662	0.00	1.01	1.09	0.00	-0.46	0.46	U	U	N
RP11-113D6.10	11:18230685-18236109	0.00	1.01	1.09	0.00	-0.46	0.46	U	U	N
XPNPEP3	22:41253081-41363838	899.89	1092.57	976.72	954.98	839.08	960.70	U	U	N
ELAVL3	19:11562141-11591861	1.98	5.04	4.38	2.15	0.82	3.15	U	U	N
C9orf163	9:139377947-139380518	1.98	4.03	3.28	2.15	1.26	2.71	U	U	N
HIST1H2BM	6:27782822-27783267	0.00	2.02	2.19	0.00	-0.91	0.91	U	U	N
ZNF774	15:90895477-90909324	43.65	75.59	59.13	40.82	31.63	55.68	U	U	N
MYO6	6:76458909-76629254	3318.77	3790.73	3510.51	3310.76	3149.90	3487.64	U	U	N
GOLGA6L9	15:83098710-83108085	16.87	28.22	35.04	19.34	10.60	23.13	U	U	N
TTC30B	2:178413726-178417742	104.18	130.02	113.88	109.57	95.82	112.53	U	U	N
CBWD3	9:70856397-70914929	47.62	53.42	59.13	47.27	43.42	51.83	U	U	N
ANXA4	2:69871557-70053596	1909.90	2122.65	2006.01	1936.83	1838.71	1981.10	U	U	N
FAM163B	9:136444079-136451319	0.00	1.01	1.09	0.00	-0.46	0.46	U	U	N
ZNF682	19:20107867-20150315	101.20	125.99	111.69	106.35	93.19	109.21	U	U	N
IPP	1:46159996-46216322	614.15	660.18	722.69	632.72	578.57	649.72	U	U	N
PCDHB11	5:140579183-140582618	2.98	6.05	4.38	3.22	1.93	4.03	U	U	N
RPF2	6:111303218-111349466	1814.66	2007.74	2068.42	1843.37	1721.82	1907.49	U	U	N
WDR96	10:105889646-105992120	42.66	51.40	50.37	45.12	39.53	45.80	U	U	N
ZNF780A	19:40575059-40596845	163.71	182.43	185.05	167.58	155.74	171.67	U	U	N
ZNF347	19:53627325-53662328	214.31	313.46	255.13	238.48	182.65	245.97	U	U	N
LEKR1	3:156543270-156763918	19.84	25.20	27.37	19.34	16.86	22.82	U	U	N
IRAK4	12:44152747-44183346	1065.58	1149.01	1112.50	1080.67	1037.85	1093.31	U	U	N
MRPL42	12:93861264-93897545	2666.92	3051.93	3010.11	2582.43	2488.62	2845.22	U	U	N
ZNF770	15:35270542-35280488	3235.43	3911.68	3673.67	3139.96	2961.95	3508.91	U	U	N
AKR1C4	10:5237425-5260912	0.00	2.02	1.09	0.00	-0.73	0.73	U	U	N
C3orf27	3:128290843-128294929	1.98	4.03	3.28	2.15	1.26	2.71	U	U	N
RYR3	15:33603163-34158303	0.99	2.02	3.28	1.07	0.19	1.79	U	U	N
EFCAB2	1:245133007-245290466	189.50	210.65	200.38	187.99	181.57	197.43	U	U	N
AC092811.1	1:225600404-225602040	0.00	1.01	1.09	0.00	-0.46	0.46	U	U	N
ECT2L	6:139117063-139225207	1.98	6.05	4.38	2.15	0.53	3.44	U	U	N
HIST1H3H	6:27777842-27778314	5.95	13.10	10.95	6.45	3.34	8.57	U	U	N
RIPPLY2	6:84562985-84567234	0.99	6.05	3.28	0.00	-1.02	3.01	U	U	N
NAP1L6	X:72345876-72347919	0.99	4.03	8.76	1.07	-1.74	3.73	U	U	N
C6orf47	6:31626075-31628549	610.18	642.03	624.14	611.23	599.04	621.32	U	U	N
TRIM31	6:30070674-30080883	0.99	2.02	3.28	1.07	0.19	1.79	U	U	N

REXO1L1	8:86568695-86575726	0.00	1.01	2.19	0.00	-0.78	0.78	U	U	N
AC074091.13	2:27928653-27938599	0.00	4.03	2.19	1.07	-1.29	1.29	U	U	N
LYRM5	12:25348150-25362579	564.54	770.04	769.77	634.87	487.76	641.32	U	U	N
AL359878.1	10:1017097-1034281	0.00	1.01	1.09	0.00	-0.46	0.46	U	U	N
PTPLB	3:123209667-123304032	2362.33	2658.85	2544.74	2356.85	2251.77	2472.89	U	U	N
TAS2R14	12:11090005-11324172	68.46	85.67	90.88	70.90	60.21	76.70	U	U	N
AL161915.1	1:54569968-54571750	36.71	48.38	45.99	34.38	31.57	41.85	U	U	N
AC114546.1	15:35270552-35272268	40.68	48.38	48.18	41.89	37.63	43.73	U	U	N
AC073063.1	7:99040552-99040747	0.00	6.05	4.38	1.07	-2.11	2.11	U	U	N
COL28A1	7:7395834-7575484	4.96	10.08	8.76	6.45	3.24	6.68	U	U	N
CBWD7	9:42668608-42714962	4.96	7.06	7.66	5.37	3.98	5.94	U	U	N
AC007390.5	2:37423631-37443336	3032.04	3659.70	3505.04	2943.37	2768.82	3295.25	U	U	N
FAM200A	7:99143931-99156159	224.23	239.88	255.13	225.59	213.38	235.08	U	U	N
HEPN1	11:124789146-124790573	0.00	1.01	1.09	0.00	-0.46	0.46	U	U	N
C1orf229	1:247273462-247275719	1.98	6.05	4.38	3.22	0.69	3.28	U	U	N
OR52B4	11:4388493-4389616	0.00	1.01	1.09	0.00	-0.46	0.46	U	U	N
C10orf55	10:75669727-75682535	0.00	1.01	2.19	0.00	-0.78	0.78	U	U	N
LTB	6:31548302-31550299	0.00	3.02	1.09	0.00	-1.07	1.07	U	U	N
AC002553.1	17:15943505-15943994	1.98	7.06	4.38	3.22	0.36	3.61	U	U	N
MTRNR2L4	16:3421053-3422283	0.00	1.01	2.19	0.00	-0.78	0.78	U	U	N
OR1J4	9:125281420-125282361	0.99	5.04	4.38	1.07	-0.61	2.60	U	U	N
JMJD7	15:42120283-42129779	0.99	6.05	3.28	1.07	-0.79	2.78	U	U	N
ZNF512	2:27805897-27858041	302.61	363.85	330.68	288.97	277.76	327.46	U	U	N
TMEM110-MUSTN1	3:52867137-52931578	0.99	9.07	5.47	1.07	-1.93	3.91	U	U	N
RP11-766F14.2	4:100557686-100575805	0.00	1.01	1.09	0.00	-0.46	0.46	U	U	N
AC005609.1	5:140240341-140243224	0.99	2.02	3.28	1.07	0.19	1.79	U	U	N
ATP5L2	22:43035809-43036607	0.00	3.02	4.38	1.07	-1.47	1.47	U	U	N
PCDHGA3	5:140723601-140892546	15.87	20.16	25.18	18.26	12.91	18.84	U	U	N
SDHD	11:111957627-112064528	0.00	1.01	1.09	0.00	-0.46	0.46	U	U	N
MTRNR2L1	17:22022437-22023991	0.00	1.01	1.09	0.00	-0.46	0.46	U	U	N
LIMS3L	2:111222628-111230511	0.00	2.02	1.09	0.00	-0.73	0.73	U	U	N
RP11-144F15.1	12:106889736-107168696	0.00	1.01	1.09	0.00	-0.46	0.46	U	U	N
ZNF625	19:12251032-12267546	0.99	2.02	2.19	1.07	0.53	1.46	U	U	N
SPECC1L-ADORA2A	22:24666866-24838324	0.00	5.04	3.28	1.07	-1.69	1.69	U	U	N
RP11-597K23.2	15:82380935-82390035	6.95	12.09	14.23	7.52	4.29	9.60	U	U	N
GOLGA8T	15:30427352-30437770	0.99	2.02	3.28	1.07	0.19	1.79	U	U	N
GOLGA8H	15:30896329-30906764	1.98	4.03	7.66	2.15	0.00	3.96	U	U	N
CTB-133G6.1	19:7413848-7448271	0.00	1.01	2.19	0.00	-0.78	0.78	U	U	N
AP000349.1	22:24124467-24126145	0.00	1.01	1.09	0.00	-0.46	0.46	U	U	N
AL590452.1	1:144989309-144991365	25.80	36.28	39.42	26.86	20.70	30.89	U	U	N
AC007382.1	2:37068327-37068530	19.84	37.29	37.23	24.71	13.19	26.50	U	U	N
AC069547.1	10:52094298-52095876	0.00	1.01	1.09	0.00	-0.46	0.46	U	U	N
AC024257.1	12:48759919-48761738	0.00	1.01	1.09	0.00	-0.46	0.46	U	U	N
AL669831.1	1:738532-739137	0.00	1.01	1.09	0.00	-0.46	0.46	U	U	N
AC079602.1	12:121407641-121410095	1.98	6.05	4.38	1.07	0.29	3.68	U	U	N

Supplementary Table 2. Enrichment analysis results showing the involvement of shortlisted candidates that are involved in several important canonical pathways such as: Leptin Signaling in Obesity, Estrogen Biosynthesis, Androgen Biosynthesis, Sonic Hedgehog Signaling, PDGF Signaling, Human Embryonic Stem Cell Pluripotency.

Ingenuity Canonical Pathways	-log(p-value)	Down regulated	Overlaps with dataset	Up regulated	No overlap with dataset	Molecules
Estrogen Biosynthesis	2.68	0/41 (0%)	8/41 (20%)	0/41 (0%)	33/41 (80%)	AKR1B15,AKR1C4,CYP19A1,CYP1A1,CYP1A2,CYP2D6,CYP4X1,HSD17B3
Tight Junction Signaling	2.41	0/168 (0%)	19/168 (11%)	0/168 (0%)	149/168 (89%)	ACTG2,CLDN14,CLDN15,CLDN2,CPSF1,HSF1,LLGL1,MYH11,MYL2,MYL4, NAPB,NECTIN2,PATJ,PPP2CA,PPP2CB,PPP2R5E,PRKACB,TGFB2,VAPA
Oxidative Phosphorylation	2.39	0/109 (0%)	14/109 (13%)	0/109 (0%)	95/109 (87%)	ATP5F1D,ATP5F1E,ATP5MGL,COX5A,COX7A2L,COX8A,NDUFA3,NDUFA9 NDUFB10,SDHC,SDHD,UQCR10,UQCRFS1,UQCRQ
Sphingosine and Sphingosine-1-phosphate Metabolism	2.06	0/8 (0%)	3/8 (38%)	0/8 (0%)	5/8 (63%)	ACER3,ASAH2B,SGPP1
Gαs Signaling	2.06	0/107 (0%)	13/107 (12%)	0/107 (0%)	94/107 (88%)	ADCY2,ADCY8,ADORA2A,CNGA4,CREB1,CRHR1,HCAR2,MAPK3,MC4R,PR KACB,RYR3,SRC,VIPR2
Systemic Lupus Erythematosus In B Cell Signaling Pathway	2.02	0/275 (0%)	26/275 (9%)	0/275 (0%)	249/275 (91%)	BAD,CD70,CD72,CSK,ERAS,FCGR2B,FOXO4,FOXO6,IL17C,IL37,INPP5D,I RAK4,LTB,MAPK3,MYD88,NFATC2,RALA,RASGRP1,RASGRP3,SRC,TGFB 2,TLR9,TNFRSF13C,TRAF1,TRAF5,TYK2
D-glucuronate Degradation I	2.02	0/3 (0%)	2/3 (67%)	0/3 (0%)	1/3 (33%)	CRYL1,DCXR
Geranylgeranyldiphosphate Biosynthesis	2.02	0/3 (0%)	2/3 (67%)	0/3 (0%)	1/3 (33%)	FNTB,GGPS1
Mitochondrial Dysfunction	2	0/171 (0%)	18/171 (11%)	0/171 (0%)	153/171 (89%)	APH1A,ATP5F1D,ATP5F1E,ATP5MGL,COX5A,COX7A2L,COX8A,DHODH,N DUFA3,NDUFA9,NDUFB10,PRDX3,SDHC,SDHD,SOD2,UQCR10,UQCRFS1 UQCRQ
Ceramide Signaling	1.91	0/88 (0%)	11/88 (13%)	0/88 (0%)	77/88 (88%)	BAD,ERAS,MAPK3,PPP2CA,PPP2CB,PPP2R5E,RALA,S1PR4,SMPD3,SPH K1,SPHK2
Bupropion Degradation	1.89	0/25 (0%)	5/25 (20%)	0/25 (0%)	20/25 (80%)	CYP19A1,CYP1A1,CYP1A2,CYP2D6,CYP4X1
G-Protein Coupled Receptor Signaling	1.84	0/271 (0%)	25/271 (9%)	0/271 (0%)	246/271 (91%)	ADCY2,ADCY8,ADORA2A,ADRA1A,AVPR1A,CALCR,CREB1,CRHR1,DRD2, ERAS,GRK4,HCAR2,HTR1D,LAMTOR3,LTB4R,MAPK3,MC4R,OPRL1,PLCB 3,PRKACB,RALA,RASGRP1,SRC,SSTR3,VIPR2
Apelin Adipocyte Signaling Pathway	1.74	0/81 (0%)	10/81 (12%)	0/81 (0%)	71/81 (88%)	ADCY2,ADCY8,GSTP1,GSTT2/GSTT2B,GSTZ1,LIPE,MAPK3,NOX1,NOX3, PRKACB
Phospholipase C Signaling	1.6	0/257 (0%)	23/257 (9%)	0/257 (0%)	234/257 (91%)	ADCY2,ADCY8,ARHGEF16,ARHGEF9,CD247,CD3E,CREB1,ERAS,FCGR2B HDAC10,MAPK3,MARCKS,MYL2,MYL4,NFATC2,PLA2G12A,PLA2G5,PLCB 3,PLCD1,PLD5,RALA,RHOT1,SRC
Inflammasome pathway	1.59	0/20 (0%)	4/20 (20%)	0/20 (0%)	16/20 (80%)	MYD88,NEK7,NLRC4,PYCARD
Estrogen-Dependent Breast Cancer Signaling	1.58	0/74 (0%)	9/74 (12%)	0/74 (0%)	65/74 (88%)	AKR1B15,AKR1C4,CREB1,CYP19A1,ERAS,HSD17B3,MAPK3,RALA,SRC
Protein Ubiquitination Pathway	1.57	0/273 (0%)	24/273 (9%)	0/273 (0%)	249/273 (91%)	ANAPC2,CRYAB,DNAJB13,DNAJB2,DNAJB9,DNAJC10,DNAJC12,DNAJC21 ,DNAJC3,ELOB,FZR1,HSP90B1,HSPA1L,HSPB7,PSMB5,PSMB7,SKP2,UB E2A,UBE2S,UBE2V2,USP2,USP20,USP51,USP6
Estrogen Receptor Signaling	1.57	0/137 (0%)	14/137 (10%)	0/137 (0%)	123/137 (90%)	ERAS,G6PC,GTF2E1,MAPK3,MED10,MED18,POLR2C,POLR2D,POLR2F,P OLR2I,RALA,SMARCA4,SRC,TAF7L

Acetone Degradation I (to Methylglyoxal)	1.57	0/30 (0%)	5/30 (17%)	0/30 (0%)	25/30 (83%)	CYP19A1,CYP1A1,CYP1A2,CYP2D6,CYP4X1
TREM1 Signaling	1.55	0/75 (0%)	9/75 (12%)	0/75 (0%)	66/75 (88%)	FCGR2B,MAPK3,MPO,MYD88,NLRC4,NLRP14,NOD1,TLR6,TLR9
Gαi Signaling	1.54	0/125 (0%)	13/125 (10%)	0/125 (0%)	112/125 (90%)	ADCY2,ADCY8,DRD2,ERAS,HCAR2,HTR1D,LTB4R,MAPK3,OPRL1,PRKACB,RALA,SRC,SSTR3
Trans, trans-farnesyl Diphosphate Biosynthesis	1.53	0/5 (0%)	2/5 (40%)	0/5 (0%)	3/5 (60%)	GGPS1,IDI1
Tyrosine Degradation I	1.53	0/5 (0%)	2/5 (40%)	0/5 (0%)	3/5 (60%)	FAH,GSTZ1
Superpathway of Melatonin Degradation	1.48	0/65 (0%)	8/65 (12%)	0/65 (0%)	57/65 (88%)	CYP19A1,CYP1A1,CYP1A2,CYP2D6,CYP4X1,MPO,SULT1A2,SULT1C4
Dopamine Receptor Signaling	1.48	0/77 (0%)	9/77 (12%)	0/77 (0%)	68/77 (88%)	ADCY2,ADCY8,DRD2,GCH1,PPP2CA,PPP2CB,PPP2R5E,PRKACB,SLC18A2
Urate Biosynthesis/Inosine 5'-phosphate Degradation	1.45	0/13 (0%)	3/13 (23%)	0/13 (0%)	10/13 (77%)	IMPDH1,NT5C,NT5C3A
cAMP-mediated signaling	1.4	0/227 (0%)	20/227 (9%)	0/227 (0%)	207/227 (91%)	ADCY2,ADCY8,ADORA2A,AKAP14,CNGA4,CREB1,CRHR1,DRD2,GRK4,HC AR2,HTR1D,LAMTOR3,LTB4R,MAPK3,MC4R,OPRL1,PRKACB,SRC,SSTR3, VIPR2
Aldosterone Signaling in Epithelial Cells	1.4	0/158 (0%)	15/158 (9%)	0/158 (0%)	143/158 (91%)	AHCY,CRYAB,DNAJB13,DNAJB2,DNAJB9,DNAJC10,DNAJC12,DNAJC21,DNAJC3,HSP90B1,HSPA1L,HSPB7,MAPK3,PLCB3,PLCD1
Ceramide Degradation	1.37	0/6 (0%)	2/6 (33%)	0/6 (0%)	4/6 (67%)	ACER3,ASAH2B
Androgen Biosynthesis	1.36	0/14 (0%)	3/14 (21%)	0/14 (0%)	11/14 (79%)	AKR1C4,EBP,HSD17B3
HIPPO signaling	1.25	0/85 (0%)	9/85 (11%)	0/85 (0%)	76/85 (89%)	LLGL1,MOB1A,PATJ,PPP2CA,PPP2CB,PPP2R5E,SKP2,SMAD1,SMAD5
BMP signaling pathway	1.25	0/85 (0%)	9/85 (11%)	0/85 (0%)	76/85 (89%)	BMP8A,CREB1,ERAS,HOXC9,MAPK3,PRKACB,RALA,SMAD1,SMAD5
Melatonin Degradation I	1.25	0/60 (0%)	7/60 (12%)	0/60 (0%)	53/60 (88%)	CYP19A1,CYP1A1,CYP1A2,CYP2D6,CYP4X1,SULT1A2,SULT1C4
Acyl Carrier Protein Metabolism	1.24	0/1 (0%)	1/1 (100%)	0/1 (0%)	0/1 (0%)	AASDHPPT
Melatonin Degradation III	1.24	0/1 (0%)	1/1 (100%)	0/1 (0%)	0/1 (0%)	MPO
Thiamin Salvage III	1.24	0/1 (0%)	1/1 (100%)	0/1 (0%)	0/1 (0%)	TPK1
Asparagine Biosynthesis I	1.24	0/1 (0%)	1/1 (100%)	0/1 (0%)	0/1 (0%)	ASNS
D-mannose Degradation	1.24	0/1 (0%)	1/1 (100%)	0/1 (0%)	0/1 (0%)	MPI
Epithelial Adherens Junction Signaling	1.23	0/153 (0%)	14/153 (9%)	0/153 (0%)	139/153 (91%)	ACTG2,ARPC5,ERAS,MYH11,MYL2,MYL4,NECTIN2,RALA,SNAI1,SRC,TCF7L2,TGFB2,TUBB4B,VAV2
Hereditary Breast Cancer Signaling	1.21	0/140 (0%)	13/140 (9%)	0/140 (0%)	127/140 (91%)	ERAS,FAAP100,FANCC,H2AFX,HDAC10,POLR2C,POLR2D,POLR2F,POLR2 I,RALA,SLC19A1,SMARCA4,SMARCC2
Gap Junction Signaling	1.18	0/198 (0%)	17/198 (9%)	0/198 (0%)	181/198 (91%)	ACTG2,ADCY2,ADCY8,DRD2,ERAS,GJB7,GJC2,MAPK3,NT5C,PLCB3,PLCD1,PRKACB,RALA,SGSM3,SMARCC2,SRC,TUBB4B
Assembly of RNA Polymerase II Complex	1.17	0/50 (0%)	6/50 (12%)	0/50 (0%)	44/50 (88%)	GTF2E1,POLR2C,POLR2D,POLR2F,POLR2I,TAF7L
Hepatic Cholestasis	1.17	0/184 (0%)	16/184 (9%)	0/184 (0%)	168/184 (91%)	ABCB1,ABCG8,ADCY2,ADCY8,CD70,FGFR4,IL17C,IL37,IRAK4,LTB,MAP4K2,MYD88,PRKACB,SLCO1B1,SREBF1,TGFB2
Aryl Hydrocarbon Receptor Signaling	1.15	0/143 (0%)	13/143 (9%)	0/143 (0%)	130/143 (91%)	ALDH16A1,ALDH4A1,CYP1A1,CYP1A2,GSTP1,GSTT2/GSTT2B,GSTZ1,HSP90B1,HSPB7,MAPK3,SMARCA4,SRC,TGFB2

Dermatan Sulfate Degradation (Metazoa)	1.15	0/17 (0%)	3/17 (18%)	0/17 (0%)	14/17 (82%)	HYAL1,HYAL2,IDS
Superpathway of Geranylgeranyldiphosphate Biosynthesis I (via Mevalonate)	1.15	0/17 (0%)	3/17 (18%)	0/17 (0%)	14/17 (82%)	FNTB,GGPS1,IDI1
Thyroid Cancer Signaling	1.14	0/51 (0%)	6/51 (12%)	0/51 (0%)	45/51 (88%)	ERAS,MAPK3,NTRK3,RALA,RET,TCF7L2
Synaptic Long Term Depression	1.12	0/187 (0%)	16/187 (9%)	0/187 (0%)	171/187 (91%)	CACNA1E,CRHR1,ERAS,GRID1,MAPK3,NT5C,PLA2G12A,PLA2G5,PLCB3,PLCD1,PPP2CA,PPP2CB,PPP2R5E,RALA,RYR3,SMARCC2
Sphingosine-1-phosphate Signaling	1.12	0/117 (0%)	11/117 (9%)	0/117 (0%)	106/117 (91%)	ACER3,ADCY2,ADCY8,ASAH2B,MAPK3,PLCB3,PLCD1,RHOT1,S1PR4,SMPD3,SPHK1
Cellular Effects of Sildenafil (Viagra)	1.12	0/131 (0%)	12/131 (9%)	0/131 (0%)	119/131 (91%)	ACTG2,ADCY2,ADCY8,CACNA1E,GPR37,MYH11,MYL2,MYL4,PLCB3,PLCD1,PRKACB,SLC4A5
Purine Nucleotides Degradation II (Aerobic)	1.09	0/18 (0%)	3/18 (17%)	0/18 (0%)	15/18 (83%)	IMPDH1,NT5C,NT5C3A
NRF2-mediated Oxidative Stress Response	1.08	0/190 (0%)	16/190 (8%)	0/190 (0%)	174/190 (92%)	ACTG2,DNAJB13,DNAJB2,DNAJB9,DNAJC10,DNAJC21,DNAJC3,ERAS,ERP29,GSTP1,GSTT2/GSTT2B,GSTZ1,JUNB,MAPK3,RALA,SOD2
Mitotic Roles of Polo-Like Kinase	1.07	0/66 (0%)	7/66 (11%)	0/66 (0%)	59/66 (89%)	ANAPC13,ANAPC2,FZR1,HSP90B1,PPP2CA,PPP2CB,PPP2R5E
Sperm Motility	1.05	0/221 (0%)	18/221 (8%)	0/221 (0%)	203/221 (92%)	CNGA4,CSK,DDR1,EPHA5,EPHB3,ERBB2,FGFR4,MST1R,NPPB,NTRK3,PLA2G12A,PLA2G5,PLCB3,PLCD1,PRKACB,RET,SRC,TYK2
Role of BRCA1 in DNA Damage Response	1.04	0/80 (0%)	8/80 (10%)	0/80 (0%)	72/80 (90%)	BABAM1,E2F7,FAAP100,FANCC,MDC1,SLC19A1,SMARCA4,SMARCC2
IL-15 Production	1.04	0/121 (0%)	11/121 (9%)	0/121 (0%)	110/121 (91%)	CSK,DDR1,EPHA5,EPHB3,ERBB2,FGFR4,MST1R,NTRK3,RET,SRC,TYK2
Melanocyte Development and Pigmentation Signaling	1.03	0/94 (0%)	9/94 (10%)	0/94 (0%)	85/94 (90%)	ADCY2,ADCY8,CREB1,ERAS,MAPK3,PRKACB,RALA,SH2B2,SRC
Colorectal Cancer Metastasis Signaling	1.02	0/253 (0%)	20/253 (8%)	0/253 (0%)	233/253 (92%)	ADCY2,ADCY8,BAD,ERAS,LRP5,MAPK3,MMP15,MMP24,MMP26,PGF,PRKACB,RALA,RHOT1,SRC,TCF7L2,TGFB2,TLR6,TLR9,TYK2,WNT6
Serotonin Receptor Signaling	1	0/43 (0%)	5/43 (12%)	0/43 (0%)	38/43 (88%)	ADCY2,ADCY8,GCH1,HTR1D,SLC18A2
Remodeling of Epithelial Adherens Junctions	0.991	0/69 (0%)	7/69 (10%)	0/69 (0%)	62/69 (90%)	ACTG2,ARPC5,DNM3,MAPRE1,RALA,SRC,TUBB4B
Unfolded protein response	0.987	0/56 (0%)	6/56 (11%)	0/56 (0%)	50/56 (89%)	DNAJB9,DNAJC3,HSP90B1,HSPA1L,INSIG1,SREBF1
TGF-β Signaling	0.987	0/96 (0%)	9/96 (9%)	0/96 (0%)	87/96 (91%)	ERAS,HOXC9,INHBC,MAPK3,RALA,RUNX3,SMAD1,SMAD5,TGFB2
Xenobiotic Metabolism Signaling	0.967	0/287 (0%)	22/287 (8%)	0/287 (0%)	265/287 (92%)	ABCB1,ALDH16A1,ALDH4A1,CHST12,CYP1A1,CYP1A2,ERAS,FMO5,GSTP1,GSTT2/GSTT2B,GSTZ1,HS6ST3,HSP90B1,MAPK3,MGMT,PPP2CA,PPP2CB,PPP2R5E,RALA,SLCO1B1,SULT1A2,SULT1C4
Role of CHK Proteins in Cell Cycle Checkpoint Control	0.959	0/57 (0%)	6/57 (11%)	0/57 (0%)	51/57 (89%)	E2F7,MDC1,PPP2CA,PPP2CB,PPP2R5E,SLC19A1
FXR/RXR Activation	0.951	0/126 (0%)	11/126 (9%)	0/126 (0%)	115/126 (91%)	ABCG8,APOF,BAAT,CYP19A1,FGFR4,G6PC,IL37,SLC51A,SLCO1B1,SREBF1,TF
GDP-L-fucose Biosynthesis II (from L-fucose)	0.951	0/2 (0%)	1/2 (50%)	0/2 (0%)	1/2 (50%)	FUK

4-hydroxyproline Degradation I	0.951	0/2 (0%)	1/2 (50%)	0/2 (0%)	1/2 (50%)	ALDH4A1
PTEN Signaling	0.951	0/126 (0%)	11/126 (9%)	0/126 (0%)	115/126 (91%)	BAD,BCAR1,ERAS,FGFR4,FOXO4,FOXO6,INPP5D,MAPK3,MCRS1,NTRK3,RALA
Cancer Drug Resistance By Drug Efflux	0.932	0/58 (0%)	6/58 (10%)	0/58 (0%)	52/58 (90%)	ABCB1,ERAS,FOXO4,FOXO6,MAPK3,RALA
Systemic Lupus Erythematosus Signaling	0.928	0/230 (0%)	18/230 (8%)	0/230 (0%)	212/230 (92%)	CD247,CD3E,CD72,ERAS,FCGR2B,FCGR3A/FCGR3B,IL37,INPP5D,MAPK3,NFATC2,RALA,SF3B4,SNRNP35,SNRPD3,SNRPG,TLR9,TNFRSF13C,ZCR[illegible]1
Circadian Rhythm Signaling	0.921	0/33 (0%)	4/33 (12%)	0/33 (0%)	29/33 (88%)	CREB1,PER1,PER3,VIPR2
PDGF Signaling	0.91	0/86 (0%)	8/86 (9%)	0/86 (0%)	78/86 (91%)	ERAS,INPP5D,MAPK3,RALA,SPHK1,SPHK2,SRC,TYK2
Sirtuin Signaling Pathway	0.91	0/292 (0%)	22/292 (8%)	0/292 (0%)	270/292 (92%)	ACADL,ATG4B,ATP5F1D,ATP5F1E,ESRRA,FOXO4,GABPB1,HIST1H1E,HSF1,MAPK3,NDUFA3,NDUFA9,NDUFB10,POLR2F,SDHC,SDHD,SOD2,SREB[illegible]1,TIMM10,TIMM22,TIMM23B,UQCRFS1
White Adipose Tissue Browning Pathway	0.9	0/129 (0%)	11/129 (9%)	0/129 (0%)	118/129 (91%)	ADCY2,ADCY8,CACNA1E,CREB1,DIO2,FGFR4,HOXC9,LIPE,NPPB,NRF1,PRKACB
γ-glutamyl Cycle	0.886	0/11 (0%)	2/11 (18%)	0/11 (0%)	9/11 (82%)	GGT6,OPLAH
Neuroprotective Role of THOP1 in Alzheimer's Disease	0.876	0/116 (0%)	10/116 (9%)	0/116 (0%)	106/116 (91%)	CREB1,F11,GNRH2,HGFAC,HTRA3,PRKACB,PRSS21,PRSS27,PRSS33,PRSS36
Autophagy	0.857	0/61 (0%)	6/61 (10%)	0/61 (0%)	55/61 (90%)	ATG4B,LAMP2,RB1CC1,STX17,VPS33A,VPS41
Nucleotide Excision Repair Pathway	0.851	0/35 (0%)	4/35 (11%)	0/35 (0%)	31/35 (89%)	POLR2C,POLR2D,POLR2F,POLR2I
PI3K/AKT Signaling	0.851	0/132 (0%)	11/132 (8%)	0/132 (0%)	121/132 (92%)	BAD,ERAS,HSP90B1,INPP5D,MAPK3,PPP2CA,PPP2CB,PPP2R5E,RALA,RHEB,TYK2
Regulation of IL-2 Expression in Activated and Anergic T Lymphocytes	0.848	0/89 (0%)	8/89 (9%)	0/89 (0%)	81/89 (91%)	CD247,CD3E,ERAS,MAPK3,NFATC2,RALA,TGFB2,VAV2
Chondroitin Sulfate Biosynthesis (Late Stages)	0.848	0/48 (0%)	5/48 (10%)	0/48 (0%)	43/48 (90%)	CHST12,CSGALNACT2,HS6ST3,SULT1A2,SULT1C4
ERK/MAPK Signaling	0.827	0/193 (0%)	15/193 (8%)	0/193 (0%)	178/193 (92%)	BAD,BCAR1,CREB1,ERAS,HSPB7,LAMTOR3,MAPK3,PLA2G12A,PLA2G5,PPP2CA,PPP2CB,PPP2R5E,PRKACB,RALA,SRC
Assembly of RNA Polymerase I Complex	0.824	0/12 (0%)	2/12 (17%)	0/12 (0%)	10/12 (83%)	POLR2F,TAF1C
Guanosine Nucleotides Degradation III	0.824	0/12 (0%)	2/12 (17%)	0/12 (0%)	10/12 (83%)	NT5C,NT5C3A
LPS/IL-1 Mediated Inhibition of RXR Function	0.818	0/224 (0%)	17/224 (8%)	0/224 (0%)	207/224 (92%)	ABCB1,ABCG8,ALDH16A1,ALDH4A1,CHST12,FMO5,GSTP1,GSTT2/GSTT2B,GSTZ1,HS6ST3,IL37,MGMT,MYD88,SLC27A1,SREBF1,SULT1A2,SULT1C4
T Cell Receptor Signaling	0.815	0/105 (0%)	9/105 (9%)	0/105 (0%)	96/105 (91%)	CD247,CD3E,CSK,ERAS,MAPK3,NFATC2,RALA,RASGRP1,VAV2
Antiproliferative Role of Somatostatin Receptor 2	0.807	0/77 (0%)	7/77 (9%)	0/77 (0%)	70/77 (91%)	ERAS,MAPK3,NT5C,RALA,SMARCC2,SRC,SSTR2

mTOR Signaling	0.804	0/210 (0%)	16/210 (8%)	0/210 (0%)	194/210 (92%)	ARHGAP8/PRR5-ARHGAP8,EIF3J,ERAS,MAPK3,PGF,PLD5,PPP2CA,PPP2CB,PPP2R5E,PRR5L,RALA,RHEB,RHOT1,RPS17,RPS19,RPS4Y2
Glutathione Redox Reactions I	0.801	0/24 (0%)	3/24 (13%)	0/24 (0%)	21/24 (88%)	GSTP1,GSTT2/GSTT2B,GSTZ1
Phagosome Maturation	0.801	0/150 (0%)	12/150 (8%)	0/150 (0%)	138/150 (92%)	ATP6V0D2,DYNC1LI1,LAMP2,MPO,NAPB,NOX1,NOX3,PRDX2,RILP,TUBB4B,VPS33A,VPS41
Agrin Interactions at Neuromuscular Junction	0.788	0/78 (0%)	7/78 (9%)	0/78 (0%)	71/78 (91%)	ACTG2,ERAS,ERBB2,GABPB1,MAPK3,RALA,SRC
Cell Cycle Regulation by BTG Family Proteins	0.788	0/37 (0%)	4/37 (11%)	0/37 (0%)	33/37 (89%)	E2F7,PPP2CA,PPP2CB,PPP2R5E
Diphthamide Biosynthesis	0.788	0/3 (0%)	1/3 (33%)	0/3 (0%)	2/3 (67%)	DPH6
Methylglyoxal Degradation I	0.788	0/3 (0%)	1/3 (33%)	0/3 (0%)	2/3 (67%)	GLO1
Coenzyme A Biosynthesis	0.788	0/3 (0%)	1/3 (33%)	0/3 (0%)	2/3 (67%)	PPCS
Proline Degradation	0.788	0/3 (0%)	1/3 (33%)	0/3 (0%)	2/3 (67%)	ALDH4A1
Thyronamine and Iodothyronamine Metabolism	0.788	0/3 (0%)	1/3 (33%)	0/3 (0%)	2/3 (67%)	DIO2
Tetrahydrobiopterin Biosynthesis I	0.788	0/3 (0%)	1/3 (33%)	0/3 (0%)	2/3 (67%)	GCH1
Hypusine Biosynthesis	0.788	0/3 (0%)	1/3 (33%)	0/3 (0%)	2/3 (67%)	EIF5A
Anandamide Degradation	0.788	0/3 (0%)	1/3 (33%)	0/3 (0%)	2/3 (67%)	FAAH
Thyroid Hormone Metabolism I (via Deiodination)	0.788	0/3 (0%)	1/3 (33%)	0/3 (0%)	2/3 (67%)	DIO2
Tetrahydrobiopterin Biosynthesis II	0.788	0/3 (0%)	1/3 (33%)	0/3 (0%)	2/3 (67%)	GCH1
AMPK Signaling	0.78	0/212 (0%)	16/212 (8%)	0/212 (0%)	196/212 (92%)	ADRA1A,AK3,CHRNA4,CREB1,FOXO4,FOXO6,LIPE,PPM1M,PPP2CA,PPP2CB,PPP2R5E,PRKACB,RAB39A,SMARCA4,SMARCC2,SRC
Adrenomedullin signaling pathway	0.777	0/197 (0%)	15/197 (8%)	0/197 (0%)	182/197 (92%)	ADCY2,ADCY8,BAD,CSK,ERAS,GPR37,IL37,MAPK3,MYLK3,NT5C,PLCB3,PLCD1,PRKACB,RALA,SMARCC2
Factors Promoting Cardiogenesis in Vertebrates	0.772	0/93 (0%)	8/93 (9%)	0/93 (0%)	85/93 (91%)	BMP8A,LRP5,MYL2,NPPB,SMAD1,SMAD5,TCF7L2,TGFB2
UVC-Induced MAPK Signaling	0.77	0/51 (0%)	5/51 (10%)	0/51 (0%)	46/51 (90%)	ERAS,MAPK3,RALA,SMPD3,SRC
Bile Acid Biosynthesis, Neutral Pathway	0.767	0/13 (0%)	2/13 (15%)	0/13 (0%)	11/13 (85%)	AKR1C4,BAAT
Leukocyte Extravasation Signaling	0.764	0/198 (0%)	15/198 (8%)	0/198 (0%)	183/198 (92%)	ACTG2,ARHGAP8/PRR5-ARHGAP8,BCAR1,CLDN14,CLDN15,CLDN2,MMP15,MMP24,MMP26,NOX1,NOX3,RASGRP1,SPN,SRC,VAV2
CDK5 Signaling	0.764	0/108 (0%)	9/108 (8%)	0/108 (0%)	99/108 (92%)	ADCY2,ADCY8,ERAS,MAPK3,PPP2CA,PPP2CB,PPP2R5E,PRKACB,RALA
ErbB2-ErbB3 Signaling	0.764	0/65 (0%)	6/65 (9%)	0/65 (0%)	59/65 (91%)	BAD,ERAS,ERBB2,MAPK3,RALA,TYK2
Nicotine Degradation II	0.764	0/65 (0%)	6/65 (9%)	0/65 (0%)	59/65 (91%)	CYP19A1,CYP1A1,CYP1A2,CYP2D6,CYP4X1,FMO5

D-myo-inositol (1,4,5)-Trisphosphate Biosynthesis	0.762	0/25 (0%)	3/25 (12%)	0/25 (0%)	22/25 (88%)	CDIPT,PLCB3,PLCD1
PI3K Signaling in B Lymphocytes	0.759	0/138 (0%)	11/138 (8%)	0/138 (0%)	127/138 (92%)	CR2,CREB1,ERAS,FCGR2B,INPP5D,MAPK3,NFATC2,PLCB3,PLCD1,RALA,VAV2
Notch Signaling	0.757	0/38 (0%)	4/38 (11%)	0/38 (0%)	34/38 (89%)	APH1A,DLL3,HES5,MFNG
Fcγ Receptor-mediated Phagocytosis in Macrophages and Monocytes	0.754	0/94 (0%)	8/94 (9%)	0/94 (0%)	86/94 (91%)	ACTG2,ARPC5,FCGR3A/FCGR3B,INPP5D,MAPK3,PLD5,SRC,VAV2
Pancreatic Adenocarcinoma Signaling	0.75	0/109 (0%)	9/109 (8%)	0/109 (0%)	100/109 (92%)	BAD,E2F7,ERBB2,MAPK3,PGF,PLD5,RALA,TGFB2,TYK2
Chemokine Signaling	0.747	0/80 (0%)	7/80 (9%)	0/80 (0%)	73/80 (91%)	ERAS,MAPK3,MYL2,NOX1,PLCB3,RALA,SRC
Role of Pattern Recognition Receptors in Recognition of Bacteria and Viruses	0.745	0/154 (0%)	12/154 (8%)	0/154 (0%)	142/154 (92%)	CD70,CREB1,IL17C,IL37,LTB,MAPK3,MYD88,NLRC4,NOD1,TGFB2,TLR6,TLR9
Regulation of Cellular Mechanics by Calpain Protease	0.745	0/66 (0%)	6/66 (9%)	0/66 (0%)	60/66 (91%)	CAPN6,CNGA4,ERAS,MAPK3,RALA,SRC
Breast Cancer Regulation by Stathmin1	0.74	0/200 (0%)	15/200 (8%)	0/200 (0%)	185/200 (93%)	ADCY2,ADCY8,ARHGEF16,ARHGEF9,E2F7,ERAS,MAPK3,PLCB3,PPP2CA,PPP2CB,PPP2R5E,PRKACB,RALA,RB1CC1,TUBB4B
α-Adrenergic Signaling	0.738	0/95 (0%)	8/95 (8%)	0/95 (0%)	87/95 (92%)	ADCY2,ADCY8,ADRA1A,ERAS,MAPK3,PHKG2,PRKACB,RALA
FAK Signaling	0.738	0/95 (0%)	8/95 (8%)	0/95 (0%)	87/95 (92%)	ACTG2,BCAR1,CAPN6,CSK,ERAS,MAPK3,RALA,SRC
Phagosome Formation	0.73	0/125 (0%)	10/125 (8%)	0/125 (0%)	115/125 (92%)	CR2,FCAMR,FCGR2B,FCGR3A/FCGR3B,MARCKS,PLCB3,PLCD1,RHOT1,TLR6,TLR9
Cyclins and Cell Cycle Regulation	0.728	0/81 (0%)	7/81 (9%)	0/81 (0%)	74/81 (91%)	E2F7,HDAC10,PPP2CA,PPP2CB,PPP2R5E,SKP2,TGFB2
NAD Salvage Pathway II	0.726	0/26 (0%)	3/26 (12%)	0/26 (0%)	23/26 (88%)	NMNAT2,NT5C,NT5C3A
Neuregulin Signaling	0.721	0/96 (0%)	8/96 (8%)	0/96 (0%)	88/96 (92%)	BAD,ERAS,ERBB2,GRB7,HSP90B1,MAPK3,RALA,SRC
Axonal Guidance Signaling	0.721	0/486 (0%)	33/486 (7%)	0/486 (0%)	453/486 (93%)	ADAM8,ADAMTS14,ADAMTS15,ARPC5,BCAR1,BMP8A,EPHA5,EPHB3,ERAP2,ERAS,ERBB2,KEL,MAPK3,MMP15,MMP24,MMP26,MYL2,MYL4,NFATC2,NTN3,NTRK3,PGF,PLCB3,PLCD1,PRKACB,RALA,SEMA3F,SEMA3G,SEMA5A,STK36,TUBB4B,UNC5D,WNT6
Role of Macrophages, Fibroblasts and Endothelial Cells in Rheumatoid Arthritis	0.712	0/312 (0%)	22/312 (7%)	0/312 (0%)	290/312 (93%)	CREB1,DKK2,ERAS,FCGR3A/FCGR3B,IL37,IRAK4,LRP5,LTB,MAPK3,MYD88,NFATC2,PGF,PLCB3,PLCD1,RALA,SRC,TCF7L2,TLR6,TLR9,TRAF1,TRAF5,WNT6
Bladder Cancer Signaling	0.706	0/97 (0%)	8/97 (8%)	0/97 (0%)	89/97 (92%)	ERAS,ERBB2,MAPK3,MMP15,MMP24,MMP26,PGF,RALA
B Cell Activating Factor Signaling	0.678	0/41 (0%)	4/41 (10%)	0/41 (0%)	37/41 (90%)	NFATC2,TNFRSF13C,TRAF1,TRAF5
p70S6K Signaling	0.674	0/129 (0%)	10/129 (8%)	0/129 (0%)	119/129 (92%)	BAD,ERAS,MAPK3,PLCB3,PLCD1,PPP2CA,PPP2CB,PPP2R5E,RALA,SRC
Pentose Phosphate Pathway (Oxidative Branch)	0.674	0/4 (0%)	1/4 (25%)	0/4 (0%)	3/4 (75%)	PGLS

Spermine and Spermidine Degradation I	0.674	0/4 (0%)	1/4 (25%)	0/4 (0%)	3/4 (75%)	SAT2
Arginine Degradation I (Arginase Pathway)	0.674	0/4 (0%)	1/4 (25%)	0/4 (0%)	3/4 (75%)	ALDH4A1
Adenosine Nucleotides Degradation II	0.67	0/15 (0%)	2/15 (13%)	0/15 (0%)	13/15 (87%)	NT5C,NT5C3A
CREB Signaling in Neurons	0.664	0/207 (0%)	15/207 (7%)	0/207 (0%)	192/207 (93%)	ADCY2,ADCY8,CACNA1E,CREB1,ERAS,GRID1,MAPK3,PLCB3,PLCD1,POLR2C,POLR2D,POLR2F,POLR2I,PRKACB,RALA
Superpathway of Cholesterol Biosynthesis	0.662	0/28 (0%)	3/28 (11%)	0/28 (0%)	25/28 (89%)	EBP,GGPS1,IDI1
VEGF Signaling	0.658	0/100 (0%)	8/100 (8%)	0/100 (0%)	92/100 (92%)	ACTG2,BAD,EIF1AX,ERAS,MAPK3,PGF,RALA,SRC
Nicotine Degradation III	0.656	0/56 (0%)	5/56 (9%)	0/56 (0%)	51/56 (91%)	CYP19A1,CYP1A1,CYP1A2,CYP2D6,CYP4X1
Chondroitin Sulfate Biosynthesis	0.656	0/56 (0%)	5/56 (9%)	0/56 (0%)	51/56 (91%)	CHST12,CSGALNACT2,HS6ST3,SULT1A2,SULT1C4
Protein Kinase A Signaling	0.652	0/399 (0%)	27/399 (7%)	0/399 (0%)	372/399 (93%)	ADCY2,ADCY8,AKAP14,ANAPC13,ANAPC2,BAD,CNGA4,CREB1,DUSP18,EYA2,HIST1H1E,LIPE,MAPK3,MYL2,MYL4,MYLK3,NFATC2,PGP,PHKG2,PLCB3,PLCD1,PRKACB,PTPRN,PTPRS,RYR3,TCF7L2,TGFB2
Dopamine-DARPP32 Feedback in cAMP Signaling	0.633	0/163 (0%)	12/163 (7%)	0/163 (0%)	151/163 (93%)	ADCY2,ADCY8,ATP2A1,CACNA1E,CREB1,DRD2,PLCB3,PLCD1,PPP2CA,PPP2CB,PPP2R5E,PRKACB
Oncostatin M Signaling	0.629	0/43 (0%)	4/43 (9%)	0/43 (0%)	39/43 (91%)	ERAS,MAPK3,RALA,TYK2
Chondroitin Sulfate Degradation (Metazoa)	0.629	0/16 (0%)	2/16 (13%)	0/16 (0%)	14/16 (88%)	HYAL1,HYAL2
Melatonin Signaling	0.627	0/72 (0%)	6/72 (8%)	0/72 (0%)	66/72 (92%)	GNRH2,MAPK3,MTNR1A,PLCB3,PLCD1,PRKACB
NER Pathway	0.613	0/103 (0%)	8/103 (8%)	0/103 (0%)	95/103 (92%)	HIST1H4D,LIG4,POLD4,POLR2C,POLR2D,POLR2F,POLR2I,SLC19A1
Dopamine Degradation	0.604	0/30 (0%)	3/30 (10%)	0/30 (0%)	27/30 (90%)	ALDH4A1,SULT1A2,SULT1C4
Human Embryonic Stem Cell Pluripotency	0.595	0/135 (0%)	10/135 (7%)	0/135 (0%)	125/135 (93%)	BMP8A,FGFR4,NTRK3,S1PR4,SMAD1,SMAD5,SPHK1,TCF7L2,TGFB2,WNT6
Dermatan Sulfate Biosynthesis	0.595	0/59 (0%)	5/59 (8%)	0/59 (0%)	54/59 (92%)	CHST12,CSGALNACT2,HS6ST3,SULT1A2,SULT1C4
Leptin Signaling in Obesity	0.593	0/74 (0%)	6/74 (8%)	0/74 (0%)	68/74 (92%)	ADCY2,ADCY8,MAPK3,PLCB3,PLCD1,PRKACB
Ubiquinol-10 Biosynthesis (Eukaryotic)	0.59	0/17 (0%)	2/17 (12%)	0/17 (0%)	15/17 (88%)	CYP4F11,ECHDC1
Eumelanin Biosynthesis	0.59	0/5 (0%)	1/5 (20%)	0/5 (0%)	4/5 (80%)	DDT
Lysine Degradation II	0.59	0/5 (0%)	1/5 (20%)	0/5 (0%)	4/5 (80%)	AASDHPPT
Myo-inositol Biosynthesis	0.59	0/5 (0%)	1/5 (20%)	0/5 (0%)	4/5 (80%)	ISYNA1
Lysine Degradation V	0.59	0/5 (0%)	1/5 (20%)	0/5 (0%)	4/5 (80%)	AASDHPPT
CXCR4 Signaling	0.588	0/167 (0%)	12/167 (7%)	0/167 (0%)	155/167 (93%)	ADCY2,ADCY8,BCAR1,ELMO1,ERAS,MAPK3,MYL2,MYL4,PLCB3,RALA,RHOT1,SRC
IL-8 Signaling	0.583	0/199 (0%)	14/199 (7%)	0/199 (0%)	185/199 (93%)	CR2,ERAS,IRAK4,MAPK3,MPO,MYL2,NOX1,NOX3,PGF,PLD5,RAB11FIP2,RALA,RHOT1,SRC

Androgen Signaling	0.583	0/136 (0%)	10/136 (7%)	0/136 (0%)	126/136 (93%)	CACNA1E,GTF2E1,MAPK3,POLR2C,POLR2D,POLR2F,POLR2I,PRKACB,SHBG,SRC
Endometrial Cancer Signaling	0.577	0/60 (0%)	5/60 (8%)	0/60 (0%)	55/60 (92%)	BAD,ERAS,ERBB2,MAPK3,RALA
Iron homeostasis signaling pathway	0.572	0/137 (0%)	10/137 (7%)	0/137 (0%)	127/137 (93%)	ATP6V0D2,BMP8A,HBQ1,MAPK3,SKP2,SLC11A2,SMAD1,SMAD5,TF,TYK2
Prostate Cancer Signaling	0.565	0/91 (0%)	7/91 (8%)	0/91 (0%)	84/91 (92%)	BAD,CREB1,ERAS,GSTP1,HSP90B1,MAPK3,RALA
Dermatan Sulfate Biosynthesis (Late Stages)	0.562	0/46 (0%)	4/46 (9%)	0/46 (0%)	42/46 (91%)	CHST12,HS6ST3,SULT1A2,SULT1C4
PFKFB4 Signaling Pathway	0.562	0/46 (0%)	4/46 (9%)	0/46 (0%)	42/46 (91%)	CREB1,MAPK3,PRKACB,TGFB2
Heparan Sulfate Biosynthesis (Late Stages)	0.561	0/76 (0%)	6/76 (8%)	0/76 (0%)	70/76 (92%)	CHST12,DTD2,HS6ST3,LIPE,SULT1A2,SULT1C4
Toll-like Receptor Signaling	0.561	0/76 (0%)	6/76 (8%)	0/76 (0%)	70/76 (92%)	IL37,IRAK4,MYD88,TLR6,TLR9,TRAF1
Phospholipases	0.559	0/61 (0%)	5/61 (8%)	0/61 (0%)	56/61 (92%)	PLA2G12A,PLA2G5,PLCB3,PLCD1,PLD5
Telomerase Signaling	0.558	0/107 (0%)	8/107 (7%)	0/107 (0%)	99/107 (93%)	ERAS,HDAC10,HSP90B1,MAPK3,PPP2CA,PPP2CB,PPP2R5E,RALA
1D-myo-inositol Hexakisphosphate Biosynthesis II (Mammalian)	0.554	0/18 (0%)	2/18 (11%)	0/18 (0%)	16/18 (89%)	INPP5D,ITPKC
D-myo-inositol (1,3,4)-trisphosphate Biosynthesis	0.554	0/18 (0%)	2/18 (11%)	0/18 (0%)	16/18 (89%)	INPP5D,ITPKC
4-1BB Signaling in T Lymphocytes	0.551	0/32 (0%)	3/32 (9%)	0/32 (0%)	29/32 (91%)	MAPK3,TNFRSF9,TRAF1
Glutathione-mediated Detoxification	0.551	0/32 (0%)	3/32 (9%)	0/32 (0%)	29/32 (91%)	GSTP1,GSTT2/GSTT2B,GSTZ1
Role of Osteoblasts, Osteoclasts and Chondrocytes in Rheumatoid Arthritis	0.538	0/220 (0%)	15/220 (7%)	0/220 (0%)	205/220 (93%)	BAD,BMP8A,CALCR,DKK2,DLX5,IL37,LRP5,MAPK3,NFATC2,SMAD1,SMAD5,SRC,TCF7L2,TRAF5,WNT6
Regulation of eIF4 and p70S6K Signaling	0.526	0/157 (0%)	11/157 (7%)	0/157 (0%)	146/157 (93%)	EIF1AX,EIF3J,ERAS,MAPK3,PPP2CA,PPP2CB,PPP2R5E,RALA,RPS17,RPS19,RPS4Y2
Wnt/β-catenin Signaling	0.526	0/173 (0%)	12/173 (7%)	0/173 (0%)	161/173 (93%)	DKK2,KREMEN1,LRP5,PPP2CA,PPP2CB,PPP2R5E,SOX7,SRC,TCF7L2,TGFB2,TLE1,WNT6
Chondroitin and Dermatan Biosynthesis	0.523	0/6 (0%)	1/6 (17%)	0/6 (0%)	5/6 (83%)	CSGALNACT2
Serotonin and Melatonin Biosynthesis	0.523	0/6 (0%)	1/6 (17%)	0/6 (0%)	5/6 (83%)	AANAT
NAD Biosynthesis III	0.523	0/6 (0%)	1/6 (17%)	0/6 (0%)	5/6 (83%)	NMNAT2
GDP-mannose Biosynthesis	0.523	0/6 (0%)	1/6 (17%)	0/6 (0%)	5/6 (83%)	MPI
Glucocorticoid Receptor Signaling	0.52	0/336 (0%)	22/336 (7%)	0/336 (0%)	314/336 (93%)	CD247,CD3E,CREB1,ERAS,GTF2E1,HSP90B1,HSPA1L,KRT23,KRT80,MAPK3,NFATC2,POLR2C,POLR2D,POLR2F,POLR2I,PRKACB,RALA,SLPI,SMARCA4,SMARCC2,TAF7L,TGFB2

PPARα/RXRα Activation	0.517	0/190 (0%)	13/190 (7%)	0/190 (0%)	177/190 (93%)	ACADL,ADCY2,ADCY8,ERAS,HELZ2,HSP90B1,MAPK3,PLCB3,PLCD1,PRKACB,RALA,SLC27A1,TGFB2
14-3-3-mediated Signaling	0.506	0/127 (0%)	9/127 (7%)	0/127 (0%)	118/127 (93%)	BAD,ERAS,GFAP,MAPK3,PLCB3,PLCD1,RALA,SRC,TUBB4B
P2Y Purigenic Receptor Signaling Pathway	0.506	0/127 (0%)	9/127 (7%)	0/127 (0%)	118/127 (93%)	ADCY2,ADCY8,CREB1,ERAS,MAPK3,PLCB3,PLCD1,PRKACB,RALA
eNOS Signaling	0.506	0/159 (0%)	11/159 (7%)	0/159 (0%)	148/159 (93%)	ADCY2,ADCY8,AQP10,AQP6,CHRNA4,CNGA4,HSP90B1,HSPA1L,LPAR2,PGF,PRKACB
MIF-mediated Glucocorticoid Regulation	0.504	0/34 (0%)	3/34 (9%)	0/34 (0%)	31/34 (91%)	MAPK3,PLA2G12A,PLA2G5
Endocannabinoid Cancer Inhibition Pathway	0.504	0/143 (0%)	10/143 (7%)	0/143 (0%)	133/143 (93%)	ADCY2,ADCY8,BAD,CREB1,MAPK3,PGF,PRKACB,SMPD3,SRC,TCF7L2
Thrombin Signaling	0.5	0/208 (0%)	14/208 (7%)	0/208 (0%)	194/208 (93%)	ADCY2,ADCY8,ARHGEF16,ARHGEF9,CREB1,ERAS,MAPK3,MYL2,MYL4,PLCB3,PLCD1,RALA,RHOT1,SRC
Netrin Signaling	0.492	0/65 (0%)	5/65 (8%)	0/65 (0%)	60/65 (92%)	CACNA1E,NFATC2,PRKACB,RYR3,UNC5D
Cardiomyocyte Differentiation via BMP Receptors	0.491	0/20 (0%)	2/20 (10%)	0/20 (0%)	18/20 (90%)	MYL2,NPPB
CDP-diacylglycerol Biosynthesis I	0.491	0/20 (0%)	2/20 (10%)	0/20 (0%)	18/20 (90%)	LCLAT1,TAZ
Fatty Acid α-oxidation	0.491	0/20 (0%)	2/20 (10%)	0/20 (0%)	18/20 (90%)	ALDH4A1,ALOXE3
Melanoma Signaling	0.485	0/50 (0%)	4/50 (8%)	0/50 (0%)	46/50 (92%)	BAD,ERAS,MAPK3,RALA
Corticotropin Releasing Hormone Signaling	0.484	0/145 (0%)	10/145 (7%)	0/145 (0%)	135/145 (93%)	ADCY2,ADCY8,ARPC5,CACNA1E,CREB1,CRHR1,MAPK3,NT5C,PRKACB,SMARCC2
ATM Signaling	0.484	0/97 (0%)	7/97 (7%)	0/97 (0%)	90/97 (93%)	CBX5,CREB1,H2AFX,MDC1,PPP2CA,PPP2CB,PPP2R5E
PAK Signaling	0.484	0/97 (0%)	7/97 (7%)	0/97 (0%)	90/97 (93%)	EPHB3,ERAS,MAPK3,MYL2,MYL4,PAK1IP1,RALA
HIF1α Signaling	0.483	0/113 (0%)	8/113 (7%)	0/113 (0%)	105/113 (93%)	ELOB,ERAS,MAPK3,MMP15,MMP24,MMP26,PGF,RALA
RAR Activation	0.48	0/194 (0%)	13/194 (7%)	0/194 (0%)	181/194 (93%)	ADCY2,ADCY8,AKR1C4,CSK,NRIP2,PRKACB,RDH8,SMAD1,SMAD5,SMARCA4,SMARCC2,SRC,TGFB2
Calcium-induced T Lymphocyte Apoptosis	0.478	0/66 (0%)	5/66 (8%)	0/66 (0%)	61/66 (92%)	ATP2A1,CD247,CD3E,NFATC2,ORAI1
Molecular Mechanisms of Cancer	0.478	0/392 (0%)	25/392 (6%)	0/392 (0%)	367/392 (94%)	ADCY2,ADCY8,APH1A,ARHGEF16,ARHGEF9,BAD,BMP8A,CDK20,E2F7,ERAS,LAMTOR3,LRP5,MAPK3,PLCB3,PRKACB,RALA,RASGRP1,RHOT1,SMAD1,SMAD5,SRC,STK36,TGFB2,TYK2,WNT6
UVA-Induced MAPK Signaling	0.472	0/98 (0%)	7/98 (7%)	0/98 (0%)	91/98 (93%)	ERAS,MAPK3,PARP2,PLCB3,PLCD1,RALA,SMPD3
Amyloid Processing	0.469	0/51 (0%)	4/51 (8%)	0/51 (0%)	47/51 (92%)	APH1A,CAPN6,MAPK3,PRKACB
NAD Biosynthesis from 2-amino-3-carboxymuconate Semialdehyde	0.467	0/7 (0%)	1/7 (14%)	0/7 (0%)	6/7 (86%)	NMNAT2
NAD Salvage Pathway III	0.467	0/7 (0%)	1/7 (14%)	0/7 (0%)	6/7 (86%)	NMNAT2
Putrescine Degradation III	0.463	0/21 (0%)	2/21 (10%)	0/21 (0%)	19/21 (90%)	ALDH4A1,SAT2

Endoplasmic Reticulum Stress Pathway	0.463	0/21 (0%)	2/21 (10%)	0/21 (0%)	19/21 (90%)	DNAJC3,HSP90B1
Cell Cycle: G1/S Checkpoint Regulation	0.462	0/67 (0%)	5/67 (7%)	0/67 (0%)	62/67 (93%)	E2F7,HDAC10,PAK1IP1,SKP2,TGFB2
Endocannabinoid Developing Neuron Pathway	0.461	0/115 (0%)	8/115 (7%)	0/115 (0%)	107/115 (93%)	ADCY2,ADCY8,CREB1,ERAS,MAPK3,PRKACB,RALA,SRC
FLT3 Signaling in Hematopoietic Progenitor Cells	0.46	0/83 (0%)	6/83 (7%)	0/83 (0%)	77/83 (93%)	BAD,CREB1,ERAS,INPP5D,MAPK3,RALA
Heparan Sulfate Biosynthesis	0.46	0/83 (0%)	6/83 (7%)	0/83 (0%)	77/83 (93%)	CHST12,DTD2,HS6ST3,LIPE,SULT1A2,SULT1C4
Triacylglycerol Degradation	0.452	0/52 (0%)	4/52 (8%)	0/52 (0%)	48/52 (92%)	ABHD16A,DTD2,FAAH,LIPE
fMLP Signaling in Neutrophils	0.45	0/116 (0%)	8/116 (7%)	0/116 (0%)	108/116 (93%)	ARPC5,ERAS,MAPK3,NFATC2,NOX1,NOX3,PLCB3,RALA
VEGF Family Ligand-Receptor Interactions	0.447	0/84 (0%)	6/84 (7%)	0/84 (0%)	78/84 (93%)	ERAS,MAPK3,PGF,PLA2G12A,PLA2G5,RALA
Superpathway of Inositol Phosphate Compounds	0.447	0/198 (0%)	13/198 (7%)	0/198 (0%)	185/198 (93%)	CDIPT,EPHX2,EYA4,HACD2,INPP5D,ITPKC,NT5C,PLCB3,PLCD1,PPFIA2,PPP2R5E,PPP4C,PTPRN
Neuropathic Pain Signaling In Dorsal Horn Neurons	0.437	0/101 (0%)	7/101 (7%)	0/101 (0%)	94/101 (93%)	CREB1,GPR37,MAPK3,PLCB3,PLCD1,PRKACB,SRC
Phosphatidylglycerol Biosynthesis II (Non-plastidic)	0.437	0/22 (0%)	2/22 (9%)	0/22 (0%)	20/22 (91%)	LCLAT1,TAZ
Methionine Degradation I (to Homocysteine)	0.437	0/22 (0%)	2/22 (9%)	0/22 (0%)	20/22 (91%)	AHCY,MGMT
Lymphotoxin β Receptor Signaling	0.435	0/53 (0%)	4/53 (8%)	0/53 (0%)	49/53 (92%)	LTB,MAPK3,TRAF1,TRAF5
Dendritic Cell Maturation	0.435	0/183 (0%)	12/183 (7%)	0/183 (0%)	171/183 (93%)	CD58,CREB1,FCGR2B,FCGR3A/FCGR3B,IL37,IRF8,LTB,MAPK3,MYD88,PLCB3,PLCD1,TLR9
Neuroinflammation Signaling Pathway	0.429	0/300 (0%)	19/300 (6%)	0/300 (0%)	281/300 (94%)	APH1A,CREB1,CRP,GABRB1,IRAK4,MAPK3,MYD88,NFATC2,NOX1,NOX3,PLA2G12A,PLA2G5,PYCARD,SLC6A12,SOD2,TGFB2,TLR6,TLR9,TYK2
Thyroid Hormone Metabolism II (via Conjugation and/or Degradation)	0.424	0/38 (0%)	3/38 (8%)	0/38 (0%)	35/38 (92%)	DIO2,SULT1A2,SULT1C4
STAT3 Pathway	0.423	0/135 (0%)	9/135 (7%)	0/135 (0%)	126/135 (93%)	ERAS,FGFR4,IL11RA,MAPK3,NTRK3,RALA,SRC,TGFB2,TYK2
Superoxide Radicals Degradation	0.421	0/8 (0%)	1/8 (13%)	0/8 (0%)	7/8 (88%)	SOD2
Sphingomyelin Metabolism	0.421	0/8 (0%)	1/8 (13%)	0/8 (0%)	7/8 (88%)	SMPD3
Transcriptional Regulatory Network in Embryonic Stem Cells	0.42	0/54 (0%)	4/54 (7%)	0/54 (0%)	50/54 (93%)	HIST1H4D,LHX5,OTX1,SIX3
Cholecystokinin/Gastrin-mediated Signaling	0.418	0/119 (0%)	8/119 (7%)	0/119 (0%)	111/119 (93%)	BCAR1,ERAS,IL37,MAPK3,PLCB3,RALA,RHOT1,SRC

Role of NANOG in Mammalian Embryonic Stem Cell Pluripotency	0.418	0/119 (0%)	8/119 (7%)	0/119 (0%)	111/119 (93%)	BMP8A,ERAS,MAPK3,RALA,SMAD1,SMAD5,TYK2,WNT6
Chronic Myeloid Leukemia Signaling	0.415	0/103 (0%)	7/103 (7%)	0/103 (0%)	96/103 (93%)	BAD,E2F7,ERAS,HDAC10,MAPK3,RALA,TGFB2
Mouse Embryonic Stem Cell Pluripotency	0.415	0/103 (0%)	7/103 (7%)	0/103 (0%)	96/103 (93%)	ERAS,MAPK3,RALA,SMAD1,SMAD5,TCF7L2,TYK2
Superpathway of D-myo-inositol (1,4,5)-trisphosphate Metabolism	0.412	0/23 (0%)	2/23 (9%)	0/23 (0%)	21/23 (91%)	INPP5D,ITPKC
Apelin Cardiac Fibroblast Signaling Pathway	0.412	0/23 (0%)	2/23 (9%)	0/23 (0%)	21/23 (91%)	SPHK1,TGFB2
Sertoli Cell-Sertoli Cell Junction Signaling	0.41	0/186 (0%)	12/186 (6%)	0/186 (0%)	174/186 (94%)	ACTG2,BCAR1,CLDN14,CLDN15,CLDN2,ERAS,MAPK3,NECTIN2,PRKACB,RALA,SRC,TUBB4B
April Mediated Signaling	0.406	0/39 (0%)	3/39 (8%)	0/39 (0%)	36/39 (92%)	NFATC2,TRAF1,TRAF5
Inhibition of Matrix Metalloproteases	0.406	0/39 (0%)	3/39 (8%)	0/39 (0%)	36/39 (92%)	MMP15,MMP24,MMP26
Huntington's Disease Signaling	0.403	0/237 (0%)	15/237 (6%)	0/237 (0%)	222/237 (94%)	ATP5F1D,ATP5F1E,CAPN6,CREB1,DNM3,HDAC10,HSPA1L,MAPK3,NAPB,PLCB3,POLR2C,POLR2D,POLR2F,POLR2I,UBE2S
ERK5 Signaling	0.395	0/72 (0%)	5/72 (7%)	0/72 (0%)	67/72 (93%)	BAD,CREB1,ERAS,RALA,SRC
IL-22 Signaling	0.39	0/24 (0%)	2/24 (8%)	0/24 (0%)	22/24 (92%)	MAPK3,TYK2
TCA Cycle II (Eukaryotic)	0.39	0/24 (0%)	2/24 (8%)	0/24 (0%)	22/24 (92%)	SDHC,SDHD
Cysteine Biosynthesis III (mammalia)	0.39	0/24 (0%)	2/24 (8%)	0/24 (0%)	22/24 (92%)	AHCY,MGMT
CTLA4 Signaling in Cytotoxic T Lymphocytes	0.388	0/89 (0%)	6/89 (7%)	0/89 (0%)	83/89 (93%)	AP1S1,CD247,CD3E,PPP2CA,PPP2CB,PPP2R5E
Insulin Receptor Signaling	0.386	0/139 (0%)	9/139 (6%)	0/139 (0%)	130/139 (94%)	BAD,ERAS,FOXO4,INPP5D,LIPE,MAPK3,PRKACB,RALA,STXBP4
Calcium Signaling	0.385	0/206 (0%)	13/206 (6%)	0/206 (0%)	193/206 (94%)	ATP2A1,CACNA1E,CHRNA4,CREB1,HDAC10,MAPK3,MYH11,MYL2,MYL4,NFATC2,PRKACB,RYR3,TRDN
Cardiac Hypertrophy Signaling	0.383	0/240 (0%)	15/240 (6%)	0/240 (0%)	225/240 (94%)	ADCY2,ADCY8,ADRA1A,CACNA1E,CREB1,ERAS,MAPK3,MYL2,MYL4,PLCB3,PLCD1,PRKACB,RALA,RHOT1,TGFB2
Non-Small Cell Lung Cancer Signaling	0.383	0/73 (0%)	5/73 (7%)	0/73 (0%)	68/73 (93%)	BAD,ERAS,ERBB2,MAPK3,RALA
D-myo-inositol-5-phosphate Metabolism	0.383	0/156 (0%)	10/156 (6%)	0/156 (0%)	146/156 (94%)	EPHX2,EYA4,HACD2,NT5C,PLCB3,PLCD1,PPFIA2,PPP2R5E,PPP4C,PTPRN
GPCR-Mediated Integration of Enteroendocrine Signaling Exemplified by an L Cell	0.383	0/73 (0%)	5/73 (7%)	0/73 (0%)	68/73 (93%)	ADCY2,ADCY8,PLCB3,PLCD1,PRKACB
Sucrose Degradation V (Mammalian)	0.382	0/9 (0%)	1/9 (11%)	0/9 (0%)	8/9 (89%)	ALDOA
Leucine Degradation I	0.382	0/9 (0%)	1/9 (11%)	0/9 (0%)	8/9 (89%)	BCAT1

GNRH Signaling	0.381	0/173 (0%)	11/173 (6%)	0/173 (0%)	162/173 (94%)	ADCY2,ADCY8,CACNA1E,CREB1,ERAS,GNRH2,MAPK3,PLCB3,PRKACB,RALA,SRC
RhoA Signaling	0.38	0/123 (0%)	8/123 (7%)	0/123 (0%)	115/123 (93%)	ACTG2,ARHGAP8/PRR5-ARHGAP8,ARPC5,LPAR2,MYL2,MYL4,MYLK3,SEMA3F
EIF2 Signaling	0.377	0/224 (0%)	14/224 (6%)	0/224 (0%)	210/224 (94%)	ACTG2,EIF1AX,EIF3J,ERAS,MAPK3,RALA,RPL13,RPL36,RPLP1,RPLP2,RPS17,RPS19,RPS4Y2,SREBF1
Altered T Cell and B Cell Signaling in Rheumatoid Arthritis	0.377	0/90 (0%)	6/90 (7%)	0/90 (0%)	84/90 (93%)	CXCL13,IL37,LTB,TLR6,TLR9,TNFRSF13C
Cardiac β-adrenergic Signaling	0.377	0/140 (0%)	9/140 (6%)	0/140 (0%)	131/140 (94%)	ADCY2,ADCY8,AKAP14,ATP2A1,CACNA1E,PPP2CA,PPP2CB,PPP2R5E,PRKACB
ILK Signaling	0.373	0/191 (0%)	12/191 (6%)	0/191 (0%)	179/191 (94%)	ACTG2,CREB1,MAPK3,MYH11,MYL2,MYL4,PGF,PPP2CA,PPP2CB,PPP2R5E,RHOT1,SNAI1
Hypoxia Signaling in the Cardiovascular System	0.371	0/74 (0%)	5/74 (7%)	0/74 (0%)	69/74 (93%)	CREB1,HSP90B1,UBE2A,UBE2S,UBE2V2
Role of JAK family kinases in IL-6-type Cytokine Signaling	0.369	0/25 (0%)	2/25 (8%)	0/25 (0%)	23/25 (92%)	MAPK3,TYK2
Agranulocyte Adhesion and Diapedesis	0.366	0/192 (0%)	12/192 (6%)	0/192 (0%)	180/192 (94%)	ACTG2,CLDN14,CLDN15,CLDN2,CXCL13,IL37,MMP15,MMP24,MMP26,MYH11,MYL2,MYL4
MSP-RON Signaling Pathway	0.364	0/58 (0%)	4/58 (7%)	0/58 (0%)	54/58 (93%)	ACTG2,F11,KLKB1,MST1R
IL-6 Signaling	0.362	0/125 (0%)	8/125 (6%)	0/125 (0%)	117/125 (94%)	ABCB1,CRP,CYP19A1,ERAS,HSPB7,IL37,MAPK3,RALA
FcγRIIB Signaling in B Lymphocytes	0.359	0/75 (0%)	5/75 (7%)	0/75 (0%)	70/75 (93%)	CACNA1E,ERAS,FCGR2B,INPP5D,RALA
Synaptogenesis Signaling Pathway	0.358	0/312 (0%)	19/312 (6%)	0/312 (0%)	293/312 (94%)	ADCY2,ADCY8,ARPC5,BAD,CREB1,EPHA5,EPHB3,ERAS,MAPK3,MARCKS,NAPB,PRKACB,RALA,RASGRP1,SNCB,SRC,STXBP4,SYT3,SYT8
MIF Regulation of Innate Immunity	0.357	0/42 (0%)	3/42 (7%)	0/42 (0%)	39/42 (93%)	MAPK3,PLA2G12A,PLA2G5
Paxillin Signaling	0.355	0/109 (0%)	7/109 (6%)	0/109 (0%)	102/109 (94%)	ACTG2,ARF1,BCAR1,CSK,ERAS,RALA,SRC
Antiproliferative Role of TOB in T Cell Signaling	0.349	0/26 (0%)	2/26 (8%)	0/26 (0%)	24/26 (92%)	SKP2,TGFB2
Embryonic Stem Cell Differentiation into Cardiac Lineages	0.349	0/10 (0%)	1/10 (10%)	0/10 (0%)	9/10 (90%)	SP4
Estrogen-mediated S-phase Entry	0.349	0/26 (0%)	2/26 (8%)	0/26 (0%)	24/26 (92%)	E2F7,SKP2
Calcium Transport I	0.349	0/10 (0%)	1/10 (10%)	0/10 (0%)	9/10 (90%)	ATP2A1
Pentose Phosphate Pathway	0.349	0/10 (0%)	1/10 (10%)	0/10 (0%)	9/10 (90%)	PGLS
Mineralocorticoid Biosynthesis	0.349	0/10 (0%)	1/10 (10%)	0/10 (0%)	9/10 (90%)	EBP
GDNF Family Ligand-Receptor Interactions	0.348	0/76 (0%)	5/76 (7%)	0/76 (0%)	71/76 (93%)	CREB1,ERAS,MAPK3,RALA,RET
Neurotrophin/TRK Signaling	0.348	0/76 (0%)	5/76 (7%)	0/76 (0%)	71/76 (93%)	CREB1,ERAS,MAPK3,NTRK3,RALA

CNTF Signaling	0.338	0/60 (0%)	4/60 (7%)	0/60 (0%)	56/60 (93%)	ERAS,MAPK3,RALA,TYK2
NF-κB Signaling	0.337	0/179 (0%)	11/179 (6%)	0/179 (0%)	168/179 (94%)	ERAS,FGFR4,IL37,IRAK4,MYD88,NTRK3,PRKACB,RALA,TLR6,TLR9,TRAF5
iCOS-iCOSL Signaling in T Helper Cells	0.336	0/111 (0%)	7/111 (6%)	0/111 (0%)	104/111 (94%)	BAD,CD247,CD3E,CSK,ICOSLG/LOC102723996,INPP5D,NFATC2
Endocannabinoid Neuronal Synapse Pathway	0.336	0/128 (0%)	8/128 (6%)	0/128 (0%)	120/128 (94%)	ADCY2,ADCY8,CACNA1E,FAAH,MAPK3,PLCB3,PLCD1,PRKACB
Integrin Signaling	0.332	0/214 (0%)	13/214 (6%)	0/214 (0%)	201/214 (94%)	ACTG2,ARF1,ARPC5,BCAR1,CAPN6,ERAS,GRB7,MAPK3,MYL2,MYLK3,RALA,RHOT1,SRC
Synaptic Long Term Potentiation	0.328	0/129 (0%)	8/129 (6%)	0/129 (0%)	121/129 (94%)	ADCY8,CREB1,ERAS,MAPK3,PLCB3,PLCD1,PRKACB,RALA
GABA Receptor Signaling	0.327	0/95 (0%)	6/95 (6%)	0/95 (0%)	89/95 (94%)	ADCY2,ADCY8,CACNA1E,GABRB1,GPR37,SLC6A12
Role of MAPK Signaling in the Pathogenesis of Influenza	0.326	0/78 (0%)	5/78 (6%)	0/78 (0%)	73/78 (94%)	ERAS,MAPK3,PLA2G12A,PLA2G5,RALA
Opioid Signaling Pathway	0.321	0/250 (0%)	15/250 (6%)	0/250 (0%)	235/250 (94%)	ADCY2,ADCY8,BAD,CACNA1E,CREB1,ERAS,GRK4,MAPK3,NPBWR1,OPRL1,PRKACB,RALA,RYR3,SCN7A,SRC
Gα12/13 Signaling	0.32	0/130 (0%)	8/130 (6%)	0/130 (0%)	122/130 (94%)	ERAS,LPAR2,MAPK3,MYL2,MYL4,RALA,SRC,VAV2
Purine Nucleotides De Novo Biosynthesis II	0.319	0/11 (0%)	1/11 (9%)	0/11 (0%)	10/11 (91%)	IMPDH1
Glucocorticoid Biosynthesis	0.319	0/11 (0%)	1/11 (9%)	0/11 (0%)	10/11 (91%)	EBP
IL-3 Signaling	0.316	0/79 (0%)	5/79 (6%)	0/79 (0%)	74/79 (94%)	BAD,ERAS,INPP5D,MAPK3,RALA
Wnt/Ca+ pathway	0.315	0/62 (0%)	4/62 (6%)	0/62 (0%)	58/62 (94%)	CREB1,NFATC2,PLCB3,PLCD1
iNOS Signaling	0.313	0/45 (0%)	3/45 (7%)	0/45 (0%)	42/45 (93%)	IRAK4,MYD88,TYK2
Eicosanoid Signaling	0.293	0/64 (0%)	4/64 (6%)	0/64 (0%)	60/64 (94%)	CYSLTR1,LTB4R,PLA2G12A,PLA2G5
Cleavage and Polyadenylation of Pre-mRNA	0.292	0/12 (0%)	1/12 (8%)	0/12 (0%)	11/12 (92%)	CPSF1
BER pathway	0.292	0/12 (0%)	1/12 (8%)	0/12 (0%)	11/12 (92%)	POLB
Endothelin-1 Signaling	0.291	0/186 (0%)	11/186 (6%)	0/186 (0%)	175/186 (94%)	ADCY2,ADCY8,ERAS,MAPK3,PLA2G12A,PLA2G5,PLCB3,PLCD1,PLD5,RALA,SRC
Nitric Oxide Signaling in the Cardiovascular System	0.291	0/99 (0%)	6/99 (6%)	0/99 (0%)	93/99 (94%)	ATP2A1,CACNA1E,HSP90B1,MAPK3,PGF,PRKACB
Apoptosis Signaling	0.291	0/99 (0%)	6/99 (6%)	0/99 (0%)	93/99 (94%)	BAD,CAPN6,ENDOG,ERAS,MAPK3,RALA
Apelin Cardiomyocyte Signaling Pathway	0.291	0/99 (0%)	6/99 (6%)	0/99 (0%)	93/99 (94%)	ATP2A1,MAPK3,MYL2,MYL4,PLCB3,PLCD1
Ephrin A Signaling	0.288	0/47 (0%)	3/47 (6%)	0/47 (0%)	44/47 (94%)	BCAR1,EPHA5,VAV2
PEDF Signaling	0.287	0/82 (0%)	5/82 (6%)	0/82 (0%)	77/82 (94%)	ERAS,MAPK3,RALA,SOD2,TCF7L2
Fc Epsilon RI Signaling	0.287	0/117 (0%)	7/117 (6%)	0/117 (0%)	110/117 (94%)	ERAS,INPP5D,MAPK3,PLA2G12A,PLA2G5,RALA,VAV2
PXR/RXR Activation	0.282	0/65 (0%)	4/65 (6%)	0/65 (0%)	61/65 (94%)	ABCB1,CYP1A2,G6PC,PRKACB
Sonic Hedgehog Signaling	0.281	0/30 (0%)	2/30 (7%)	0/30 (0%)	28/30 (93%)	PRKACB,STK36

Renin-Angiotensin Signaling	0.279	0/118 (0%)	7/118 (6%)	0/118 (0%)	111/118 (94%)	ADCY2,ADCY8,ERAS,MAPK3,NOX1,PRKACB,RALA
p38 MAPK Signaling	0.279	0/118 (0%)	7/118 (6%)	0/118 (0%)	111/118 (94%)	CREB1,HSPB7,IL37,IRAK4,PLA2G12A,PLA2G5,TGFB2
Natural Killer Cell Signaling	0.272	0/119 (0%)	7/119 (6%)	0/119 (0%)	112/119 (94%)	CD247,ERAS,FCGR3A/FCGR3B,INPP5D,MAPK3,RALA,VAV2
TR/RXR Activation	0.269	0/84 (0%)	5/84 (6%)	0/84 (0%)	79/84 (94%)	ATP2A1,DIO2,G6PC,SREBF1,TBL1XR1
Germ Cell-Sertoli Cell Junction Signaling	0.269	0/172 (0%)	10/172 (6%)	0/172 (0%)	162/172 (94%)	ACTG2,BCAR1,ERAS,MAPK3,NECTIN2,RALA,RHOT1,SRC,TGFB2,TUBB4B
Assembly of RNA Polymerase III Complex	0.268	0/13 (0%)	1/13 (8%)	0/13 (0%)	12/13 (92%)	GTF3A
Role of IL-17A in Psoriasis	0.268	0/13 (0%)	1/13 (8%)	0/13 (0%)	12/13 (92%)	S100A9
Fatty Acid Activation	0.268	0/13 (0%)	1/13 (8%)	0/13 (0%)	12/13 (92%)	SLC27A1
Leukotriene Biosynthesis	0.268	0/13 (0%)	1/13 (8%)	0/13 (0%)	12/13 (92%)	GGT6
Cholesterol Biosynthesis I	0.268	0/13 (0%)	1/13 (8%)	0/13 (0%)	12/13 (92%)	EBP
NAD Phosphorylation and Dephosphorylation	0.268	0/13 (0%)	1/13 (8%)	0/13 (0%)	12/13 (92%)	NADK2
Mevalonate Pathway I	0.268	0/13 (0%)	1/13 (8%)	0/13 (0%)	12/13 (92%)	IDI1
Cholesterol Biosynthesis II (via 24,25-dihydrolanosterol)	0.268	0/13 (0%)	1/13 (8%)	0/13 (0%)	12/13 (92%)	EBP
Cholesterol Biosynthesis III (via Desmosterol)	0.268	0/13 (0%)	1/13 (8%)	0/13 (0%)	12/13 (92%)	EBP
Pregnenolone Biosynthesis	0.268	0/13 (0%)	1/13 (8%)	0/13 (0%)	12/13 (92%)	CYP4F11
Th2 Pathway	0.268	0/137 (0%)	8/137 (6%)	0/137 (0%)	129/137 (94%)	APH1A,CD247,CD3E,ICOSLG/LOC102723996,NFATC2,RUNX3,TNFRSF4,TYK2
3-phosphoinositide Degradation	0.266	0/155 (0%)	9/155 (6%)	0/155 (0%)	146/155 (94%)	EPHX2,EYA4,HACD2,INPP5D,NT5C,PPFIA2,PPP2R5E,PPP4C,PTPRN
Serotonin Degradation	0.263	0/67 (0%)	4/67 (6%)	0/67 (0%)	63/67 (94%)	ADH1A,ALDH4A1,SULT1A2,SULT1C4
IL-4 Signaling	0.261	0/85 (0%)	5/85 (6%)	0/85 (0%)	80/85 (94%)	ERAS,INPP5D,NFATC2,RALA,TYK2
Primary Immunodeficiency Signaling	0.254	0/50 (0%)	3/50 (6%)	0/50 (0%)	47/50 (94%)	CD3E,RFX5,TNFRSF13C
ErbB4 Signaling	0.253	0/68 (0%)	4/68 (6%)	0/68 (0%)	64/68 (94%)	APH1A,ERAS,MAPK3,RALA
Ethanol Degradation II	0.253	0/32 (0%)	2/32 (6%)	0/32 (0%)	30/32 (94%)	ADH1A,ALDH4A1
DNA Double-Strand Break Repair by Non-Homologous End Joining	0.248	0/14 (0%)	1/14 (7%)	0/14 (0%)	13/14 (93%)	LIG4
NAD biosynthesis II (from tryptophan)	0.248	0/14 (0%)	1/14 (7%)	0/14 (0%)	13/14 (93%)	NMNAT2
Glutaryl-CoA Degradation	0.248	0/14 (0%)	1/14 (7%)	0/14 (0%)	13/14 (93%)	CA1
Isoleucine Degradation I	0.248	0/14 (0%)	1/14 (7%)	0/14 (0%)	13/14 (93%)	BCAT1
Colanic Acid Building Blocks Biosynthesis	0.248	0/14 (0%)	1/14 (7%)	0/14 (0%)	13/14 (93%)	MPI

SPINK1 General Cancer Pathway	0.244	0/69 (0%)	4/69 (6%)	0/69 (0%)	65/69 (94%)	ERAS,MAPK3,RALA,TYK2
Choline Biosynthesis III	0.229	0/15 (0%)	1/15 (7%)	0/15 (0%)	14/15 (93%)	PLD5
Histidine Degradation VI	0.229	0/15 (0%)	1/15 (7%)	0/15 (0%)	14/15 (93%)	CYP4F11
Cytotoxic T Lymphocyte-mediated Apoptosis of Target Cells	0.228	0/34 (0%)	2/34 (6%)	0/34 (0%)	32/34 (94%)	CD247,CD3E
DNA Methylation and Transcriptional Repression Signaling	0.228	0/34 (0%)	2/34 (6%)	0/34 (0%)	32/34 (94%)	HIST1H4D,MTA2
Role of JAK2 in Hormone-like Cytokine Signaling	0.228	0/34 (0%)	2/34 (6%)	0/34 (0%)	32/34 (94%)	SH2B2,TYK2
Retinoate Biosynthesis I	0.228	0/34 (0%)	2/34 (6%)	0/34 (0%)	32/34 (94%)	AKR1C4,RDH8
Granzyme B Signaling	0.212	0/16 (0%)	1/16 (6%)	0/16 (0%)	15/16 (94%)	ENDOG
Mismatch Repair in Eukaryotes	0.212	0/16 (0%)	1/16 (6%)	0/16 (0%)	15/16 (94%)	SLC19A1
Parkinson's Signaling	0.212	0/16 (0%)	1/16 (6%)	0/16 (0%)	15/16 (94%)	GPR37
RAN Signaling	0.197	0/17 (0%)	1/17 (6%)	0/17 (0%)	16/17 (94%)	RANGAP1
γ-linolenate Biosynthesis II (Animals)	0.197	0/17 (0%)	1/17 (6%)	0/17 (0%)	16/17 (94%)	SLC27A1
Mitochondrial L-carnitine Shuttle Pathway	0.197	0/17 (0%)	1/17 (6%)	0/17 (0%)	16/17 (94%)	SLC27A1
D-myo-inositol (1,4,5)-trisphosphate Degradation	0.197	0/17 (0%)	1/17 (6%)	0/17 (0%)	16/17 (94%)	INPP5D
Histamine Degradation	0.197	0/17 (0%)	1/17 (6%)	0/17 (0%)	16/17 (94%)	ALDH4A1
IL-10 Signaling	0	0/69 (0%)	3/69 (4%)	0/69 (0%)	66/69 (96%)	FCGR2B,IL37,TYK2
Amyotrophic Lateral Sclerosis Signaling	0	0/97 (0%)	4/97 (4%)	0/97 (0%)	93/97 (96%)	CACNA1E,CAPN6,GRID1,PGF
Actin Cytoskeleton Signaling	0	0/219 (0%)	12/219 (5%)	0/219 (0%)	207/219 (95%)	ACTG2,ARPC5,BCAR1,CSK,ERAS,MAPK3,MYH11,MYL2,MYL4,MYLK3,RALA,VAV2
p53 Signaling	0	0/98 (0%)	3/98 (3%)	0/98 (0%)	95/98 (97%)	RPRM,SCO2,TP53AIP1
Coagulation System	0	0/35 (0%)	2/35 (6%)	0/35 (0%)	33/35 (94%)	F11,KLKB1
Acute Phase Response Signaling	0	0/179 (0%)	9/179 (5%)	0/179 (0%)	170/179 (95%)	CRP,ERAS,IL37,KLKB1,MAPK3,MYD88,RALA,SOD2,TF
LXR/RXR Activation	0	0/121 (0%)	5/121 (4%)	0/121 (0%)	116/121 (96%)	ABCG8,APOF,IL37,SREBF1,TF
Hepatic Fibrosis / Hepatic Stellate Cell Activation	0	0/186 (0%)	9/186 (5%)	0/186 (0%)	177/186 (95%)	COL22A1,COL28A1,COL6A6,IGFBP4,MYH11,MYL2,MYL4,PGF,TGFB2
VDR/RXR Activation	0	0/78 (0%)	2/78 (3%)	0/78 (0%)	76/78 (97%)	LRP5,TGFB2
Regulation of Actin-based Motility by Rho	0	0/94 (0%)	5/94 (5%)	0/94 (0%)	89/94 (95%)	ACTG2,ARPC5,MYL2,MYL4,RHOT1
Erythropoietin Signaling	0	0/76 (0%)	4/76 (5%)	0/76 (0%)	72/76 (95%)	ERAS,MAPK3,RALA,SRC
Caveolar-mediated Endocytosis Signaling	0	0/73 (0%)	3/73 (4%)	0/73 (0%)	70/73 (96%)	ACTG2,COPE,SRC

Clathrin-mediated Endocytosis Signaling	0	0/193 (0%)	10/193 (5%)	0/193 (0%)	183/193 (95%)	ACTG2,AP1S1,APOF,ARPC5,DNM3,MYO6,PGF,SRC,STAM,TF
IL-12 Signaling and Production in Macrophages	0	0/132 (0%)	6/132 (5%)	0/132 (0%)	126/132 (95%)	APOF,IRF8,MAPK3,MST1R,MYD88,TGFB2
Role of PKR in Interferon Induction and Antiviral Response	0	0/41 (0%)	1/41 (2%)	0/41 (0%)	40/41 (98%)	TRAF5
Role of NFAT in Regulation of the Immune Response	0	0/181 (0%)	10/181 (6%)	0/181 (0%)	171/181 (94%)	CD247,CD3E,ERAS,FCGR2B,FCGR3A/FCGR3B,MAPK3,NFATC2,ORAI1,PLCB3,RALA
LPS-stimulated MAPK Signaling	0	0/82 (0%)	4/82 (5%)	0/82 (0%)	78/82 (95%)	CREB1,ERAS,MAPK3,RALA
NF-κB Activation by Viruses	0	0/82 (0%)	4/82 (5%)	0/82 (0%)	78/82 (95%)	CR2,ERAS,MAPK3,RALA
CCR5 Signaling in Macrophages	0	0/94 (0%)	3/94 (3%)	0/94 (0%)	91/94 (97%)	CACNA1E,CD247,CD3E
CD40 Signaling	0	0/65 (0%)	3/65 (5%)	0/65 (0%)	62/65 (95%)	MAPK3,TRAF1,TRAF5
CD27 Signaling in Lymphocytes	0	0/53 (0%)	2/53 (4%)	0/53 (0%)	51/53 (96%)	CD70,TRAF5
IL-17 Signaling	0	0/80 (0%)	4/80 (5%)	0/80 (0%)	76/80 (95%)	CRP,ERAS,MAPK3,RALA
Thrombopoietin Signaling	0	0/63 (0%)	3/63 (5%)	0/63 (0%)	60/63 (95%)	ERAS,MAPK3,RALA
Induction of Apoptosis by HIV1	0	0/61 (0%)	2/61 (3%)	0/61 (0%)	59/61 (97%)	SLC25A10,TRAF1
T Helper Cell Differentiation	0	0/73 (0%)	1/73 (1%)	0/73 (0%)	72/73 (99%)	ICOSLG/LOC102723996
CCR3 Signaling in Eosinophils	0	0/124 (0%)	6/124 (5%)	0/124 (0%)	118/124 (95%)	ERAS,MAPK3,PLA2G12A,PLA2G5,PLCB3,RALA
CD28 Signaling in T Helper Cells	0	0/120 (0%)	5/120 (4%)	0/120 (0%)	115/120 (96%)	ARPC5,CD247,CD3E,CSK,NFATC2
IL-15 Signaling	0	0/71 (0%)	4/71 (6%)	0/71 (0%)	67/71 (94%)	ERAS,MAPK3,RALA,TYK2
Virus Entry via Endocytic Pathways	0	0/107 (0%)	5/107 (5%)	0/107 (0%)	102/107 (95%)	ACTG2,AP1S1,ERAS,RALA,SRC
Role of Cytokines in Mediating Communication between Immune Cells	0	0/54 (0%)	1/54 (2%)	0/54 (0%)	53/54 (98%)	IL37
Mechanisms of Viral Exit from Host Cells	0	0/41 (0%)	2/41 (5%)	0/41 (0%)	39/41 (95%)	ACTG2,CHMP2B
Reelin Signaling in Neurons	0	0/78 (0%)	4/78 (5%)	0/78 (0%)	74/78 (95%)	ARHGEF16,ARHGEF9,PAFAH1B3,SRC
Angiopoietin Signaling	0	0/75 (0%)	4/75 (5%)	0/75 (0%)	71/75 (95%)	BAD,ERAS,GRB7,RALA
Relaxin Signaling	0	0/149 (0%)	7/149 (5%)	0/149 (0%)	142/149 (95%)	ADCY2,ADCY8,CREB1,MAPK3,NT5C,PRKACB,SMARCC2
Docosahexaenoic Acid (DHA) Signaling	0	0/38 (0%)	1/38 (3%)	0/38 (0%)	37/38 (97%)	BAD
Semaphorin Signaling in Neurons	0	0/60 (0%)	2/60 (3%)	0/60 (0%)	58/60 (97%)	MAPK3,RHOT1
Lipid Antigen Presentation by CD1	0	0/26 (0%)	1/26 (4%)	0/26 (0%)	25/26 (96%)	CD3E
HGF Signaling	0	0/111 (0%)	3/111 (3%)	0/111 (0%)	108/111 (97%)	ERAS,MAPK3,RALA

HMGB1 Signaling	0	0/165 (0%)	9/165 (5%)	0/165 (0%)	156/165 (95%)	CD70,ERAS,IL17C,IL37,LTB,MAPK3,RALA,RHOT1,TGFB2
Maturity Onset Diabetes of Young (MODY) Signaling	0	0/21 (0%)	1/21 (5%)	0/21 (0%)	20/21 (95%)	CACNA1E
Polyamine Regulation in Colon Cancer	0	0/22 (0%)	1/22 (5%)	0/22 (0%)	21/22 (95%)	SAT2
Growth Hormone Signaling	0	0/71 (0%)	1/71 (1%)	0/71 (0%)	70/71 (99%)	MAPK3
Prolactin Signaling	0	0/81 (0%)	4/81 (5%)	0/81 (0%)	77/81 (95%)	ERAS,MAPK3,NMI,RALA
Renal Cell Carcinoma Signaling	0	0/80 (0%)	4/80 (5%)	0/80 (0%)	76/80 (95%)	ELOB,ERAS,MAPK3,RALA
Type I Diabetes Mellitus Signaling	0	0/111 (0%)	4/111 (4%)	0/111 (0%)	107/111 (96%)	CD247,CD3E,MYD88,PTPRN
Small Cell Lung Cancer Signaling	0	0/71 (0%)	3/71 (4%)	0/71 (0%)	68/71 (96%)	SKP2,TRAF1,TRAF5
Basal Cell Carcinoma Signaling	0	0/73 (0%)	4/73 (5%)	0/73 (0%)	69/73 (95%)	BMP8A,STK36,TCF7L2,WNT6
Glioma Signaling	0	0/110 (0%)	4/110 (4%)	0/110 (0%)	106/110 (96%)	E2F7,ERAS,MAPK3,RALA
Acute Myeloid Leukemia Signaling	0	0/89 (0%)	5/89 (6%)	0/89 (0%)	84/89 (94%)	BAD,ERAS,MAPK3,RALA,TCF7L2
Graft-versus-Host Disease Signaling	0	0/48 (0%)	1/48 (2%)	0/48 (0%)	47/48 (98%)	IL37
Type II Diabetes Mellitus Signaling	0	0/142 (0%)	4/142 (3%)	0/142 (0%)	138/142 (97%)	CACNA1E,MAPK3,SLC27A1,SMPD3
Production of Nitric Oxide and Reactive Oxygen Species in Macrophages	0	0/188 (0%)	9/188 (5%)	0/188 (0%)	179/188 (95%)	APOF,IRF8,MAPK3,MPO,PPP2CA,PPP2CB,PPP2R5E,RHOT1,TYK2
Myc Mediated Apoptosis Signaling	0	0/65 (0%)	3/65 (5%)	0/65 (0%)	62/65 (95%)	BAD,ERAS,RALA
G Beta Gamma Signaling	0	0/122 (0%)	7/122 (6%)	0/122 (0%)	115/122 (94%)	ADCY2,CACNA1E,ERAS,MAPK3,PRKACB,RALA,SRC
G Protein Signaling Mediated by Tubby	0	0/31 (0%)	1/31 (3%)	0/31 (0%)	30/31 (97%)	PLCB3
Communication between Innate and Adaptive Immune Cells	0	0/96 (0%)	4/96 (4%)	0/96 (0%)	92/96 (96%)	IL37,TLR6,TLR9,TNFRSF13C
Crosstalk between Dendritic Cells and Natural Killer Cells	0	0/89 (0%)	4/89 (4%)	0/89 (0%)	85/89 (96%)	ACTG2,LTB,NECTIN2,TLR9
Cdc42 Signaling	0	0/167 (0%)	9/167 (5%)	0/167 (0%)	158/167 (95%)	ARPC5,CD247,CD3E,LLGL1,MYL2,MYL4,RALA,SRC,VAV2
Retinoic acid Mediated Apoptosis Signaling	0	0/60 (0%)	1/60 (2%)	0/60 (0%)	59/60 (98%)	PARP2
Rac Signaling	0	0/111 (0%)	6/111 (5%)	0/111 (0%)	105/111 (95%)	ARPC5,ERAS,MAPK3,NOX1,NOX3,RALA

Ovarian Cancer Signaling	0	0/139 (0%)	8/139 (6%)	0/139 (0%)	131/139 (94%)	ERAS,MAPK3,PGF,PRKACB,RALA,SRC,TCF7L2,WNT6
HER-2 Signaling in Breast Cancer	0	0/84 (0%)	4/84 (5%)	0/84 (0%)	80/84 (95%)	BAD,ERAS,ERBB2,RALA
Atherosclerosis Signaling	0	0/124 (0%)	5/124 (4%)	0/124 (0%)	119/124 (96%)	ALOXE3,APOF,IL37,PLA2G12A,PLA2G5
Role of NFAT in Cardiac Hypertrophy	0	0/214 (0%)	12/214 (6%)	0/214 (0%)	202/214 (94%)	ADCY2,ADCY8,CACNA1E,ERAS,HDAC10,MAPK3,PLCB3,PLCD1,PRKACB, RALA,SRC,TGFB2
Glioma Invasiveness Signaling	0	0/74 (0%)	4/74 (5%)	0/74 (0%)	70/74 (95%)	ERAS,MAPK3,RALA,RHOT1
B Cell Development	0	0/36 (0%)	1/36 (3%)	0/36 (0%)	35/36 (97%)	SPN
IL-1 Signaling	0	0/91 (0%)	5/91 (5%)	0/91 (0%)	86/91 (95%)	ADCY2,ADCY8,IRAK4,MYD88,PRKACB
RANK Signaling in Osteoclasts	0	0/88 (0%)	4/88 (5%)	0/88 (0%)	84/88 (95%)	MAPK3,NFATC2,SRC,TRAF5
Glioblastoma Multiforme Signaling	0	0/165 (0%)	9/165 (5%)	0/165 (0%)	156/165 (95%)	E2F7,ERAS,MAPK3,PLCB3,PLCD1,RALA,RHOT1,SRC,WNT6
Granzyme A Signaling	0	0/20 (0%)	1/20 (5%)	0/20 (0%)	19/20 (95%)	HIST1H1E
Role of Wnt/GSK-3β Signaling in the Pathogenesis of Influenza	0	0/78 (0%)	2/78 (3%)	0/78 (0%)	76/78 (97%)	TCF7L2,WNT6
TWEAK Signaling	0	0/35 (0%)	1/35 (3%)	0/35 (0%)	34/35 (97%)	TRAF1
Nur77 Signaling in T Lymphocytes	0	0/59 (0%)	2/59 (3%)	0/59 (0%)	57/59 (97%)	CD247,CD3E
PKCθ Signaling in T Lymphocytes	0	0/155 (0%)	8/155 (5%)	0/155 (0%)	147/155 (95%)	CACNA1E,CD247,CD3E,ERAS,MAPK3,NFATC2,RALA,VAV2
TNFR1 Signaling	0	0/50 (0%)	1/50 (2%)	0/50 (0%)	49/50 (98%)	MAP4K2
TNFR2 Signaling	0	0/30 (0%)	1/30 (3%)	0/30 (0%)	29/30 (97%)	TRAF1
Role of PI3K/AKT Signaling in the Pathogenesis of Influenza	0	0/64 (0%)	1/64 (2%)	0/64 (0%)	63/64 (98%)	MAPK3
Role of Hypercytokinemia/hyperchemokinemia in the Pathogenesis of Influenza	0	0/43 (0%)	1/43 (2%)	0/43 (0%)	42/43 (98%)	IL37
OX40 Signaling Pathway	0	0/90 (0%)	4/90 (4%)	0/90 (0%)	86/90 (96%)	CD247,CD3E,TNFRSF4,TRAF5
Tumoricidal Function of Hepatic Natural Killer Cells	0	0/24 (0%)	1/24 (4%)	0/24 (0%)	23/24 (96%)	ENDOG
Intrinsic Prothrombin Activation Pathway	0	0/42 (0%)	2/42 (5%)	0/42 (0%)	40/42 (95%)	F11,KLKB1
Cell Cycle Control of Chromosomal Replication	0	0/56 (0%)	2/56 (4%)	0/56 (0%)	54/56 (96%)	CDK20,ORC5
Role of Tissue Factor in Cancer	0	0/120 (0%)	4/120 (3%)	0/120 (0%)	116/120 (97%)	ERAS,MAPK3,RALA,SRC
IL-17A Signaling in Airway Cells	0	0/64 (0%)	2/64 (3%)	0/64 (0%)	62/64 (97%)	MAPK3,TYK2
Role of IL-17A in Arthritis	0	0/54 (0%)	1/54 (2%)	0/54 (0%)	53/54 (98%)	MAPK3
IL-17A Signaling in Fibroblasts	0	0/35 (0%)	1/35 (3%)	0/35 (0%)	34/35 (97%)	MAPK3

IL-17A Signaling in Gastric Cells	0	0/25 (0%)	1/25 (4%)	0/25 (0%)	24/25 (96%)	MAPK3
Role of IL-17F in Allergic Inflammatory Airway Diseases	0	0/45 (0%)	2/45 (4%)	0/45 (0%)	43/45 (96%)	CREB1,MAPK3
Role of JAK1, JAK2 and TYK2 in Interferon Signaling	0	0/24 (0%)	1/24 (4%)	0/24 (0%)	23/24 (96%)	TYK2
Role of JAK1 and JAK3 in γc Cytokine Signaling	0	0/69 (0%)	3/69 (4%)	0/69 (0%)	66/69 (96%)	ERAS,MAPK3,RALA
Actin Nucleation by ARP-WASP Complex	0	0/72 (0%)	4/72 (6%)	0/72 (0%)	68/72 (94%)	ARPC5,ERAS,RALA,RHOT1
NGF Signaling	0	0/114 (0%)	5/114 (4%)	0/114 (0%)	109/114 (96%)	CREB1,ERAS,MAPK3,RALA,SMPD3
Signaling by Rho Family GTPases	0	0/243 (0%)	11/243 (5%)	0/243 (0%)	232/243 (95%)	ACTG2,ARHGEF16,ARHGEF9,ARPC5,GFAP,MAPK3,MYL2,MYL4,NOX1,NOX3,RHOT1
RhoGDI Signaling	0	0/180 (0%)	9/180 (5%)	0/180 (0%)	171/180 (95%)	ACTG2,ARHGAP8/PRR5-ARHGAP8,ARHGEF16,ARHGEF9,ARPC5,MYL2,MYL4,RHOT1,SRC
Hematopoiesis from Pluripotent Stem Cells	0	0/49 (0%)	2/49 (4%)	0/49 (0%)	47/49 (96%)	CD247,CD3E
nNOS Signaling in Neurons	0	0/47 (0%)	1/47 (2%)	0/47 (0%)	46/47 (98%)	CAPN6
nNOS Signaling in Skeletal Muscle Cells	0	0/41 (0%)	2/41 (5%)	0/41 (0%)	39/41 (95%)	CACNA1E,RYR3
Ephrin B Signaling	0	0/72 (0%)	3/72 (4%)	0/72 (0%)	69/72 (96%)	EPHB3,MAPK3,VAV2
ErbB Signaling	0	0/94 (0%)	4/94 (4%)	0/94 (0%)	90/94 (96%)	ERAS,ERBB2,MAPK3,RALA
Pyrimidine Ribonucleotides De Novo Biosynthesis	0	0/44 (0%)	1/44 (2%)	0/44 (0%)	43/44 (98%)	NME3
The Visual Cycle	0	0/20 (0%)	1/20 (5%)	0/20 (0%)	19/20 (95%)	RDH8
Pyridoxal 5'-phosphate Salvage Pathway	0	0/65 (0%)	3/65 (5%)	0/65 (0%)	62/65 (95%)	G6PC,GRK4,MAPK3
Pyrimidine Deoxyribonucleotides De Novo Biosynthesis I	0	0/22 (0%)	1/22 (5%)	0/22 (0%)	21/22 (95%)	NME3
Tryptophan Degradation X (Mammalian, via Tryptamine)	0	0/25 (0%)	1/25 (4%)	0/25 (0%)	24/25 (96%)	ALDH4A1
tRNA Charging	0	0/39 (0%)	1/39 (3%)	0/39 (0%)	38/39 (97%)	VARS
D-myo-inositol (1,4,5,6)-Tetrakisphosphate Biosynthesis	0	0/141 (0%)	8/141 (6%)	0/141 (0%)	133/141 (94%)	EPHX2,EYA4,HACD2,NT5C,PPFIA2,PPP2R5E,PPP4C,PTPRN
Valine Degradation I	0	0/18 (0%)	1/18 (6%)	0/18 (0%)	17/18 (94%)	BCAT1
D-myo-inositol (3,4,5,6)-tetrakisphosphate Biosynthesis	0	0/141 (0%)	8/141 (6%)	0/141 (0%)	133/141 (94%)	EPHX2,EYA4,HACD2,NT5C,PPFIA2,PPP2R5E,PPP4C,PTPRN
3-phosphoinositide Biosynthesis	0	0/165 (0%)	9/165 (5%)	0/165 (0%)	156/165 (95%)	CDIPT,EPHX2,EYA4,HACD2,NT5C,PPFIA2,PPP2R5E,PPP4C,PTPRN
Retinol Biosynthesis	0	0/42 (0%)	2/42 (5%)	0/42 (0%)	40/42 (95%)	LIPE,RDH8

Tryptophan Degradation III (Eukaryotic)	0	0/22 (0%)	1/22 (5%)	0/22 (0%)	21/22 (95%)	CA1
Triacylglycerol Biosynthesis	0	0/41 (0%)	2/41 (5%)	0/41 (0%)	39/41 (95%)	LCLAT1,TAZ
Salvage Pathways of Pyrimidine Ribonucleotides	0	0/97 (0%)	5/97 (5%)	0/97 (0%)	92/97 (95%)	G6PC,GRK4,MAPK3,NME3,UPP1
Pyrimidine Ribonucleotides Interconversion	0	0/42 (0%)	1/42 (2%)	0/42 (0%)	41/42 (98%)	NME3
Glycolysis I	0	0/26 (0%)	1/26 (4%)	0/26 (0%)	25/26 (96%)	ALDOA
Gluconeogenesis I	0	0/26 (0%)	1/26 (4%)	0/26 (0%)	25/26 (96%)	ALDOA
Stearate Biosynthesis I (Animals)	0	0/45 (0%)	1/45 (2%)	0/45 (0%)	44/45 (98%)	SLC27A1
Noradrenaline and Adrenaline Degradation	0	0/35 (0%)	2/35 (6%)	0/35 (0%)	33/35 (94%)	ADH1A,ALDH4A1
Oxidative Ethanol Degradation III	0	0/19 (0%)	1/19 (5%)	0/19 (0%)	18/19 (95%)	ALDH4A1
Ethanol Degradation IV	0	0/23 (0%)	1/23 (4%)	0/23 (0%)	22/23 (96%)	ALDH4A1
Fatty Acid β-oxidation I	0	0/32 (0%)	1/32 (3%)	0/32 (0%)	31/32 (97%)	SLC27A1
Superpathway of Methionine Degradation	0	0/37 (0%)	2/37 (5%)	0/37 (0%)	35/37 (95%)	AHCY,MGMT
Antioxidant Action of Vitamin C	0	0/107 (0%)	6/107 (6%)	0/107 (0%)	101/107 (94%)	MAPK3,PLA2G12A,PLA2G5,PLCB3,PLCD1,PLD5
Gαq Signaling	0	0/157 (0%)	9/157 (6%)	0/157 (0%)	148/157 (94%)	ADRA1A,AVPR1A,CALCR,CSK,MAPK3,NFATC2,PLCB3,PLD5,RHOT1
Granulocyte Adhesion and Diapedesis	0	0/179 (0%)	8/179 (4%)	0/179 (0%)	171/179 (96%)	CLDN14,CLDN15,CLDN2,CXCL13,IL37,MMP15,MMP24,MMP26
Regulation of the Epithelial-Mesenchymal Transition Pathway	0	0/193 (0%)	10/193 (5%)	0/193 (0%)	183/193 (95%)	APH1A,ERAS,FGFR4,MAPK3,RALA,SNAI1,TCF7L2,TGFB2,TYK2,WNT6
Tec Kinase Signaling	0	0/164 (0%)	5/164 (3%)	0/164 (0%)	159/164 (97%)	ACTG2,RHOT1,SRC,TYK2,VAV2
UVB-Induced MAPK Signaling	0	0/52 (0%)	2/52 (4%)	0/52 (0%)	50/52 (96%)	BAD,MAPK3
Adipogenesis pathway	0	0/134 (0%)	6/134 (4%)	0/134 (0%)	128/134 (96%)	FGFR4,HDAC10,SMAD1,SMAD5,SREBF1,TBL1XR1
PCP pathway	0	0/61 (0%)	2/61 (3%)	0/61 (0%)	59/61 (97%)	JUNB,WNT6
EGF Signaling	0	0/55 (0%)	2/55 (4%)	0/55 (0%)	53/55 (96%)	MAPK3,SRC
SAPK/JNK Signaling	0	0/102 (0%)	3/102 (3%)	0/102 (0%)	99/102 (97%)	ERAS,MAP4K2,RALA
Interferon Signaling	0	0/36 (0%)	2/36 (6%)	0/36 (0%)	34/36 (94%)	IFITM3,TYK2
IL-2 Signaling	0	0/61 (0%)	3/61 (5%)	0/61 (0%)	58/61 (95%)	ERAS,MAPK3,RALA
FGF Signaling	0	0/84 (0%)	3/84 (4%)	0/84 (0%)	81/84 (96%)	CREB1,FGFR4,MAPK3
JAK/Stat Signaling	0	0/80 (0%)	4/80 (5%)	0/80 (0%)	76/80 (95%)	ERAS,MAPK3,RALA,TYK2
Cell Cycle: G2/M DNA Damage Checkpoint Regulation	0	0/49 (0%)	2/49 (4%)	0/49 (0%)	47/49 (96%)	RPRM,SKP2
Antigen Presentation Pathway	0	0/39 (0%)	1/39 (3%)	0/39 (0%)	38/39 (97%)	PSMB5

B Cell Receptor Signaling	0	0/185 (0%)	10/185 (5%)	0/185 (0%)	175/185 (95%)	BAD,CREB1,CSK,ERAS,FCGR2B,INPP5D,MAPK3,NFATC2,RALA,VAV2
Phototransduction Pathway	0	0/53 (0%)	3/53 (6%)	0/53 (0%)	50/53 (94%)	GUCA1A,PRKACB,RGR
Death Receptor Signaling	0	0/91 (0%)	3/91 (3%)	0/91 (0%)	88/91 (97%)	ACTG2,HSPB7,PARP2
PPAR Signaling	0	0/104 (0%)	5/104 (5%)	0/104 (0%)	99/104 (95%)	ERAS,HSP90B1,IL37,MAPK3,RALA
IGF-1 Signaling	0	0/104 (0%)	6/104 (6%)	0/104 (0%)	98/104 (94%)	BAD,ERAS,IGFBP4,MAPK3,PRKACB,RALA
Glutamate Receptor Signaling	0	0/57 (0%)	2/57 (4%)	0/57 (0%)	55/57 (96%)	GRID1,HOMER3
GPCR-Mediated Nutrient Sensing in Enteroendocrine Cells	0	0/112 (0%)	6/112 (5%)	0/112 (0%)	106/112 (95%)	ADCY2,ADCY8,CACNA1E,PLCB3,PLCD1,PRKACB
Gustation Pathway	0	0/153 (0%)	6/153 (4%)	0/153 (0%)	147/153 (96%)	ADCY2,ADCY8,CACNA1E,PRKACB,TAS2R14,TAS2R8
Macropinocytosis Signaling	0	0/76 (0%)	3/76 (4%)	0/76 (0%)	73/76 (96%)	ERAS,RALA,SRC
PD-1, PD-L1 cancer immunotherapy pathway	0	0/106 (0%)	6/106 (6%)	0/106 (0%)	100/106 (94%)	CD247,CSK,RASGRP1,SKP2,TGFB2,TYK2
Sumoylation Pathway	0	0/103 (0%)	5/103 (5%)	0/103 (0%)	98/103 (95%)	RANGAP1,RHOT1,SAE1,SLC19A1,SUMO4
Th1 and Th2 Activation Pathway	0	0/172 (0%)	8/172 (5%)	0/172 (0%)	164/172 (95%)	APH1A,CD247,CD3E,ICOSLG/LOC102723996,NFATC2,RUNX3,TNFRSF4,TYK2
Th1 Pathway	0	0/122 (0%)	7/122 (6%)	0/122 (0%)	115/122 (94%)	APH1A,CD247,CD3E,ICOSLG/LOC102723996,NFATC2,RUNX3,TYK2
Osteoarthritis Pathway	0	0/213 (0%)	9/213 (4%)	0/213 (0%)	204/213 (96%)	CREB1,DLX5,ITLN1,PGF,S100A9,SMAD1,SMAD5,SPHK1,TCF7L2
GP6 Signaling Pathway	0	0/119 (0%)	4/119 (3%)	0/119 (0%)	115/119 (97%)	COL22A1,COL28A1,COL6A6,NOX1
IL-7 Signaling Pathway	0	0/78 (0%)	4/78 (5%)	0/78 (0%)	74/78 (95%)	BAD,FOXO4,FOXO6,MAPK3
Th17 Activation Pathway	0	0/91 (0%)	5/91 (5%)	0/91 (0%)	86/91 (95%)	HSP90B1,IRAK4,MYD88,NFATC2,TYK2
SPINK1 Pancreatic Cancer Pathway	0	0/60 (0%)	2/60 (3%)	0/60 (0%)	58/60 (97%)	CPA6,KLKB1
Apelin Pancreas Signaling Pathway	0	0/44 (0%)	1/44 (2%)	0/44 (0%)	43/44 (98%)	PRKACB
Apelin Endothelial Signaling Pathway	0	0/115 (0%)	6/115 (5%)	0/115 (0%)	109/115 (95%)	ADCY2,ADCY8,ERAS,MAPK3,PLCB3,RALA
Apelin Muscle Signaling Pathway	0	0/19 (0%)	1/19 (5%)	0/19 (0%)	18/19 (95%)	NRF1
BAG2 Signaling Pathway	0	0/43 (0%)	2/43 (5%)	0/43 (0%)	41/43 (95%)	HSPA1L,MAPK3
Cardiac Hypertrophy Signaling (Enhanced)	0	0/486 (0%)	24/486 (5%)	0/486 (0%)	462/486 (95%)	ADCY2,ADCY8,ADRA1A,ATP2A1,CACNA1E,CD70,ERAS,FGFR4,HDAC10,HSPB7,IL11RA,IL17C,IL37,LTB,MAPK3,NFATC2,NPPB,PLCB3,PLCD1,PRKACB,RALA,RYR3,TGFB2,WNT6
FAT10 Cancer Signaling Pathway	0	0/46 (0%)	1/46 (2%)	0/46 (0%)	45/46 (98%)	TGFB2

T Cell Exhaustion Signaling Pathway	0	0/175 (0%)	8/175 (5%)	0/175 (0%)	167/175 (95%)	ERAS,MAPK3,NFATC2,PPP2CA,PPP2CB,PPP2R5E,RALA,TYK2
IL-23 Signaling Pathway	0	0/44 (0%)	1/44 (2%)	0/44 (0%)	43/44 (98%)	TYK2
Systemic Lupus Erythematosus In T Cell Signaling Pathway	0	0/334 (0%)	14/334 (4%)	0/334 (0%)	320/334 (96%)	CD247,CD3E,CD70,CREB1,ERAS,ICOSLG/LOC102723996,MAPK3,NFATC2,ORAI1,PPP2CA,PPP2CB,PPP2R5E,RALA,RHOT1
HOTAIR Regulatory Pathway	0	0/159 (0%)	7/159 (4%)	0/159 (0%)	152/159 (96%)	ERBB2,HSF1,MMP15,MMP24,MMP26,TCF7L2,WNT6
GM-CSF Signaling	0	0/70 (0%)	3/70 (4%)	0/70 (0%)	67/70 (96%)	ERAS,MAPK3,RALA
Complement System	0	0/37 (0%)	2/37 (5%)	0/37 (0%)	35/37 (95%)	CR2,MASP2
Ephrin Receptor Signaling	0	0/180 (0%)	10/180 (6%)	0/180 (0%)	170/180 (94%)	ARPC5,BCAR1,CREB1,EPHA5,EPHB3,ERAS,MAPK3,PGF,RALA,SRC

Supplementary Table 3. Enrichment analysis results showing the key markers associated with pathways such as DHH, Activin, WNT, TGFb, Notch, and PDGF signaling.

ACTIVIN SIGNALING PATHWAY

Ensembl	Location	Symbol	B4_0.y	B4_1.y	B4_1.y	log2Fold Change	pvalue
ENSG00000121989.10	2:148602086-148688393	ACVR2A	689.55	717.21	717.21	-0.12	0.61
ENSG00000175189.3	12:57828543-57844611	INHBC	2.98	5.47	5.47	-0.04	0.71
ENSG00000139269.2	12:57846106-57853063	INHBE	584.38	1053.37	1053.37	-0.58	0.03

DHH SIGNALING PATHWAY

Ensembl	Location	Symbol	B4_0.y	B4_1.y	B4_1.y	log2Fold Change	pvalue
ENSG00000126603.4	16:4364762-4389598	GLIS2	2002.17	2421.01	2421.01	-0.29	0.23
ENSG00000082701.10	3:119540170-119813264	GSK3B	3636.26	4009.83	4009.83	-0.20	0.39
ENSG00000142875.15	1:84543745-84704181	PRKACB	1665.83	2046.52	2046.52	-0.30	0.21
ENSG00000165059.5	9:71627469-71629039	PRKACG	0.00	1.09	1.09	-0.02	0.68
ENSG00000114302.11	3:48782030-48885279	PRKAR2A	3160.02	3610.16	3610.16	-0.23	0.31
ENSG00000117425.9	1:45285516-45308735	PTCH2	59.53	70.08	70.08	-0.12	0.63

WNT SIGNALING PATHWAY

Ensembl	Location	Symbol	B4_0.y	B4_1.y	B4_1.y	log2Fold Change	pvalue
ENSG00000115170.9	2:158592958-158732374	ACVR1	2313.71	2012.58	2012.58	0.06	0.79
ENSG00000135503.8	12:52345451-52390862	ACVR1B	1779.93	1391.72	1391.72	0.17	0.46
ENSG00000123612.11	2:158383279-158485517	ACVR1C	12.90	9.85	9.85	0.03	0.87
ENSG00000114739.9	3:38495342-38534633	ACVR2B	261.93	211.33	211.33	0.11	0.67
ENSG00000105221.12	19:40736224-40791443	AKT2	3680.91	3271.81	3271.81	0.04	0.86
ENSG00000117020.12	1:243651535-244014381	AKT3	3631.30	3080.19	3080.19	0.09	0.69
ENSG00000134982.12	5:112043195-112181936	APC	992.16	692.03	692.03	0.28	0.26
ENSG00000115266.7	19:1446300-1473243	APC2	53.58	42.70	42.70	0.06	0.80
ENSG00000157500.6	3:57261765-57307496	APPL1	1703.54	1691.75	1691.75	-0.08	0.72
ENSG00000136044.7	12:105567074-105630016	APPL2	3813.86	2911.56	2911.56	0.20	0.38
ENSG00000103126.10	16:337440-402673	AXIN1	1642.02	1606.34	1606.34	-0.07	0.78
ENSG00000168646.8	17:63524681-63557765	AXIN2	275.82	132.49	132.49	0.45	0.09
ENSG00000116128.5	1:147013182-147098017	BCL9	359.16	281.41	281.41	0.14	0.58
ENSG00000204217.8	2:203241659-203432474	BMPR2	3926.96	3484.24	3484.24	0.04	0.85
ENSG00000166167.13	10:103113820-103317078	BTRC	964.38	893.51	893.51	-0.01	0.98
ENSG00000110092.3	11:69455855-69469242	CCND1	9791.61	6008.17	6008.17	0.42	0.08
ENSG00000026508.12	11:35160417-35253949	CD44	14884.36	8779.57	8779.57	0.45	0.06
ENSG00000039068.14	16:68771128-68869451	CDH1	496.08	480.70	480.70	-0.05	0.84
ENSG00000154162.9	5:21750782-22853731	CDH12	3.97	0.00	0.00	0.05	0.49
ENSG00000170558.4	18:25530930-25757410	CDH2	3315.79	3312.32	3312.32	-0.09	0.69
ENSG00000062038.9	16:68670092-68756519	CDH3	87.31	78.84	78.84	0.01	0.97
ENSG00000179776.13	16:66400533-66438686	CDH5	5.95	0.00	0.00	0.07	0.40
ENSG00000147889.12	9:21967751-21995300	CDKN2A	289.71	282.51	282.51	-0.05	0.84
ENSG00000141551.10	17:80196899-80231607	CSNK1D	6167.25	4872.67	4872.67	0.17	0.45
ENSG00000213923.6	22:38686697-38794527	CSNK1E	10374.00	7505.01	7505.01	0.26	0.25
ENSG00000169118.11	15:64457716-64648442	CSNK1G1	890.96	750.06	750.06	0.09	0.70
ENSG00000133275.11	19:1941188-1981337	CSNK1G2	2915.95	2682.71	2682.71	0.00	1.00
ENSG00000151292.13	5:122847793-122952739	CSNK1G3	2904.05	2653.14	2653.14	0.01	0.97
ENSG00000101266.12	20:459116-524465	CSNK2A1	6567.09	5743.18	5743.18	0.06	0.80

ENSG00000070770.4	16:58191811-58231824	CSNK2A2	3916.05	3607.97	3607.97	0.00	1.00
ENSG00000204435.9	6:31633013-31638120	CSNK2B	209.35	111.69	111.69	0.37	0.16
ENSG00000107984.5	10:54074056-54077802	DKK1	1485.26	1288.79	1288.79	0.06	0.79
ENSG00000104371.4	8:42231586-42234750	DKK4	2.98	1.09	1.09	0.02	0.74
ENSG00000107404.13	1:1270656-1284730	DVL1	4469.67	3841.20	3841.20	0.08	0.73
ENSG00000161202.13	3:183873176-183891398	DVL3	3806.91	3552.12	3552.12	-0.02	0.95
ENSG00000100393.9	22:41487790-41576081	EP300	1898.00	1672.04	1672.04	0.05	0.84
ENSG00000165879.7	10:99079022-99081672	FRAT1	117.07	60.22	60.22	0.31	0.22
ENSG00000157240.2	7:90893783-90898123	FZD1	683.60	523.40	523.40	0.18	0.46
ENSG00000180340.5	17:42634925-42636907	FZD2	759.00	733.64	733.64	-0.05	0.84
ENSG00000104290.6	8:28351729-28431775	FZD3	217.28	169.72	169.72	0.13	0.62
ENSG00000174804.3	11:86656721-86666433	FZD4	4692.91	4271.53	4271.53	0.01	0.96
ENSG00000163251.3	2:208627310-208634287	FZD5	126.00	157.68	157.68	-0.22	0.42
ENSG00000164930.7	8:104310661-104345094	FZD6	3401.12	2822.87	2822.87	0.11	0.61
ENSG00000155760.1	2:202899310-202903160	FZD7	177.60	152.20	152.20	0.05	0.84
ENSG00000177283.4	10:35927177-35930362	FZD8	157.75	200.38	200.38	-0.24	0.36
ENSG00000188763.3	7:72848109-72850450	FZD9	26.79	25.18	25.18	-0.01	0.98
ENSG00000087258.9	16:56225302-56391356	GNAO1	8.93	4.38	4.38	0.05	0.68
ENSG00000105723.7	19:42734338-42746777	GSK3A	950.49	738.02	738.02	0.17	0.47
ENSG00000082701.10	3:119540170-119813264	GSK3B	3636.26	4009.83	4009.83	-0.20	0.39
ENSG00000116478.7	1:32757687-32799236	HDAC1	2956.63	2607.15	2607.15	0.05	0.83
ENSG00000135100.13	12:121416346-121440315	HNF1A	24.80	18.61	18.61	0.05	0.80
ENSG00000166333.9	11:6624961-6632102	ILK	4535.15	3845.58	3845.58	0.09	0.68
ENSG00000162337.7	11:68080077-68216743	LRP5	1071.53	916.50	916.50	0.08	0.75
ENSG00000070018.4	12:12268959-12419946	LRP6	1156.86	1148.64	1148.64	-0.08	0.73
ENSG00000104814.8	19:39078281-39109522	MAP4K1	18.85	7.66	7.66	0.12	0.46
ENSG00000072518.16	11:63606400-63678491	MARK2	1067.56	911.03	911.03	0.08	0.74
ENSG00000136997.10	8:128747680-128753674	MYC	18267.61	15077.91	15077.91	0.12	0.58
ENSG00000087095.8	17:26368763-26523407	NLK	334.36	309.88	309.88	-0.01	0.98
ENSG00000116833.9	1:199996730-200146552	NR5A2	14.88	5.47	5.47	0.11	0.48
ENSG00000112033.9	6:35310335-35395968	PPARD	2795.90	2244.71	2244.71	0.15	0.52
ENSG00000155367.11	1:113252616-113258099	PPM1J	110.13	63.51	63.51	0.26	0.32
ENSG00000163590.9	3:160473390-160796695	PPM1L	29.76	26.28	26.28	0.01	0.95
ENSG00000137713.11	11:111597632-111637151	PPP2R1B	1511.06	1389.53	1389.53	0.00	1.00
ENSG00000221914.4	8:26149007-26230196	PPP2R2A	3620.38	3402.11	3402.11	-0.02	0.92
ENSG00000156475.14	5:145967936-146464347	PPP2R2B	27.78	24.09	24.09	0.02	0.94
ENSG00000074211.9	4:6322305-6565327	PPP2R2C	762.97	323.02	323.02	0.61	0.02
ENSG00000073711.6	3:135684515-135866733	PPP2R3A	668.71	586.91	586.91	0.05	0.84
ENSG00000167393.12	X:294698-347690	PPP2R3B	331.38	220.09	220.09	0.27	0.30
ENSGR0000167393.12	Y:244698-297690	PPP2R3B	0.00	0.00	0.00	NA	NA
ENSG00000066027.7	1:212458879-212535200	PPP2R5A	1732.31	1356.68	1356.68	0.17	0.46
ENSG00000068971.9	11:64685025-64701945	PPP2R5B	636.97	603.34	603.34	-0.03	0.91
ENSG00000112640.10	6:42952237-42980080	PPP2R5D	1613.25	1398.29	1398.29	0.06	0.78
ENSG00000131759.13	17:38465444-38513094	RARA	842.34	689.84	689.84	0.12	0.62
ENSG00000077092.14	3:25215823-25639423	RARB	1040.77	409.52	409.52	0.21	0.30
ENSG00000172819.12	12:53604354-53626764	RARG	1395.97	987.67	987.67	0.27	0.26
ENSG00000143947.8	2:55459039-55462989	RPS27A	43219.40	34615.69	34615.69	0.16	0.48
ENSG00000183207.8	19:49496705-49519252	RUVBL2	2583.58	2207.49	2207.49	0.08	0.72
ENSG00000104332.7	8:41119481-41167016	SFRP1	4407.17	3709.80	3709.80	0.10	0.66
ENSG00000145423.4	4:154701744-154710272	SFRP2	4.96	4.38	4.38	0.00	0.98
ENSG00000106483.7	7:37945543-38065297	SFRP4	246.06	1601.96	1601.96	-0.12	0.32
ENSG00000128602.5	7:128828713-128853386	SMO	294.67	244.18	244.18	0.09	0.72

ENSG00000182968.3	13:112721913-112726020	SOX1	1.98	0.00	0.00	0.02	0.61
ENSG00000176887.5	2:5832799-5841516	SOX11	6.95	1.09	1.09	0.07	0.46
ENSG00000177732.6	20:306207-310865	SOX12	1254.09	850.80	850.80	0.31	0.21
ENSG00000143842.10	1:204042243-204096863	SOX13	1169.75	775.25	775.25	0.33	0.19
ENSG00000164736.5	8:55370495-55373448	SOX17	283.76	146.73	146.73	0.42	0.11
ENSG00000181449.2	3:181429714-181432221	SOX2	11.91	10.95	10.95	0.00	1.00
ENSG00000134532.11	12:23682440-24103966	SOX5	205.38	197.10	197.10	-0.03	0.90
ENSG00000110693.11	11:15987995-16761138	SOX6	10.91	7.66	7.66	0.03	0.83
ENSG00000005513.9	16:1031808-1036979	SOX8	140.89	49.27	49.27	0.46	0.07
ENSG00000125398.5	17:70117161-70122561	SOX9	86.32	42.70	42.70	0.28	0.25
ENSG00000197122.7	20:35973088-36034453	SRC	1548.76	1372.01	1372.01	0.04	0.86
ENSG00000100324.9	22:39795746-39833065	TAB1	749.08	581.44	581.44	0.17	0.49
ENSG00000071564.10	19:1609291-1652604	TCF3	2262.12	1811.10	1811.10	0.15	0.51
ENSG00000196628.9	18:52889562-53332018	TCF4	2349.43	1507.79	1507.79	0.37	0.13
ENSG00000081059.15	5:133450402-133487556	TCF7	23.81	16.42	16.42	0.07	0.74
ENSG00000152284.4	2:85360533-85537511	TCF7L1	108.15	60.22	60.22	0.27	0.29
ENSG00000105329.5	19:41807492-41859816	TGFB1	4097.61	2625.77	2625.77	0.37	0.12
ENSG00000119699.3	14:76424442-76449334	TGFB3	82.35	59.13	59.13	0.13	0.61
ENSG00000163513.13	3:30647994-30735634	TGFBR2	10561.52	8685.40	8685.40	0.13	0.57
ENSG00000069702.6	1:92145902-92371892	TGFBR3	1144.95	837.66	837.66	0.23	0.33
ENSG00000196781.9	9:84198598-84304220	TLE1	1489.23	1445.38	1445.38	-0.06	0.81
ENSG00000140332.11	15:70340129-70390515	TLE3	2930.83	2371.73	2371.73	0.14	0.54
ENSG00000106829.14	9:82186688-82341658	TLE4	833.41	536.54	536.54	0.34	0.18
ENSG00000221983.3	19:18682540-18688360	UBA52	17659.42	13733.27	13733.27	0.19	0.40
ENSG00000125084.7	12:49372398-49375459	WNT1	9.92	3.28	3.28	0.08	0.52
ENSG00000135925.4	2:219745085-219764303	WNT10A	1.98	0.00	0.00	0.02	0.61
ENSG00000169884.9	12:49359123-49365546	WNT10B	32.74	19.71	19.71	0.12	0.58
ENSG00000085741.8	11:75897369-75921780	WNT11	4.96	1.09	1.09	0.05	0.57
ENSG00000105989.4	7:116916685-116963343	WNT2	5.95	0.00	0.00	0.07	0.40
ENSG00000134245.13	1:113009163-113072787	WNT2B	2916.94	1781.54	1781.54	0.41	0.09
ENSG00000108379.5	17:44839872-44910520	WNT3	286.73	271.56	271.56	-0.03	0.92
ENSG00000154342.4	1:228194752-228248961	WNT3A	2.98	0.00	0.00	0.04	0.54
ENSG00000162552.10	1:22443798-22470462	WNT4	13.89	2.19	2.19	0.13	0.31
ENSG00000114251.9	3:55499743-55523973	WNT5A	11327.47	7030.88	7030.88	0.41	0.09
ENSG00000111186.8	12:1639057-1756409	WNT5B	1159.83	846.42	846.42	0.24	0.33
ENSG00000115596.3	2:219724544-219738955	WNT6	1.98	0.00	0.00	0.02	0.61
ENSG00000154764.5	3:13857755-13921618	WNT7A	0.99	0.00	0.00	0.01	0.72
ENSG00000061492.7	5:137419581-137428054	WNT8A	1.98	0.00	0.00	0.02	0.61
ENSG00000143816.7	1:228106357-228135599	WNT9A	16.87	4.38	4.38	0.14	0.35

TGF beta SIGNALING PATHWAY

Ensembl	Location	Symbol	B4_0.y	B4_1.y	B4_1.y	log2Fold Change	pvalue
ENSG00000115170.9	2:158592958-158732374	ACVR1	2313.71	2012.58	2012.58	0.06	0.79
ENSG00000135503.8	12:52345451-52390862	ACVR1B	1779.93	1391.72	1391.72	0.17	0.46
ENSG00000123612.11	2:158383279-158485517	ACVR1C	12.90	9.85	9.85	0.03	0.87
ENSG00000114739.9	3:38495342-38534633	ACVR2B	261.93	211.33	211.33	0.11	0.67
ENSG00000125845.6	20:6748311-6760927	BMP2	565.53	428.14	428.14	0.19	0.46
ENSG00000101144.8	20:55743804-55841685	BMP7	61.51	47.08	47.08	0.08	0.73
ENSG00000107779.7	10:88516407-88692595	BMPR1A	1868.23	1582.25	1582.25	0.09	0.70
ENSG00000204217.8	2:203241659-203432474	BMPR2	3926.96	3484.24	3484.24	0.04	0.85

ENSG00000070831.11	1:22379120-22419437	CDC42	8976.05	8255.08	8255.08	0.00	1.00
ENSG00000100393.9	22:41487790-41576081	EP300	1898.00	1672.04	1672.04	0.05	0.84
ENSG00000187682.1	X:48687283-48688548	ERAS	0.99	0.00	0.00	0.01	0.72
ENSG00000170345.5	14:75745477-75748933	FOS	64318.63	58356.01	58356.01	0.02	0.94
ENSG00000160973.7	8:145698795-145701718	FOXH1	11.91	3.28	3.28	0.10	0.44
ENSG00000133937.3	14:95234553-95236562	GSC	2.98	1.09	1.09	0.02	0.74
ENSG00000116478.7	1:32757687-32799236	HDAC1	2956.63	2607.15	2607.15	0.05	0.83
ENSG00000180806.4	12:54388679-54397121	HOXC9	195.46	169.72	169.72	0.05	0.86
ENSG00000123999.4	2:220433884-220440435	INHA	92.27	51.46	51.46	0.25	0.32
ENSG00000122641.9	7:41724712-41742706	INHBA	4921.10	4119.33	4119.33	0.11	0.64
ENSG00000163083.5	2:121103719-121109384	INHBB	142.87	62.41	62.41	0.40	0.12
ENSG00000133703.7	12:25357723-25403870	KRAS	1765.05	1705.98	1705.98	-0.05	0.82
ENSG00000169032.5	15:66679155-66784650	MAP2K1	3557.88	2975.07	2975.07	0.11	0.64
ENSG00000126934.9	19:4090319-4124126	MAP2K2	5918.22	5709.24	5709.24	-0.05	0.81
ENSG00000034152.14	17:21187984-21218552	MAP2K3	2502.22	1288.79	1288.79	0.54	0.03
ENSG00000108984.9	17:67410839-67539472	MAP2K6	41.67	36.13	36.13	0.02	0.92
ENSG00000104814.8	19:39078281-39109522	MAP4K1	18.85	7.66	7.66	0.12	0.46
ENSG00000185386.10	22:50702142-50709196	MAPK11	520.88	489.46	489.46	-0.02	0.93
ENSG00000188130.9	22:50683879-50700254	MAPK12	1120.15	1113.60	1113.60	-0.08	0.73
ENSG00000156711.12	6:36095586-36107842	MAPK13	427.62	341.63	341.63	0.13	0.60
ENSG00000112062.6	6:35995488-36079013	MAPK14	3368.38	2869.95	2869.95	0.09	0.70
ENSG00000102882.7	16:30125426-30134827	MAPK3	3481.48	3082.38	3082.38	0.04	0.85
ENSG00000107643.11	10:49514698-49647403	MAPK8	1331.48	1226.38	1226.38	0.00	1.00
ENSG00000183072.9	5:172659112-172662360	NKX2-5	40.68	28.47	28.47	0.09	0.68
ENSG00000156574.5	10:72192071-72207707	NODAL	0.99	0.00	0.00	0.01	0.72
ENSG00000105229.2	19:4007644-4039384	PIAS4	596.29	552.97	552.97	-0.01	0.98
ENSG00000124225.11	20:56223448-56286592	PMEPA1	306.58	266.08	266.08	0.05	0.84
ENSG00000132155.7	3:12625100-12705725	RAF1	4991.55	4660.25	4660.25	-0.02	0.94
ENSG00000144118.9	2:120997640-121052289	RALB	2090.48	1853.81	1853.81	0.04	0.86
ENSG00000125249.6	13:98086476-98121382	RAP2A	2122.23	1721.31	1721.31	0.14	0.55
ENSG00000181467.2	3:152880029-152886265	RAP2B	1123.12	981.10	981.10	0.06	0.81
ENSG00000108551.4	17:17397751-17399709	RASD1	4595.68	2385.97	2385.97	0.54	0.03
ENSG00000100302.6	22:35936915-35950048	RASD2	19.84	8.76	8.76	0.12	0.49
ENSG00000157450.11	15:59157374-59389618	RNF111	1076.49	1066.51	1066.51	-0.08	0.74
ENSG00000126458.3	19:50138549-50143458	RRAS	5200.89	4522.28	4522.28	0.06	0.78
ENSG00000133818.8	11:14299472-14386052	RRAS2	5177.08	4315.33	4315.33	0.11	0.62
ENSG00000124813.16	6:45295894-45632086	RUNX2	1571.58	1088.41	1088.41	0.29	0.23
ENSG00000106366.7	7:100770370-100782547	SERPINE 1	450356.41	232430.90	232430.90	0.56	0.03
ENSG00000157933.9	1:2160134-2241558	SKI	3300.91	2959.74	2959.74	0.03	0.90
ENSG00000175387.11	18:45357922-45457515	SMAD2	3688.84	3581.69	3581.69	-0.06	0.79
ENSG00000166949.11	15:67356101-67487533	SMAD3	2107.34	1427.86	1427.86	0.32	0.19
ENSG00000141646.9	18:48494410-48611415	SMAD4	3683.88	3628.77	3628.77	-0.08	0.74
ENSG00000137834.10	15:66994566-67074338	SMAD6	287.73	141.25	141.25	0.45	0.09
ENSG00000101665.4	18:46446223-46477081	SMAD7	1509.07	1066.51	1066.51	0.27	0.26
ENSG00000120693.9	13:37418968-37494902	SMAD9	779.84	476.32	476.32	0.38	0.14
ENSG00000198742.5	7:98625061-98741723	SMURF1	1728.34	1639.19	1639.19	-0.03	0.89
ENSG00000108854.11	17:62538413-62658186	SMURF2	3026.08	2635.62	2635.62	0.06	0.79
ENSG00000115904.8	2:39208537-39351486	SOS1	1825.57	1675.32	1675.32	0.00	0.99
ENSG00000100485.7	14:50583847-50698276	SOS2	1720.40	1439.90	1439.90	0.10	0.66
ENSG00000100324.9	22:39795746-39833065	TAB1	749.08	581.44	581.44	0.17	0.49
ENSG00000068323.12	X:48886242-48901012	TFE3	3788.06	3317.80	3317.80	0.06	0.81

ENSG00000105329.5	19:41807492-41859816	TGFB1	4097.61	2625.77	2625.77	0.37	0.12
ENSG00000119699.3	14:76424442-76449334	TGFB3	82.35	59.13	59.13	0.13	0.61
ENSG00000163513.13	3:30647994-30735634	TGFBR2	10561.52	8685.40	8685.40	0.13	0.57
ENSG00000115297.9	2:74740590-74744274	TLX2	21.83	17.52	17.52	0.03	0.87
ENSG00000175104.10	11:36508577-36531822	TRAF6	502.03	401.86	401.86	0.13	0.60
ENSG00000157077.10	1:52608046-52812358	ZFYVE9	1559.67	1442.09	1442.09	0.00	0.98
ENSG00000102935.7	16:49521435-49891830	ZNF423	50.60	48.18	48.18	-0.01	0.95

NOTCH SIGNALING PATHWAT

Ensembl	Location	Symbol	B4_0.y	B4_1.y	B4_1.y	log2Fold Change	pvalue
ENSG00000117362.8	1:150237804-150241980	APH1A	4165.08	4006.54	4006.54	-0.05	0.83
ENSG00000138613.9	15:63568217-63601325	APH1B	1278.89	1202.29	1202.29	-0.02	0.92
ENSG00000198719.7	6:170591294-170599561	DLL1	942.55	627.43	627.43	0.32	0.20
ENSG00000090932.6	19:39989535-39999121	DLL3	19.84	16.42	16.42	0.02	0.91
ENSG00000128917.5	15:41221538-41231237	DLL4	18.85	17.52	17.52	0.00	0.99
ENSG00000091073.15	7:76090993-76135312	DTX2	313.52	232.14	232.14	0.19	0.47
ENSG00000178498.11	12:57998405-58003587	DTX3	762.97	631.81	631.81	0.11	0.66
ENSG00000110042.3	11:58938903-58976060	DTX4	55.56	38.32	38.32	0.12	0.62
ENSG00000197921.5	1:2460184-2461684	HES5	8.93	5.47	5.47	0.04	0.77
ENSG00000179111.4	17:8023908-8027410	HES7	62.51	52.56	52.56	0.04	0.86
ENSG00000164683.12	8:80676245-80680098	HEY1	1014.98	790.58	790.58	0.17	0.48
ENSG00000135547.4	6:126068810-126082415	HEY2	13.89	13.14	13.14	0.00	0.98
ENSG00000106003.8	7:2552163-2568811	LFNG	22.82	10.95	10.95	0.12	0.50
ENSG00000105695.10	19:35783028-35804707	MAG	142.87	110.59	110.59	0.12	0.65
ENSG00000161021.7	5:179159851-179223512	MAML1	1264.01	1181.49	1181.49	-0.02	0.94
ENSG00000184384.9	11:95709762-96076344	MAML2	1815.65	1454.14	1454.14	0.15	0.52
ENSG00000196782.8	4:140637907-141075338	MAML3	208.35	140.16	140.16	0.23	0.38
ENSG00000100060.13	22:37865101-37882439	MFNG	11.91	4.38	4.38	0.09	0.52
ENSG00000134250.13	1:120454176-120612240	NOTCH2	6344.85	5544.99	5544.99	0.06	0.79
ENSG00000074181.4	19:15270444-15311792	NOTCH3	3270.15	1942.50	1942.50	0.44	0.08
ENSG00000133961.15	14:73741815-73930348	NUMB	3509.26	3254.29	3254.29	-0.01	0.97
ENSG00000080815.14	14:73603126-73690399	PSEN1	3346.55	3431.68	3431.68	-0.12	0.60
ENSG00000168214.16	4:26165077-26436541	RBPJ	3556.89	2982.73	2982.73	0.10	0.65
ENSG00000270408.1	20:10625847-10627014	JAG1	0.99	0.00	0.00	0.01	0.72

PDGF SIGNALING PATHWAY

Ensembl	Location	Symbol	B4_0.y	B4_1.y	B4_1.y	log2Fold Change	pvalue
ENSG00000097007.13	9:133589333-133763062	ABL1	7047.30	6515.15	6515.15	0.00	0.98
ENSG00000143727.11	2:264140-278283	ACP1	3475.53	3424.01	3424.01	-0.08	0.74
ENSG00000105974.7	7:116164839-116201233	CAV1	16739.69	13841.68	13841.68	0.12	0.59
ENSG00000167193.7	17:1323983-1366456	CRK	4332.75	4123.71	4123.71	-0.04	0.87
ENSG00000099942.8	22:21271714-21308037	CRKL	2765.14	2746.22	2746.22	-0.08	0.71
ENSG00000101266.12	20:459116-524465	CSNK2A1	6567.09	5743.18	5743.18	0.06	0.80
ENSG00000070770.4	16:58191811-58231824	CSNK2A2	3916.05	3607.97	3607.97	0.00	1.00
ENSG00000204435.9	6:31633013-31638120	CSNK2B	209.35	111.69	111.69	0.37	0.16
ENSG00000055332.12	2:37326353-37384208	EIF2AK2	3594.59	3593.73	3593.73	-0.09	0.68
ENSG00000126767.13	X:47494920-47510003	ELK1	2689.74	2279.75	2279.75	0.09	0.69
ENSG00000187682.1	X:48687283-48688548	ERAS	0.99	0.00	0.00	0.01	0.72

ENSG00000170345.5	14:75745477-75748933	FOS	64318.63	58356.01	58356.01	0.02	0.94
ENSG00000204084.8	1:38326369-38412729	INPP5B	1146.93	1099.36	1099.36	-0.04	0.85
ENSG00000198825.7	10:121485609-121588652	INPP5F	967.35	651.51	651.51	0.31	0.22
ENSG00000185133.9	22:31518717-31530682	INPP5J	96.24	79.93	79.93	0.06	0.82
ENSG00000165458.9	11:71934745-71950149	INPPL1	4062.89	3722.94	3722.94	0.01	0.98
ENSG00000096968.8	9:4985033-5128183	JAK2	1150.90	1098.27	1098.27	-0.04	0.87
ENSG00000105639.14	19:17935589-17958880	JAK3	769.91	584.72	584.72	0.19	0.44
ENSG00000133703.7	12:25357723-25403870	KRAS	1765.05	1705.98	1705.98	-0.05	0.82
ENSG00000169032.5	15:66679155-66784650	MAP2K1	3557.88	2975.07	2975.07	0.11	0.64
ENSG00000126934.9	19:4090319-4124126	MAP2K2	5918.22	5709.24	5709.24	-0.05	0.81
ENSG00000095015.5	5:56111401-56191979	MAP3K1	506.00	482.89	482.89	-0.04	0.89
ENSG00000102882.7	16:30125426-30134827	MAPK3	3481.48	3082.38	3082.38	0.04	0.85
ENSG00000107643.11	10:49514698-49647403	MAPK8	1331.48	1226.38	1226.38	0.00	1.00
ENSG00000136997.10	8:128747680-128753674	MYC	18267.61	15077.91	15077.91	0.12	0.58
ENSG00000100311.12	22:39619364-39640756	PDGFB	57.55	37.23	37.23	0.14	0.55
ENSG00000134853.7	4:55095264-55164414	PDGFRA	29438.32	13944.61	13944.61	0.62	0.02
ENSG00000113721.9	5:149493400-149535435	PDGFRB	3394.17	3035.29	3035.29	0.03	0.89
ENSG00000011405.9	11:17099277-17229530	PIK3C2A	8499.82	6329.00	6329.00	0.23	0.31
ENSG00000133056.9	1:204391756-204463852	PIK3C2B	56.55	29.56	29.56	0.21	0.37
ENSG00000139144.5	12:18400548-18801348	PIK3C2G	4.96	1.09	1.09	0.05	0.57
ENSG00000078142.7	18:39535171-39667794	PIK3C3	3910.09	3323.27	3323.27	0.09	0.69
ENSG00000121879.3	3:178865902-178957881	PIK3CA	2890.16	2322.46	2322.46	0.15	0.52
ENSG00000051382.4	3:138372860-138553780	PIK3CB	970.33	884.75	884.75	0.01	0.97
ENSG00000171608.11	1:9711790-9789172	PIK3CD	666.73	573.77	573.77	0.07	0.78
ENSG00000105851.6	7:106505723-106547590	PIK3CG	1.98	0.00	0.00	0.02	0.61
ENSG00000117461.10	1:46505812-46642160	PIK3R3	203.39	192.72	192.72	-0.02	0.93
ENSG00000196455.3	3:130397779-130465673	PIK3R4	967.35	909.93	909.93	-0.02	0.92
ENSG00000174083.13	17:8706041-8770994	PIK3R6	0.99	0.00	0.00	0.01	0.72
ENSG00000124181.10	20:39765600-39811629	PLCG1	2857.42	2399.11	2399.11	0.10	0.66
ENSG00000154229.7	17:64298754-64806861	PRKCA	4473.64	4136.85	4136.85	-0.01	0.98
ENSG00000166501.8	16:23847322-24231932	PRKCB	25.80	18.61	18.61	0.06	0.77
ENSG00000132155.7	3:12625100-12705725	RAF1	4991.55	4660.25	4660.25	-0.02	0.94
ENSG00000144118.9	2:120997640-121052289	RALB	2090.48	1853.81	1853.81	0.04	0.86
ENSG00000125249.6	13:98086476-98121382	RAP2A	2122.23	1721.31	1721.31	0.14	0.55
ENSG00000181467.2	3:152880029-152886265	RAP2B	1123.12	981.10	981.10	0.06	0.81
ENSG00000145715.10	5:86563705-86687748	RASA1	1617.22	1136.59	1136.59	0.28	0.25
ENSG00000108551.4	17:17397751-17399709	RASD1	4595.68	2385.97	2385.97	0.54	0.03
ENSG00000100302.6	22:35936915-35950048	RASD2	19.84	8.76	8.76	0.12	0.49
ENSG00000126458.3	19:50138549-50143458	RRAS	5200.89	4522.28	4522.28	0.06	0.78
ENSG00000133818.8	11:14299472-14386052	RRAS2	5177.08	4315.33	4315.33	0.11	0.62
ENSG00000160691.14	1:154934774-154946871	SHC1	14114.44	10747.26	10747.26	0.21	0.35
ENSG00000115904.8	2:39208537-39351486	SOS1	1825.57	1675.32	1675.32	0.00	0.99
ENSG00000100485.7	14:50583847-50698276	SOS2	1720.40	1439.90	1439.90	0.10	0.66
ENSG00000176170.9	17:74372665-74383941	SPHK1	4000.38	3431.68	3431.68	0.08	0.73
ENSG00000063176.11	19:49122548-49133974	SPHK2	323.44	302.22	302.22	-0.01	0.96
ENSG00000197122.7	20:35973088-36034453	SRC	1548.76	1372.01	1372.01	0.04	0.86
ENSG00000115415.14	2:191829084-191885686	STAT1	4652.23	5882.25	5882.25	-0.34	0.16
ENSG00000159082.13	21:34001069-34100359	SYNJ1	234.15	181.77	181.77	0.14	0.60
ENSG00000078269.9	6:158402888-158520208	SYNJ2	1155.86	965.77	965.77	0.10	0.67
ENSG00000105397.9	19:10461209-10491352	TYK2	2239.30	1973.16	1973.16	0.05	0.83
