# Supplementary material for: Leptin secreted from testicular microenvironment modulates hedgehog signaling to augment the endogenous function of Leydig cells
Source: Cell Death Dis. 2022 Mar 4;13(3):208. doi: 10.1038/s41419-022-04658-3 (PMC8897450; doi:10.1038/s41419-022-04658-3)

Supplementary Material. Showing full western blots for Figure 1C, 2F, Supplementary Figure 4A, 4F and 6 respectively.

Raw Blot_Fig 1C

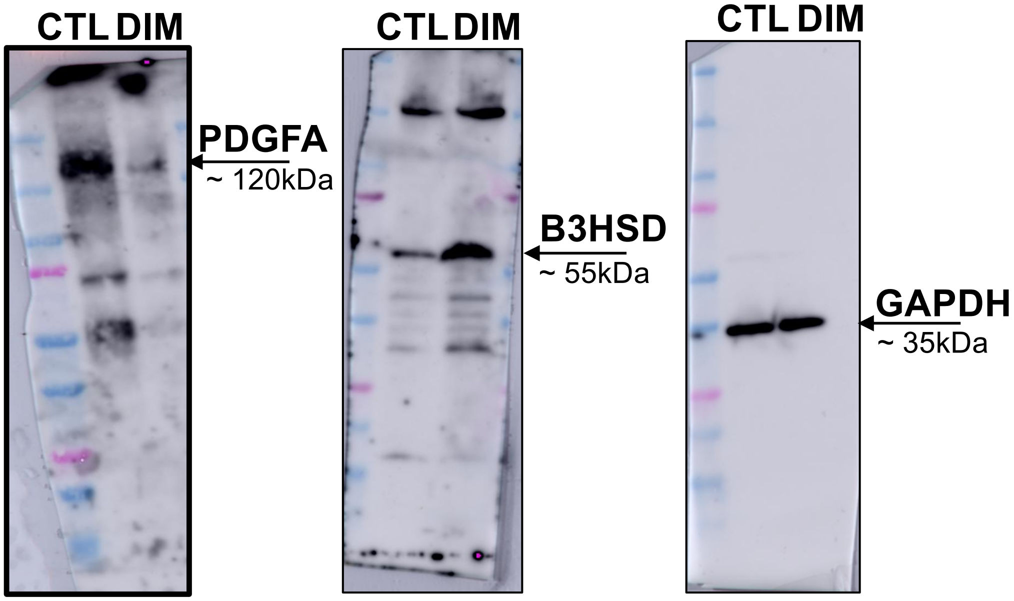

Raw Blot_Fig 2F

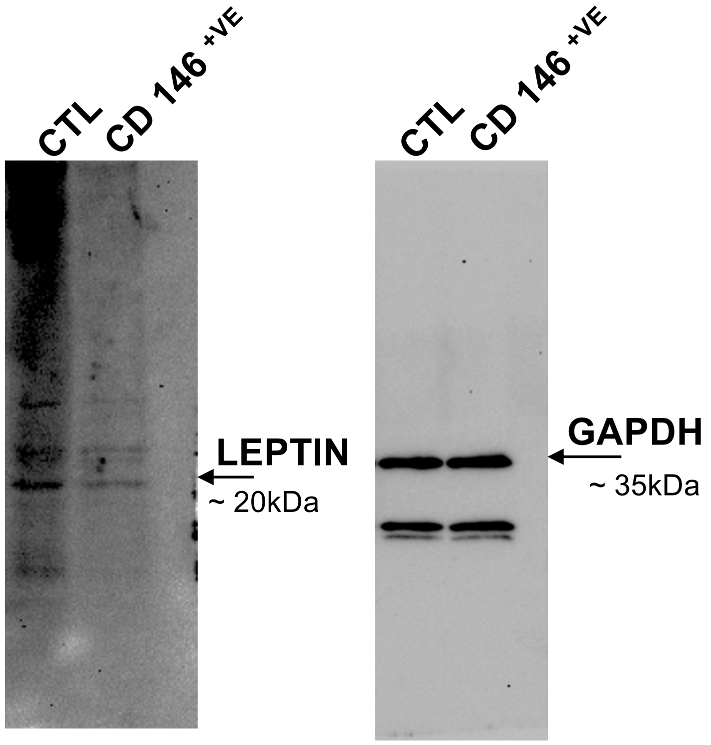

Raw Blot_Supp Fig 4A

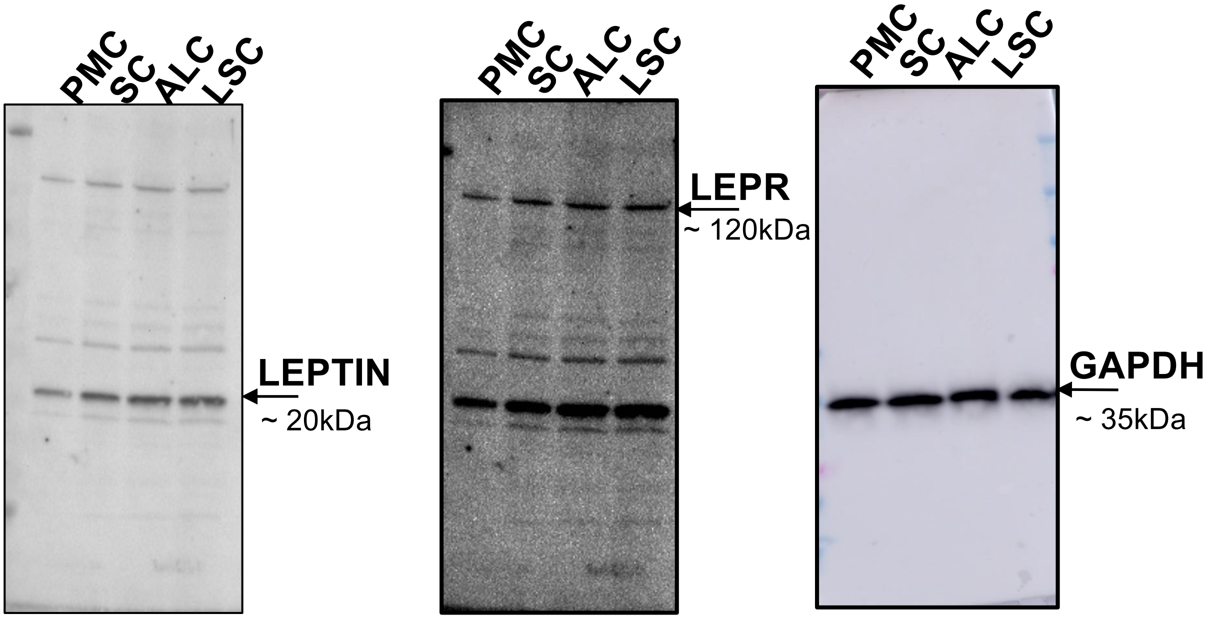

Raw Blot_Supp Fig 4F

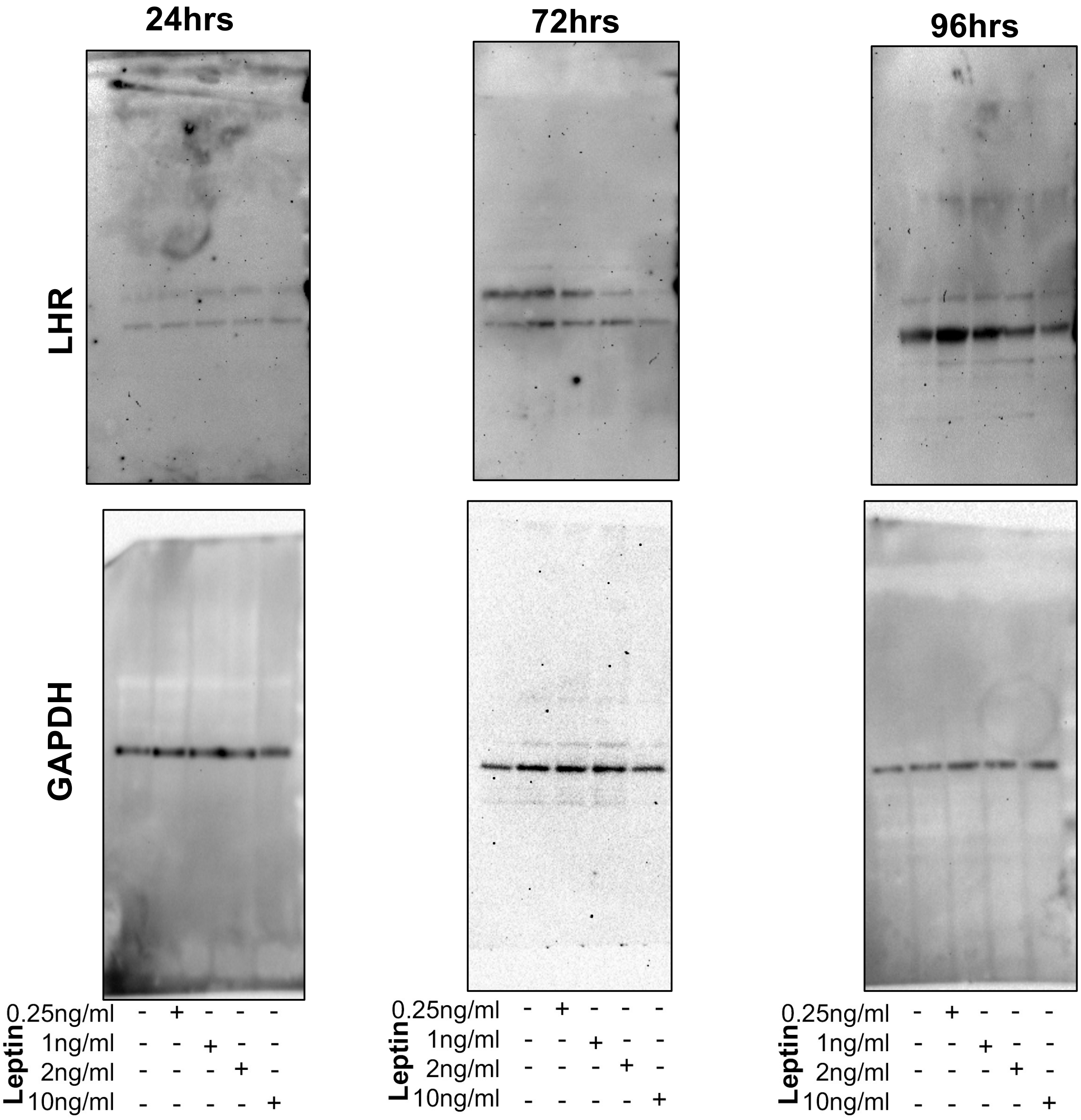

Raw Blot_Supp Fig 6

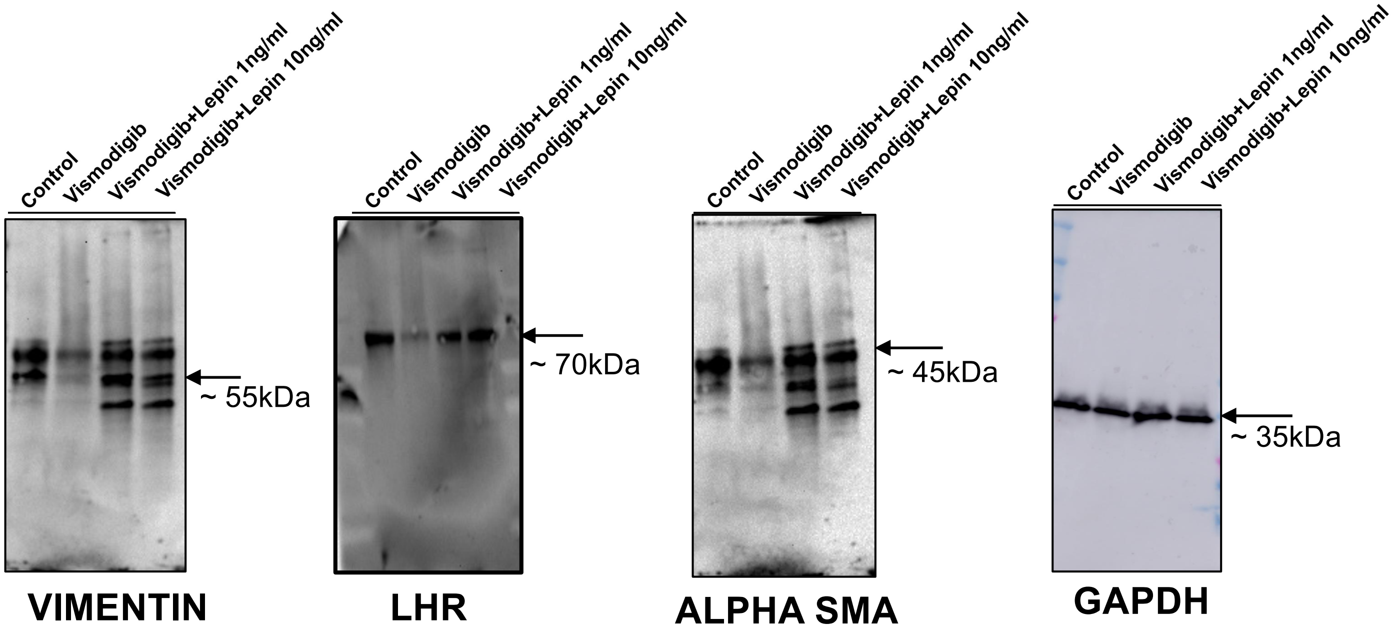

Supplement: Supplementary file 2 — Supplementary Material 2 [file 41419_2022_4658_MOESM2_ESM.pdf]
